# Pipeline validation for the identification of antimicrobial-resistant genes in carbapenem-resistant *Klebsiella pneumoniae*

**DOI:** 10.1038/s41598-023-42154-6

**Published:** 2023-09-14

**Authors:** Andressa de Almeida Vieira, Bruna Candia Piccoli, Thaís Regina y Castro, Bruna Campestrini Casarin, Luiza Funck Tessele, Roberta Cristina Ruedas Martins, Alexandre Vargas Schwarzbold, Priscila de Arruda Trindade

**Affiliations:** 1 Laboratório de Biologia Molecular e Bioinformática Aplicada à Microbiologia Clínica, Programa de Pós-Graduação em Ciências Farmacêuticas, Universidade Federal de Santa Maria, Santa Maria, 97105-900 Brazil; 2 Laboratório de Parasitologia Médica (LIM-46), Departamento de Doenças Infecciosas e Parasitárias, Instituto de Medicina Tropical da Universidade de São Paulo, Faculdade de Medicina da Universidade de São Paulo, São Paulo, 01246-903 Brazil; 3 Departamento de Clínica Médica, Universidade Federal de Santa Maria, Rio Grande do Sul, Brazil

**Keywords:** Computational biology and bioinformatics, Microbiology, Antimicrobials, Bacteria

## Abstract

Antimicrobial-resistant *Klebsiella pneumoniae* is a global threat to healthcare and an important cause of nosocomial infections. Antimicrobial resistance causes prolonged treatment periods, high mortality rates, and economic impacts. Whole Genome Sequencing (WGS) has been used in laboratory diagnosis, but there is limited evidence about pipeline validation to parse generated data. Thus, the present study aimed to validate a bioinformatics pipeline for the identification of antimicrobial resistance genes from carbapenem-resistant *K. pneumoniae* WGS. Sequences were obtained from a publicly available database, trimmed, de novo assembled, mapped to the *K. pneumoniae* reference genome, and annotated. Contigs were submitted to different tools for bacterial (Kraken2 and SpeciesFinder) and antimicrobial resistance gene identification (ResFinder and ABRicate). We analyzed 201 K*. pneumoniae* genomes. In the bacterial identification by Kraken2, all samples were correctly identified, and in SpeciesFinder, 92.54% were correctly identified as *K. pneumoniae*, 6.96% erroneously as *Pseudomonas aeruginosa*, and 0.5% erroneously as *Citrobacter freundii*. ResFinder found a greater number of antimicrobial resistance genes than ABRicate; however, many were identified more than once in the same sample. All tools presented 100% repeatability and reproducibility and > 75% performance in other metrics. Kraken2 was more assertive in recognizing bacterial species, and SpeciesFinder may need improvements.

## Introduction

Widespread use of antimicrobials has generated microorganisms' selective pressure^1,2^. The emergence and spread of antimicrobial-resistant bacteria become a threat to public health^3^. One of the most worrying pathogens is *Klebsiella pneumoniae*. This microorganism belongs to the *Enterobacterales* order and *Enterobacteriaceae* family, which are composed of gram-negative encapsulated, non-spore-forming, and rod-shaped bacteria^4–6^. In human hosts, it can constitute the normal enteric microbiota. It can also infect the respiratory system, endocardium, surgical site wounds, reach the bloodstream, and cause sepsis^7^. Neonates, the elderly, and immunocompromised hospitalized patients present a worse prognosis^8,9^. It is capable of causing serious community-acquired infections especially due to hypervirulent strains^7^.

β-lactam antimicrobials (carbapenems, cephalosporins, and monobactams) present a β-lactam ring in their molecular structure, which inhibits the transpeptidases. Consequently, they inhibit cell wall synthesis, leading to bacterial death^10^. *K. pneumoniae*'s accessory genome acquired genes encoding β-lactamases as a resistance mechanism to hydrolyze the β-lactam ring^7,11^. The first reported gene was Carbapenem-hydrolyzing beta-lactamase KPC (*bla*_KPC_) in 1996^12,13^. *bla*_KPC_ became stable in the accessory genome of some *K. pneumoniae* strains^7,11,12^. Since then, other genes encoding β-lactamases have been identified, such as oxacillinases (*bla*_OXA_), and metallo-β-lactamases (*bla*_NDM_, *bla*_IMP_, and *bla*_VIM_)^7,11,14^.

Antimicrobial resistance is complex, multifactorial, and causes prolonged treatment periods, high mortality rates, and economic impacts^1,15^. Available molecular tests are unable to detect emerging genetic characteristics of pathogens. To ensure successful treatment, recovery, and patient safety, the identification and characterization of microorganisms causing infections are essential^16,17^. Whole Genome Sequencing (WGS) has the ability to replace traditional molecular techniques as it provides benefits in terms of higher resolution, speed, reduced cost, and numerous additional information such as species, strain type, resistance, and virulence profiles^18,19^. Analyzing and interpreting genome-scale data pose challenges due to the volume and complexity of the data^20^. Thus, the objective of this study is to validate a bioinformatics pipeline for in silico analysis of WGS of carbapenem-resistant *K. pneumoniae* isolates to produce standardized data that will enable interlaboratory comparisons.

## Results

We analyzed 201 K*. pneumoniae* genomes to validate the pipeline for predicting antimicrobial resistance genes, especially carbapenems. For this purpose, we took advantage of seven BioProjects with carbapenem-resistant *K. pneumoniae* SRAs available on the National Center for Biotechnology Information (NCBI) platform. *K. pneumoniae* strain ATCC 35657 (PRJNA279657), lacking carbapenem-resistance genes, was used as a negative control. We trimmed, de novo assembled, ordered, and annotated the SRAs. De novo assembly and mapping quality metrics are listed in Table 1. A high percentage of genome coverage (mean of 93.8%) and depth (mean of 125.5x) were obtained.

**Table 1:** De novo assembly quality metrics. Results were shown as mean.

Metric	1	2	3	4	5	6	7
Reads	1,667,674.91	1,091,649.50	1,593,738.60	1,719,599.30	1,385,878.40	9,052,915.20	5,313,384
Coverage (%)	94.6	94.5	94.8	96.2	92.9	92.6	90.9
Depth (x)	67.9	45.6	61.9	75.9	48.4	327.5	251.2
Contig number	142	99.6	110	96.5	79.3	47.3	17
Length of the longest contig (nt)	482,279.78	670,750.10	663,956.60	623,432.60	674,034.90	1,155,637	2,052,661
Total length (nt)	5,634,605.77	5,707,949.80	5,679,227.60	5,732,894.70	5,613,962.30	5,648,880	5,349,908
GC content (%)	57.1	57	57	57	57.1	57.1	57.4
N50	165,681.46	241,806	223,144	222,369	298,608	453,211	847,522

**Figure 1:**
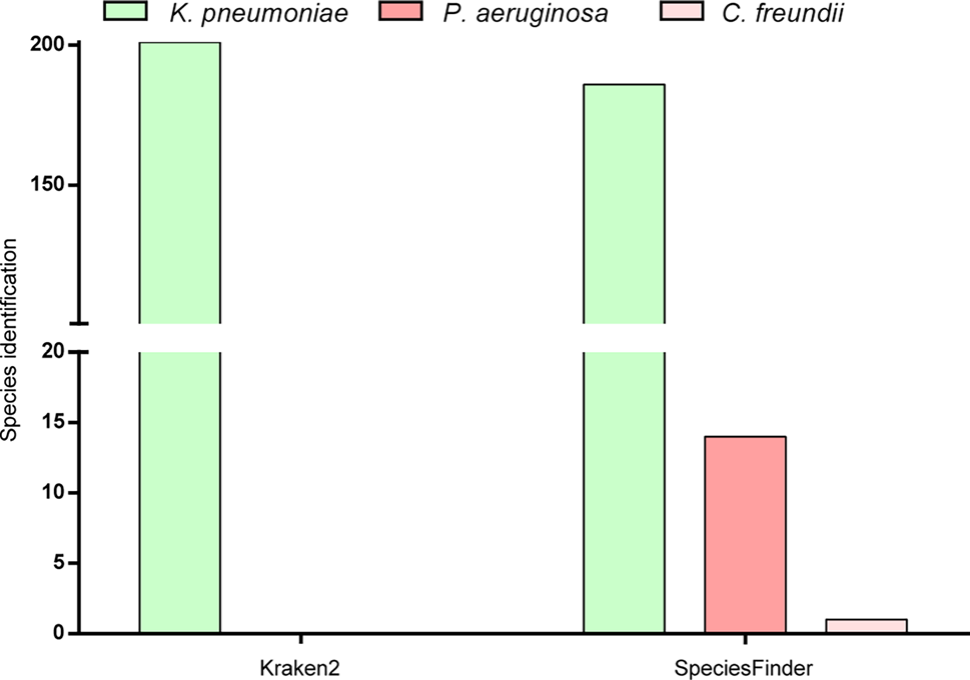
Bacteria identified by Kraken and SpeciesFinder databases.

Kraken2 and SpeciesFinder tools were used for bacterial identification. For Kraken2, all samples (100%) were identified correctly, and for SpeciesFinder, 92.54% (186) were identified as *K. pneumoniae*, 6.96% (14) as *Pseudomonas aeruginosa*, and 0.5% (1) as *Citrobacter freundii* (Fig. 1 and Table S1). Both tools obtained 100% reproducibility and repeatability (Table 2). The other validation metrics could not be calculated due to the lack of adequate definitions for the analysis.

**Table 2:** Repeatability and reproducibility of bacterial identification from Kraken2 and SpeciesFinder tools.

BioProject	Kraken2		SpeciesFinder	
	Repeatability (%)	Reproducibility	Repeatability (%)	Reproducibility
1	100	–	100	–
2	100	100%	100	100%
3	100	–	100	–
4	100	–	100	–
5	100	–	100	–
6	100	–	100	–
7	100	–	100	–

**Figure 2:**
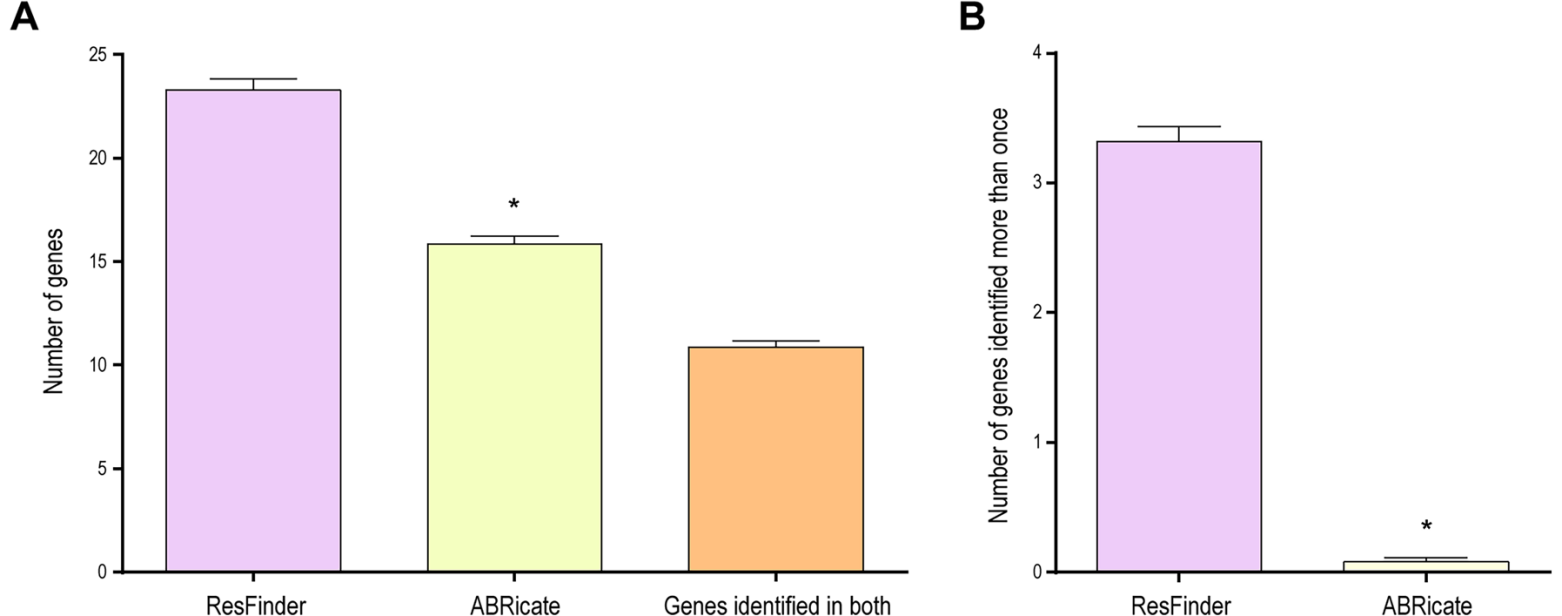
Resistance genes found by ResFinder and ABRicate databases in 201 SRAs (**A**). Same gene was indicated more than once in each sample (**B**). Results were presented as mean ± SEM and analyzed by Student's t test. * means statistical difference from the ResFinder group (p ≤ 0.05).

ResFinder and ABRicate tools were used for identifying antimicrobial resistance genes. We evaluated 273 antimicrobial resistance genes, among them twelve are specific to carbapenems, *i.e.*, *bla*_KPC-2_, *bla*_KPC-3_, *bla*_NDM-1_, *bla*_NDM-7_, *bla*_OXA-48_, *bla*_OXA-162_, *bla*_OXA-181_, *bla*_OXA-232_, *bla*_OXA-245_, *bla*_VIM-1_, *bla*_VIM-19_, and *bla*_VIM-27_ (Table S2). ResFinder identified a higher number of antimicrobial resistance genes, corresponding to 23.27 ± 0.56, compared to 15.85 ± 0.39 (ABRicate) (Fig. 2A and Table S3). Of these, 55% were found by both tools. It is important to note that, in all samples, ResFinder indicated up to 6 × the same gene (Fig. 2B). ABRicate only showed duplicated genes in eight samples. Although ResFinder found a greater number of genes, this value was distorted due to gene duplication.

**Figure 3:**
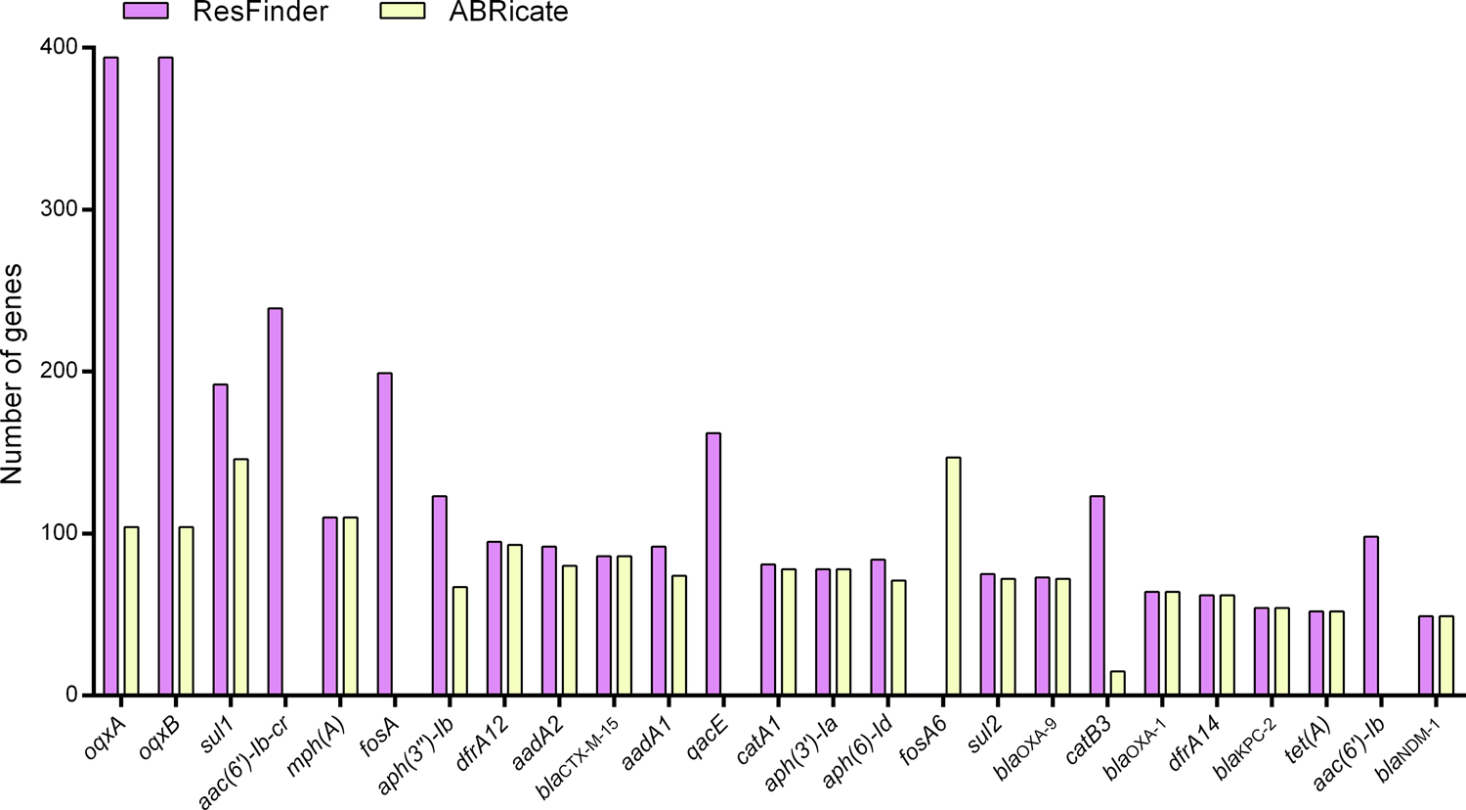
Twenty-five genes most frequently identified by ResFinder and ABRicate databases.

The genes most frequently identified by ResFinder in the 201 samples were *oqxA* and *oqxB* genes (394 times) (Fig. 3). Differently, *fosA6* gene, followed by *sul1* gene, were the genes most identified by ABRicate. Among the 25 genes most frequently identified by the tools, *fosA6* gene was found only by ABRicate, and *aac(6')-Ib-cr*, *fosA*, *qacE* gene, and *aac(6')-Ib* gene were found only by ResFinder. We only found one carbapenem resistance gene (*bla*_KPC-2_).

**Figure 4:**
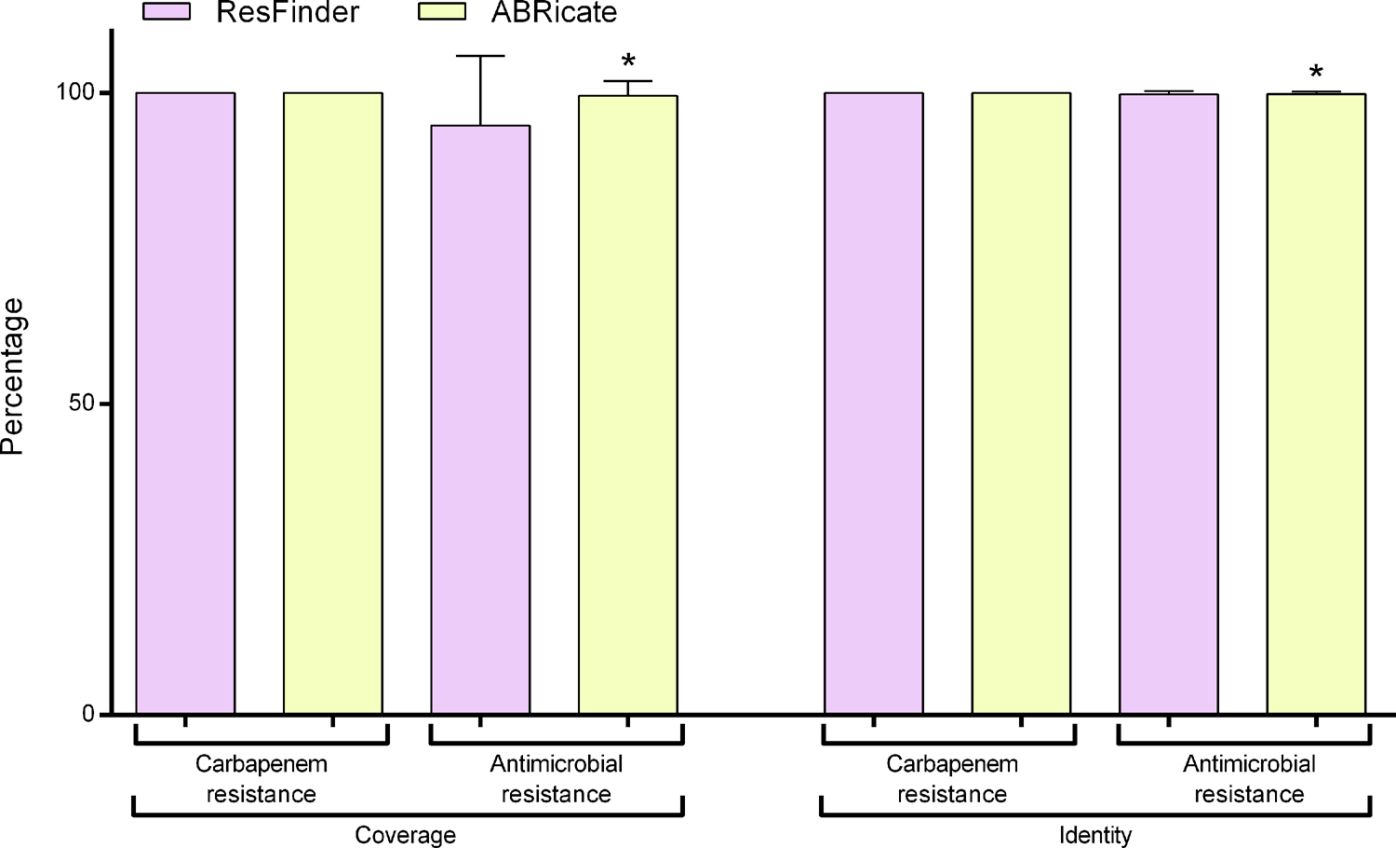
Percent coverage and identity of antimicrobial resistance genes found by ResFinder and ABRicate databases. Results were presented as mean ± SEM and analyzed by Student's t test. * means statistical difference from the ResFinder group (p ≤ 0.05).

Carbapenem-resistant genes identified by ResFinder and ABRicate showed similar coverage and identity percentages (Fig. 4). When we consider all antimicrobial resistance genes identified, ABRicate had the highest coverage percentage [t(7165) = 22.6; p < 0.0001] and identity [t(7165) = 3.784; p = 0.0002)]. These results indicate that, probably, genes were present in the samples and were correctly identified with greater reliability by ABRicate.

Pipeline validation metrics for ABRicate and ResFinder tools, highlighting carbapenem resistance genes and all antimicrobial resistance genes, are shown in Table 3. Sequences were analyzed in triplicate on the same day to determine repeatability. Samples from BioProjects PRJNA292902/PRJNA292904, which had more than one technical replicate, were evaluated on alternate days to calculate reproducibility. Accuracy, precision, sensitivity, and specificity calculations were performed by comparing the results obtained with the reference sequence (RefSeq). ABRicate presented lower precision and sensitivity in BioProject 1 (PRJEB28660) when considering only the carbapenem resistance genes. However, when all antimicrobial resistance genes were evaluated, ResFinder showed lower percentages in 17 parameters (mainly related to accuracy, precision, sensitivity, and specificity) in five different BioProjects, compared to four parameters of ABRicate. These results indicate that ABRicate seems to be more suitable for antimicrobial resistance gene identification.

**Table 3:** Validation metrics of ABRicate and ResFinder tools for resistance genes.

Tool	Gene	Metric	1	2	3	4	5	6	7
ABRicate	Carbapenem resistance genes	Repeatability	100%	100%	100%	100%	100%	100%	100%
		Reproducibility	–	100%	–	–	–	–	–
		Accuracy	99.99%	99.99%	100%	100%	100%	99.98%	100%
		Precision	90.19%	95.45%	100%	100%	100%	100%	0
		Sensitivity	92.00%	82.89%	100%	100%	100%	55.55%	0
		Specificity	99.99%	99.99%	100%	100%	100%	100%	100%
	All antimicrobial resistance genes	Repeatability	100%	100%	100%	100%	100%	100%	100%
		Reproducibility	–	44.92%	–	–	–	–	–
		Accuracy	99.88%	99.88%	99.97%	99.94%	99.97%	99.85%	100%
		Precision	93.79%	97.76%	96.85%	95.95%	97.39%	100%	100%
		Sensitivity	74.28%	79.59%	96.63%	90.97%	97.90%	72.36%	100%
		Specificity	99.98%	99.99%	99.98%	99.98%	99.98%	100%	100%
ResFinder	Carbapenem resistance genes	Repeatability	100%	100%	100%	100%	100%	100%	100%
		Reproducibility	–	100%	–	–	–	–	–
		Accuracy	99.99%	99.99%	100%	100%	100%	99.98%	100%
		Precision	91.07%	95.45%	100%	100%	100%	100%	0
		Sensitivity	92.72%	82.89%	100%	100%	100%	55.55%	0
		Specificity	99.99%	99.99%	100%	100%	100%	100%	100%
	All antimicrobial resistance genes	Repeatability	100%	100%	100%	100%	100%	100%	100%
		Reproducibility	–	36.23%	–	–	–	–	–
		Accuracy	99.80%	99.79%	99.94%	99.86%	99.97%	99.84%	100%
		Precision	90.44%	89.12%	92.14%	90.96%	97.79%	100%	100%
		Sensitivity	72.88%	79.36%	96.75%	85.93%	98.51%	76.92%	100%
		Specificity	99.95%	99.93%	99.95%	99.94%	99.98%	100%	100%

**Figure 5:**
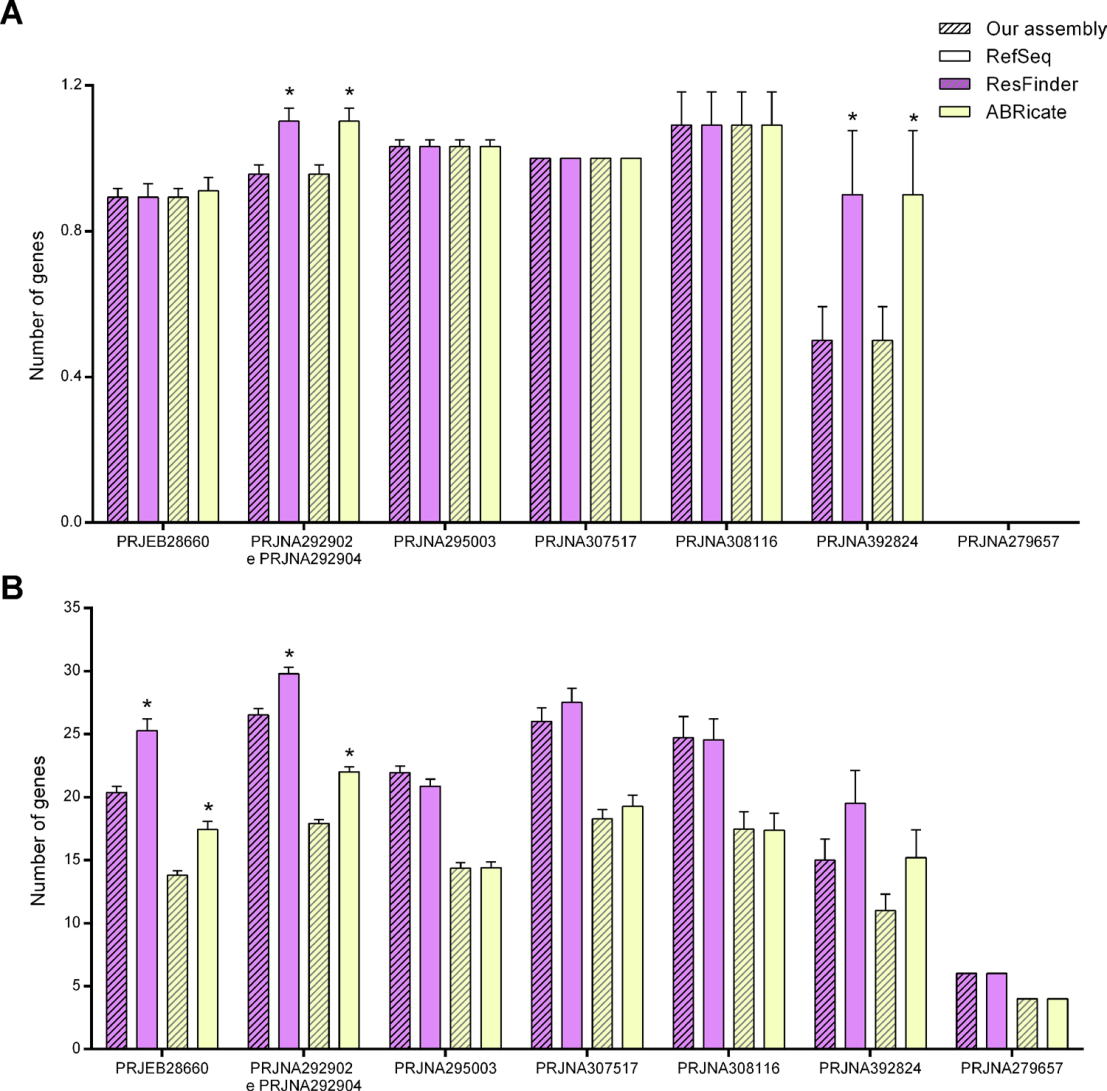
Resistance genes identified by ResFinder and ABRicate databases using the samples assembled using the pipeline described in this study and their RefSeq. Carbapenem resistance genes (**A**) and all antimicrobial resistance genes identified (**B**) by the databases in each bioproject. Results were presented as mean ± SEM and analyzed by Student's t test. * means statistical difference from our assembly (p ≤ 0.05).

We compared the number of genes identified by the samples assembled in this study with their respective RefSeqs (Fig. 5). As expected, no carbapenem resistance gene was identified in the negative control (PRJNA279657) (Fig. 5A). A higher number of carbapenem resistance genes were found in the RefSeqs of the BioProjects PRJNA292902/PRJNA292904 and PRJNA392824 than in the samples assembled using the pipeline described in this study, as identified by both tools (Fig. 5A). Similarly, more antimicrobial resistance genes were found in the RefSeqs of the PRJEB28660 and PRJNA292902/PRJNA292904 BioProjects, as shown in Fig. 5B. These results corroborate the lower sensitivity found in these BioProjects (Table 3). Performing a manual curation, we detected that, in the RefSeq, a greater number of genes were found because the same gene (same name and accession) was identified in the sample in more than one contig; in the same contig, but in different *loci*; or in the same contig and at the same *locus*, but with different accessions. These results indicated a high number of false negatives (FN), which affected the tool sensitivities.

**Figure 6:**
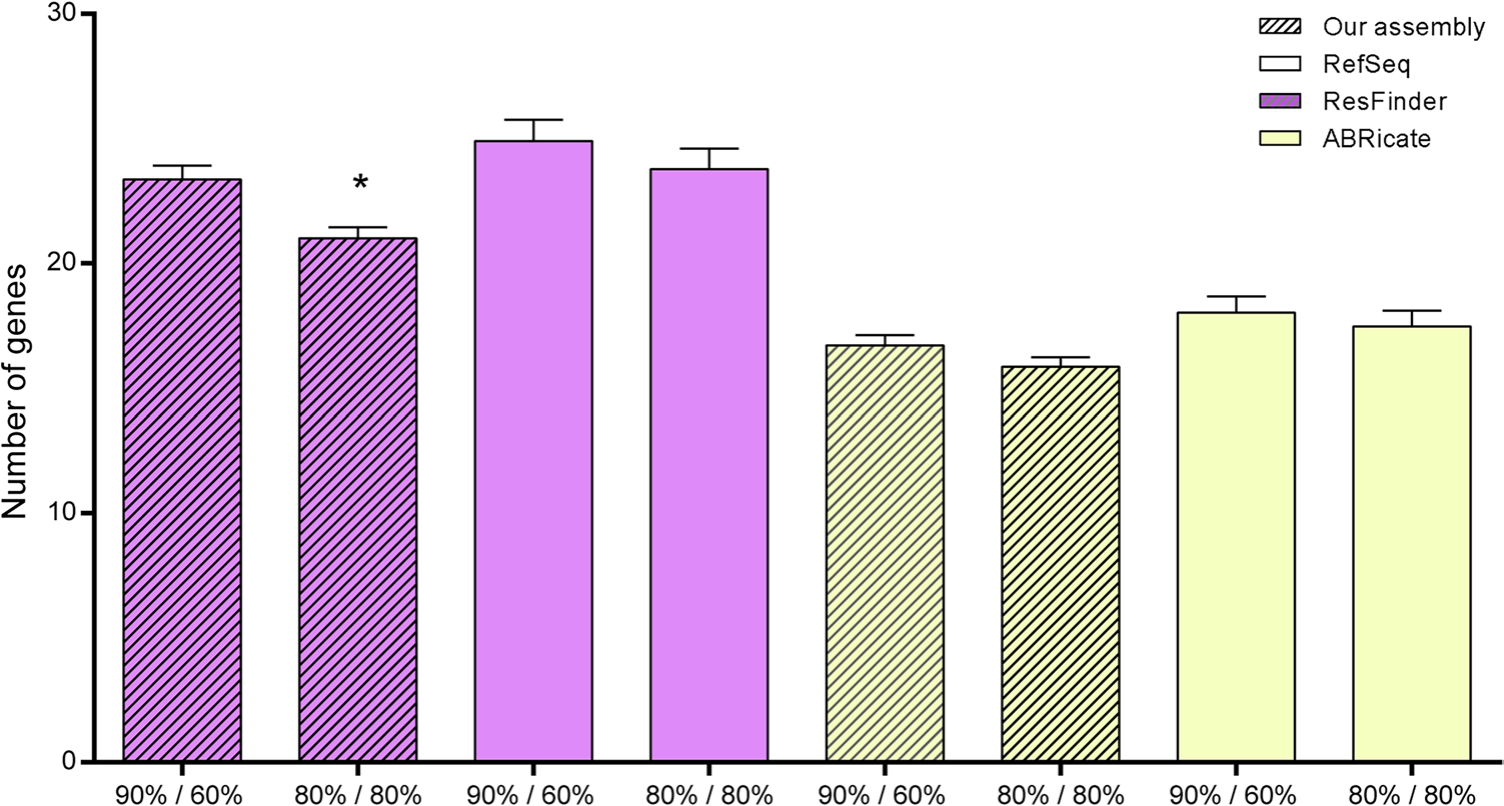
Resistance genes identified by ResFinder with BLAST parameters set at 80% identity and coverage (default parameters of ABRicate) and ABRicate with BLAST parameters set at 90% identity and 60% coverage (default parameters of ResFinder), using the samples assembled using the pipeline described in this study and their RefSeq. Results were presented as mean ± SEM and analyzed by Student's t test. * means statistical difference from 90% identity and 60% coverage (p ≤ 0.05).

We additionally evaluated the influence of the default parameters of Basic Local Alignment Search Tool (BLAST) on the performance of ABRicate and ResFinder. We identified antimicrobial resistance genes using ABRicate with parameters set at 90% identity and 60% coverage (default parameters of ResFinder), and for ResFinder, we employed parameters set at 80% identity and coverage (default parameters of ABRicate) (Fig. 6). ResFinder identified a greater number of antimicrobial resistance genes compared to ABRicate under both parameter settings, considering our assembly and the RefSeq dataset. When applying the criteria of 80% sequence identity and 80% coverage, ResFinder identified a reduced number of antimicrobial resistance genes in samples assembled using the pipeline described in this study [t(399) = 3.286; p = 0.0011]. However, the results were similar when using the RefSeq dataset (p > 0.05). ABRicate exhibited a statistically similar antimicrobial resistance gene number under both BLAST parameter settings.

## Discussion

In this study, we validated a bioinformatics pipeline for *K. pneumoniae* identification and the prediction of antimicrobial resistance genes in sequenced samples obtained from humans infected with this pathogen. The *K. pneumoniae* genome has approximately two thousand conserved genes^11,21^. It also presents an accessory genome consisting of genes located on chromosomes and plasmids that vary among isolates. *K. pneumoniae* has, on average, five to six thousand accessory genes^11^. These genes are acquired through horizontal transfer, as evidenced by the presence of genomic islands and mobile genetic elements. Accessory genes could encode virulence factors, enzymes, and antimicrobial resistance mechanisms, potentially worsening the prognosis of infected individuals^11^. Thus, identifying the infecting microorganism and its resistance genes is crucial for patient diagnosis and treatment.

We used the pipeline validation protocol described by Bogaerts et al.^19^. The authors performed the first bioinformatics pipeline validation for microbiological sequence isolates using *Neisseria meningitidis* as a model. Traditional metrics of repeatability, reproducibility, precision, sensitivity, and specificity were evaluated, adapted for WGS data. The dataset consisted of 131 sequences, divided into two subsets: the main subset (composed of 67 samples sequenced in triplicate) and the extended subset (composed of 64 sequenced samples publicly available on NCBI). In our study, we used 201 sequenced samples. Among them, 132 were single replicates used to calculate the repeatability, and 69 comprised three or four technical replicates, considered for both repeatability and reproducibility calculations.

Due to the range of bioinformatic approaches used to manipulate the data, three stages of analysis can lead to discrepant results: (i) sequencing quality, (ii) databases, or (iii) software used. Sample quality control is critical to improving sensitivity. High coverage (at least 90%) and depth (at least 30x) are also recommended. Values below the recommended thresholds can generate false positive (FP) results^22^. To minimize erroneous results, the pipeline contains a trimming step to remove poorly sequenced nucleotides, adapters, and short reads. The remaining reads were mapped against the reference genome, resulting in > 90% coverage and 45 × depth (Table 1).

After ensuring the read quality and optimal coverage and depth values, sequences were submitted to Kraken2 and SpeciesFinder to identify their bacterial species. Both tools showed high repeatability and reproducibility. Kraken2 correctly identified all sequences. SpeciesFinder identified 92.54% of the sequences as *K. pneumoniae* and the rest, erroneously, as *Pseudomonas aeruginosa* and *Citrobacter freundii*. The bacteria *C. freundii* and *K. pneumoniae* belong to the same family (*Enterobacteriaceae*)^23^. However, *P. aeruginosa* only shares the same class^24^, and it is counterintuitive that *K. pneumoniae* sequences were identified as *P. aeruginosa*. SpeciesFinder maps the contigs against the 16S rRNA sequence using the BLAST. The 16S rRNA corresponds to 0.1% of the microbial genome coding sequence^25^. We hypothesize that *P. aeruginosa* and *C. freundii* were identified in *K. pneumoniae* SRAs because mapping occurred in a small region of the genome, although the 16S rRNA is considered a highly conserved gene. Kraken2 performs a comprehensive genome analysis, mapping short genomic sequences (k-mers) in genomes present in its database and comparing them to a taxonomic tree to identify the common ancestor^26,27^. This could justify Kraken2's assertiveness in identifying species.

ResFinder and ABRicate were used to identify antimicrobial resistance genes. ResFinder identified a wide range of resistance genes in the analyzed sequences; however, ResFinder provides up to six copies of the same gene (Fig. 2A,B). These tools are composed of different gene variants and/or isoforms. Thus, the high percentage of identity among the sequences (> 90%) guarantees the correct gene identification^22^. In our study, we achieved > 99.8% identity and > 94.8% genomic coverage (Fig. 4). Doyle et al.,^22^, also found disagreements in the total number of genes associated with antimicrobial resistance, as well as in gene variants of pathogens resistant to carbapenems. These results show that the choice of a resistance gene identification tool can significantly impact the results.

ResFinder and ABRicate showed high repeatability and reproducibility when considering only the carbapenem resistance genes. Reproducibility was reduced to 44.92% (ABRicate) and 36.23% (ResFinder) when evaluating all antimicrobial resistance genes. Reproducibility is calculated by sequencing the same sample under different conditions. In this study, we used publicly available SRAs, some of which contained technical replicates. However, the exact sequencing conditions are not known, which is a limitation of our in silico study since we were unable to sequence the samples. The other performance metrics, including accuracy, precision, sensitivity, and specificity, were similar for both tools in the identification of carbapenem resistance genes. When we evaluated these parameters for the identification of all antimicrobial resistance genes, ABRicate showed better accuracy (mean of 97.39%) than ResFinder (mean of 93.88%). Bogaerts et al.^19^ found a performance of 100% in all metrics evaluated for ResFinder and NDARO tools. The identification of other resistance genes was also done, and the metrics showed > 70% performance, except for reproducibility (36.23%).

Sensitivity presented the lowest percentages (< 55%). It is calculated by comparing the number of genes found in the RefSeq with the number found in the consensus sequences. Resistance gene identification tools (ResFinder and ABRicate) found a greater number of genes in RefSeq than in the consensus sequences assembled by our pipeline. After performing manual curation, we realized that this higher number was related to gene duplication. Similarly, Kozyreva et al.^28^ used reference sequences from the US Food and Drug Administration (FDA)-CDC Antimicrobial Resistance (AR) Isolate Bank, previously evaluated with the ResFinder database. The authors found discrepancies in the detection of resistance genes between reference sequences and those assembled by them, leading to FP. The RefSeqs were trimmed and assembled differently from what was proposed by the pipeline, which may have influenced the identification of antimicrobial resistance genes. The difference in assembly software can alter or make it infeasible to identify a gene if it is divided into one or more contigs^29,30^. Also, the presence of duplicate genes in the tools leads to an overestimation of these genes^31^. After this manual curation, we considered that the de novo assembly proposed by our pipeline is adequate, as well as the sensitivity of the tools. It is important to notice the different BLAST default parameter settings between ABRicate and ResFinder. In both tools, default settings were employed to enhance the user-friendliness and accessibility of the pipeline, catering to operators with limited expertise in bioinformatics. Furthermore, adhering to these default parameters prevents the introduction of biases that could potentially alter diagnostic outcomes, thereby preserving the integrity of results and maintaining consistency in both intra- and inter-laboratory reproducibility.

The importance of standardized methodologies and pipelines used in WGS in microbiology laboratories is evident^28^. Therefore, the validation strategy suggested by Bogaerts et al.^19^ and performed in our study can be extended to other sequencing technologies and pathogens for use in laboratory routine. Since bioinformatics expertise is one of the main challenges in WGS, it is essential to have bioinformatics professionals permanently employed in clinical laboratories to provide expert interpretation. Additionally, the generation of a centralized and standardized database, as well as computational reproducibility, is of paramount importance^19,22^.

In summary, we validated a bioinformatics pipeline for *K. pneumoniae* identification and its antimicrobial resistance genes. This pipeline can be used in laboratory routine to identify the infecting microorganisms and their antimicrobial resistance mechanisms. Using this pipeline, infected patients could receive more individualized treatment, leading to a reduction in hospitalization duration and mortality rates. Kraken2, as a species identifier, proved to be more accurate, while ABRicate was more effective in identifying antimicrobial resistance genes. SpeciesFinder and ResFinder may need updates. Given the variety of bioinformatics tools and resistance determinant databases available, the validation strategy used in our study can be applied to different bioinformatic pipelines and tools to ensure standardization of intra- and inter-laboratory validation.

## Methodology

### Dataset

Search for carbapenem-resistant *K. pneumoniae* BioProjects was performed in NCBI database (https://www.ncbi.nlm.nih.gov/sra/). Three criteria were used to select the BioProjects: (i) to have carbapenem-resistant *K. pneumoniae* samples isolated from human hosts, (ii) to have been sequenced by Illumina MiSeq technology, and (iii) to present genome assembly as the RefSeq. Seven BioProjects (PRJEB28660, PRJNA292902, PRJNA292904, PRJNA295003, PRJNA307517, PRJNA308116, and PRJNA392824) and 201 SRA met these criteria (Table 4). In addition, a negative control sample was selected. SRAs were downloaded with the fastq-dump tool v. 2.10.9 from SRAToolkit, capable of converting SRA to fastq files.

**Table 4:** BioProjects used for pipeline validation.

No.	BioProject	Sample	Total no. used	Ref
1	PRJEB28660	Carbapenemase-producing *Klebsiella pneumoniae*	56	–
2	PRJNA292902 and PRJNA292904	Carbapenemase-producing *Klebsiella pneumoniae*	69	–
3	PRJNA295003	Carbapenemase-producing *Klebsiella pneumoniae*	31	^32^ ^33^
4	PRJNA307517	Carbapenemase-producing *Klebsiella pneumoniae*	23	^34^ ^35^
5	PRJNA308116	Carbapenemase-producing *Klebsiella pneumoniae*	11	–
6	PRJNA392824	Carbapenemase-producing *Klebsiella pneumoniae*	10	^36^
7	PRJNA279657	*Klebsiella pneumoniae* ATCC 35,657	1	–

### Bacterial genome assembly, annotation, and species identification

**Figure 7:**
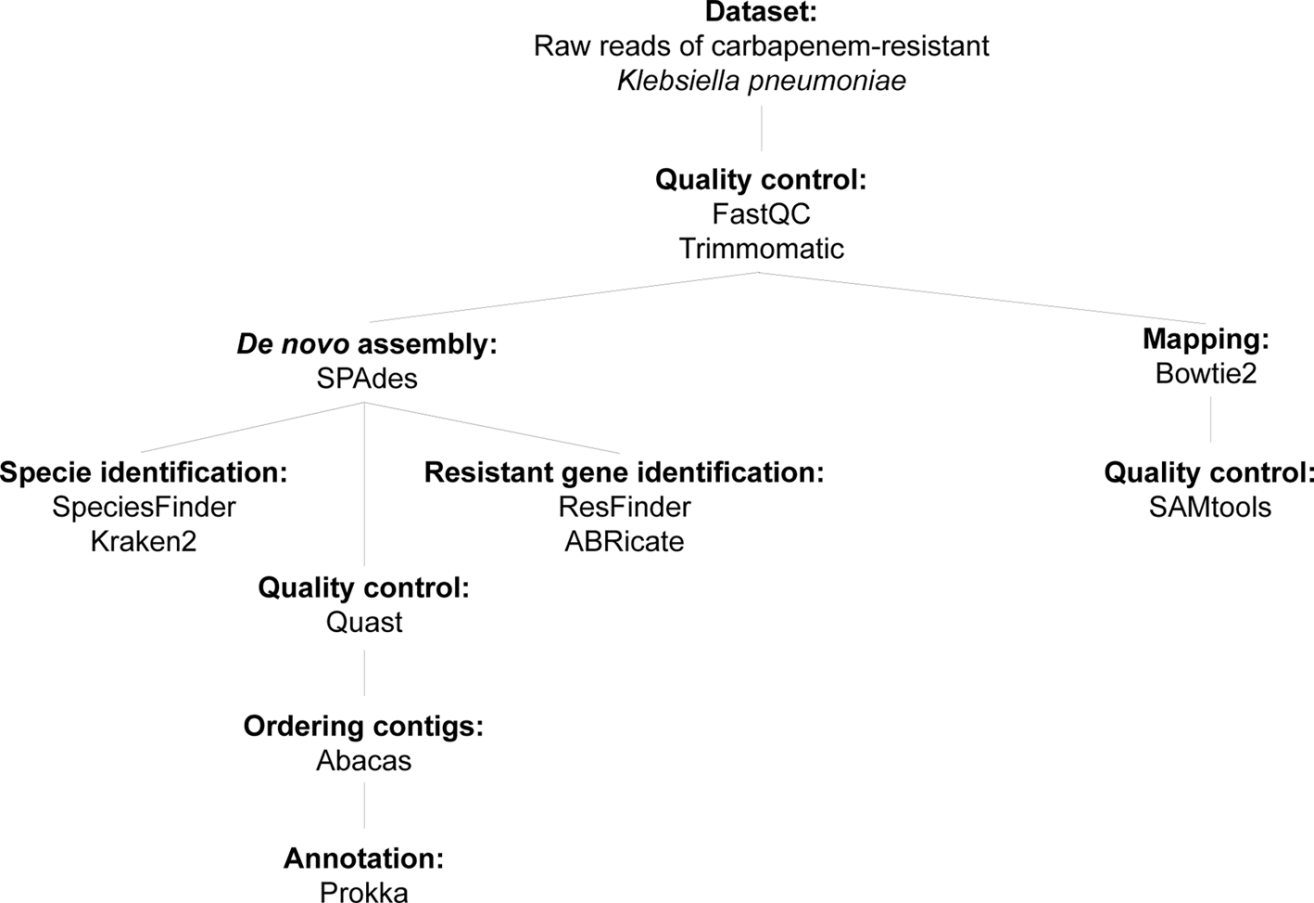
Bioinformatics pipeline used in the work.

Raw sequencing data were evaluated using the FastQC v0.11.9 program with default settings at the Babraham Institute, Cambridge, UK. Subsequently, the samples were subjected to trimming in Trimmomatic v0.39^37^, removing adapter residues, bases with Q-score < 3 at the beginning and end of reads, and Q-score < 15 in a four-base sequence. De novo assembly of the genomes was performed using SPAdes v3.13.1 with the –careful option enabled to reduce the number of mismatches^38^. For mapping, Bowtie2 v2.3.0 was employed, utilizing the *K. pneumoniae* reference genome (NC_016845)^39^. The de novo assembly and mapping statistics were assessed through the online interface of QUAST^40^ and SAMtools^41^, respectively. The generated contigs were then sorted by the ABACAS v1.3.1 program, following the *K. pneumoniae* reference genome (NC_016845)^42^, and subsequently annotated using Prokka v1.14.5^43^ (Fig. 7).

### Species identification

Species identification was performed using the Kraken tool v2.1.1^26^ and SpeciesFinder 2.0^44^ (Fig. 7).

### Identificaction of antimicrobial resistance genes

Identification of antimicrobial resistance genes was performed using ResFinder v4.1^45^ and ABRicate v1.0.1^46^ under default parameters. ABRicate uses the NCBI database by default, while the BLAST tool is configured with an 80% identity and 80% coverage threshold. On the other hand, ResFinder employs the BLAST tool with parameters set at 90% identity and 60% coverage. The bioinformatics pipeline used in the study is shown in Fig. 7.

### Evaluation criteria

Performance analysis, as well as pipeline validation, was performed according to Bogaerts et al.^19^ with adaptations. The following metrics were evaluated: repeatability, reproducibility, accuracy, precision, sensitivity, and specificity (Table 5). For the repeatability calculation, the bioinformatics pipeline was run on the same day using the same dataset. For the reproducibility calculation, the PRJNA292902 and PRJNA292904 BioProjects were selected, which had more than one technical replicate. The pipeline was run on alternate days to evaluate the intra-run reproducibility. Results were considered in agreement when genes were present or absent in both runs. To evaluate accuracy, precision, sensitivity, and specificity, results were categorized as true positive (TP), false positive (FP), true negative (TN), or false negative (FN). TP indicates a gene found by our pipeline and in the reference genome; FP indicates a gene found by our pipeline but absent in the reference genome; TN indicates a gene not found by our pipeline nor in the reference genome, and FN indicates a gene absent from our pipeline but present in the reference genome (Table 5). Some metrics were not evaluated for all bioinformatic assays, as suitable definitions cannot always be found in the context of the specific analysis^19,47^.

**Table 5:** Parameters evaluated in the performance analysis and pipeline validation. TP = true positive; TN = true negative; FP = false positive; FN = false negative.

Metrics	Definition	Formula for calculation
Repeatability	Concordancy based in replicates on the same run in the same assay	Repeatability = (number of intra-assay concordant repetitions)/(total number of repetitions) × 100%
Reproducibility	Concordance based on results generated by different runs for the same sample	Reproducibility = (number of concordant repetitions between assays)/(total number of repetitions) × 100%
Accuracy	Probability that the assay results are correct	Accuracy = (TP + TN)/ (TP + TN + FP + FN) × 100%
Precision	Probability that the detected results are truly positive	Precision = TP/(TP + FP) × 100%
Sensibility	Probability that the result will be correctly detected in the assay when present	Sensibilidade = TP/(TP + FN) × 100%
Specificity	Probability that a result will not be falsely detected in an assay when absent	Specificity = TN/(TN + FP) × 100%

### Supplementary Information

Supplementary Information 1.

Supplementary Information 2.

Supplementary Information 3.

Supplementary Information 4.

## Data Availability

The SRAs are available at NCBI under BioProject ID PRJEB28660, PRJNA292902 and PRJNA292904, PRJNA295003, PRJNA307517, PRJNA308116, PRJNA392824, and PRJNA279657. The SRAs used are listed in detail in Table S4.
