# Supplementary material for: Pipeline validation for the identification of antimicrobial-resistant genes in carbapenem-resistant Klebsiella pneumoniae
Source: Sci Rep. 2023 Sep 14;13:15189. doi: 10.1038/s41598-023-42154-6 (PMC10502106; doi:10.1038/s41598-023-42154-6)
Supplement: Supplementary file 1 — Supplementary Information 1. [file 41598_2023_42154_MOESM1_ESM.pdf]

Table S1 – Identified bacteria by Kraken and SpeciesFinder databases.

| | Kraken | | SpeciesFinder | | |
|---|---|---|---|---|---|
| **SRA** | **Identified bacteria** | **Mapped reads (%)** | **Identified bacteria** | **Bacteria ID** | **Result confidence** |
| ERR2796902 | *K. pneumoniae* | 61 .09 | *K. pneumoniae* | NGTB01000001 | FAIL |
| ERR2796903 | *K. pneumoniae* | 54 .62 | *K. pneumoniae* | NGTB01000001 | FAIL |
| ERR2796904 | *K. pneumoniae* | 62 .92 | *K. pneumoniae* | NGTB01000001 | FAIL |
| ERR2796907 | *K. pneumoniae* | 43 .75 | *P. aeruginosa* | CP008865 | FAIL |
| ERR2796910 | *K. pneumoniae* | 67 .25 | *K. pneumoniae* | NGTB01000001 | FAIL |
| ERR2796911 | *K. pneumoniae* | 48 .39 | *K. pneumoniae* | NGTB01000001 | PASS |
| ERR2796912 | *K. pneumoniae* | 50 .46 | *K. pneumoniae* | NGTB01000001 | FAIL |
| ERR2796917 | *K. pneumoniae* | 46 .96 | *K. pneumoniae* | NGTB01000001 | FAIL |
| ERR2796919 | *K. pneumoniae* | 60 .08 | *K. pneumoniae* | NGTB01000001 | FAIL |
| ERR2796920 | *K. pneumoniae* | 48 .60 | *K. pneumoniae* | NGTB01000001 | FAIL |
| ERR2796923 | *K. pneumoniae* | 58 .65 | *K. pneumoniae* | LGJQ01000010 | FAIL |
| ERR2796926 | *K. pneumoniae* | 56 .83 | *P. aeruginosa* | CP008865 | FAIL |
| ERR2796929 | *K. pneumoniae* | 50 .62 | *K. pneumoniae* | NGTB01000001 | PASS |
| ERR2796930 | *K. pneumoniae* | 36 .46 | *K. pneumoniae* | LGJY01000007 | PASS |
| ERR2796931 | *K. pneumoniae* | 81 .54 | *K. pneumoniae* | NGTB01000001 | PASS |
| ERR2796942 | *K. pneumoniae* | 58 .45 | *K. pneumoniae* | NGTB01000001 | FAIL |
| ERR2796944 | *K. pneumoniae* | 72 .00 | *K. pneumoniae* | NGTB01000001 | FAIL |
| ERR2796946 | *K. pneumoniae* | 64 .63 | *K. pneumoniae* | NGTB01000001 | FAIL |
| ERR2796948 | *K. pneumoniae* | 58 .48 | *K. pneumoniae* | NGTB01000001 | FAIL |
| ERR2796949 | *K. pneumoniae* | 59 .26 | *K. pneumoniae* | NGTB01000001 | FAIL |
| ERR2796950 | *K. pneumoniae* | 59 .02 | *K. pneumoniae* | NGTB01000001 | PASS |
| ERR2796952 | *K. pneumoniae* | 58 .77 | *K. pneumoniae* | NGTB01000001 | FAIL |
| ERR2796953 | *K. pneumoniae* | 55 .47 | *K. pneumoniae* | NGTB01000001 | FAIL |
| ERR2796955 | *K. pneumoniae* | 63 .61 | *K. pneumoniae* | NGTB01000001 | FAIL |
| ERR2796956 | *K. pneumoniae* | 53 .16 | *K. pneumoniae* | NGTB01000001 | FAIL |
| ERR2796957 | *K. pneumoniae* | 58 .55 | *K. pneumoniae* | NGTB01000001 | FAIL |

| | | | | | |
|---|---|---|---|---|---|
| ERR2796958 | *K. pneumoniae* | 41 .18 | *K. pneumoniae* | LGJQ01000010 | FAIL |
| ERR2796961 | *K. pneumoniae* | 51 .27 | *K. pneumoniae* | NGTB01000001 | FAIL |
| ERR2796963 | *K. pneumoniae* | 54 .46 | *K. pneumoniae* | NGTB01000001 | FAIL |
| ERR2796964 | *K. pneumoniae* | 39 .37 | *K. pneumoniae* | LGJY01000007 | PASS |
| ERR2796969 | *K. pneumoniae* | 44 .09 | *K. pneumoniae* | NGTB01000001 | FAIL |
| ERR2796976 | *K. pneumoniae* | 46 .40 | *K. pneumoniae* | NGTB01000001 | PASS |
| ERR2796977 | *K. pneumoniae* | 46 .92 | *K. pneumoniae* | NGTB01000001 | PASS |
| ERR2796978 | *K. pneumoniae* | 62 .56 | *K. pneumoniae* | NGTB01000001 | PASS |
| ERR2796981 | *K. pneumoniae* | 41 .90 | *K. pneumoniae* | NGTB01000001 | PASS |
| ERR2796983 | *K. pneumoniae* | 40 .57 | *K. pneumoniae* | NGTB01000001 | FAIL |
| ERR2796984 | *K. pneumoniae* | 66 .67 | *P. aeruginosa* | CP008865 | FAIL |
| ERR2796988 | *K. pneumoniae* | 45 .06 | *K. pneumoniae* | NGTB01000001 | FAIL |
| ERR2796989 | *K. pneumoniae* | 51 .49 | *K. pneumoniae* | NGTB01000001 | FAIL |
| ERR2796990 | *K. pneumoniae* | 60 .00 | *K. pneumoniae* | NGTB01000001 | FAIL |
| ERR2796992 | *K. pneumoniae* | 55 .61 | *K. pneumoniae* | NGTB01000001 | FAIL |
| ERR2796993 | *K. pneumoniae* | 56 .70 | *K. pneumoniae* | NGTB01000001 | FAIL |
| ERR2796994 | *K. pneumoniae* | 38 .50 | *P. aeruginosa* | CP008865 | FAIL |
| ERR2796995 | *K. pneumoniae* | 71 .74 | *K. pneumoniae* | NGTB01000001 | FAIL |
| ERR2796996 | *K. pneumoniae* | 71 .71 | *K. pneumoniae* | NGTB01000001 | FAIL |
| ERR2796998 | *K. pneumoniae* | 62 .35 | *K. pneumoniae* | NGTB01000001 | FAIL |
| ERR2797002 | *K. pneumoniae* | 69 .59 | *K. pneumoniae* | NGTB01000001 | FAIL |
| ERR2797003 | *K. pneumoniae* | 62 .50 | *K. pneumoniae* | NGTB01000001 | FAIL |
| ERR2797004 | *K. pneumoniae* | 66 .02 | *K. pneumoniae* | NGTB01000001 | FAIL |
| ERR2797005 | *K. pneumoniae* | 56 .31 | *K. pneumoniae* | NGTB01000001 | FAIL |
| ERR2797006 | *K. pneumoniae* | 55 .16 | *K. pneumoniae* | NGTB01000001 | FAIL |
| ERR2797007 | *K. pneumoniae* | 71 .15 | *K. pneumoniae* | NGTB01000001 | FAIL |
| ERR2797008 | *K. pneumoniae* | 55 .10 | *K. pneumoniae* | NGTB01000001 | FAIL |
| ERR2797009 | *K. pneumoniae* | 52 .36 | *K. pneumoniae* | NGTB01000001 | FAIL |
| ERR2797010 | *K. pneumoniae* | 43 .49 | *P. aeruginosa* | CP008865 | FAIL |

| | | | | | |
|---|---|---|---|---|---|
| ERR2797011 | *K. pneumoniae* | 64 .69 | *K. pneumoniae* | NGTB01000001 | FAIL |
| SRR3242012 | *K. pneumoniae* | 65 .74 | *K. pneumoniae* | NGTB01000001 | PASS |
| SRR4025850 | *K. pneumoniae* | 44 .03 | *K. pneumoniae* | NGTB01000001 | FAIL |
| SRR4025851 | *K. pneumoniae* | 43 .79 | *K. pneumoniae* | NGTB01000001 | FAIL |
| SRR4025861 | *K. pneumoniae* | 46 .88 | *K. pneumoniae* | NGTB01000001 | FAIL |
| SRR4025863 | *K. pneumoniae* | 50 .00 | *K. pneumoniae* | NGTB01000001 | FAIL |
| SRR4025977 | *K. pneumoniae* | 40 .83 | *K. pneumoniae* | NGTB01000001 | FAIL |
| SRR4025979 | *K. pneumoniae* | 39 .41 | *K. pneumoniae* | NGTB01000001 | FAIL |
| SRR4025980 | *K. pneumoniae* | 44 .55 | *K. pneumoniae* | NGTB01000001 | PASS |
| SRR4025983 | *K. pneumoniae* | 45 .28 | *K. pneumoniae* | NGTB01000001 | FAIL |
| SRR4025984 | *K. pneumoniae* | 40 .80 | *K. pneumoniae* | NGTB01000001 | FAIL |
| SRR4025985 | *K. pneumoniae* | 41 .09 | *K. pneumoniae* | LGJY01000007 | PASS |
| SRR4025987 | *K. pneumoniae* | 41 .98 | *K. pneumoniae* | NGTB01000001 | FAIL |
| SRR4025990 | *K. pneumoniae* | 34 .09 | *K. pneumoniae* | NGTB01000001 | FAIL |
| SRR4025991 | *K. pneumoniae* | 53 .30 | *K. pneumoniae* | NGTB01000001 | FAIL |
| SRR4025992 | *K. pneumoniae* | 49 .75 | *P. aeruginosa* | CP008865 | FAIL |
| SRR4025993 | *K. pneumoniae* | 37 .11 | *K. pneumoniae* | NGTB01000001 | FAIL |
| SRR4025994 | *K. pneumoniae* | 43 .66 | *K. pneumoniae* | NGTB01000001 | FAIL |
| SRR4025996 | *K. pneumoniae* | 45 .23 | *K. pneumoniae* | LGJQ01000010 | FAIL |
| SRR4026000 | *K. pneumoniae* | 41 .53 | *K. pneumoniae* | NGTB01000001 | FAIL |
| SRR4026003 | *K. pneumoniae* | 35 .76 | *K. pneumoniae* | NGTB01000001 | PASS |
| SRR5122322 | *K. pneumoniae* | 38 .42 | *K. pneumoniae* | NGTB01000001 | FAIL |
| SRR5146462 | *K. pneumoniae* | 50 .82 | *K. pneumoniae* | NGTB01000001 | FAIL |
| SRR5146463 | *K. pneumoniae* | 52 .60 | *K. pneumoniae* | NGTB01000001 | FAIL |
| SRR5167852 | *K. pneumoniae* | 51 .23 | *K. pneumoniae* | NGTB01000001 | FAIL |
| SRR5167853 | *K. pneumoniae* | 52 .48 | *K. pneumoniae* | NGTB01000001 | FAIL |
| SRR5168221 | *K. pneumoniae* | 58 .82 | *K. pneumoniae* | NGTB01000001 | PASS |
| SRR5168222 | *K. pneumoniae* | 60 .26 | *K. pneumoniae* | NGTB01000001 | PASS |
| SRR5168231 | *K. pneumoniae* | 58 .29 | *K. pneumoniae* | NGTB01000001 | FAIL |

| | | | | | |
|---|---|---|---|---|---|
| SRR5168232 | *K. pneumoniae* | 56 .41 | *K. pneumoniae* | NGTB01000001 | FAIL |
| SRR5168235 | *K. pneumoniae* | 63 .24 | *K. pneumoniae* | NGTB01000001 | FAIL |
| SRR5168236 | *K. pneumoniae* | 64 .14 | *K. pneumoniae* | NGTB01000001 | FAIL |
| SRR5168243 | *K. pneumoniae* | 45 .86 | *K. pneumoniae* | NGTB01000001 | FAIL |
| SRR5168244 | *K. pneumoniae* | 52 .81 | *K. pneumoniae* | NGTB01000001 | FAIL |
| SRR5168370 | *K. pneumoniae* | 49 .69 | *K. pneumoniae* | NGTB01000001 | FAIL |
| SRR5168371 | *K. pneumoniae* | 50 .94 | *K. pneumoniae* | NGTB01000001 | FAIL |
| SRR5168372 | *K. pneumoniae* | 52 .15 | *K. pneumoniae* | NGTB01000001 | FAIL |
| SRR5168375 | *K. pneumoniae* | 53 .97 | *K. pneumoniae* | NGTB01000001 | FAIL |
| SRR5168376 | *K. pneumoniae* | 46 .85 | *K. pneumoniae* | NGTB01000001 | FAIL |
| SRR5168377 | *K. pneumoniae* | 52 .22 | *K. pneumoniae* | NGTB01000001 | PASS |
| SRR5168378 | *K. pneumoniae* | 49 .55 | *K. pneumoniae* | NGTB01000001 | PASS |
| SRR5168384 | *K. pneumoniae* | 56 .13 | *K. pneumoniae* | NGTB01000001 | FAIL |
| SRR5168385 | *K. pneumoniae* | 56 .29 | *K. pneumoniae* | NGTB01000001 | FAIL |
| SRR5168386 | *K. pneumoniae* | 43 .53 | *K. pneumoniae* | NGTB01000001 | FAIL |
| SRR5168387 | *K. pneumoniae* | 53 .40 | *K. pneumoniae* | NGTB01000001 | FAIL |
| SRR5168388 | *K. pneumoniae* | 48 .40 | *K. pneumoniae* | NGTB01000001 | FAIL |
| SRR5168389 | *K. pneumoniae* | 44 .36 | *K. pneumoniae* | NGTB01000001 | FAIL |
| SRR5168390 | *K. pneumoniae* | 41 .48 | *K. pneumoniae* | LGJQ01000010 | FAIL |
| SRR5168393 | *K. pneumoniae* | 52 .31 | *K. pneumoniae* | NGTB01000001 | FAIL |
| SRR5168394 | *K. pneumoniae* | 50 .75 | *K. pneumoniae* | NGTB01000001 | FAIL |
| SRR5168481 | *K. pneumoniae* | 58 .01 | *K. pneumoniae* | NGTB01000001 | FAIL |
| SRR5168482 | *K. pneumoniae* | 60 .12 | *K. pneumoniae* | NGTB01000001 | FAIL |
| SRR5168483 | *K. pneumoniae* | 60 .62 | *K. pneumoniae* | NGTB01000001 | FAIL |
| SRR5168485 | *K. pneumoniae* | 57 .51 | *K. pneumoniae* | NGTB01000001 | PASS |
| SRR5168486 | *K. pneumoniae* | 61 .11 | *K. pneumoniae* | NGTB01000001 | FAIL |
| SRR5168488 | *K. pneumoniae* | 49 .55 | *P. aeruginosa* | CP008865 | FAIL |
| SRR5168489 | *K. pneumoniae* | 51 .50 | *P. aeruginosa* | CP008865 | FAIL |
| SRR5168490 | *K. pneumoniae* | 43 .96 | *K. pneumoniae* | NGTB01000001 | FAIL |

| | | | | | |
|---|---|---|---|---|---|
| SRR5168491 | *K. pneumoniae* | 48 .48 | *K. pneumoniae* | NGTB01000001 | FAIL |
| SRR5168492 | *K. pneumoniae* | 52 .00 | *K. pneumoniae* | NGTB01000001 | FAIL |
| SRR5168493 | *K. pneumoniae* | 50 .43 | *K. pneumoniae* | NGTB01000001 | FAIL |
| SRR5168496 | *K. pneumoniae* | 46 .85 | *K. pneumoniae* | NGTB01000001 | FAIL |
| SRR5168497 | *K. pneumoniae* | 47 .22 | *K. pneumoniae* | NGTB01000001 | FAIL |
| SRR5168498 | *K. pneumoniae* | 51 .96 | *K. pneumoniae* | NGTB01000001 | FAIL |
| SRR5168509 | *K. pneumoniae* | 53 .28 | *K. pneumoniae* | NGTB01000001 | FAIL |
| SRR5168510 | *K. pneumoniae* | 48 .12 | *K. pneumoniae* | NGTB01000001 | FAIL |
| SRR5168511 | *K. pneumoniae* | 46 .15 | *K. pneumoniae* | NGTB01000001 | FAIL |
| SRR5168517 | *K. pneumoniae* | 40 .07 | *K. pneumoniae* | NGTB01000001 | PASS |
| SRR5168518 | *K. pneumoniae* | 42 .66 | *K. pneumoniae* | NGTB01000001 | PASS |
| SRR5168519 | *K. pneumoniae* | 41 .16 | *K. pneumoniae* | NGTB01000001 | PASS |
| SRR2724077 | *K. pneumoniae* | 41 .56 | *K. pneumoniae* | LEZY01000019 | FAIL |
| SRR2724078 | *K. pneumoniae* | 52 .84 | *K. pneumoniae* | NGTB01000001 | FAIL |
| SRR2724081 | *K. pneumoniae* | 39 .57 | *P. aeruginosa* | CP008865 | FAIL |
| SRR2724082 | *K. pneumoniae* | 51 .33 | *K. pneumoniae* | NGTB01000001 | FAIL |
| SRR2724083 | *K. pneumoniae* | 41 .24 | *K. pneumoniae* | NGTB01000001 | FAIL |
| SRR2724085 | *K. pneumoniae* | 47 .50 | *K. pneumoniae* | NGTB01000001 | FAIL |
| SRR2724086 | *K. pneumoniae* | 47 .52 | *K. pneumoniae* | NGTB01000001 | FAIL |
| SRR2724087 | *K. pneumoniae* | 54 .70 | *K. pneumoniae* | NGTB01000001 | FAIL |
| SRR2724088 | *K. pneumoniae* | 56 .27 | *K. pneumoniae* | NGTB01000001 | FAIL |
| SRR2724089 | *K. pneumoniae* | 48 .28 | *K. pneumoniae* | NGTB01000001 | FAIL |
| SRR2724090 | *K. pneumoniae* | 69 .44 | *K. pneumoniae* | NGTB01000001 | FAIL |
| SRR2724091 | *K. pneumoniae* | 25 .17 | *K. pneumoniae* | NGTB01000001 | FAIL |
| SRR2724092 | *K. pneumoniae* | 70 .37 | *P. aeruginosa* | CP008865 | FAIL |
| SRR2724093 | *K. pneumoniae* | 38 .07 | *K. pneumoniae* | NGTB01000001 | FAIL |
| SRR2724096 | *K. pneumoniae* | 59 .00 | *K. pneumoniae* | NGTB01000001 | FAIL |
| SRR2724097 | *K. pneumoniae* | 45 .23 | *K. pneumoniae* | NGTB01000001 | FAIL |
| SRR2724098 | *K. pneumoniae* | 46 .06 | *K. pneumoniae* | NGTB01000001 | FAIL |

| | | | | | |
|---|---|---|---|---|---|
| SRR2724100 | *K. pneumoniae* | 55 .81 | *K. pneumoniae* | NGTB01000001 | FAIL |
| SRR2724109 | *K. pneumoniae* | 41 .18 | *K. pneumoniae* | NGTB01000001 | PASS |
| SRR2724111 | *K. pneumoniae* | 59 .35 | *K. pneumoniae* | NGTB01000001 | FAIL |
| SRR2724113 | *K. pneumoniae* | 24 .55 | *P. aeruginosa* | CP008865 | FAIL |
| SRR2724122 | *K. pneumoniae* | 56 .95 | *K. pneumoniae* | NGTB01000001 | FAIL |
| SRR2724123 | *K. pneumoniae* | 63 .46 | *K. pneumoniae* | NGTB01000001 | FAIL |
| SRR2724129 | *K. pneumoniae* | 40 .22 | *K. pneumoniae* | NGTB01000001 | FAIL |
| SRR2724130 | *K. pneumoniae* | 56 .87 | *K. pneumoniae* | NGTB01000001 | FAIL |
| SRR2724134 | *K. pneumoniae* | 56 .13 | *K. pneumoniae* | NGTB01000001 | FAIL |
| SRR2724135 | *K. pneumoniae* | 39 .76 | *K. pneumoniae* | NGTB01000001 | PASS |
| SRR2724137 | *K. pneumoniae* | 65 .49 | *K. pneumoniae* | NGTB01000001 | PASS |
| SRR2724138 | *K. pneumoniae* | 48 .39 | *K. pneumoniae* | NGTB01000001 | FAIL |
| SRR2724139 | *K. pneumoniae* | 56 .77 | *K. pneumoniae* | NGTB01000001 | FAIL |
| SRR2724140 | *K. pneumoniae* | 56 .50 | *K. pneumoniae* | NGTB01000001 | FAIL |
| SRR3465532 | *K. pneumoniae* | 33 .90 | *C. freundii* | NGRE01000001 | FAIL |
| SRR4115668 | *K. pneumoniae* | 45 .41 | *K. pneumoniae* | NGTB01000001 | PASS |
| SRR5132378 | *K. pneumoniae* | 39 .90 | *K. pneumoniae* | NGTB01000001 | FAIL |
| SRR5132379 | *K. pneumoniae* | 37 .08 | *K. pneumoniae* | NGTB01000001 | PASS |
| SRR5132381 | *K. pneumoniae* | 39 .90 | *K. pneumoniae* | CP026495 | PASS |
| SRR5132447 | *K. pneumoniae* | 43 .75 | *K. pneumoniae* | NGTB01000001 | PASS |
| SRR5132448 | *K. pneumoniae* | 50 .51 | *K. pneumoniae* | NGTB01000001 | FAIL |
| SRR5132449 | *K. pneumoniae* | 60 .87 | *K. pneumoniae* | NGTB01000001 | FAIL |
| SRR5132450 | *K. pneumoniae* | 47 .98 | *K. pneumoniae* | NGTB01000001 | PASS |
| SRR5132451 | *K. pneumoniae* | 42 .35 | *K. pneumoniae* | NGTB01000001 | FAIL |
| SRR5132452 | *K. pneumoniae* | 52 .08 | *K. pneumoniae* | NGTB01000001 | FAIL |
| SRR5132453 | *K. pneumoniae* | 54 .38 | *K. pneumoniae* | NGTB01000001 | FAIL |
| SRR5809251 | *K. pneumoniae* | 27 .38 | *K. pneumoniae* | NGTB01000001 | PASS |
| SRR5809254 | *K. pneumoniae* | 27 .91 | *K. pneumoniae* | NGTB01000001 | PASS |
| SRR5809260 | *K. pneumoniae* | 47 .06 | *K. pneumoniae* | NGTB01000001 | FAIL |

| | | | | | |
|---|---|---|---|---|---|
| SRR5809261 | *K. pneumoniae* | 23 .47 | *K. pneumoniae* | NGTB01000001 | PASS |
| SRR5809262 | *K. pneumoniae* | 47 .57 | *K. pneumoniae* | NGTB01000001 | FAIL |
| SRR5809264 | *K. pneumoniae* | 51 .55 | *K. pneumoniae* | NGTB01000001 | FAIL |
| SRR5809265 | *K. pneumoniae* | 46 .53 | *K. pneumoniae* | NGTB01000001 | FAIL |
| SRR5809266 | *K. pneumoniae* | 27 .27 | *K. pneumoniae* | NGTB01000001 | PASS |
| SRR5809267 | *K. pneumoniae* | 48 .91 | *K. pneumoniae* | NGTB01000001 | FAIL |
| SRR5809268 | *K. pneumoniae* | 28 .24 | *K. pneumoniae* | NGTB01000001 | PASS |
| SRR8607448 | *K. pneumoniae* | 41 .95 | *P. aeruginosa* | CP008865 | FAIL |
| SRR8607449 | *K. pneumoniae* | 53 .09 | *K. pneumoniae* | NGTB01000001 | FAIL |
| SRR8607450 | *K. pneumoniae* | 40 .86 | *K. pneumoniae* | NGTB01000001 | FAIL |
| SRR8607451 | *K. pneumoniae* | 48 .72 | *K. pneumoniae* | NGTB01000001 | FAIL |
| SRR8607452 | *K. pneumoniae* | 35 .71 | *P. aeruginosa* | CP008865 | FAIL |
| SRR8607453 | *K. pneumoniae* | 34 .30 | *P. aeruginosa* | CP008865 | FAIL |
| SRR8607454 | *K. pneumoniae* | 55 .17 | *K. pneumoniae* | NGTB01000001 | FAIL |
| SRR8607455 | *K. pneumoniae* | 47 .27 | *K. pneumoniae* | NGTB01000001 | FAIL |
| SRR8607456 | *K. pneumoniae* | 53 .08 | *K. pneumoniae* | NGTB01000001 | FAIL |
| SRR8607457 | *K. pneumoniae* | 54 .19 | *K. pneumoniae* | NGTB01000001 | FAIL |
| SRR8607458 | *K. pneumoniae* | 53 .80 | *K. pneumoniae* | NGTB01000001 | FAIL |
| SRR8607459 | *K. pneumoniae* | 59 .73 | *K. pneumoniae* | NGTB01000001 | FAIL |
| SRR8607460 | *K. pneumoniae* | 59 .21 | *K. pneumoniae* | NGTB01000001 | FAIL |
| SRR8607461 | *K. pneumoniae* | 60 .48 | *K. pneumoniae* | NGTB01000001 | FAIL |
| SRR8607462 | *K. pneumoniae* | 57 .14 | *K. pneumoniae* | NGTB01000001 | FAIL |
| SRR8607463 | *K. pneumoniae* | 53 .29 | *K. pneumoniae* | NGTB01000001 | FAIL |
| SRR8607464 | *K. pneumoniae* | 59 .88 | *K. pneumoniae* | NGTB01000001 | FAIL |
| SRR8607465 | *K. pneumoniae* | 60 .34 | *K. pneumoniae* | NGTB01000001 | FAIL |
| SRR8607466 | *K. pneumoniae* | 57 .48 | *K. pneumoniae* | NGTB01000001 | FAIL |
| SRR8607467 | *K. pneumoniae* | 48 .82 | *K. pneumoniae* | NGTB01000001 | FAIL |
| SRR8607468 | *K. pneumoniae* | 54 .41 | *K. pneumoniae* | NGTB01000001 | FAIL |
| SRR8607470 | *K. pneumoniae* | 40 .08 | *K. pneumoniae* | NGTB01000001 | FAIL |

| | | | | | |
|---|---|---|---|---|---|
| SRR8607471 | *K. pneumoniae* | 54 .04 | *K. pneumoniae* | NGTB01000001 | FAIL |
