# Supplementary material for: Pipeline validation for the identification of antimicrobial-resistant genes in carbapenem-resistant Klebsiella pneumoniae
Source: Sci Rep. 2023 Sep 14;13:15189. doi: 10.1038/s41598-023-42154-6 (PMC10502106; doi:10.1038/s41598-023-42154-6)
Supplement: Supplementary file 2 — Supplementary Information 2. [file 41598_2023_42154_MOESM2_ESM.pdf]

Table S2 - Antimicrobial resistance genes evaluated by ResFinder and ABRicate databases. [1]ResFinder access; [2]ABRicate access; [3]different access, but same sequence; [4]different access and sequence.

**Gene**	**ResFinder[1]**	**ABRicate[2]**	**Nomenclature**	**Resistance phenotype**
bla$_{KPC-2}$	AY034847	NG_049253.1	Carbapenem-hydrolyzing class A beta-lactamase KPC-2	Carbapenem
bla$_{KPC-3}$	HM769262[3]	NG_049257.1[3]	Carbapenem-hydrolyzing class A beta-lactamase KPC-3	Carbapenem
bla$_{NDM-1}$	FN396876	NG_049326.1	Subclass B1 metallo-beta-lactamase NDM-1	Carbapenem
bla$_{NDM-7}$	JX262694	NG_049339.1	Subclass B1 metallo-beta-lactamase NDM-7	Carbapenem
bla$_{OXA-48}$	AY236073	NG_049762.1	OXA-48 family carbapenem-hydrolyzing class D beta-lactamase OXA-48	Carbapenem
bla$_{OXA-162}$	GU197550[3]	NG_049461.1[3]	OXA-48 family carbapenem-hydrolyzing class D beta-lactamase OXA-162	Carbapenem
bla$_{OXA-181}$	CM004561[3]	NG_049482.1[3]	OXA-48 family carbapenem-hydrolyzing class D beta-lactamase OXA-181	Carbapenem
bla$_{OXA-232}$	JX423831	NG_049528.1	OXA-48 family carbapenem-hydrolyzing class D beta-lactamase OXA-232	Carbapenem
bla$_{OXA-245}$	JX438001	NG_049540.1	OXA-48 family carbapenem-hydrolyzing class D beta-lactamase OXA-245	Carbapenem
bla$_{VIM-1}$	Y18050	NG_050336.1	Subclass B1 metallo-beta-lactamase VIM-1	Carbapenem
bla$_{VIM-19}$	FJ499397[3]	NG_050346.1[3]	Subclass B1 metallo-beta-lactamase VIM-19	Carbapenem
bla$_{VIM-27}$	HQ858608	NG_050353.1	Subclass B1 metallo-beta-lactamase VIM-27	Carbapenem
aac(3)-I	AJ877225	ND	AAC(3)-I family aminoglycoside 3-N-acetyltransferase	Aminoglycoside
aac(3)-Ia	ND	NG_047234.1	Aminoglycoside N-acetyltransferase AAC(3)-Ia	Aminoglycoside
aac(3)-II	ND	NG_047231.1	Aminoglycoside N-acetyltransferase AAC(3)-iig	Aminoglycoside
aac(3)-IIe/aac(3)-IIa	X51534	NG_047244.1	Aminoglycoside N-acetyltransferase AAC(3)-iie	Aminoglycoside
aac(3)-IVa/aac(3)-IV	DQ241380	NG_047253.1	Aminoglycoside N-acetyltransferase AAC(3)-iva	Aminoglycoside
armA	AY220558	NG_047476.1	16S rrna (guanine(1405)-N(7))-methyltransferase arma	Aminoglycoside

aac(6')-Ib-AGKT	ND	NG_052219.1	AAC(6')-Ib family aminoglycoside 6'-N-acetyltransferase	Aminoglycoside
aac(6')-Ib-AKT	ND	NG_052262.1	AAC(6')-Ib family aminoglycoside 6'-N-acetyltransferase	Aminoglycoside
aac(6')-Ib-AKT	ND	NG_052358.1	AAC(6')-Ib family aminoglycoside 6'-N-acetyltransferase	Aminoglycoside
aac(6')-Ib-AKT	ND	NG_052418.1	AAC(6')-Ib family aminoglycoside 6'-N-acetyltransferase	Aminoglycoside
aac(6')-Ib-AKT/aac(6')-Ib	M21682[3]	NG_056043.1[3]	AAC(6')-Ib family aminoglycoside 6'-N-acetyltransferase	Aminoglycoside
aac(6')-Ib-D181Y	ND	NG_067946.1	AAC(6')-Ib family aminoglycoside 6'-N-acetyltransferase	Aminoglycoside
aac(6')-Ib-G	ND	NG_047291.1	AAC(6')-Ib family aminoglycoside 6'-N-acetyltransferase	Aminoglycoside
aac(6')-Ib-G	ND	NG_052361.1	AAC(6')-Ib family aminoglycoside 6'-N-acetyltransferase	Aminoglycoside
aac(6')-Ib'	ND	NG_051695.1	Aminoglycoside N-acetyltransferase AAC(6')-Ib'	Aminoglycoside
aac(6')-Ib11	ND	NG_048578.1	Aminoglycoside N-acetyltransferase AAC(6')-Ib11	Aminoglycoside
aac(6')-33	GQ337064	NG_047267.1	Aminoglycoside 6'-N-acetyltransferase AAC(6')-33	Aminoglycoside
aac(3)-IIa	CP023555	ND	Aminoglycoside N-acetyltransferase AAC(3)-iia	Aminoglycoside
aac(3)-IId	EU022314[4]	NG_047251.1[4]	Aminoglycoside N-acetyltransferase AAC(3)-iid	Aminoglycoside
aac(6')-Ib-Hangzhou	FJ503047	ND	Aminoglycoside acetyltransferase	Aminoglycoside
aac(6')-Ib3	X60321	ND	Aminoglycoside N-acetyltransferase AAC(6')-Ib3	Aminoglycoside
aac(6')-IIa	M29695[4]	NG_052142.1[4]	Aminoglycoside N-acetyltransferase AAC(6')-iia	Aminoglycoside
aac(6')-IIc	NC_012555[3]	NG_047273.1[3]	Aminoglycoside N-acetyltransferase AAC(6')-iic	Aminoglycoside
aac(6')-Il	U13880	NG_047303.1	Aminoglycoside N-acetyltransferase AAC(6')-Il	Aminoglycoside
aac(6')-Im	AF337947	NG_047306.1	Aminoglycoside N-acetyltransferase AAC(6')-Im	Aminoglycoside
ant(2'')-Ia	DQ266447	ND	Aminoglycoside nucleotidyltransferase ANT(2'')-Ia	Aminoglycoside
	HQ880250	ND		

	X04555[3]	NG_047387.1[3]		
aph(2'')-IIa/aph(2'')-Ib	AF337947	NG_047401.1	Aminoglycoside O-phosphotransferase APH(2")-iia	Aminoglycoside
ant(3'')-Ia	X02340	ND	ANT(3")-Ia family aminoglycoside nucleotidyltransferase aada	Aminoglycoside
aph(3'')-Ib	AF024602[4]	NG_056002.2[4]	Aminoglycoside O-phosphotransferase APH(3")-Ib	Aminoglycoside
	AF313472[4]			
	AF321550[4]			
	AF321551[4]			
aph(3')-Ia	V00359	NG_047430.1	Aminoglycoside O-phosphotransferase APH(3')-Ia	Aminoglycoside
	X62115	NG_047431.1		
	EU855787	NG_047440.1		
aph(3')-VI	KC170992	NG_051730.1	APH(3')-VI family aminoglycoside O-phosphotransferase	Aminoglycoside
aph(3')-VIa	X07753	NG_047448.1	Aminoglycoside O-phosphotransferase APH(3')-via	Aminoglycoside
aph(4)-Ia	V01499	NG_047456.1	Aminoglycoside O-phosphotransferase APH(4)-Ia	Aminoglycoside
aph(6)-Id	CP000971[3]	NG_047466.1[3]	Aminoglycoside O-phosphotransferase APH(6)-Id	Aminoglycoside
	M28829	NG_047464.1		
aadA1	FJ591054[4]	NG_047325.1[4]	ANT(3")-Ia family aminoglycoside nucleotidyltransferase aada1	Aminoglycoside
	JQ414041[4]	NG_052028.1[4]		
	JQ480156[3]	NG_047327.1[3]		
	JX185132[4]	NG_052230.1[4]		
	ND	NG_052266.1		
	ND	NG_052279.1		
	ND	NG_052440.1		

aadA16	EU675686[4]	NG_047339.1[4]	ANT(3")-Ia family aminoglycoside nucleotidyltransferase aada16	Aminoglycoside
aadA2	JQ364967	NG_047343.1	ANT(3")-Ia family aminoglycoside nucleotidyltransferase aada2	Aminoglycoside
	NC_010870[4]	NG_051846.1[4]		
aadA5	AF137361	NG_047357.1	ANT(3")-Ia family aminoglycoside nucleotidyltransferase aada5	Aminoglycoside
aadA22	AM261837	ND	ANT(3")-Ia family aminoglycoside nucleotidyltransferase aada22	Aminoglycoside
aadA23	AJ809407	ND	Aminoglycoside adenyltransferase	Aminoglycoside
aadA24	DQ677333	ND	Aminoglycoside adenyltransferase	Aminoglycoside
aadA2b	D43625	ND	Streptomycin-spectinomycin adenylyltransferase	Aminoglycoside
rmtB1/rmtB	AB103506	NG_048058.1	16S rrna (guanine(1405)-N(7))-methyltransferase rmtb1	Aminoglycoside
rmtC	AB194779	NG_048060.1	Rmtc family 16S rrna (guanine(1405)-N(7))-methyltransferase	Aminoglycoside
rmtD1/rmtD	DQ914960	NG_048061.1	16S rrna (guanine(1405)-N(7))-methyltransferase rmtd1	Aminoglycoside
rmtF1/rmtF	JQ808129	NG_048062.1	16S rrna (guanine(1405)-N(7))-methyltransferase rmtf1	Aminoglycoside
rmtG	JX486113	NG_048064.1	Rmtg family 16S rrna (guanine(1405)-N(7))-methyltransferase	Aminoglycoside
aac(6')-Ib-cr5/aac(6')-Ib-cr	EF636461[3]	NG_051711.1[3]	Fluoroquinolone-acetylating aminoglycoside 6'-N-acetyltransferase AAC(6')-Ib-cr5	Aminoglycoside/Quinolone
aac(6')-Ib-cr	DQ303918	ND	Fluoroquinolone-acetylating aminoglycoside 6'-N-acetyltransferase AAC(6')-Ib-cr	Aminoglycoside/Quinolone
bla$_{CMY-16}$	AJ781421	NG_048810.1	Class C beta-lactamase CMY-16	β-lactams
bla$_{CMY-4}$	LNHZ01000079[3]	NG_048834.1[3]	Class C beta-lactamase CMY-4	β-lactams
bla$_{CMY-6}$	AJ011293	NG_048855.1	Class C beta-lactamase CMY-6	β-lactams
bla$_{CTX-M-14}$	AF252622	NG_048929.1	Extended-spectrum class A beta-lactamase CTX-M-14	β-lactams
bla$_{CTX-M-15}$	AY044436	NG_048935.1	Extended-spectrum class A beta-lactamase CTX-M-15	β-lactams
bla$_{CTX-M-2}$	AB176535[3]	NG_048968.1[3]	Extended-spectrum class A beta-lactamase CTX-M-2	β-lactams
bla$_{CTX-M-8}$	AF189721	NG_049032.1	Extended-spectrum class A beta-lactamase CTX-M-8	β-lactams

bla$_{\text{CTX-M-97}}$	HM776707	ND	Extended-spectrum class A beta-lactamase CTX-M-97	β-lactams
bla$_{\text{DHA-1}}$	Y16410	NG_049055.1	Class C beta-lactamase DHA-1	β-lactams
bla$_{\text{DHA-24}}$	KU759569	ND	Class C beta-lactamase DHA-24	β-lactams
bla$_{\text{DHA-7}}$	HQ456945	NG_049075.1	Class C beta-lactamase DHA-7	β-lactams
bla$_{\text{LAP-2}}$	EU159120	NG_049264.1	Class A beta-lactamase LAP-2	β-lactams
bla$_{\text{OXA-1}}$	HQ170510[3]	NG_049392.1[3]	Oxacillin-hydrolyzing class D beta-lactamase OXA-1	β-lactams
bla$_{\text{OXA-9}}$	KQ089875[3]	NG_049830.1[3]	Oxacillin-hydrolyzing class D beta-lactamase OXA-9	β-lactams
bla$_{\text{OXA-10}}$	J03427[3]	NG_049393.1[3]	Oxacillin-hydrolyzing class D beta-lactamase OXA-10	β-lactams
bla$_{\text{OXA-16}}$	AF043100	ND	Beta-lactamase OXA-16	β-lactams
bla$_{\text{SCO-1}}$	EF063111	NG_049978.1	Class A beta-lactamase SCO-1	β-lactams
bla$_{\text{SHV-1}}$	AF148850	NG_049989.1	Broad-spectrum class A beta-lactamase SHV-1	β-lactams
bla$_{\text{SHV-101}}$	EU155018	ND	Broad-spectrum class A beta-lactamase SHV-101	β-lactams
bla$_{\text{SHV-102}}$	EU024485	ND	Extended-spectrum class A beta-lactamase SHV-102	β-lactams
bla$_{\text{SHV-106}}$	AM922307	NG_049996.1	Extended-spectrum class A beta-lactamase SHV-106	β-lactams
bla$_{\text{SHV-108}}$	AM922309	NG_049998.1	Broad-spectrum class A beta-lactamase SHV-108	β-lactams
bla$_{\text{SHV-11}}$	X98101	NG_050000.1	Broad-spectrum class A beta-lactamase SHV-11	β-lactams
bla$_{\text{SHV-110}}$	HQ877615	NG_050001.1	Class A beta-lactamase SHV-110	β-lactams
bla$_{\text{SHV-12}}$	KF976405	NG_050590.1	Extended-spectrum class A beta-lactamase SHV-12	β-lactams
bla$_{\text{SHV-120}}$	JF812965	ND	Extended-spectrum class A beta-lactamase SHV-120	β-lactams
bla$_{\text{SHV-129}}$	GU827715	ND	Extended-spectrum class A beta-lactamase SHV-129	β-lactams
bla$_{\text{SHV-13}}$	AF164577	ND	Extended-spectrum class A beta-lactamase SHV-13	β-lactams
bla$_{\text{SHV-133}}$	AB551737	ND	Broad-spectrum class A beta-lactamase SHV-133	β-lactams

bla$_{SHV-141}$	JQ388884	NG_050015.1	Broad-spectrum class A beta-lactamase SHV-141	β-lactams
bla$_{SHV-142}$	JQ029959	ND	Broad-spectrum class A beta-lactamase SHV-142	β-lactams
bla$_{SHV-145}$	JX013655	NG_050019.1	Broad-spectrum class A beta-lactamase SHV-145	β-lactams
bla$_{SHV-148}$	JX121115	ND	Broad-spectrum class A beta-lactamase SHV-148	β-lactams
bla$_{SHV-15}$	AJ011428	ND	Extended-spectrum class A beta-lactamase SHV-15	β-lactams
bla$_{SHV-153}$	JX121120	ND	Extended-spectrum class A beta-lactamase SHV-153	β-lactams
bla$_{SHV-155}$	JX121122	NG_050030.1	Class A beta-lactamase SHV-155	β-lactams
bla$_{SHV-158}$	JX121125	NG_050033.1	Class A beta-lactamase SHV-158	β-lactams
bla$_{SHV-159}$	JX121126	ND	Class A beta-lactamase SHV-159	β-lactams
bla$_{SHV-160}$	JX121127	ND	Extended-spectrum class A beta-lactamase SHV-160	β-lactams
bla$_{SHV-165}$	ND	NG_050041.1	Extended-spectrum class A beta-lactamase SHV-165	β-lactams
bla$_{SHV-172}$	KF513177	ND	Class A beta-lactamase SHV-172	β-lactams
bla$_{SHV-179}$	KF705208	ND	Broad-spectrum class A beta-lactamase SHV-179	β-lactams
bla$_{SHV-182}$	KP050489	ND	Class A beta-lactamase SHV-182	β-lactams
bla$_{SHV-185}$	KM233164	ND	Broad-spectrum class A beta-lactamase SHV-185	β-lactams
bla$_{SHV-187}$	LN515533	NG_050053.1	Extended-spectrum class A beta-lactamase SHV-187	β-lactams
bla$_{SHV-190}$	KP868753	NG_050056.1	Class A beta-lactamase SHV-190	β-lactams
bla$_{SHV-191}$	KP868754	ND	Class A beta-lactamase SHV-191	β-lactams
bla$_{SHV-194}$	KX421191	ND	Class A beta-lactamase SHV-194	β-lactams
bla$_{SHV-199}$	MF373391	ND	Class A beta-lactamase SHV-199	β-lactams
bla$_{SHV-2}$	AF148851	ND	Extended-spectrum class A beta-lactamase SHV-2	β-lactams
bla$_{SHV-212}$	ND	NG_062284.1	Class A beta-lactamase SHV-212	β-lactams

bla$_{SHV-215}$	ND	NG_062287.1	Class A beta-lactamase SHV-215	β-lactams
bla$_{SHV-25}$	AF208796	ND	Broad-spectrum class A beta-lactamase SHV-25	β-lactams
bla$_{SHV-26}$	AF227204	ND	Inhibitor-resistant broad-spectrum class A beta-lactamase SHV-26	β-lactams
bla$_{SHV-27}$	AF293345	NG_050064.1	Broad-spectrum class A beta-lactamase SHV-27	β-lactams
bla$_{SHV-28}$	AF299299[4]	NG_051877.1[4]	Broad-spectrum class A beta-lactamase SHV-28	β-lactams
bla$_{SHV-3}$	KX092356	ND	Extended-spectrum class A beta-lactamase SHV-3	β-lactams
bla$_{SHV-30}$	AY661885	NG_050069.1	Extended-spectrum class A beta-lactamase SHV-30	β-lactams
bla$_{SHV-31}$	AY277255	ND	Extended-spectrum class A beta-lactamase SHV-31	β-lactams
bla$_{SHV-33}$	AY037779	NG_050072.1	Broad-spectrum class A beta-lactamase SHV-33	β-lactams
bla$_{SHV-36}$	AF467947	NG_050075.1	Broad-spectrum class A beta-lactamase SHV-36	β-lactams
bla$_{SHV-38}$	AY079099	ND	Extended-spectrum class A beta-lactamase SHV-38	β-lactams
bla$_{SHV-40}$	AF535128	ND	Broad-spectrum class A beta-lactamase SHV-40	β-lactams
bla$_{SHV-44}$	AY259119	ND	Broad-spectrum class A beta-lactamase SHV-44	β-lactams
bla$_{SHV-46}$	AY210887	ND	Extended-spectrum class A beta-lactamase SHV-46	β-lactams
bla$_{SHV-48}$	AY263404	ND	Broad-spectrum class A beta-lactamase SHV-48	β-lactams
bla$_{SHV-49}$	AY528718	ND	Inhibitor-resistant broad-spectrum class A beta-lactamase SHV-49	β-lactams
bla$_{SHV-5}$	X55640	ND	Extended-spectrum class A beta-lactamase SHV-5	β-lactams
bla$_{SHV-50}$	AY288915	ND	Broad-spectrum class A beta-lactamase SHV-50	β-lactams
bla$_{SHV-56}$	EU586041	ND	Inhibitor-resistant broad-spectrum class A beta-lactamase SHV-56	β-lactams
bla$_{SHV-61}$	AJ866284	ND	Broad-spectrum class A beta-lactamase SHV-61	β-lactams
bla$_{SHV-66}$	DQ174306	ND	Extended-spectrum class A beta-lactamase SHV-66	β-lactams
bla$_{SHV-67}$	DQ174307	ND	Class A beta-lactamase SHV-67	β-lactams

bla$_{SHV-69}$	DQ174308	ND	Class A beta-lactamase SHV-69	β-lactams
bla$_{SHV-70}$	DQ013287	ND	Extended-spectrum class A beta-lactamase SHV-70	β-lactams
bla$_{SHV-76}$	AM176551	NG_050111.1	Broad-spectrum class A beta-lactamase SHV-76	β-lactams
bla$_{SHV-78}$	AM176553	ND	Broad-spectrum class A beta-lactamase SHV-78	β-lactams
bla$_{SHV-79}$	AM176554	ND	Broad-spectrum class A beta-lactamase SHV-79	β-lactams
bla$_{SHV-81}$	AM176556	ND	Broad-spectrum class A beta-lactamase SHV-81	β-lactams
bla$_{SHV-85}$	DQ322460	ND	Broad-spectrum class A beta-lactamase SHV-85	β-lactams
bla$_{SHV-86}$	DQ328802	ND	Extended-spectrum class A beta-lactamase SHV-86	β-lactams
bla$_{SHV-89}$	DQ193536	ND	Broad-spectrum class A beta-lactamase SHV-89	β-lactams
bla$_{SHV-94}$	EF373970	ND	Class A beta-lactamase SHV-94	β-lactams
bla$_{SHV-96}$	EF373971	ND	Class A beta-lactamase SHV-96	β-lactams
bla$_{SHV-98}$	AM941844	ND	Broad-spectrum class A beta-lactamase SHV-98	β-lactams
bla$_{TEM-1}$/*bla*$_{TEM-1B}$	AY458016	NG_050145.1	Broad-spectrum class A beta-lactamase TEM-1	β-lactams
bla$_{TEM-12}$	ND	NG_050163.1	Extended-spectrum class A beta-lactamase TEM-12	β-lactams
bla$_{TEM-104}$	AF516719	ND	Class A beta-lactamase TEM-104	β-lactams
bla$_{TEM-122}$	AY307100	NG_050166.1	Inhibitor-resistant broad-spectrum class A beta-lactamase TEM-122	β-lactams
bla$_{TEM-126}$	AY628199	ND	Extended-spectrum class A beta-lactamase TEM-126	β-lactams
bla$_{TEM-135}$	GQ896333	ND	Broad-spectrum class A beta-lactamase TEM-135	β-lactams
bla$_{TEM-141}$	AY956335	ND	Broad-spectrum class A beta-lactamase TEM-141	β-lactams
bla$_{TEM-148}$	AM087454	ND	Class A beta-lactamase TEM-148	β-lactams
bla$_{TEM-150}$	AM183304	NG_050194.1	Class A beta-lactamase TEM-150	β-lactams

bla$_{TEM-163}$	EU815939	ND	Broad-spectrum class A beta-lactamase TEM-163	β-lactams
bla$_{TEM-164}$	EU274580	ND	Extended-spectrum class A beta-lactamase TEM-164	β-lactams
bla$_{TEM-168}$	FJ919776	ND	Extended-spectrum class A beta-lactamase TEM-168	β-lactams
bla$_{TEM-171}$	GQ149347	ND	Class A beta-lactamase TEM-171	β-lactams
bla$_{TEM-176}$	GU550123	ND	Class A beta-lactamase TEM-176	β-lactams
bla$_{TEM-181}$	KM977568	ND	Class A beta-lactamase TEM-181	β-lactams
bla$_{TEM-183}$	HQ529916	ND	Broad-spectrum class A beta-lactamase TEM-183	β-lactams
bla$_{TEM-198}$	AB700703	ND	Class A beta-lactamase TEM-198	β-lactams
bla$_{TEM-1A}$	HM749966	ND	TEM-1 beta-lactamase	β-lactams
bla$_{TEM-1C}$	FJ560503	ND	TEM beta lactamase	β-lactams
bla$_{TEM-1D}$	AF188200	ND	Beta-lactamase variant TEM-1D	β-lactams
bla$_{TEM-206}$	KC783461	ND	Broad-spectrum class A beta-lactamase TEM-206	β-lactams
bla$_{TEM-207}$	KC818234	ND	Extended-spectrum class A beta-lactamase TEM-207	β-lactams
bla$_{TEM-209}$	KF240808	ND	Class A beta-lactamase TEM-209	β-lactams
bla$_{TEM-210}$	KJ484630	ND	Class A beta-lactamase TEM-210	β-lactams
bla$_{TEM-214}$	KP050491	ND	Class A beta-lactamase TEM-214	β-lactams
bla$_{TEM-216}$	KF944358	ND	Class A beta-lactamase TEM-216	β-lactams
bla$_{TEM-217}$	HG934763	ND	Class A beta-lactamase TEM-217	β-lactams
bla$_{TEM-220}$	KM998962	ND	Class A beta-lactamase TEM-220	β-lactams
bla$_{TEM-230}$	MG821377	ND	Class A beta-lactamase TEM-230	β-lactams
bla$_{TEM-231}$	MG821378	ND	Class A beta-lactamase TEM-231	β-lactams
bla$_{TEM-234}$	MH243353	ND	Class A beta-lactamase TEM-234	β-lactams

bla_{TEM-29}	DQ269440	ND	Extended-spectrum class A beta-lactamase TEM-29	β-lactams
bla_{TEM-30}	AJ437107	ND	Inhibitor-resistant broad-spectrum class A beta-lactamase TEM-30	β-lactams
bla_{TEM-33}	GU371926	ND	Inhibitor-resistant broad-spectrum class A beta-lactamase TEM-33	β-lactams
bla_{TEM-34}	KC292503	ND	Inhibitor-resistant broad-spectrum class A beta-lactamase TEM-34	β-lactams
bla_{TEM-40}	FR717535	ND	Inhibitor-resistant broad-spectrum class A beta-lactamase TEM-40	β-lactams
bla_{TEM-54}	AF104442	ND	Inhibitor-resistant broad-spectrum class A beta-lactamase TEM-54	β-lactams
bla_{TEM-55}	DQ286729	ND	Broad-spectrum class A beta-lactamase TEM-55	β-lactams
bla_{TEM-57}	FJ405211	ND	Broad-spectrum class A beta-lactamase TEM-57	β-lactams
bla_{TEM-70}	AF188199	ND	Class A beta-lactamase TEM-70	β-lactams
bla_{TEM-79}	AF190692	ND	Inhibitor-resistant broad-spectrum class A beta-lactamase TEM-79	β-lactams
bla_{TEM-90}	AF351241	ND	Broad-spectrum class A beta-lactamase TEM-90	β-lactams
bla_{TEM-93}	AJ318093	ND	Extended-spectrum class A beta-lactamase TEM-93	β-lactams
bla_{TEM-97}	AF397067	ND	Class A beta-lactamase TEM-97	β-lactams
bla_{TEM-98}	AF397068	ND	Class A beta-lactamase TEM-98	β-lactams
bla_{VEB-1}	DQ393569[3]	NG_050317.1[3]	Extended-spectrum class A beta-lactamase VEB-1	β-lactams
ble_{MBL}	ND	NG_047559.1	Bleomycin binding protein Ble-MBL	Bleomycin
mcr-9/mcr-9.1	NZ_NAAN01000063.1[3]	NG_064792.1[3]	Phosphoethanolamine--lipid A transferase MCR-9.1	Colistin
qacE	X68232	ND	Quaternary ammonium compound efflux SMR transporter qace	Desinfectant
sitABCD	AY598030	ND	Periplasmic binding protein (sitA), ATP-binding component(sitB), inner membrane component (sitC), inner membrane component(sitD)	Desinfectant
sat2_gen	ND	NG_048068.1	Streptothricin N-acetyltransferase Sat2	Streptotricin
cat	M35190	ND	Type A-13 chloramphenicol O-acetyltransferase	Fenicol

catA1	V00622	NG_047582.1	Type A-1 chloramphenicol O-acetyltransferase	Fenicol
	ND	NG_047586.1		
catA13	ND	NG_055993.2	Type A-13 chloramphenicol O-acetyltransferase	Fenicol
catA2	X53796[4]	NG_047596.1[4]	Type A-2 chloramphenicol O-acetyltransferase catii	Fenicol
catB	ND	NG_047599.1	Type B chloramphenicol O-acetyltransferase	Fenicol
catB11	ND	NG_050948.1	Type B-2 chloramphenicol O-acetyltransferase catb11	Fenicol
catB3	AJ009818	ND	Type B-3 chloramphenicol O-acetyltransferase catb3	Fenicol
	U13880	NG_047604.1		
cmlA1	M64556[4]	NG_047648.1[4]	Chloramphenicol efflux MFS transporter cmla1	Fenicol
cmlA5	ND	NG_051436.1	Chloramphenicol efflux MFS transporter cmla5	Fenicol
floR	AF118107[4]	NG_047865.1[4]	Chloramphenicol/florfenicol efflux MFS transporter flor	Fenicol
	ND	NG_047869.1		
floR2	ND	NG_047875.1	Chloramphenicol/florfenicol efflux MFS transporter flor2	Fenicol
OqxA/oqxA	EU370913	NG_048024.1	Multidrug efflux RND transporter periplasmic adaptor subunit oqxa	Fenicol/Quinolone
OqxB/oqxB	EU370913	NG_048025.1	Multidrug efflux RND transporter permease subunit oqxb	Fenicol/Quinolone
oqxA10	ND	NG_050418.1	Multidrug efflux RND transporter periplasmic adaptor subunit oqxa10	Fenicol/Quinolone
oqxA11	ND	NG_050419.1	Multidrug efflux RND transporter periplasmic adaptor subunit oqxa11	Fenicol/Quinolone
oqxA3	ND	NG_050421.1	Multidrug efflux RND transporter periplasmic adaptor subunit oqxa3	Fenicol/Quinolone
oqxA5	ND	NG_050423.1	Multidrug efflux RND transporter periplasmic adaptor subunit oqxa5	Fenicol/Quinolone
oqxA6	ND	NG_050424.1	Multidrug efflux RND transporter periplasmic adaptor subunit oqxa6	Fenicol/Quinolone
oqxA8	ND	NG_050426.1	Multidrug efflux RND transporter periplasmic adaptor subunit oqxa8	Fenicol/Quinolone
oqxA9	ND	NG_050427.1	Multidrug efflux RND transporter periplasmic adaptor subunit oqxa9	Fenicol/Quinolone
oqxB10	ND	NG_050428.1	Multidrug efflux RND transporter permease subunit oqxb10	Fenicol/Quinolone
oqxB11	ND	NG_050429.1	Multidrug efflux RND transporter permease subunit oqxb11	Fenicol/Quinolone
oqxB12	ND	NG_050430.1	Multidrug efflux RND transporter permease subunit oqxb12	Fenicol/Quinolone

oqxB13	ND	NG_050431.1	Multidrug efflux RND transporter permease subunit oqxb13	Fenicol/Quinolone
oqxB17	ND	NG_050435.1	Multidrug efflux RND transporter permease subunit oqxb17	Fenicol/Quinolone
oqxB18	ND	NG_050436.1	Multidrug efflux RND transporter permease subunit oqxb18	Fenicol/Quinolone
oqxB19	ND	NG_050437.1	Multidrug efflux RND transporter permease subunit oqxb19	Fenicol/Quinolone
oqxB20	ND	NG_050439.1	Multidrug efflux RND transporter permease subunit oqxb20	Fenicol/Quinolone
oqxB24	ND	NG_050443.1	Multidrug efflux RND transporter permease subunit oqxb24	Fenicol/Quinolone
oqxB25	ND	NG_050444.1	Multidrug efflux RND transporter permease subunit oqxb25	Fenicol/Quinolone
oqxB32	ND	NG_050452.1	Multidrug efflux RND transporter permease subunit oqxb32	Fenicol/Quinolone
oqxB4	ND	NG_050453.1	Multidrug efflux RND transporter permease subunit oqxb4	Fenicol/Quinolone
fosA_gen/fosA	ACZD01000244	NG_047881.1	Fosa5 family fosfomycin resistance glutathione transferase	Fosfomycin
	AFBO01000747	NG_047882.1	Fosa5 family fosfomycin resistance glutathione transferase	Fosfomycin
	ACWO01000079	NG_047884.1	Fosfomycin resistance glutathione transferase fosa10	Fosfomycin
fosA5	EU195449	NG_050409.1	Fosfomycin resistance glutathione transferase fosa5	Fosfomycin
fosA6	ND	NG_051497.1	Fosfomycin resistance glutathione transferase fosa6	Fosfomycin
ere(A)	AF099140	NG_047763.1	Erea family erythromycin esterase	Macrolide
	FN396877	NG_047770.1		
ere(B)	X03988	NG_047768.1	Ereb family erythromycin esterase	Macrolide
erm(B)	JN899585	NG_047804.1	23S rrna (adenine(2058)-N(6))-methyltransferase Erm(B)	Macrolide
mph(A)	D16251[4]	NG_047986.1[4]	Mph(A) family macrolide 2'-phosphotransferase	Macrolide
mph(E)	DQ839391[3]	NG_064660.1[3]	Mph(E) family macrolide 2'-phosphotransferase	Macrolide
msr(E)	FR751518[3]	NG_048007.1[3]	ABC-F type ribosomal protection protein Msr(E)	Macrolide
qnrA6	DQ151889	NG_050467.1	Quinolone resistance pentapeptide repeat protein qnra6	Quinolone
qnrB1	DQ351241	NG_050469.1	Quinolone resistance pentapeptide repeat protein qnrb1	Quinolone
qnrB19	EU432277	NG_050479.1	Quinolone resistance pentapeptide repeat protein qnrb19	Quinolone
qnrB4	DQ303921	NG_050502.1	Quinolone resistance pentapeptide repeat protein qnrb4	Quinolone

qnrB9	EF526508	NG_050540.1	Quinolone resistance pentapeptide repeat protein qnrb9	Quinolone
qnrE1	KY073238	NG_054677.1	Quinolone resistance pentapeptide repeat protein qnre1	Quinolone
qnrS1	AB187515	NG_050543.1	Quinolone resistance pentapeptide repeat protein qnrs1	Quinolone
ARR-2	HQ141279[3]	NG_048580.1[3]	NAD(+)--rifampin ADP-ribosyltransferase Arr-2	Rifamycin
arr-269927220	ND	NG_047480.1	NAD(+)--rifampin ADP-ribosyltransferase	Rifamycin
ARR-3	JF806499[3]	NG_048581.1[3]	NAD(+)--rifampin ADP-ribosyltransferase Arr-3	Rifamycin
ARR-6	JF922883	ND	NAD(+)--rifampin ADP-ribosyltransferase Arr-6	Rifamycin
sul1	AJ746361	ND	Sulfonamide-resistant dihydropteroate synthase Sul1	Sulfonamide
sul1	AM040449	ND	Sulfonamide-resistant dihydropteroate synthase Sul1	Sulfonamide
sul1	AM746675	ND	Sulfonamide-resistant dihydropteroate synthase Sul1	Sulfonamide
sul1	AY260546	ND	Sulfonamide-resistant dihydropteroate synthase Sul1	Sulfonamide
sul1	AY522923	NG_048089.1	Sulfonamide-resistant dihydropteroate synthase Sul1	Sulfonamide
sul1	AY963803	ND	Sulfonamide-resistant dihydropteroate synthase Sul1	Sulfonamide
sul1	DQ125241	ND	Sulfonamide-resistant dihydropteroate synthase Sul1	Sulfonamide
sul1	DQ914960	ND	Sulfonamide-resistant dihydropteroate synthase Sul1	Sulfonamide
sul1	EF667294	ND	Sulfonamide-resistant dihydropteroate synthase Sul1	Sulfonamide
sul1	EU117158	ND	Sulfonamide-resistant dihydropteroate synthase Sul1	Sulfonamide
sul1	EU780013	ND	Sulfonamide-resistant dihydropteroate synthase Sul1	Sulfonamide
sul1	GU562437	ND	Sulfonamide-resistant dihydropteroate synthase Sul1	Sulfonamide
sul1	JF262165	NG_048102.1	Sulfonamide-resistant dihydropteroate synthase Sul1	Sulfonamide
sul1	U12338	NG_048082.1	Sulfonamide-resistant dihydropteroate synthase Sul1	Sulfonamide
sul1	X15024	ND	Sulfonamide-resistant dihydropteroate synthase Sul1	Sulfonamide
sul2	AJ830710[4]	NG_048118.1[4]	Sulfonamide-resistant dihydropteroate synthase Sul2	Sulfonamide
sul2	AY034138	NG_051852.1	Sulfonamide-resistant dihydropteroate synthase Sul2	Sulfonamide
sul2	FN995456	ND	Sulfonamide-resistant dihydropteroate synthase Sul2	Sulfonamide

	HQ840942	ND		
sul3	AJ459418	NG_048120.1	Sulfonamide-resistant dihydropteroate synthase Sul3	Sulfonamide
tet(A)	AF534183	NG_048153.1	Tetracycline efflux MFS transporter Tet(A)	Tetracycline
	AJ517790[3]	NG_048154.1[3]		
	ND	NG_048157.1		
tet(B)	AF326777	NG_048163.1	Tetracycline efflux MFS transporter Tet(B)	Tetracycline
	AP000342	NG_048161.1		
tet(D)	AF467077[4]	NG_048184.1[4]	Tetracycline efflux MFS transporter Tet(D)	Tetracycline
tet(G)	AF133140[3]	NG_051907.1[3]	Tetracycline efflux MFS transporter Tet(G)	Tetracycline
dfrA1	AF203818	ND	Trimethoprim-resistant dihydrofolate reductase dfra1	Trimethoprim
	AJ238350	NG_047677.1		
	X00926	NG_047676.1		
dfrA12	AM040708	NG_047689.1	Trimethoprim-resistant dihydrofolate reductase dfra12	Trimethoprim
	EU650399	ND		
	FR875302	NG_051893.1		
dfrA14	AF393510	NG_047696.1	Trimethoprim-resistant dihydrofolate reductase dfra14	Trimethoprim
	KF921535	NG_056035.1		
dfrA17	FJ460238[4]	NG_047710.1[4]	Trimethoprim-resistant dihydrofolate reductase dfra17	Trimethoprim
dfrA19	EU855687[3]	NG_050403.1[3]	Trimethoprim-resistant dihydrofolate reductase dfra19	Trimethoprim
dfrA21	AY552589	NG_047715.1	Trimethoprim-resistant dihydrofolate reductase dfra21	Trimethoprim
dfrA23	AJ746361	NG_047719.1	Trimethoprim-resistant dihydrofolate reductase dfra23	Trimethoprim
dfrA27	FJ459817[3]	NG_047723.1[3]	Trimethoprim-resistant dihydrofolate reductase dfra27	Trimethoprim
dfrA30	AM997279	NG_047727.1	Trimethoprim-resistant dihydrofolate reductase dfra30	Trimethoprim
dfrA5	X12868[3]	NG_051699.1[3]	Trimethoprim-resistant dihydrofolate reductase dfra5	Trimethoprim

dfrA8	U10186	NG_047741.1	Trimethoprim-resistant dihydrofolate reductase dfra8	Trimethoprim
