# Supplementary material for: Pipeline validation for the identification of antimicrobial-resistant genes in carbapenem-resistant Klebsiella pneumoniae
Source: Sci Rep. 2023 Sep 14;13:15189. doi: 10.1038/s41598-023-42154-6 (PMC10502106; doi:10.1038/s41598-023-42154-6)
Supplement: Supplementary file 3 — Supplementary Information 3. [file 41598_2023_42154_MOESM3_ESM.pdf]

Table S3: Antimicrobial resistance genes identified in each SRA.

SRA	Contig	ResFinder	ABRicate
ERR2796902	NODE_124_length_518cov_22.066878	*aadA2b*	*aadA2*
		qacE	
		sul1	*sul1*
	NODE_140_length_3486_cov_7.578744	*mph(A)*	*mph(A)*
	NODE_165_length_1498_cov_13.638220	$bla_{SHV-182}$	$bla_{SHV-158}$
	NODE_168_length_1443_cov_29.610182	bla_{OXA-9}	bla_{OXA-9}
	NODE_169_length_1406_cov_25.258014	*dfrA12*	*dfrA12*
	NODE_177_length_1128_cov_48.031968	*aac(6')-Ib*	*aac(6')-Ib-AKT*
		aac(6')-Ib-cr	
	NODE_183_length_923_cov_29.804020	bla_{TEM-1A}	bla_{TEM-1}
	NODE_43_length_40804_cov_18.230499	*catA1*	*catA1*
	NODE_44_length_3990cov_14.052145	*fosA*	*fosA6*
	NODE_57_length_29547_cov_17.450850	bla_{KPC-3}	bla_{KPC-3}
	NODE_8_length_149503_cov_14.763764	*oqxA*	*oqxA*
		OqxA	
		oqxB	*oqxB*
		OqxB	
ERR2796903	NODE_104_length_1605_cov_50.928281	*dfrA12*	*dfrA12*
	NODE_110_length_1292_cov_20.883262	*aph(3')-Ia*	*aph(3')-Ia*
	NODE_28_length_70622_cov_43.003178	*catA1*	*catA1*
	NODE_32_length_62989_cov_60.135694	bla_{KPC-3}	bla_{KPC-3}
	NODE_4_length_270453_cov_40.644944	*fosA*	*fosA6*
	NODE_49_length_20503_cov_33.060512	*oqxA*	*oqxA*
		OqxA	
		oqxB	*oqxB*
		OqxB	
	NODE_62_length_13389_cov_54.961846	*aac(6')-Ib*	*aac(6')-Ib-AKT*
		aac(6')-Ib-cr	
	NODE_84_length_3484_cov_50.426571	*mph(A)*	*mph(A)*
	NODE_86_length_3320_cov_97.356091	$bla_{SHV-182}$	$bla_{SHV-158}$
	NODE_94_length_2234_cov_65.365923	*aadA2*	
		qacE	
		sul1	*sul1*
ERR2796904	NODE_114_length_3484_cov_15.325588	*mph(A)*	*mph(A)*
	NODE_124_length_2234_cov_22.221642	*aadA2*	
		qacE	
		sul1	*sul1*
	NODE_136_length_1605_cov_24.205007	*dfrA12*	*dfrA12*
	NODE_146_length_1293_cov_16.586621	*aph(3')-Ia*	*aph(3')-Ia*
	NODE_1length_131562_cov_12.385088	*OqxA*	*oqxA*

		oqxA	
		OqxB	*oqxB*
		oqxB	
	NODE_2_length_270452_cov_15.214072	*fosA*	*fosA6*
	NODE_42_length_38212_cov_9.070159	*bla*$_{SHV-182}$	*bla*$_{SHV-158}$
	NODE_77_length_14918_cov_16.121155	*catA1*	*catA1*
	NODE_79_length_13389_cov_27.166792	*aac(6')-Ib*	*aac(6')-Ib-AKT*
		aac(6')-Ib-cr	
	NODE_88_length_9910_cov_53.516508	*bla*$_{KPC-3}$	*bla*$_{KPC-3}$
ERR2796907	NODE_32_length_47749_cov_54.744257	*bla*$_{CMY-16}$	*bla*$_{CMY-16}$
		bla$_{CMY-4}$	
	NODE_37_length_29113_cov_46.943524	*sul2*	*sul2*
	NODE_39_length_25226_cov_41.009722	*aadA1*	*aadA1*
		aadA1	
		ARR-2	*arr-2*
		bla$_{OXA-10}$	*bla*$_{OXA-10}$
		cmlA1	*cmlA5*
		dfrA14	*dfrA14*
		qacE	
		qnrA6	*qnrA6*
		sul1	*sul1*
		sul1	*sul1*
	NODE_48_length_8932_cov_41.562976	*floR*	*floR*
		tet(A)	*tet(A)*
	NODE_56_length_4020_cov_61.397380	*bla*$_{CTX-M-15}$	*bla*$_{CTX-M-15}$
	NODE_60_length_2847_cov_68.985294	*aac(3)-IIa*	*aac(3)-IIe*
	NODE_67_length_1714_cov_125.721487	*aph(3'')-Ib*	*aph(3'')-Ib*
		aph(3'')-Ib	
		aph(3'')-Ib	
		aph(3'')-Ib	
		aph(6)-Id	*aph(6)-Id*
	NODE_6length_2436_cov_43.113036	*aac(6')-Ib-cr*	*aac(6')-Ib-D181Y*
		aac(6')-Ib-cr	
		bla$_{OXA-1}$	*bla*$_{OXA-1}$
		catB3	
		catB3	
	NODE_7_length_222970_cov_30.582513	*fosA*	*fosA6*
	NODE_8_length_213156_cov_25.102845	*bla*$_{SHV-187}$	*bla*$_{SHV-187}$
	NODE_80_length_1082_cov_25.894241	*aph(3')-Ia*	*aph(3')-Ia*
	NODE_84_length_1032_cov_74.902502	*catA1*	*catA1*
	NODE_23_length_82106_cov_48.745386	*bla*$_{KPC-3}$	*bla*$_{KPC-3}$

ERR2796910	NODE_32_length_52299_cov_16.602622	*bla*$_{\text{SHV-182}}$	*bla*$_{\text{SHV-158}}$
	NODE_44_length_33788_cov_26.831823	*fosA*	*fosA6*
	NODE_47_length_31013_cov_22.009033	*oqxA*	*oqxA*
		OqxA	
		oqxB	*oqxB*
		OqxB	
	NODE_63_length_13070_cov_42.850112	*aac(6')-Ib*	*aac(6')-Ib-AKT*
		aac(6')-Ib-cr	
	NODE_79_length_5344_cov_21.673759	*aadA2b*	*aadA2*
		qacE	
		sul1	*sul1*
ERR2796911	NODE_15_length_110778_cov_196.986064	*bla*$_{\text{OXA-232}}$	*bla*$_{\text{OXA-232}}$
		OqxB	
		oqxB	
	NODE_2_length_750363_cov_26.756733	*bla*$_{\text{SHV-106}}$	*bla*$_{\text{SHV-212}}$
		bla$_{\text{SHV-28}}$	
	NODE_28_length_33318_cov_38.995993	*OqxA*	
		oqxA	*oqxA10*
	NODE_34_length_1563cov_22.386223	*sitABCD*	
	NODE_36_length_6394_cov_420.970480	*bla*$_{\text{TEM-1B}}$	*bla*$_{\text{TEM-1}}$
	NODE_37_length_595cov_25.313702	*aac(6')-Ib-cr*	*aac(6')-Ib-G*
		aac(6')-Ib-Hangzhou	*catB*
		rmtF	*rmtF1*
	NODE_4_length_355269_cov_41.388895	*fosA*	*fosA_gen*
	NODE_44_length_3635_cov_21.987457	*qacE*	
		sul1	*sul1*
	NODE_45_length_3485_cov_20.489875	*mph(A)*	*mph(A)*
	NODE_46_length_3199_cov_8.701172	*erm(B)*	*erm(B)*
	NODE_52_length_2194_cov_22.705370	*aadA2*	*aadA2*
		dfrA12	*dfrA12*
	NODE_67_length_749_cov_31.059486	*ARR-2*	*arr-2*
	NODE_69_length_719_cov_17.329392	*catA1*	
	NODE_6length_1082_cov_80.780105	*bla*$_{\text{CTX-M-15}}$	*bla*$_{\text{CTX-M-15}}$
ERR2796912	NODE_17_length_8660cov_23.605592	*OqxA*	
		oqxA	*oqxA5*
		OqxB	
		oqxB	*oqxB19*
	NODE_1length_150458_cov_21.447932	*aph(3'')-Ib*	*aph(3'')-Ib*
		aph(6)-Id	*aph(6)-Id*
		bla$_{\text{TEM-1B}}$	*bla*$_{\text{TEM-1}}$
		sul2	*sul2*

	NODE_3_length_378117_cov_27.943128	*fosA*	*fosA6*
	NODE_33_length_13793_cov_22.251207	*qnrB1*	*qnrB1*
		tet(A)	*tet(A)*
	NODE_36_length_6980_cov_20.592004	*dfrA14*	*dfrA14*
	NODE_38_length_5906_cov_19.026302	*bla*$_{\text{CTX-M-15}}$	*bla*$_{\text{CTX-M-15}}$
	NODE_42_length_223cov_45.277354	*bla*$_{\text{OXA-48}}$	*bla*$_{\text{OXA-48}}$
	NODE_4length_2440_cov_16.952875	*aac(6')-Ib-cr*	*aac(6')-Ib-D181Y*
		aac(6')-Ib-cr	
		bla$_{\text{OXA-1}}$	*bla*$_{\text{OXA-1}}$
		catB3	
		catB3	
	NODE_length_1304358_cov_19.454759	*bla*$_{\text{SHV-106}}$	*bla*$_{\text{SHV-106}}$
		bla$_{\text{SHV-28}}$	
ERR2796917	NODE_22_length_57158_cov_57.294577	*oqxA*	*oqxA6*
		OqxA	
		oqxB	*oqxB10*
		OqxB	
	NODE_33_length_8602_cov_57.587847	*aadA1*	*aadA1*
		ant(2")-Ia	*ant(2")-Ia*
		qacE	
		sul1	*sul1*
	NODE_37_length_4120_cov_68.842474	*bla*$_{\text{CTX-M-15}}$	*bla*$_{\text{CTX-M-15}}$
	NODE_38_length_381cov_74.454126	*tet(A)*	*tet(A)*
	NODE_42_length_2852_cov_86.851376	*aac(3)-IIa*	*aac(3)-IIe*
	NODE_43_length_2437_cov_50.770996	*aac(6')-Ib-cr*	*aac(6')-Ib-D181Y*
		aac(6')-Ib-cr	
		bla$_{\text{OXA-1}}$	*bla*$_{\text{OXA-1}}$
		catB3	
		catB3	
	NODE_44_length_223cov_71.789652	*bla*$_{\text{OXA-48}}$	*bla*$_{\text{OXA-48}}$
	NODE_5_length_34863cov_68.303563	*fosA*	*fosA_gen*
	NODE_length_796494_cov_50.594112	*bla*$_{\text{SHV-1}}$	*bla*$_{\text{SHV-1}}$
ERR2796919	NODE_104_length_2256_cov_53.433067	*aac(3)-IIa*	*aac(3)-IIe*
	NODE_105_length_223cov_111.602629	*bla*$_{\text{OXA-48}}$	*bla*$_{\text{OXA-48}}$
	NODE_123_length_1265_cov_126.009666	*bla*$_{\text{TEM-1A}}$	*bla*$_{\text{TEM-150}}$
	NODE_145_length_797_cov_119.729851	*dfrA14*	*dfrA14*
	NODE_22_length_82427_cov_25.673985	*bla*$_{\text{SHV-106}}$	*bla*$_{\text{SHV-212}}$
		bla$_{\text{SHV-28}}$	
	NODE_26_length_73870_cov_33.866984	*OqxA*	
		oqxA	*oqxA10*
		OqxB	

		oqxB	*oqxB17*
	NODE_35_length_47749_cov_73.251480	*bla*$_{\text{CMY-16}}$	*bla*$_{\text{CMY-16}}$
		bla$_{\text{CMY-4}}$	
	NODE_36_length_46892_cov_39.424121	*fosA*	*fosA_gen*
	NODE_44_length_38060_cov_68.784278	*aph(3'')-Ib*	*aph(3'')-Ib*
		aph(6)-Id	*aph(6)-Id*
		floR	*floR*
		sul2	*sul2*
		tet(A)	*tet(A)*
	NODE_58_length_16089_cov_42.035146	*aadA1*	*aadA1*
		aadA1	
		armA	*armA*
		ARR-2	*arr-2*
		bla$_{\text{OXA-10}}$	*bla*$_{\text{OXA-10}}$
		cmlA1	*cmlA5*
		mph(E)	*mph(E)*
		msr(E)	*msr(E)*
		qacE	
		sul1	*sul1*
	NODE_7length_8094_cov_44.714573	*bla*$_{\text{CTX-M-15}}$	*bla*$_{\text{CTX-M-15}}$
	NODE_87_length_4086_cov_41.038394	*bla*$_{\text{SCO-1}}$	*bla*$_{\text{SCO-1}}$
ERR2796920	NODE_10_length_195847_cov_42.198789	*oqxA*	*oqxA11*
		OqxA	
		oqxB	*oqxB20*
		OqxB	
	NODE_14_length_152920_cov_44.133789	*fosA*	*fosA6*
	NODE_24_length_48717_cov_51.546038	*ant(3'')-Ia*	
	NODE_5length_4012_cov_1153.015701	*bla*$_{\text{SHV-1}}$	*bla*$_{\text{SHV-1}}$
		bla$_{\text{SHV-102}}$	
		bla$_{\text{SHV-48}}$	
ERR2796923	NODE_3length_67970_cov_23.678773	*bla*$_{\text{SHV-110}}$	*bla*$_{\text{SHV-110}}$
		bla$_{\text{SHV-191}}$	
		bla$_{\text{SHV-27}}$	
	NODE_89_length_223cov_83.554283	*bla*$_{\text{OXA-48}}$	*bla*$_{\text{OXA-48}}$
	NODE_9_length_156900_cov_38.747673	*fosA*	*fosA_gen*
	NODE_length_258824_cov_34.944104	*OqxA*	
		oqxA	*oqxA11*
		OqxB	
		oqxB	*oqxB20*
ERR2796926	NODE_10_length_178576_cov_15.008736	*bla*$_{\text{SHV-106}}$	*bla*$_{\text{SHV-212}}$
		bla$_{\text{SHV-28}}$	
	NODE_2_length_35083cov_19.939573	*fosA*	*fosA_gen*

	NODE_32_length_55158_cov_16.798750	*oqxA*	*oqxA10*
		OqxA	
		oqxB	*oqxB17*
		OqxB	
	NODE_67_length_7970_cov_14.563815	*dfrA14*	*dfrA14*
	NODE_78_length_4349_cov_17.385836	*aac(6')-Ib-cr*	*aac(6')-Ib-AKT*
		aac(6')-Ib-cr	
		aadA1	*aadA1*
		*bla*OXA-9	*bla*OXA-9
		*bla*TEM-1A	*bla*TEM-150
	NODE_80_length_3763_cov_15.610836	*tet(D)*	*tet(D)*
	NODE_85_length_3292_cov_13.779779	*bla*CTX-M-15	*bla*CTX-M-15
	NODE_87_length_2853_cov_15.012472	*aac(3)-IIa*	*aac(3)-IIe*
	NODE_90_length_223cov_36.711620	*bla*OXA-48	*bla*OXA-48
	NODE_94_length_1859_cov_10.725751	*bla*OXA-1	*bla*OXA-1
		catB3	
		catB3	
ERR2796929	NODE_23_length_89842_cov_37.934738	*fosA*	*fosA_gen*
	NODE_3_length_354803_cov_35.312150	*OqxA*	
		oqxA	*oqxA*
		OqxB	
		oqxB	*oqxB*
	NODE_48_length_9613_cov_40.753004	*armA*	*armA*
		mph(E)	*mph(E)*
		msr(E)	*msr(E)*
	NODE_52_length_8478_cov_1363.092324	*aph(3')-VIa*	*aph(3')-VIa*
		aph(6)-Id	
		aph(6)-Id	
	NODE_53_length_7786_cov_53.682857	*qnrS1*	*qnrS1*
	NODE_56_length_5815_cov_53.978376	*dfrA1*	*dfrA1*
		tet(A)	*tet(A)*
	NODE_63_length_3589_cov_49.431542	*aadA5*	*aadA5*
		dfrA17	*dfrA17*
	NODE_68_length_2853_cov_72.595011	*aac(3)-IIa*	*aac(3)-IIe*
	NODE_69_length_2438_cov_46.312852	*aac(6')-Ib-cr*	*aac(6')-Ib-D181Y*
		aac(6')-Ib-cr	
		*bla*OXA-1	*bla*OXA-1
		catB3	
		catB3	
	NODE_70_length_223cov_47.944869	*bla*OXA-48	*bla*OXA-48
	NODE_73_length_1936_cov_45.633499	*bla*CTX-M-15	*bla*CTX-M-15

	NODE_83_length_1202_cov_40.765581	*catA1*	*catA1*
	NODE_85_length_1188_cov_88.059378	*bla*$_{\text{TEM-1B}}$	*bla*$_{\text{TEM-1}}$
	NODE_8length_1316_cov_83.745164	*qacE*	
		sul1	*sul1*
	NODE_9_length_19954cov_25.901612	*bla*$_{\text{SHV-182}}$	*bla*$_{\text{SHV-158}}$
ERR2796930	NODE_23_length_8785cov_64.354430	*fosA*	*fosA_gen*
	NODE_3_length_416260_cov_46.396801	*bla*$_{\text{SHV-145}}$	*bla*$_{\text{SHV-145}}$
		bla$_{\text{SHV-179}}$	
		bla$_{\text{SHV-194}}$	
		bla$_{\text{SHV-199}}$	
		bla$_{\text{SHV-26}}$	
		bla$_{\text{SHV-78}}$	
		bla$_{\text{SHV-98}}$	
	NODE_4_length_347896_cov_60.494092	*OqxA*	
		oqxA	*oqxA11*
		OqxB	
		oqxB	*oqxB20*
	NODE_45_length_17278_cov_57.412629	*qnrB19*	*qnrB19*
	NODE_57_length_6918_cov_75.818878	*bla*$_{\text{KPC-2}}$	*bla*$_{\text{KPC-2}}$
	NODE_62_length_5077_cov_62.624040	*rmtD*	*rmtD1*
	NODE_64_length_3812_cov_131.588060	*aac(6')-Ib*	*aac(6')-Ib-AGKT*
		aac(6')-Ib-cr	
		aadA1	*aadA1*
		bla$_{\text{OXA-9}}$	*bla*$_{\text{OXA-9}}$
	NODE_66_length_3344_cov_58.623251	*cat*	*catA13*
		sul1	*sul1*
	NODE_67_length_3247_cov_145.282051	*qnrE1*	*qnrE1*
	NODE_74_length_2375_cov_103.549377	*bla*$_{\text{CTX-M-8}}$	*bla*$_{\text{CTX-M-8}}$
	NODE_77_length_1539_cov_82.326487	*dfrA8*	*dfrA8*
	NODE_7length_2825_cov_58.364715	*catA1*	*catA1*
	NODE_84_length_977_cov_103.490588	*bla*$_{\text{TEM-1A}}$	*bla*$_{\text{TEM-150}}$
	NODE_32_length_38508_cov_53.105339		*sat2_gen*
ERR2796931	NODE_122_length_16118_cov_47.446439	*fosA*	*fosA6*
	NODE_140_length_13734_cov_26.839274	*qnrB1*	*qnrB1*
		tet(A)	*tet(A)*
	NODE_222_length_6790_cov_18.020561	*dfrA14*	*dfrA14*
	NODE_275_length_4429_cov_17.617155	*bla*$_{\text{CTX-M-15}}$	*bla*$_{\text{CTX-M-15}}$
	NODE_304_length_3188_cov_20.710552	*aph(3'')-Ib*	*aph(3'')-Ib*
		aph(6)-Id	*aph(6)-Id*
		sul2	*sul2*
	NODE_32_length_3669cov_31.338311	*bla*$_{\text{SHV-106}}$	*bla*$_{\text{SHV-106}}$

		bla$_{SHV-28}$	
		bla$_{OXA-48}$	*bla*$_{OXA-48}$
	NODE_344_length_1680_cov_11.594334	*bla*$_{TEM-1B}$	*bla*$_{TEM-1}$
	NODE_384_length_917_cov_34.606329	*aac(6')-Ib-cr*	*aac(6')-Ib-D181Y*
		aac(6')-Ib-cr	
	NODE_407_length_660_cov_19.656660	*catB3*	
		catB3	
	NODE_94_length_19605_cov_37.587483	*oqxA*	*oqxA5*
		OqxA	
		oqxB	*oqxB19*
		OqxB	
ERR2796942	NODE_103_length_1292_cov_21.160515	*aph(3')-Ia*	*aph(3')-Ia*
	NODE_108_length_1128_cov_96.862138	*aac(6')-Ib*	*aac(6')-Ib'*
		aac(6')-Ib-cr	
	NODE_114_length_923_cov_36.045226	*bla*$_{TEM-1A}$	*bla*$_{TEM-1}$
	NODE_33_length_41048_cov_34.057452	*catA1*	*catA1*
	NODE_34_length_40743_cov_44.694775	*bla*$_{KPC-3}$	*bla*$_{KPC-3}$
	NODE_5_length_270452_cov_30.640248	*fosA*	*fosA6*
	NODE_75_length_3484_cov_35.872207	*mph(A)*	*mph(A)*
	NODE_78_length_3320_cov_66.375196	*bla*$_{SHV-182}$	*bla*$_{SHV-158}$
	NODE_87_length_2234_cov_49.541528	*aadA2*	
		aadA2	
		qacE	
		sul1	*sul1*
	NODE_9_length_199790_cov_26.633773	*OqxA*	
		oqxA	*oqxA*
		OqxB	
		oqxB	*oqxB*
	NODE_94_length_1605_cov_41.378890	*dfrA12*	*dfrA12*
	NODE_96_length_1443_cov_40.688450	*bla*$_{OXA-9}$	*bla*$_{OXA-9}$
ERR2796944	NODE_23_length_85215_cov_18.091917	*bla*$_{SHV-182}$	*bla*$_{SHV-158}$
	NODE_25_length_82106_cov_51.913429	*bla*$_{KPC-3}$	*bla*$_{KPC-3}$
	NODE_38_length_33766_cov_27.056274	*fosA*	*fosA6*
	NODE_57_length_13070_cov_47.570115	*aac(6')-Ib*	*aac(6')-Ib-AKT*
		aac(6')-Ib-cr	
	NODE_69_length_5344_cov_23.891700	*aadA2b*	*aadA2*
		qacE	
		sul1	*sul1*
	NODE_length_29573cov_24.528153	*OqxA*	
		oqxA	*oqxA*
		OqxB	

		oqxB	*oqxB*
ERR2796946	NODE_103_length_7733_cov_46.293715	*dfrA14*	*dfrA14*
	NODE_123_length_4086_cov_34.840111	*bla*$_{\text{SCO-1}}$	*bla*$_{\text{SCO-1}}$
	NODE_124_length_376cov_54.780407	*tet(D)*	*tet(D)*
	NODE_126_length_2972_cov_43.558524	*bla*$_{\text{CTX-M-15}}$	*bla*$_{\text{CTX-M-15}}$
	NODE_132_length_2437_cov_34.791775	*aac(6')-Ib-cr*	
		aac(6')-Ib-cr	*aac(6')-Ib-D181Y*
		bla$_{\text{OXA-1}}$	*bla*$_{\text{OXA-1}}$
		catB3	
		catB3	
	NODE_139_length_223cov_71.112383	*bla*$_{\text{OXA-48}}$	*bla*$_{\text{OXA-48}}$
	NODE_170_length_107cov_62.939619	*bla*$_{\text{TEM-150}}$	*bla*$_{\text{TEM-150}}$
		bla$_{\text{TEM-1A}}$	
	NODE_17length_1003_cov_118.203196	*aac(3)-IIa*	*aac(3)-IIe*
		aac(3)-IIa	
	NODE_36_length_51764_cov_28.272866	*bla*$_{\text{SHV-106}}$	*bla*$_{\text{SHV-212}}$
		bla$_{\text{SHV-28}}$	
	NODE_62_length_32497_cov_32.020791	*OqxA*	
		oqxA	*oqxA10*
		OqxB	
		oqxB	*oqxB17*
	NODE_84_length_14988_cov_40.597941	*fosA*	*fosA_gen*
ERR2796948	NODE_105_length_1605_cov_41.228687	*dfrA12*	*dfrA12*
	NODE_11length_1293_cov_18.656947	*aph(3')-Ia*	*aph(3')-Ia*
	NODE_1length_163728_cov_19.690803	*bla*$_{\text{SHV-182}}$	*bla*$_{\text{SHV-158}}$
	NODE_27_length_65120_cov_49.876833	*bla*$_{\text{KPC-3}}$	*bla*$_{\text{KPC-3}}$
	NODE_2length_94577_cov_26.165262	*OqxA*	
		oqxA	*oqxA*
		OqxB	
		oqxB	*oqxB*
	NODE_35_length_48918_cov_28.915886	*fosA*	*fosA6*
	NODE_38_length_42173_cov_29.607834	*catA1*	*catA1*
	NODE_63_length_13389_cov_48.861635	*aac(6')-Ib*	*aac(6')-Ib-AKT*
		aac(6')-Ib-cr	
	NODE_84_length_3484_cov_28.782544	*mph(A)*	*mph(A)*
	NODE_92_length_2234_cov_37.885619	*aadA2*	
		aadA2	
		qacE	
		sul1	*sul1*
ERR2796949	NODE_20_length_92255_cov_32.733566	*bla*$_{\text{KPC-3}}$	*bla*$_{\text{KPC-3}}$
	NODE_49_length_13177_cov_34.995479	*aac(6')-Ib*	*aac(6')-Ib-AKT*

		aac(6')-Ib-cr	
	NODE_59_length_5452_cov_31.857089	*aadA2b*	*aadA2*
		qacE	
		sul1	*sul1*
	NODE_6_length_270452_cov_34.487976	*fosA*	*fosA6*
	NODE_76_length_1498_cov_51.372721	*bla*$_{SHV-182}$	*bla*$_{SHV-158}$
	NODE_9_length_199615_cov_30.856993	*OqxA*	
		oqxA	*oqxA*
		OqxB	
		oqxB	*oqxB*
ERR2796950	NODE_12_length_175448_cov_27.261657	*bla*$_{SHV-106}$	*bla*$_{SHV-106}$
		bla$_{SHV-28}$	
	NODE_16_length_132895_cov_32.700538	*OqxA*	
		oqxA	*oqxA6*
		OqxB	
		oqxB	*oqxB20*
	NODE_39_length_8333_cov_41.930660	*tet(A)*	*tet(A)*
	NODE_50_length_2972_cov_42.727944	*bla*$_{CTX-M-15}$	*bla*$_{CTX-M-15}$
	NODE_53_length_2439_cov_29.055796	*aac(6')-Ib-cr*	*aac(6')-Ib-D181Y*
		aac(6')-Ib-cr	
		bla$_{OXA-1}$	*bla*$_{OXA-1}$
		catB3	
		catB3	
	NODE_6_length_26635cov_38.103086	*fosA*	*fosA6*
ERR2796952	NODE_109_length_1443_cov_44.915653	*bla*$_{OXA-9}$	*bla*$_{OXA-9}$
	NODE_119_length_1292_cov_22.624893	*aph(3')-Ia*	*aph(3')-Ia*
	NODE_130_length_1128_cov_90.042957	*aac(6')-Ib*	*aac(6')-Ib-AKT*
		aac(6')-Ib-cr	
	NODE_138_length_923_cov_45.228643	*bla*$_{TEM-1A}$	*bla*$_{TEM-1}$
	NODE_146_length_722_cov_75.339496	*bla*$_{SHV-12}$	*bla*$_{SHV-187}$
		bla$_{SHV-129}$	
		bla$_{SHV-13}$	
		bla$_{SHV-155}$	
		bla$_{SHV-172}$	
		bla$_{SHV-31}$	
	NODE_33_length_57932_cov_29.920837	*oqxA*	*oqxA*
		OqxA	
		oqxB	*oqxB*
		OqxB	
	NODE_4_length_270452_cov_34.471000	*fosA*	*fosA6*
	NODE_54_length_20602_cov_54.435507	*bla*$_{KPC-2}$	*bla*$_{KPC-2}$

	NODE_59_length_16866_cov_39.017504	*aadA2*	*aadA2*
		catA1	*catA1*
		dfrA12	*dfrA12*
		mph(A)	*mph(A)*
		qacE	
		sul1	*sul1*
ERR2796953	NODE_110_length_2234_cov_82.260085	*aadA2*	
		qacE	
		sul1	*sul1*
	NODE_119_length_1605_cov_65.955345	*dfrA12*	*dfrA12*
	NODE_125_length_1292_cov_25.578541	*aph(3')-Ia*	*aph(3')-Ia*
	NODE_2length_94476_cov_45.042300	*oqxA*	*oqxA*
		OqxA	
		oqxB	*oqxB*
		OqxB	
	NODE_30_length_65120_cov_78.826058	bla_{KPC-3}	bla_{KPC-3}
	NODE_40_length_42268_cov_55.466268	*catA1*	*catA1*
	NODE_5_length_204924_cov_53.162810	*fosA*	*fosA6*
	NODE_67_length_13389_cov_76.040567	*aac(6')-Ib*	*aac(6')-Ib-AKT*
		aac(6')-Ib-cr	
	NODE_95_length_3484_cov_69.512660	*mph(A)*	*mph(A)*
	NODE_96_length_3320_cov_133.743188	$bla_{SHV-182}$	$bla_{SHV-158}$
ERR2796955	NODE_14_length_158036_cov_14.611314	*OqxA*	
		oqxA	*oqxA*
		OqxB	
		oqxB	*oqxB*
	NODE_25_length_75609_cov_39.966933	*aadA2*	*aadA2*
		bla_{KPC-2}	bla_{KPC-2}
		catA1	*catA1*
		dfrA12	*dfrA12*
		mph(A)	*mph(A)*
		qacE	
		sul1	*sul1*
	NODE_33_length_50464_cov_16.892246	bla_{OXA-9}	bla_{OXA-9}
		bla_{TEM-1A}	bla_{TEM-1}
	NODE_34_length_42813_cov_30.553507	bla_{SHV-12}	bla_{SHV-12}
	NODE_8_length_212518_cov_16.462689	*fosA*	*fosA6*
	NODE_92_length_1292_cov_35.212876	*aph(3')-Ia*	*aph(3')-Ia*
ERR2796956	NODE_102_length_1605_cov_50.510825	*dfrA12*	*dfrA12*
	NODE_106_length_1498_cov_59.283005	$bla_{SHV-182}$	$bla_{SHV-158}$
	NODE_112_length_1292_cov_23.566524	*aph(3')-Ia*	*aph(3')-Ia*
	NODE_28_length_45002_cov_38.650763	bla_{KPC-3}	bla_{KPC-3}

	NODE_5_length_29573cov_41.074004	*OqxA*	
		oqxA	*oqxA*
		OqxB	
		oqxB	*oqxB*
	NODE_54_length_13070_cov_37.149579	*aac(6')-Ib*	*aac(6')-Ib-AKT*
		aac(6')-Ib-cr	
	NODE_6_length_262734_cov_46.160152	*fosA*	*fosA6*
	NODE_68_length_6205_cov_41.729187	*catA1*	*catA1*
	NODE_79_length_3484_cov_38.614239	*mph(A)*	*mph(A)*
	NODE_90_length_2234_cov_65.269103	*aadA2*	
		qacE	
		sul1	*sul1*
ERR2796957	NODE_107_length_1455_cov_20.337349	*bla*$_{SHV-12}$	
		bla$_{SHV-160}$	
		bla$_{SHV-66}$	
	NODE_108_length_1443_cov_15.099544	*bla*$_{OXA-9}$	*bla*$_{OXA-9}$
	NODE_114_length_1292_cov_11.529614	*aph(3')-Ia*	*aph(3')-Ia*
	NODE_128_length_923_cov_16.069095	*bla*$_{TEM-1A}$	*bla*$_{TEM-1}$
	NODE_12length_1128_cov_44.819181	*aac(6')-Ib*	*aac(6')-Ib-AKT*
		aac(6')-Ib-cr	
	NODE_37_length_3485cov_15.181661	*aadA2*	*aadA2*
		catA1	*catA1*
		dfrA12	*dfrA12*
		mph(A)	*mph(A)*
		qacE	
		sul1	*sul1*
	NODE_5_length_270440_cov_19.396651	*fosA*	*fosA6*
	NODE_56_length_20489_cov_17.641980	*bla*$_{KPC-2}$	*bla*$_{KPC-2}$
	NODE_length_35592cov_17.010641	*OqxA*	
		oqxA	*oqxA*
		OqxB	
		oqxB	*oqxB*
ERR2796958	NODE_16_length_10478cov_23.087345	*bla*$_{SHV-182}$	*bla*$_{SHV-158}$
	NODE_2_length_359638_cov_34.660531	*fosA*	*fosA6*
	NODE_25_length_74588_cov_30.624918	*oqxA*	*oqxA*
		OqxA	
		oqxB	*oqxB*
		OqxB	
	NODE_35_length_38498_cov_26.982747	*bla*$_{CTX-M-15}$	*bla*$_{CTX-M-15}$
	NODE_80_length_3376_cov_25.079717	*aac(6')-Ib-cr*	*aac(6')-Ib-D181Y*
		aac(6')-Ib-cr	

		aadA16	*aadA16*
		ARR-3	*arr-3*
		dfrA27	*dfrA27*
	NODE_86_length_2638_cov_10.031860	*mph(E)*	*mph(E)*
		msr(E)	*msr(E)*
	NODE_89_length_223cov_36.172180	*bla*$_{OXA-48}$	*bla*$_{OXA-48}$
	NODE_92_length_1758_cov_22.711220	*aadA5*	*aadA5*
		dfrA17	*dfrA17*
	NODE_99_length_1316_cov_44.520606	*qacE*	
		sul1	*sul1*
ERR2796961	NODE_16_length_118756_cov_22.859385	*fosA*	*fosA6*
	NODE_58_length_26184_cov_15.305254	*dfrA1*	*dfrA1*
		qacE	
		sul1	*sul1*
		tet(A)	*tet(A)*
	NODE_7_length_187749_cov_20.264415	*OqxA*	
		oqxA	*oqxA3*
		OqxB	
		oqxB	*oqxB12*
	NODE_72_length_7464_cov_25.597928	*bla*$_{OXA-181}$	*bla*$_{OXA-181}$
	NODE_76_length_4936_cov_13.675816	*aph(3'')-Ib*	*aph(3'')-Ib*
		aph(6)-Id	*aph(6)-Id*
		bla$_{TEM-1B}$	*bla*$_{TEM-1}$
		sul2	*sul2*
	NODE_78_length_4360_cov_25.195133	*bla*$_{CTX-M-14}$	*bla*$_{CTX-M-14}$
	NODE_8_length_166926_cov_15.077842	*bla*$_{SHV-33}$	*bla*$_{SHV-33}$
	NODE_80_length_406cov_18.807321	*aac(3)-IId*	*aac(3)-IId*
	NODE_92_length_1536_cov_13.053939	*bla*$_{LAP-2}$	*bla*$_{LAP-2}$
	NODE_98_length_1390_cov_28.038005	*qnrS1*	*qnrS1*
ERR2796963	NODE_108_length_1443_cov_45.464286	*bla*$_{OXA-9}$	*bla*$_{OXA-9}$
	NODE_113_length_1292_cov_20.971674	*aph(3')-Ia*	*aph(3')-Ia*
	NODE_12length_1128_cov_77.347652	*aac(6')-Ib*	*aac(6')-Ib-AKT*
		aac(6')-Ib-cr	
	NODE_138_length_722_cov_60.605042	*bla*$_{SHV-12}$	*bla*$_{SHV-187}$
		bla$_{SHV-129}$	
		bla$_{SHV-13}$	
		bla$_{SHV-155}$	
		bla$_{SHV-172}$	
		bla$_{SHV-31}$	
	NODE_32_length_59380_cov_31.322448	*aadA2*	*aadA2*
		catA1	*catA1*
		dfrA12	*dfrA12*

		mph(A)	*mph(A)*
		qacE	
		sul1	*sul1*
	NODE_37_length_35434_cov_48.131192	*bla*TEM-1A	*bla*TEM-1
	NODE_4_length_270452_cov_28.635627	*fosA*	*fosA6*
	NODE_5length_20517_cov_44.789063	*bla*KPC-2	*bla*KPC-2
	NODE_length_475446_cov_26.081789	*oqxA*	*oqxA*
		OqxA	
		oqxB	*oqxB*
		OqxB	
ERR2796964	NODE_105_length_1430_cov_76.234843	*dfrA8*	*dfrA8*
	NODE_10length_1545_cov_86.824401	*bla*CTX-M-8	*bla*CTX-M-8
	NODE_115_length_976_cov_271.731449	*bla*TEM-1A	*bla*TEM-150
	NODE_116_length_912_cov_104.206369	*sul1*	*sul1*
	NODE_135_length_59cov_234.301724	*aac(6')-Ib*	
		aac(6')-Ib-cr	
	NODE_2_length_437747_cov_42.249488	*OqxA*	
		oqxA	*oqxA11*
		OqxB	
		oqxB	*oqxB20*
	NODE_4_length_357724_cov_45.610248	*fosA*	*fosA_gen*
	NODE_50_length_17278_cov_56.192234	*qnrB19*	*qnrB19*
	NODE_62_length_8702_cov_76.702157	*dfrA21*	*dfrA21*
		dfrA21	*dfrA21*
		qacE	
	NODE_69_length_6918_cov_139.196878	*bla*KPC-2	*bla*KPC-2
	NODE_73_length_5077_cov_59.287677	*rmtD*	*rmtD1*
	NODE_78_length_3886_cov_64.490822	*tet(D)*	*tet(D)*
	NODE_82_length_3247_cov_92.452244	*qnrE1*	*qnrE1*
	NODE_86_length_2712_cov_316.416248	*aadA1*	*aadA1*
		*bla*OXA-9	*bla*OXA-9
	NODE_88_length_2644_cov_59.206992	*catA1*	*catA1*
	NODE_95_length_193cov_51.343126	*cat*	*catA13*
	NODE_33_length_3875cov_103.800150		*sat2_gen*
ERR2796969	NODE_12_length_159039_cov_35.602969	*fosA*	*fosA_gen*
	NODE_14_length_153849_cov_32.256469	*OqxA*	
		oqxA	*oqxA6*
		OqxB	
		oqxB	*oqxB19*
	NODE_3_length_452656_cov_24.183761	*bla*SHV-11	*bla*SHV-11
		*bla*SHV-67	
	NODE_36_length_3485_cov_104.894878	*mph(A)*	*mph(A)*

	NODE_3length_13908_cov_83.652420	*aph(3'')-Ib*	*aph(3'')-Ib*
		aph(3'')-Ib	
		aph(3'')-Ib	
		aph(3'')-Ib	
		aph(6)-Id	*aph(6)-Id*
	NODE_40_length_2592_cov_148.208925	*aadA2*	*aadA2*
		dfrA12	*dfrA12*
	NODE_45_length_1875_cov_124.698513	*aadA1*	*aadA1*
	NODE_46_length_1755_cov_115.555283	*aac(6')-Il*	*aac(6')-Il*
		bla$_{\text{VIM-1}}$	*bla*$_{\text{VIM-1}}$
	NODE_47_length_1538_cov_163.975195	*qacE*	
		sul1	*sul1*
	NODE_53_length_1292_cov_61.174249	*aph(3')-Ia*	*aph(3')-Ia*
	NODE_68_length_58cov_127.319383	*dfrA1*	*dfrA1*
		dfrA1	
		dfrA1	
ERR2796976	NODE_10_length_176282_cov_32.342534	*oqxA*	*oqxA5*
		OqxA	
		oqxB	*oqxB19*
		OqxB	
	NODE_19_length_69723_cov_34.789686	*aph(3'')-Ib*	*aph(3'')-Ib*
		aph(6)-Id	*aph(6)-Id*
		bla$_{\text{TEM-1B}}$	*bla*$_{\text{TEM-1}}$
		sul2	*sul2*
	NODE_29_length_13733_cov_39.342496	*qnrB1*	*qnrB1*
		tet(A)	*tet(A)*
	NODE_30_length_6980_cov_34.600321	*dfrA14*	*dfrA14*
	NODE_33_length_2437_cov_31.926407	*aac(6')-Ib-cr*	*aac(6')-Ib-D181Y*
		aac(6')-Ib-cr	
		bla$_{\text{OXA-1}}$	*bla*$_{\text{OXA-1}}$
		catB3	
		catB3	
	NODE_36_length_223cov_67.287532	*bla*$_{\text{OXA-48}}$	*bla*$_{\text{OXA-48}}$
	NODE_3length_5968_cov_29.085431	*bla*$_{\text{CTX-M-15}}$	*bla*$_{\text{CTX-M-15}}$
	NODE_5_length_378135_cov_37.086871	*fosA*	*fosA6*
	NODE_length_1017850_cov_29.944560	*bla*$_{\text{SHV-106}}$	*bla*$_{\text{SHV-106}}$
		bla$_{\text{SHV-28}}$	
ERR2796977	NODE_27_length_3446cov_36.424768	*bla*$_{\text{CTX-M-15}}$	*bla*$_{\text{CTX-M-15}}$
		OqxA	
		oqxA	*oqxA10*
	NODE_33_length_1563cov_23.107908	*sitABCD*	

	NODE_36_length_595cov_28.666724	*aac(6')-Ib-cr*	*aac(6')-Ib-G*
		aac(6')-Ib-Hangzhou	*catB*
		rmtF	*rmtF1*
	NODE_4_length_394863_cov_78.159504	$bla_{OXA-232}$	$bla_{OXA-232}$
		OqxB	
		oqxB	
	NODE_42_length_3635_cov_24.590365	*qacE*	
		sul1	*sul1*
	NODE_43_length_3485_cov_24.911554	*mph(A)*	*mph(A)*
	NODE_46_length_2857_cov_517.110256	bla_{TEM-1B}	bla_{TEM-1}
	NODE_5_length_35572cov_37.526072	*fosA*	*fosA_gen*
	NODE_54_length_2194_cov_25.981616	*aadA2*	*aadA2*
		dfrA12	*dfrA12*
	NODE_55_length_2018_cov_2.775899	*erm(B)*	*erm(B)*
	NODE_72_length_749_cov_34.033762	*ARR-2*	*arr-2*
	NODE_74_length_719_cov_22.273649	*catA1*	
	NODE_9_length_275974_cov_24.038837	$bla_{SHV-106}$	$bla_{SHV-212}$
		bla_{SHV-28}	
ERR2796978	NODE_100_length_3137_cov_3.943522	*erm(B)*	*erm(B)*
	NODE_113_length_1960_cov_9.555919	*aadA2*	*aadA2*
		dfrA12	*dfrA12*
	NODE_11length_217cov_14.320450	*aac(6')-Ib-cr*	*aac(6')-Ib-G*
		aac(6')-Ib-Hangzhou	
		rmtF	*rmtF1*
	NODE_130_length_1082_cov_21.741361	$bla_{CTX-M-15}$	$bla_{CTX-M-15}$
	NODE_133_length_983_cov_15.906542	*ARR-2*	*arr-2*
	NODE_143_length_720_cov_8.949410	*catA1*	
	NODE_33_length_64130_cov_6.580832	$bla_{SHV-106}$	$bla_{SHV-212}$
		bla_{SHV-28}	
	NODE_53_length_33318_cov_9.476334	*OqxA*	
		oqxA	*oqxA10*
	NODE_60_length_24877_cov_9.289818	*OqxB*	
		oqxB	
	NODE_66_length_1563cov_9.535733	*sitABCD*	
	NODE_82_length_6448_cov_458.164373	$bla_{OXA-232}$	$bla_{OXA-232}$
	NODE_96_length_3635_cov_10.542189	*qacE*	
		sul1	*sul1*
	NODE_97_length_3485_cov_9.326087	*mph(A)*	*mph(A)*
	NODE_99_length_329cov_93.936789	bla_{TEM-1B}	bla_{TEM-1}
	NODE_length_297082_cov_9.959122	*fosA*	*fosA_gen*
			catB

ERR2796981	NODE_13_length_157688_cov_29.065733	*OqxA*	
		oqxA	*oqxA10*
		OqxB	
		oqxB	*oqxB32*
	NODE_15_length_127095_cov_22.137665	*bla*$_{SHV-145}$	*bla*$_{SHV-145}$
		bla$_{SHV-179}$	
		bla$_{SHV-194}$	
		bla$_{SHV-199}$	
		bla$_{SHV-26}$	
		bla$_{SHV-78}$	
		bla$_{SHV-98}$	
	NODE_177_length_405_cov_60.151079	*ARR-3*	
	NODE_2_length_370753_cov_35.254100	*fosA5*	*fosA5*
	NODE_60_length_8425_cov_31.198482	*aac(6')-Ib-cr*	*aac(6')-Ib-AKT*
		aac(6')-Ib3	
		ARR-6	*arr-2*
		rmtF	*rmtF1*
	NODE_63_length_630cov_35.000000	*tet(A)*	*tet(A)*
	NODE_64_length_6263_cov_336.782595	*bla*$_{OXA-232}$	*bla*$_{OXA-232}$
	NODE_70_length_4433_cov_28.870413	*bla*$_{CTX-M-15}$	*bla*$_{CTX-M-15}$
	NODE_74_length_3826_cov_16.606650	*erm(B)*	*erm(B)*
	NODE_76_length_3493_cov_28.167855	*mph(A)*	*mph(A)*
	NODE_90_length_1756_cov_25.741559	*bla*$_{OXA-1}$	*bla*$_{OXA-1}$
		catB3	*catB*
		catB3	
	NODE_92_length_1534_cov_50.919687	*qacE*	
		sul1	*sul1*
	NODE_98_length_1337_cov_22.814876	*dfrA1*	*dfrA1*
ERR2796983	NODE_106_length_129cov_13.437285	*aph(3')-Ia*	*aph(3')-Ia*
	NODE_113_length_1104_cov_44.838280	*aadA1*	*aadA1*
		aadA1	
	NODE_11length_1134_cov_66.475670	*qacE*	
		sul1	*sul1*
	NODE_123_length_722_cov_50.870588	*bla*$_{SHV-12}$	*bla*$_{SHV-187}$
		bla$_{SHV-129}$	
		bla$_{SHV-13}$	
		bla$_{SHV-155}$	
		bla$_{SHV-172}$	
		bla$_{SHV-31}$	
	NODE_126_length_678_cov_40.221416	*aac(6')-Ib-cr*	
		aac(6')-Ib3	*aac(6')-Ib-cr5*
	NODE_54_length_2430cov_16.468230	*bla*$_{TEM-1B}$	*bla*$_{TEM-1}$

	NODE_59_length_14006_cov_17.067872	*bla*$_{\text{DHA-7}}$	*bla*$_{\text{DHA-7}}$
		qnrB4	*qnrB4*
	NODE_63_length_10565_cov_17.939165	*aph(3'')-Ib*	*aph(3'')-Ib*
		aph(3'')-Ib	
		aph(3'')-Ib	
		aph(3'')-Ib	
		aph(6)-Id	*aph(6)-Id*
	NODE_67_length_6562_cov_20.963792	*aac(6')-IIc*	*aac(6')-IIc*
			arr-269927220
			aac(3)-II
		ere(A)	*ere(A)*
	NODE_76_length_3484_cov_14.577599	*mph(A)*	*mph(A)*
	NODE_8_length_172964_cov_15.187402	*oqxA*	*oqxA*
		OqxA	
		oqxB	*oqxB*
		OqxB	
	NODE_83_length_2659_cov_14.874408	*mcr-9*	*mcr-9.1*
	NODE_8length_2689_cov_18.756831	*ARR-3*	*arr-3*
		bla$_{\text{OXA-1}}$	*bla*$_{\text{OXA-1}}$
		catB3	*catB3*
	NODE_97_length_165cov_14.858924	*catA2*	*catA2*
	NODE_9length_1755_cov_19.386364	*dfrA19*	*dfrA19*
	NODE_length_350053_cov_17.857607	*fosA*	*fosA6*
ERR2796984	NODE_105_length_5839_cov_11.142682	*aac(3)-IId*	*aac(3)-IId*
	NODE_106_length_5618_cov_13.373520	*dfrA1*	*dfrA1*
		tet(A)	*tet(A)*
	NODE_115_length_4378_cov_11.941426	*bla*$_{\text{DHA-1}}$	
		bla$_{\text{DHA-24}}$	
		bla$_{\text{DHA-7}}$	*ble-*$_{\text{MBL}}$
		bla$_{\text{NDM-1}}$	*bla*$_{\text{NDM-1}}$
	NODE_123_length_3485_cov_12.260572	*mph(A)*	*mph(A)*
	NODE_132_length_2439_cov_13.068772	*aac(6')-Ib-cr*	*aac(6')-Ib-D181Y*
		aac(6')-Ib-cr	
		bla$_{\text{OXA-1}}$	*bla*$_{\text{OXA-1}}$
		catB3	
		catB3	
	NODE_138_length_200cov_13.334045	*bla*$_{\text{CTX-M-15}}$	*bla*$_{\text{CTX-M-15}}$
	NODE_144_length_1759_cov_12.955882	*aadA2*	*aadA2*
	NODE_155_length_129cov_7.455326	*aph(3')-Ia*	*aph(3')-Ia*
	NODE_156_length_1203_cov_15.455390	*catA1*	*catA1*
	NODE_160_length_1134_cov_29.784508	*qacE*	

		sul1	*sul1*
	NODE_37_length_45383_cov_16.958547	*OqxA*	
		oqxA	*oqxA*
		OqxB	
		oqxB	*oqxB*
	NODE_4_length_218206_cov_16.634642	*fosA*	*fosA_gen*
	NODE_89_length_10782_cov_7.981323	*armA*	*armA*
		mph(E)	*mph(E)*
		msr(E)	*msr(E)*
	NODE_9_length_132247_cov_11.357077	*bla*$_{SHV-182}$	*bla*$_{SHV-158}$
	NODE_9length_9907_cov_16.251125	*bla*$_{TEM-1B}$	*bla*$_{TEM-1}$
		qnrS1	*qnrS1*
ERR2796988	NODE_100_length_1186_cov_112.336166	*dfrA14*	*dfrA14*
	NODE_125_length_630_cov_236.594433	*aac(6')-Ib*	
		aac(6')-Ib-cr	
	NODE_13_length_151290_cov_48.643114	*bla*$_{SHV-12}$	*bla*$_{SHV-12}$
	NODE_130_length_547_cov_118.940476	*bla*$_{TEM-150}$	
		bla$_{TEM-171}$	
		bla$_{TEM-1A}$	
		bla$_{TEM-1C}$	
		bla$_{TEM-40}$	
		bla$_{TEM-97}$	
		bla$_{TEM-98}$	
	NODE_28_length_36274_cov_117.417241	*bla*$_{KPC-3}$	*bla*$_{KPC-3}$
	NODE_30_length_34055_cov_60.207262	*catA1*	*catA1*
	NODE_6_length_270452_cov_65.859340	*fosA*	*fosA6*
	NODE_63_length_558cov_62.658783	*aadA2*	*aadA2*
		dfrA12	*dfrA12*
		qacE	
		sul1	*sul1*
	NODE_73_length_3484_cov_68.105749	*mph(A)*	*mph(A)*
	NODE_7length_3764_cov_99.895243	*aph(3'')-Ib*	*aph(3'')-Ib*
		aph(6)-Id	*aph(6)-Id*
		sul2	*sul2*
	NODE_85_length_1907_cov_105.132584	*aadA1*	*aadA1*
		bla$_{OXA-9}$	*bla*$_{OXA-9}$
	NODE_97_length_1292_cov_37.415451	*aph(3')-Ia*	*aph(3')-Ia*
	NODE_length_840292_cov_62.040597	*oqxA*	*oqxA*
		OqxA	
		oqxB	*oqxB*
		OqxB	
	NODE_100_length_249cov_12.780457	*qacE*	

ERR2796989		*sul1*	*sul1*
	NODE_114_length_1698_cov_21.595799	*bla*$_{SHV-12}$	
		bla$_{SHV-160}$	
		bla$_{SHV-66}$	
	NODE_117_length_1443_cov_25.326748	*bla*$_{OXA-9}$	*bla*$_{OXA-9}$
	NODE_122_length_1292_cov_22.054077	*aph(3')-Ia*	*aph(3')-Ia*
	NODE_138_length_923_cov_29.285176	*bla*$_{TEM-1A}$	*bla*$_{TEM-1}$
	NODE_140_length_899_cov_56.603627	*aac(6')-Ib*	*aac(6')-Ib-AKT*
		aac(6')-Ib-cr	
	NODE_14length_859_cov_30.592896	*aadA2b*	*aadA2*
	NODE_43_length_29075_cov_26.490846	*bla*$_{KPC-2}$	*bla*$_{KPC-2}$
	NODE_5_length_270412_cov_18.932960	*fosA*	*fosA6*
	NODE_6_length_259907_cov_17.487690	*oqxA*	*oqxA*
		OqxA	
		oqxB	*oqxB*
		OqxB	
	NODE_74_length_7095_cov_13.068025	*aadA1*	*aadA1*
		cmlA1	*cmlA1*
		sul3	*sul3*
	NODE_82_length_5320_cov_13.933950	*aac(3)-IV*	*aac(3)-IVa*
		aph(4)-Ia	*aph(4)-Ia*
	NODE_93_length_3486_cov_24.373325	*mph(A)*	*mph(A)*
ERR2796990	NODE_103_length_3440_cov_16.846966	*bla*$_{SCO-1}$	*bla*$_{SCO-1}$
	NODE_104_length_2972_cov_46.532865	*bla*$_{CTX-M-15}$	*bla*$_{CTX-M-15}$
	NODE_10length_376cov_62.570996	*tet(D)*	*tet(D)*
	NODE_112_length_2437_cov_40.505628	*aac(6')-Ib-cr*	*aac(6')-Ib-D181Y*
		aac(6')-Ib-cr	
		bla$_{OXA-1}$	*bla*$_{OXA-1}$
		catB3	
		catB3	
	NODE_114_length_2412_cov_43.849453	*bla*$_{TEM-1A}$	*bla*$_{TEM-150}$
	NODE_118_length_223cov_34.438931	*bla*$_{OXA-48}$	*bla*$_{OXA-48}$
	NODE_144_length_1003_cov_110.102740	*aac(3)-IIa*	*aac(3)-IIe*
		aac(3)-IIa	
	NODE_18_length_113234_cov_21.689719	*bla*$_{SHV-106}$	*bla*$_{SHV-212}$
		bla$_{SHV-28}$	
	NODE_46_length_32685_cov_27.390257	*OqxA*	
		oqxA	*oqxA10*
		OqxB	
		oqxB	*oqxB17*
	NODE_47_length_30804_cov_33.628321	*fosA*	*fosA_gen*

	NODE_80_length_7732_cov_54.575279	*dfrA14*	*dfrA14*
ERR2796992	NODE_10_length_199158_cov_35.232522	*oqxA*	*oqxA*
		OqxA	
		oqxB	*oqxB*
		OqxB	
	NODE_103_length_1605_cov_52.941137	*dfrA12*	*dfrA12*
	NODE_107_length_1444_cov_45.026576	*bla*$_{OXA-9}$	*bla*$_{OXA-9}$
	NODE_113_length_1292_cov_23.688412	*aph(3')-Ia*	*aph(3')-Ia*
	NODE_120_length_1128_cov_117.364635	*aac(6')-Ib*	*aac(6')-Ib-AKT*
		aac(6')-Ib-cr	
	NODE_128_length_923_cov_46.557789	*bla*$_{TEM-1A}$	*bla*$_{TEM-1}$
	NODE_22_length_82722_cov_27.270706	*bla*$_{SHV-182}$	*bla*$_{SHV-158}$
	NODE_36_length_40804_cov_41.077489	*catA1*	*catA1*
	NODE_37_length_39222_cov_55.640414	*bla*$_{KPC-3}$	*bla*$_{KPC-3}$
	NODE_4_length_270452_cov_40.089122	*fosA*	*fosA6*
	NODE_8length_3484_cov_49.208817	*mph(A)*	*mph(A)*
	NODE_92_length_2234_cov_62.048885	*aadA2*	
		qacE	
		sul1	*sul1*
ERR2796993	NODE_115_length_722_cov_202.364706	*bla*$_{SHV-12}$	*bla*$_{SHV-187}$
		bla$_{SHV-129}$	
		bla$_{SHV-13}$	
		bla$_{SHV-155}$	
		bla$_{SHV-172}$	
		bla$_{SHV-31}$	
	NODE_30_length_59380_cov_175.676472	*aadA2*	*aadA2*
		catA1	*catA1*
		dfrA12	*dfrA12*
		mph(A)	*mph(A)*
		qacE	
		sul1	*sul1*
	NODE_36_length_37143_cov_120.718419	*bla*$_{OXA-9}$	*bla*$_{OXA-9}$
		bla$_{TEM-1A}$	*bla*$_{TEM-1}$
	NODE_47_length_20517_cov_109.997106	*bla*$_{KPC-2}$	*bla*$_{KPC-2}$
	NODE_5_length_270452_cov_70.584526	*fosA*	*fosA6*
	NODE_55_length_12954_cov_135.707648	*aac(6')-Ib*	*aac(6')-Ib-AKT*
		aac(6')-Ib-cr	
	NODE_99_length_1292_cov_113.147639	*aph(3')-Ia*	*aph(3')-Ia*
	NODE_length_485320_cov_63.191703	*OqxA*	
		oqxA	*oqxA*
		OqxB	
		oqxB	*oqxB*

ERR2796994	NODE_14_length_137096_cov_33.260183	*oqxA*	*oqxA6*
		OqxA	
		oqxB	*oqxB20*
		OqxB	
	NODE_3length_53224_cov_43.075371	*sul1*	*sul1*
		sul1	
	NODE_47_length_11357_cov_37.048798	*bla*$_{TEM-1B}$	*bla*$_{TEM-1}$
	NODE_48_length_1120cov_36.539462		*ble*-$_{MBL}$
		bla$_{NDM-1}$	*bla*$_{NDM-1}$
		rmtC	*rmtC*
	NODE_52_length_8260_cov_35.736997	*dfrA14*	*dfrA14*
	NODE_63_length_5533_cov_37.651868	*qnrB1*	*qnrB1*
	NODE_67_length_4958_cov_65.159387	*aadA1*	*aadA1*
		aadA1	
		ARR-2	*arr-2*
		ere(A)	*ere(A)*
		qacE	
	NODE_79_length_2436_cov_28.013426	*aac(6')-Ib-cr*	*aac(6')-Ib-D181Y*
		aac(6')-Ib-cr	
		bla$_{OXA-1}$	*bla*$_{OXA-1}$
		catB3	
		catB3	
	NODE_89_length_1569_cov_23.958391	*bla*$_{CTX-M-15}$	*bla*$_{CTX-M-15}$
	NODE_8length_2245_cov_41.220491	*aac(3)-IIa*	*aac(3)-IIe*
	NODE_9_length_175433_cov_23.163714	*bla*$_{SHV-106}$	*bla*$_{SHV-106}$
		bla$_{SHV-28}$	
	NODE_length_362182_cov_38.736201	*fosA*	*fosA6*
ERR2796995	NODE_127_length_7899_cov_16.124292	*tet(A)*	*tet(A)*
	NODE_133_length_7438_cov_9.230885	*mph(A)*	*mph(A)*
		sul1	*sul1*
	NODE_15length_4368_cov_16.298515	*bla*$_{CTX-M-15}$	*bla*$_{CTX-M-15}$
	NODE_180_length_1483_cov_7.883481	*bla*$_{OXA-1}$	*bla*$_{OXA-1}$
		catB3	
		catB3	
	NODE_203_length_906_cov_21.632863	*aac(6')-Ib-cr*	*aac(6')-Ib-D181Y*
		aac(6')-Ib-cr	
	NODE_28_length_55718_cov_9.373532	*bla*$_{SHV-182}$	*bla*$_{SHV-158}$
	NODE_44_length_38052_cov_14.231615	*bla*$_{KPC-2}$	*bla*$_{KPC-2}$
	NODE_79_length_20836_cov_12.383746	*OqxA*	
		oqxA	*oqxA*
		OqxB	

		oqxB	*oqxB*
	NODE_82_length_19725_cov_15.117410	*dfrA30*	*dfrA30*
		qacE	
	NODE_length_270776_cov_14.733322	*fosA*	*fosA6*
ERR2796996	NODE_112_length_655cov_16.085461	*mph(E)*	*mph(E)*
		msr(E)	*msr(E)*
	NODE_119_length_5120_cov_42.483277	*dfrA30*	*dfrA30*
		qacE	
	NODE_13length_3484_cov_46.880548	*mph(A)*	*mph(A)*
	NODE_14length_2440_cov_16.079550	*aac(6')-Ib-cr*	*aac(6')-Ib-D181Y*
		aac(6')-Ib-cr	
		bla$_{\text{OXA-1}}$	*bla*$_{\text{OXA-1}}$
		catB3	
		catB3	
	NODE_160_length_1465_cov_39.099402	*bla*$_{\text{NDM-1}}$	*bla*$_{\text{NDM-1}}$
	NODE_168_length_1179_cov_20.166350	*aadA2b*	*aadA2*
	NODE_173_length_987_cov_81.373256	*sul1*	*sul1*
	NODE_2_length_201372_cov_27.621730	*fosA*	*fosA6*
	NODE_36_length_51830_cov_19.887569	*bla*$_{\text{SHV-182}}$	*bla*$_{\text{SHV-158}}$
	NODE_55_length_31209_cov_20.711602	*oqxA*	*oqxA*
		OqxA	
		oqxB	*oqxB*
		OqxB	
	NODE_5length_36086_cov_26.636113	*bla*$_{\text{CTX-M-15}}$	*bla*$_{\text{CTX-M-15}}$
ERR2796998	NODE_18_length_109935_cov_48.220931	*bla*$_{\text{KPC-2}}$	*bla*$_{\text{KPC-2}}$
	NODE_30_length_46729_cov_33.396743	*oqxA*	*oqxA*
		OqxA	
		oqxB	*oqxB*
		OqxB	
	NODE_45_length_12550_cov_53.730822	*bla*$_{\text{TEM-1A}}$	*bla*$_{\text{TEM-1}}$
	NODE_78_length_1443_cov_48.567629	*bla*$_{\text{OXA-9}}$	*bla*$_{\text{OXA-9}}$
	NODE_84_length_1128_cov_76.231768	*aac(6')-Ib*	*aac(6')-Ib-AKT*
		aac(6')-Ib-cr	
	NODE_9_length_212659_cov_34.139819	*fosA*	*fosA6*
	NODE_95_length_722_cov_72.176471	*bla*$_{\text{SHV-12}}$	*bla*$_{\text{SHV-187}}$
		bla$_{\text{SHV-129}}$	
		bla$_{\text{SHV-13}}$	
		bla$_{\text{SHV-155}}$	
		bla$_{\text{SHV-172}}$	
		bla$_{\text{SHV-31}}$	
	NODE_13_length_15769cov_30.170648	*bla*$_{\text{SHV-182}}$	*bla*$_{\text{SHV-158}}$

ERR2797002	NODE_23_length_82106_cov_76.301431	*bla*$_{KPC-3}$	*bla*$_{KPC-3}$
	NODE_37_length_33756_cov_42.597104	*fosA*	*fosA6*
	NODE_55_length_13070_cov_66.378583	*aac(6')-Ib*	*aac(6')-Ib-AKT*
		aac(6')-Ib-cr	
	NODE_66_length_5344_cov_72.059804	*aadA2b*	*aadA2*
		qacE	
		sul1	*sul1*
	NODE_8_length_190909_cov_38.657227	*oqxA*	*oqxA*
		OqxA	
		oqxB	*oqxB*
		OqxB	
ERR2797003	NODE_112_length_376cov_27.550358	*tet(D)*	*tet(D)*
	NODE_114_length_3534_cov_16.117112	*bla*$_{SCO-1}$	*bla*$_{SCO-1}$
	NODE_118_length_2972_cov_28.416520	*bla*$_{CTX-M-15}$	*bla*$_{CTX-M-15}$
	NODE_125_length_2415_cov_22.481206	*bla*$_{TEM-1A}$	*bla*$_{TEM-150}$
	NODE_135_length_223cov_51.985581	*bla*$_{OXA-48}$	*bla*$_{OXA-48}$
	NODE_142_length_1660_cov_10.104371	*bla*$_{OXA-1}$	*bla*$_{OXA-1}$
		catB3	
		catB3	
	NODE_162_length_1039_cov_32.699561	*aac(6')-Ib-cr*	*aac(6')-Ib-D181Y*
		aac(6')-Ib-cr	
	NODE_167_length_1003_cov_61.041096	*aac(3)-IIa*	*aac(3)-IIe*
		aac(3)-IIa	
	NODE_33_length_61068_cov_37.356443	*fosA*	*fosA_gen*
	NODE_48_length_39138_cov_24.689575	*bla*$_{SHV-106}$	*bla*$_{SHV-212}$
		bla$_{SHV-28}$	
	NODE_55_length_32600_cov_31.248268	*OqxA*	
		oqxA	*oqxA10*
		OqxB	
		oqxB	*oqxB17*
	NODE_9length_7769_cov_25.520414	*dfrA14*	*dfrA14*
ERR2797004	NODE_105_length_3484_cov_30.333035	*mph(A)*	*mph(A)*
	NODE_114_length_2234_cov_43.866635	*aadA2*	
		qacE	
		sul1	*sul1*
	NODE_125_length_1605_cov_36.231394	*dfrA12*	*dfrA12*
	NODE_129_length_1444_cov_19.927107	*bla*$_{OXA-9}$	*bla*$_{OXA-9}$
	NODE_15length_1128_cov_59.452547	*aac(6')-Ib*	*aac(6')-Ib-AKT*
		aac(6')-Ib-cr	
	NODE_34_length_57872_cov_24.322937	*oqxA*	*oqxA*
		OqxA	

		oqxB	*oqxB*
		OqxB	
	NODE_38_length_51576_cov_19.374215	*bla*$_{\text{SHV-182}}$	*bla*$_{\text{SHV-158}}$
	NODE_47_length_3385cov_25.855652	*fosA*	*fosA6*
	NODE_48_length_32348_cov_27.903789	*bla*$_{\text{KPC-3}}$	*bla*$_{\text{KPC-3}}$
	NODE_76_length_13417_cov_42.587509	*bla*$_{\text{TEM-1A}}$	*bla*$_{\text{TEM-1}}$
	NODE_92_length_5999_cov_31.166213	*catA1*	*catA1*
ERR2797005	NODE_103_length_1292_cov_41.181116	*aph(3')-Ia*	*aph(3')-Ia*
	NODE_107_length_1128_cov_87.298701	*aac(6')-Ib*	*aac(6')-Ib-AKT*
		aac(6')-Ib-cr	
	NODE_120_length_923_cov_45.086683	*bla*$_{\text{TEM-1A}}$	*bla*$_{\text{TEM-1}}$
	NODE_16_length_122237_cov_26.406216	*fosA*	*fosA6*
	NODE_2length_86708_cov_49.438595	*catA1*	*catA1*
	NODE_47_length_21824_cov_48.807669	*bla*$_{\text{KPC-3}}$	*bla*$_{\text{KPC-3}}$
	NODE_5_length_295697_cov_24.269327	*oqxA*	*oqxA*
		OqxA	
		oqxB	*oqxB*
		OqxB	
	NODE_86_length_2376_cov_59.795020	*bla*$_{\text{SHV-182}}$	*bla*$_{\text{SHV-158}}$
	NODE_87_length_2234_cov_62.468913	*aadA2*	
		qacE	
		sul1	*sul1*
	NODE_8length_3484_cov_49.628239	*mph(A)*	*mph(A)*
	NODE_97_length_1443_cov_49.449088	*bla*$_{\text{OXA-9}}$	*bla*$_{\text{OXA-9}}$
	NODE_99_length_1370_cov_56.218021	*dfrA12*	*dfrA12*
ERR2797006	NODE_103_length_1292_cov_26.397425	*aph(3')-Ia*	*aph(3')-Ia*
	NODE_25_length_70622_cov_47.512646	*catA1*	*catA1*
	NODE_27_length_62989_cov_64.852184	*bla*$_{\text{KPC-3}}$	*bla*$_{\text{KPC-3}}$
	NODE_5_length_270452_cov_44.672228	*fosA*	*fosA6*
	NODE_59_length_13389_cov_66.019756	*aac(6')-Ib*	*aac(6')-Ib-AKT*
		aac(6')-Ib-cr	
	NODE_7_length_232975_cov_40.169252	*oqxA*	*oqxA*
		OqxA	
		oqxB	*oqxB*
		OqxB	
	NODE_80_length_3484_cov_57.904975	*mph(A)*	*mph(A)*
	NODE_86_length_2234_cov_71.471761	*aadA2*	
		qacE	
		sul1	*sul1*
	NODE_8length_3320_cov_102.737551	*bla*$_{\text{SHV-182}}$	*bla*$_{\text{SHV-158}}$
	NODE_99_length_1370_cov_56.256637	*dfrA12*	*dfrA12*
	NODE_23_length_82106_cov_33.277254	*bla*$_{\text{KPC-3}}$	*bla*$_{\text{KPC-3}}$

ERR2797007	NODE_34_length_51716_cov_11.884995	*bla*$_{SHV-182}$	*bla*$_{SHV-158}$
	NODE_43_length_33840_cov_19.282621	*fosA*	*fosA6*
	NODE_60_length_19618_cov_16.040942	*oqxA*	*oqxA*
		OqxA	
		oqxB	*oqxB*
		OqxB	
	NODE_73_length_13070_cov_33.847253	*aac(6')-Ib*	*aac(6')-Ib-AKT*
		aac(6')-Ib-cr	
	NODE_95_length_5344_cov_16.166187	*aadA2b*	*aadA2*
		qacE	
		sul1	*sul1*
ERR2797008	NODE_115_length_1605_cov_56.783491	*dfrA12*	*dfrA12*
	NODE_36_length_52776_cov_22.248096	*bla*$_{SHV-182}$	*bla*$_{SHV-158}$
	NODE_4_length_27041cov_36.345992	*fosA*	*fosA6*
	NODE_5length_34014_cov_42.319090	*catA1*	*catA1*
	NODE_65_length_19275_cov_49.606852	*aadA2*	
		bla$_{DHA-1}$	*bla*$_{DHA-1}$
		qacE	
		qacE	
		qnrB4	*qnrB4*
		sul1	*sul1*
		sul1	*sul1*
	NODE_75_length_10519_cov_82.239222	*aac(6')-Ib*	*aac(6')-Ib-AKT*
		aac(6')-Ib-cr	
		aadA1	*aadA1*
		bla$_{OXA-9}$	*bla*$_{OXA-9}$
		bla$_{TEM-1A}$	*bla*$_{TEM-150}$
	NODE_98_length_2972_cov_211.280141	*bla*$_{CTX-M-15}$	*bla*$_{CTX-M-15}$
	NODE_99_length_285cov_56.020191	*aac(3)-IIa*	*aac(3)-IIe*
	NODE_length_424546_cov_32.532278	*oqxA*	*oqxA*
		OqxA	
		oqxB	*oqxB*
		OqxB	
ERR2797009	NODE_27_length_65120_cov_48.467358	*bla*$_{KPC-3}$	*bla*$_{KPC-3}$
	NODE_5_length_295696_cov_48.453180	*oqxA*	*oqxA*
		OqxA	
		oqxB	*oqxB*
		OqxB	
	NODE_5length_13177_cov_68.843065	*aac(6')-Ib*	*aac(6')-Ib-AKT*
		aac(6')-Ib-cr	
	NODE_6_length_270453_cov_54.063065	*fosA*	*fosA6*
	NODE_64_length_6205_cov_50.065482	*catA1*	*catA1*

	NODE_72_length_3484_cov_48.191540	*mph(A)*	*mph(A)*
	NODE_78_length_2234_cov_73.923588	*aadA2*	
		qacE	
		sul1	*sul1*
	NODE_88_length_1605_cov_53.525034	*dfrA12*	*dfrA12*
	NODE_89_length_1498_cov_85.134209	*bla*$_{SHV-182}$	*bla*$_{SHV-158}$
	NODE_94_length_1292_cov_24.836910	*aph(3')-Ia*	*aph(3')-Ia*
ERR2797010	NODE_102_length_5838_cov_46.340046	*qnrB1*	*qnrB1*
	NODE_109_length_4572_cov_32.286839	*aadA1*	*aadA1*
		aadA1	
		ARR-2	*arr-2*
		bla$_{OXA-10}$	*bla*$_{OXA-10}$
		cmlA1	*cmlA5*
		qacE	
	NODE_112_length_4399_cov_33.943586	*bla*$_{CTX-M-15}$	*bla*$_{CTX-M-15}$
	NODE_119_length_3780_cov_36.638379	*tet(D)*	*tet(D)*
	NODE_125_length_285cov_48.983480	*aac(3)-IIa*	*aac(3)-IIe*
	NODE_132_length_2436_cov_22.450411	*aac(6')-Ib-cr*	*aac(6')-Ib-D181Y*
		aac(6')-Ib-cr	
		bla$_{OXA-1}$	*bla*$_{OXA-1}$
		catB3	
		catB3	
	NODE_134_length_2327_cov_76.972273	*aph(3'')-Ib*	*aph(3'')-Ib*
		aph(6)-Id	*aph(6)-Id*
		sul2	
		sul2	
		sul2	
	NODE_142_length_1758_cov_31.192520	*bla*$_{NDM-1}$	*bla*$_{NDM-1}$
	NODE_149_length_1398_cov_14.494099	*aph(3')-Ia*	*aph(3')-Ia*
	NODE_152_length_1377_cov_52.348000	*bla*$_{SHV-1}$	
		bla$_{SHV-49}$	
	NODE_155_length_1212_cov_16.537327	*bla*$_{TEM-1B}$	*bla*$_{TEM-1}$
	NODE_162_length_987_cov_98.100000	*sul1*	*sul1*
	NODE_173_length_797_cov_72.470149	*dfrA14*	*dfrA14*
	NODE_38_length_47749_cov_44.653353	*bla*$_{CMY-16}$	*bla*$_{CMY-16}$
		bla$_{CMY-4}$	
	NODE_39_length_46666_cov_34.444982	*oqxA*	*oqxA11*
		OqxA	
		oqxB	*oqxB20*
		OqxB	
	NODE_45_length_42623_cov_34.252753	*fosA*	*fosA_gen*

	NODE_89_length_8967_cov_46.030882	*floR*	*floR*
		tet(A)	*tet(A)*
ERR2797011	NODE_102_length_78cov_58.475535	*bla*$_{\text{TEM-141}}$	
		bla$_{\text{TEM-1B}}$	
		bla$_{\text{TEM-206}}$	
		bla$_{\text{TEM-209}}$	
		bla$_{\text{TEM-210}}$	
		bla$_{\text{TEM-214}}$	
		bla$_{\text{TEM-216}}$	
		bla$_{\text{TEM-33}}$	
		bla$_{\text{TEM-34}}$	
	NODE_14_length_130175_cov_27.229832	*oqxA*	*oqxA5*
		OqxA	
		oqxB	*oqxB20*
		OqxB	
	NODE_3_length_373415_cov_30.805397	*fosA*	*fosA_gen*
	NODE_47_length_14493_cov_43.574760	*bla*$_{\text{KPC-2}}$	*bla*$_{\text{KPC-2}}$
	NODE_52_length_7358_cov_38.258470	*tet(A)*	*tet(A)*
	NODE_54_length_704cov_34.743708	*dfrA14*	*dfrA14*
	NODE_58_length_4093_cov_34.709279	*qnrB1*	*qnrB1*
	NODE_62_length_2863_cov_21.391447	*bla*$_{\text{CTX-M-15}}$	*bla*$_{\text{CTX-M-15}}$
	NODE_64_length_2853_cov_35.987161	*aac(3)-IIa*	*aac(3)-IIe*
	NODE_68_length_2436_cov_25.911217	*aac(6')-Ib-cr*	*aac(6')-Ib-D181Y*
		aac(6')-Ib-cr	
		bla$_{\text{OXA-1}}$	*bla*$_{\text{OXA-1}}$
		catB3	
		catB3	
	NODE_6length_3764_cov_28.554853	*aph(3'')-Ib*	*aph(3'')-Ib*
		aph(6)-Id	*aph(6)-Id*
		sul2	*sul2*
	NODE_8length_1576_cov_39.048309	*bla*$_{\text{OXA-9}}$	*bla*$_{\text{OXA-9}}$
	NODE_9_length_180685_cov_20.848891	*bla*$_{\text{SHV-38}}$	*bla*$_{\text{SHV-187}}$
SRR3465532	NODE_3_length_729794_cov_147.128164	*OqxA*	*oqxA6*
		oqxA	*oqxB12*
		OqxB	
		oqxB	
	NODE_6_length_360870_cov_146.606803	*fosA*	*fosA_gen*
	NODE_length_205266cov_128.937272	*bla*$_{\text{SHV-108}}$	*bla*$_{\text{SHV-108}}$
SRR4115668	NODE_20_length_59615_cov_25.781973	*aadA1*	*aadA1*
		aadA1	*aadA1*
		aadA1	

		aadA1	
		armA	*armA*
		ARR-2	*arr-2*
		bla$_{\text{NDM-1}}$	*bla*$_{\text{NDM-1}}$
		bla$_{\text{OXA-10}}$	*bla*$_{\text{OXA-10}}$
		bla$_{\text{OXA-16}}$	*bla*$_{\text{OXA-10}}$
		cmlA1	*cmlA5*
		mph(E)	*mph(E)*
		msr(E)	*msr(E)*
		qacE	
		qacE	*ble-*$_{\text{MBL}}$
		sul1	*sul1*
		sul1	*sul1*
	NODE_22_length_48065_cov_26.163524	*bla*$_{\text{CMY-16}}$	*bla*$_{\text{CMY-16}}$
		bla$_{\text{CMY-4}}$	
	NODE_3_length_382495_cov_16.793623	*OqxA*	
		oqxA	*oqxA6*
		OqxB	
		oqxB	*oqxB20*
	NODE_39_length_13732_cov_19.590445	*qnrB1*	*qnrB1*
		tet(A)	*tet(A)*
	NODE_4_length_350976_cov_18.243087	*fosA*	*fosA6*
	NODE_42_length_6980_cov_21.922953	*dfrA14*	*dfrA14*
	NODE_44_length_5886_cov_18.620073	*aph(3'')-Ib*	*aph(3'')-Ib*
		aph(6)-Id	*aph(6)-Id*
		bla$_{\text{TEM-1B}}$	*bla*$_{\text{TEM-1}}$
		sul2	*sul2*
	NODE_52_length_3813_cov_20.622084	*bla*$_{\text{CTX-M-15}}$	*bla*$_{\text{CTX-M-15}}$
	NODE_55_length_2853_cov_20.083272	*aac(3)-IIa*	*aac(3)-IIe*
	NODE_57_length_2439_cov_24.948962	*aac(6')-Ib-cr*	*aac(6')-Ib-D181Y*
		aac(6')-Ib-cr	
		bla$_{\text{OXA-1}}$	*bla*$_{\text{OXA-1}}$
		catB3	
		catB3	
	NODE_9_length_252050_cov_11.904062	*bla*$_{\text{SHV-106}}$	*bla*$_{\text{SHV-106}}$
		bla$_{\text{SHV-28}}$	
SRR5132378	NODE_12_length_183472_cov_11.592670	*bla*$_{\text{SHV-182}}$	*bla*$_{\text{SHV-158}}$
	NODE_3_length_355016_cov_14.983719	*OqxA*	
		oqxA	*oqxA*
		OqxB	
		oqxB	*oqxB*

	NODE_37_length_23953_cov_19.272601	*aph(3')-VI*	*aph(3')-VI*
		bla$_{\text{NDM-1}}$	*bla*$_{\text{NDM-1}}$
			ble-$_{\text{MBL}}$
	NODE_44_length_10823_cov_16.455310	*armA*	*armA*
		mph(E)	
		mph(E)	
		msr(E)	*msr(E)*
	NODE_45_length_9882_cov_10.493080	*bla*$_{\text{TEM-1B}}$	*bla*$_{\text{TEM-1}}$
		qnrS1	*qnrS1*
	NODE_49_length_5434_cov_8.317882	*dfrA1*	*dfrA1*
		tet(A)	*tet(A)*
	NODE_50_length_5139_cov_18.030527	*aadA1*	*aadA1*
		aadA1	
		ARR-2	*arr-2*
		bla$_{\text{OXA-10}}$	*bla*$_{\text{OXA-10}}$
		cmlA1	*cmlA5*
	NODE_52_length_4053_cov_12.896077	*catA1*	*catA1*
	NODE_54_length_3885_cov_9.362693	*aph(3'')-Ib*	
		aph(3'')-Ib	
		aph(3'')-Ib	
		aph(3'')-Ib	*aph(3'')-Ib*
		aph(6)-Id	*aph(6)-Id*
	NODE_59_length_2854_cov_9.192519	*aac(3)-IIa*	*aac(3)-IIe*
	NODE_62_length_2335_cov_10.357790	*bla*$_{\text{CTX-M-15}}$	*bla*$_{\text{CTX-M-15}}$
	NODE_6length_2437_cov_10.664069	*aac(6')-Ib-cr*	*aac(6')-Ib-D181Y*
		aac(6')-Ib-cr	
		bla$_{\text{OXA-1}}$	*bla*$_{\text{OXA-1}}$
		catB3	
		catB3	
	NODE_73_length_1318_cov_20.963896	*qacE*	
		sul1	*sul1*
	NODE_8_length_270438_cov_16.598451	*fosA*	*fosA6*
SRR5132379	NODE_1length_194985_cov_17.055230	*oqxA*	*oqxA6*
		OqxA	
		oqxB	*oqxB20*
		OqxB	
	NODE_23_length_87700_cov_14.222352	*bla*$_{\text{NDM-1}}$	*bla*$_{\text{NDM-1}}$
			ble-$_{\text{MBL}}$
	NODE_28_length_67923_cov_12.609800	*bla*$_{\text{SHV-106}}$	*bla*$_{\text{SHV-106}}$
		bla$_{\text{SHV-28}}$	
	NODE_46_length_15637_cov_18.056738	*bla*$_{\text{CTX-M-15}}$	*bla*$_{\text{CTX-M-15}}$

	NODE_47_length_15138_cov_12.021651	*aac(6')-Ib-cr*	*aac(6')-Ib11*
		aac(6')-Ib3	
		qacE	
		rmtC	*rmtC*
		sul1	*sul1*
	NODE_4length_34273_cov_15.581854	*bla*CMY-6	*bla*CMY-6
	NODE_6_length_266519_cov_18.927250	*fosA*	*fosA6*
SRR5132381	NODE_28_length_15162_cov_5.885933	*armA*	*armA*
		*bla*NDM-1	*bla*NDM-1
		mph(E)	*mph(E)*
		msr(E)	*msr(E)*
		qacE	*ble-*MBL
		sul1	*sul1*
	NODE_3length_11862_cov_17.590712	*aph(3'')-Ib*	*aph(3'')-Ib*
		aph(6)-Id	*aph(6)-Id*
		*bla*CTX-M-15	*bla*CTX-M-15
		*bla*TEM-1B	*bla*TEM-1
		sul2	*sul2*
	NODE_4_length_462725_cov_18.468456	*oqxA*	*oqxA5*
		OqxA	
		oqxB	*oqxB19*
		OqxB	
	NODE_45_length_3430_cov_20.248562	*dfrA14*	*dfrA14*
	NODE_5_length_378105_cov_19.002175	*fosA*	*fosA6*
	NODE_50_length_2853_cov_15.960382	*aac(3)-IIa*	*aac(3)-IIe*
	NODE_6length_173cov_16.377805	*bla*OXA-1	*bla*OXA-1
		catB3	
		catB3	
	NODE_7_length_366220_cov_13.646218	*bla*SHV-106	*bla*SHV-106
		*bla*SHV-28	
	NODE_79_length_62cov_32.825911	*aac(6')-Ib-cr*	*aac(6')-Ib-D181Y*
		aac(6')-Ib-cr	
SRR5132447	NODE_14_length_139983_cov_18.669903	*bla*SHV-106	*bla*SHV-106
		*bla*SHV-28	
	NODE_23_length_46915_cov_29.545781	*aac(6')-Ib-cr*	*aac(6')-Ib-AKT*
		aac(6')-Ib3	
		armA	*armA*
		*bla*NDM-1	*bla*NDM-1
		mph(E)	*mph(E)*
		msr(E)	*msr(E)*
		qacE	*ble-*MBL

		sul1	*sul1*
	NODE_4_length_393629_cov_24.529898	*OqxA*	
		oqxA	*oqxA6*
		OqxB	
		oqxB	*oqxB20*
	NODE_44_length_225cov_26.119115	*aph(3'')-Ib*	
		aph(3'')-Ib	
		aph(3'')-Ib	
		aph(3'')-Ib	*aph(3'')-Ib*
		aph(6)-Id	*aph(6)-Id*
	NODE_45_length_223cov_48.153944	*bla*$_{OXA-48}$	*bla*$_{OXA-48}$
	NODE_46_length_2210_cov_30.425348	*qnrB1*	*qnrB1*
	NODE_47_length_1889_cov_27.252554	*dfrA14*	*dfrA14*
	NODE_48_length_1859_cov_29.484411	*bla*$_{OXA-1}$	*bla*$_{OXA-1}$
		catB3	
		catB3	
	NODE_4length_305cov_73.021888	*bla*$_{CTX-M-15}$	*bla*$_{CTX-M-15}$
	NODE_6_length_34991cov_26.072222	*fosA*	*fosA6*
SRR5132448	NODE_14_length_155996_cov_16.230476	*bla*$_{SHV-182}$	*bla*$_{SHV-158}$
	NODE_1length_196403_cov_24.243866	*fosA*	*fosA6*
	NODE_22_length_78307_cov_22.051740	*oqxA*	*oqxA*
		OqxA	
		oqxB	*oqxB*
		OqxB	
	NODE_47_length_8659_cov_24.728200	*aac(6')-Ib-cr*	*aac(6')-Ib-G*
		aac(6')-Ib-Hangzhou	*catB*
		ARR-2	*arr-2*
		rmtF	*rmtF1*
	NODE_48_length_5606_cov_16.931374	*bla*$_{NDM-1}$	*bla*$_{NDM-1}$
			ble-$_{MBL}$
	NODE_49_length_5350_cov_28.930691	*aadA2*	*aadA2*
		dfrA12	*dfrA12*
		qacE	
		sul1	*sul1*
	NODE_58_length_3813_cov_28.562398	*bla*$_{CTX-M-15}$	*bla*$_{CTX-M-15}$
	NODE_59_length_3484_cov_24.434018	*mph(A)*	*mph(A)*
	NODE_62_length_2854_cov_25.970297	*aac(3)-IIa*	*aac(3)-IIe*
	NODE_66_length_223cov_42.546226	*bla*$_{OXA-48}$	*bla*$_{OXA-48}$
	NODE_69_length_1880_cov_32.058756	*bla*$_{OXA-1}$	*bla*$_{OXA-1}$
		catB3	
		catB3	

	NODE_75_length_1288_cov_30.000000	*aph(3')-Ia*	*aph(3')-Ia*
SRR5132449	NODE_2_length_45399cov_15.103969	*bla*SHV-182	*bla*SHV-158
	NODE_4_length_373586_cov_18.810180	*oqxA*	*oqxA6*
		OqxA	
		oqxB	*oqxB20*
		OqxB	
	NODE_40_length_1412cov_7.792697	*aac(3)-IId*	*aac(3)-IId*
		aac(6')-Ib-cr	*aac(6')-Ib11*
		aac(6')-Ib3	
		qacE	
		sul1	*sul1*
	NODE_44_length_223cov_27.495759	*bla*OXA-48	*bla*OXA-48
	NODE_6_length_27045cov_20.714683	*fosA*	*fosA6*
SRR5132450	NODE_22_length_59874_cov_33.708772	*aadA1*	*aadA1*
		aadA1	*aadA1*
		aadA1	
		aadA1	
		armA	*armA*
		ARR-2	*arr-2*
		*bla*NDM-1	*bla*NDM-1
		*bla*OXA-10	*bla*OXA-10
		*bla*OXA-16	*bla*OXA-10
		cmlA1	*cmlA5*
		mph(E)	*mph(E)*
		msr(E)	*msr(E)*
		qacE	
		qacE	*ble-*MBL
		sul1	*sul1*
		sul1	*sul1*
	NODE_24_length_48065_cov_33.015562	*bla*CMY-16	*bla*CMY-16
		*bla*CMY-4	
	NODE_3_length_38248cov_20.694516	*oqxA*	*oqxA6*
		OqxA	
		oqxB	*oqxB20*
		OqxB	
	NODE_4_length_350192_cov_21.642052	*fosA*	*fosA6*
	NODE_44_length_13734_cov_23.902550	*qnrB1*	*qnrB1*
		tet(A)	*tet(A)*
	NODE_48_length_6979_cov_26.613398	*dfrA14*	*dfrA14*
	NODE_49_length_5886_cov_26.127279	*aph(3'')-Ib*	*aph(3'')-Ib*
		aph(6)-Id	*aph(6)-Id*
		*bla*TEM-1B	

		sul2	*sul2*
	NODE_53_length_3814_cov_24.033903	*bla*$_{CTX-M-15}$	*bla*$_{CTX-M-15}$
	NODE_57_length_2853_cov_23.218269	*aac(3)-IIa*	*aac(3)-IIe*
	NODE_58_length_2438_cov_29.871051	*aac(6')-Ib-cr*	*aac(6')-Ib-D181Y*
		aac(6')-Ib-cr	
		bla$_{OXA-1}$	*bla*$_{OXA-1}$
		catB3	
		catB3	
	NODE_7_length_279990_cov_15.522159	*bla*$_{SHV-106}$	*bla*$_{SHV-106}$
		bla$_{SHV-28}$	*bla*$_{TEM-1}$
SRR5132451	NODE_12_length_15130cov_29.288343	*fosA*	*fosA6*
	NODE_2_length_50841cov_20.165750	*bla*$_{SHV-145}$	*bla*$_{SHV-145}$
		bla$_{SHV-179}$	
		bla$_{SHV-194}$	
		bla$_{SHV-199}$	
		bla$_{SHV-26}$	
		bla$_{SHV-78}$	
		bla$_{SHV-98}$	
	NODE_5_length_439325_cov_27.245195	*OqxA*	
		oqxA	*oqxA9*
		OqxB	
		oqxB	*oqxB18*
SRR5132452	NODE_15_length_12018cov_20.924367	*aac(6')-Ib-cr*	*aac(6')-Ib-G*
		aac(6')-Ib-Hangzhou	
		ARR-2	*arr-2*
		rmtF	*rmtF1*
	NODE_16_length_116554_cov_19.976569	*OqxA*	
		oqxA	*oqxA10*
		OqxB	
		oqxB	*oqxB17*
	NODE_3_length_425880_cov_14.093040	*bla*$_{SHV-106}$	*bla*$_{SHV-212}$
		bla$_{SHV-28}$	
	NODE_38_length_5420_cov_12.641035	*aph(3'')-Ib*	*aph(3'')-Ib*
		sul2	*sul2*
	NODE_44_length_339cov_14.212316	*aph(6)-Id*	
		aph(6)-Id	
		bla$_{TEM-1B}$	*bla*$_{TEM-1}$
	NODE_46_length_285cov_13.441263	*aac(3)-IIa*	*aac(3)-IIe*
	NODE_48_length_223cov_19.693808	*bla*$_{OXA-48}$	*bla*$_{OXA-48}$
	NODE_5_length_35049cov_20.732079	*fosA*	*fosA_gen*
	NODE_50_length_188cov_14.583238	*bla*$_{OXA-1}$	*bla*$_{OXA-1}$

		catB3	*catB*
		catB3	
	NODE_9_length_291642_cov_15.128889	$bla_{\text{CTX-M-15}}$	$bla_{\text{CTX-M-15}}$
SRR5132453	NODE_17_length_116554_cov_17.562541	*OqxA*	
		oqxA	*oqxA10*
		OqxB	
		oqxB	*oqxB17*
	NODE_19_length_9967cov_17.015973	*aac(6')-Ib-cr*	*aac(6')-Ib-G*
		aac(6')-Ib-Hangzhou	*catB*
		ARR-2	*arr-2*
		rmtF	*rmtF1*
	NODE_24_length_52719_cov_11.694497	$bla_{\text{CTX-M-15}}$	$bla_{\text{CTX-M-15}}$
	NODE_3_length_350490_cov_18.528730	*fosA*	*fosA_gen*
	NODE_34_length_26062_cov_16.165953	*aph(6)-Id*	
		aph(6)-Id	
		$bla_{\text{TEM-1B}}$	$bla_{\text{TEM-1}}$
	NODE_47_length_5420_cov_11.171358	*aph(3'')-Ib*	*aph(3'')-Ib*
		sul2	*sul2*
	NODE_55_length_285cov_11.071219	*aac(3)-IIa*	*aac(3)-IIe*
	NODE_57_length_223cov_17.827396	$bla_{\text{OXA-48}}$	$bla_{\text{OXA-48}}$
	NODE_59_length_188cov_10.536488	$bla_{\text{OXA-1}}$	$bla_{\text{OXA-1}}$
		catB3	
		catB3	
	NODE_8_length_275522_cov_12.059485	$bla_{\text{SHV-106}}$	$bla_{\text{SHV-212}}$
		$bla_{\text{SHV-28}}$	
SRR5809251	NODE_6_length_361456_cov_100.881388	*OqxA*	
		oqxA	*oqxA5*
		OqxB	
		oqxB	*oqxB12*
	NODE_7_length_348877_cov_107.541640	*fosA*	*fosA6*
	NODE_length_1260665_cov_78.559940	$bla_{\text{SHV-190}}$	$bla_{\text{SHV-190}}$
SRR5809254	NODE_5_length_360894_cov_144.172033	*OqxA*	
		oqxA	*oqxA5*
		OqxB	
		oqxB	*oqxB12*
	NODE_6_length_348958_cov_147.785297	*fosA*	*fosA6*
	NODE_length_1260665_cov_126.414225	$bla_{\text{SHV-190}}$	$bla_{\text{SHV-190}}$
SRR5809260	NODE_10_length_159039_cov_129.015191	*fosA*	*fosA_gen*
	NODE_2_length_749377_cov_115.515662	$bla_{\text{CTX-M-15}}$	$bla_{\text{CTX-M-15}}$
	NODE_27_length_9364_cov_559.413338	*aac(6')-Ib-cr*	*aac(6')-Ib-G*

		aac(6')-Ib-Hangzhou	*catB*
		dfrA12	*dfrA12*
		rmtF	*rmtF1*
	NODE_3_length_729639_cov_126.460871	*oqxA*	*oqxA6*
		OqxA	
		oqxB	*oqxB19*
		OqxB	
	NODE_30_length_6087_cov_313.782550	*aph(3'')-Ib*	
		aph(3'')-Ib	
		aph(3'')-Ib	
		aph(3'')-Ib	*aph(3'')-Ib*
		aph(6)-Id	*aph(6)-Id*
		bla$_{TEM-1B}$	*bla*$_{TEM-1}$
	NODE_36_length_3485_cov_381.592615	*mph(A)*	*mph(A)*
	NODE_38_length_3230_cov_2502.272962	*bla*$_{OXA-181}$	*bla*$_{OXA-181}$
	NODE_3length_5847_cov_1190.469580	*qnrB1*	*qnrB1*
	NODE_44_length_1649_cov_296.906702	*catA2*	*catA2*
	NODE_47_length_1189_cov_411.836158	*dfrA14*	*dfrA14*
	NODE_55_length_749_cov_313.762058	*ARR-2*	*arr-2*
	NODE_length_1093317_cov_92.059476	*bla*$_{SHV-11}$	*bla*$_{SHV-11}$
		bla$_{SHV-67}$	
SRR5809261	NODE_6_length_361862_cov_130.018362	*oqxA*	*oqxA5*
		OqxA	
		oqxB	*oqxB12*
		OqxB	
	NODE_7_length_348958_cov_136.139348	*fosA*	*fosA6*
	NODE_length_1260665_cov_101.866463	*bla*$_{SHV-190}$	*bla*$_{SHV-190}$
SRR5809262	NODE_10_length_159039_cov_76.186455	*fosA*	*fosA_gen*
	NODE_2_length_749377_cov_73.169211	*bla*$_{CTX-M-15}$	*bla*$_{CTX-M-15}$
	NODE_29_length_9364_cov_308.223882	*aac(6')-Ib-cr*	*aac(6')-Ib-G*
		aac(6')-Ib-Hangzhou	*catB*
		dfrA12	*dfrA12*
		rmtF	*rmtF1*
	NODE_3_length_729638_cov_75.779170	*OqxA*	
		oqxA	*oqxA6*
		OqxB	
		oqxB	*oqxB19*
	NODE_32_length_6087_cov_120.530369	*aph(3'')-Ib*	
		aph(3'')-Ib	
		aph(3'')-Ib	
		aph(3'')-Ib	*aph(3'')-Ib*

		aph(6)-Id	*aph(6)-Id*
		bla$_{\text{TEM-1B}}$	*bla*$_{\text{TEM-1}}$
	NODE_33_length_5847_cov_760.828671	*qnrB1*	*qnrB1*
	NODE_37_length_3485_cov_186.763550	*mph(A)*	*mph(A)*
	NODE_39_length_3230_cov_1638.771834	*bla*$_{\text{OXA-181}}$	*bla*$_{\text{OXA-181}}$
	NODE_45_length_1649_cov_117.251643	*catA2*	*catA2*
	NODE_48_length_1189_cov_242.245763	*dfrA14*	*dfrA14*
	NODE_56_length_749_cov_186.601286	*ARR-2*	*arr-2*
	NODE_length_1093317_cov_66.199585	*bla*$_{\text{SHV-11}}$	*bla*$_{\text{SHV-11}}$
		bla$_{\text{SHV-67}}$	
SRR5809264	NODE_2_length_749377_cov_94.931315	*bla*$_{\text{CTX-M-15}}$	*bla*$_{\text{CTX-M-15}}$
	NODE_29_length_9364_cov_264.080870	*aac(6')-Ib-cr*	*aac(6')-Ib-G*
		aac(6')-Ib-Hangzhou	*catB*
		dfrA12	*dfrA12*
		rmtF	*rmtF1*
	NODE_3_length_641956_cov_103.288523	*OqxA*	
		oqxA	*oqxA6*
		OqxB	
		oqxB	*oqxB19*
	NODE_32_length_6087_cov_144.477349	*aph(3'')-Ib*	
		aph(3'')-Ib	
		aph(3'')-Ib	
		aph(3'')-Ib	*aph(3'')-Ib*
		aph(6)-Id	*aph(6)-Id*
		bla$_{\text{TEM-1B}}$	*bla*$_{\text{TEM-1}}$
	NODE_33_length_5847_cov_830.519580	*qnrB1*	*qnrB1*
	NODE_37_length_3485_cov_129.150983	*mph(A)*	*mph(A)*
	NODE_39_length_3230_cov_2382.479214	*bla*$_{\text{OXA-181}}$	*bla*$_{\text{OXA-181}}$
	NODE_46_length_1649_cov_157.766097	*catA2*	*catA2*
	NODE_49_length_1189_cov_190.660075	*dfrA14*	*dfrA14*
	NODE_57_length_749_cov_217.483923	*ARR-2*	*arr-2*
	NODE_9_length_159039_cov_109.023522	*fosA*	*fosA_gen*
	NODE_length_1092290_cov_77.232464	*bla*$_{\text{SHV-11}}$	*bla*$_{\text{SHV-11}}$
		bla$_{\text{SHV-67}}$	
SRR5809265	NODE_10_length_159039_cov_239.842567	*fosA*	*fosA_gen*
	NODE_2_length_749377_cov_211.524852	*bla*$_{\text{CTX-M-15}}$	*bla*$_{\text{CTX-M-15}}$
	NODE_27_length_9364_cov_521.701418	*aac(6')-Ib-cr*	*aac(6')-Ib-G*
		aac(6')-Ib-Hangzhou	*catB*
		dfrA12	*dfrA12*
		rmtF	*rmtF1*

	NODE_3_length_729638_cov_232.431022	*OqxA*	
		oqxA	*oqxA6*
		OqxB	
		oqxB	*oqxB19*
	NODE_30_length_6087_cov_284.501846	*aph(3'')-Ib*	
		aph(3'')-Ib	
		aph(3'')-Ib	
		aph(3'')-Ib	*aph(3'')-Ib*
		aph(6)-Id	*aph(6)-Id*
		bla$_{\text{TEM-1B}}$	*bla*$_{\text{TEM-1}}$
	NODE_35_length_3485_cov_266.449375	*mph(A)*	*mph(A)*
	NODE_38_length_3230_cov_4729.368675	*bla*$_{\text{OXA-181}}$	*bla*$_{\text{OXA-181}}$
	NODE_3length_5847_cov_1671.865559	*qnrB1*	*qnrB1*
	NODE_43_length_1649_cov_287.047306	*catA2*	*catA2*
	NODE_46_length_1189_cov_350.673258	*dfrA14*	*dfrA14*
	NODE_54_length_749_cov_404.995177	*ARR-2*	*arr-2*
	NODE_length_880809_cov_173.507475	*bla*$_{\text{SHV-11}}$	*bla*$_{\text{SHV-11}}$
		bla$_{\text{SHV-67}}$	
SRR5809266	NODE_5_length_361310_cov_203.324495	*OqxA*	
		oqxA	*oqxA5*
		OqxB	
		oqxB	*oqxB12*
	NODE_6_length_348958_cov_218.834203	*fosA*	*fosA6*
	NODE_length_1260665_cov_175.257801	*bla*$_{\text{SHV-190}}$	*bla*$_{\text{SHV-190}}$
SRR5809267	NODE_2_length_749377_cov_106.895486	*bla*$_{\text{CTX-M-15}}$	*bla*$_{\text{CTX-M-15}}$
	NODE_26_length_9364_cov_521.621847	*aac(6')-Ib-cr*	*aac(6')-Ib-G*
		aac(6')-Ib-Hangzhou	*catB*
		dfrA12	*dfrA12*
		rmtF	*rmtF1*
	NODE_29_length_6087_cov_272.649497	*aph(3'')-Ib*	
		aph(3'')-Ib	
		aph(3'')-Ib	
		aph(3'')-Ib	*aph(3'')-Ib*
		aph(6)-Id	*aph(6)-Id*
		bla$_{\text{TEM-1B}}$	*bla*$_{\text{TEM-1}}$
	NODE_3_length_729639_cov_116.711885	*OqxA*	
		oqxA	*oqxA6*
		OqxB	
		oqxB	*oqxB19*
	NODE_30_length_5847_cov_1025.049301	*qnrB1*	*qnrB1*
	NODE_34_length_3485_cov_346.548541	*mph(A)*	*mph(A)*

	NODE_36_length_3230_cov_2133.609088	*bla*$_{\text{OXA-181}}$	*bla*$_{\text{OXA-181}}$
	NODE_42_length_1649_cov_248.431012	*catA2*	*catA2*
	NODE_45_length_1189_cov_382.949153	*dfrA14*	*dfrA14*
	NODE_53_length_749_cov_261.363344	*ARR-2*	*arr-2*
	NODE_9_length_159039_cov_119.198475	*fosA*	*fosA_gen*
	NODE_length_1093315_cov_85.561457	*bla*$_{\text{SHV-11}}$	*bla*$_{\text{SHV-11}}$
		bla$_{\text{SHV-67}}$	
SRR5809268	NODE_5_length_361456_cov_186.822829	*oqxA*	*oqxA5*
		OqxA	
		oqxB	*oqxB12*
		OqxB	
	NODE_6_length_348958_cov_200.187802	*fosA*	*fosA6*
	NODE_length_1260665_cov_158.534500	*bla*$_{\text{SHV-190}}$	*bla*$_{\text{SHV-190}}$
SRR8607448	NODE_13_length_159039_cov_13.458291	*fosA*	*fosA_gen*
	NODE_15_length_119463_cov_27.021821	*sul2*	*sul2*
	NODE_2_length_607483_cov_14.287619	*bla*$_{\text{SHV-11}}$	*bla*$_{\text{SHV-11}}$
		bla$_{\text{SHV-67}}$	
	NODE_34_length_12920_cov_19.963105	*bla*$_{\text{TEM-1B}}$	*bla*$_{\text{TEM-1}}$
		rmtB	*rmtB1*
		tet(G)	*tet(G)*
			floR2
	NODE_4_length_350410_cov_12.816563	*OqxA*	
		oqxA	*oqxA6*
		OqxB	
		oqxB	*oqxB19*
	NODE_42_length_7354_cov_22.604677	*dfrA23*	*dfrA23*
	NODE_44_length_5175_cov_25.824089	*ant(2'')-Ia*	*ant(2'')-Ia*
		ARR-2	*arr-2*
		bla$_{\text{OXA-10}}$	*bla*$_{\text{OXA-10}}$
		bla$_{\text{VEB-1}}$	*bla*$_{\text{VEB-1}}$
		cmlA1	*cmlA5*
	NODE_46_length_4609_cov_18.664212	*tet(A)*	*tet(A)*
	NODE_57_length_2463_cov_30.339897	*aac(6')-Il*	*aac(6')-Il*
		bla$_{\text{VIM-27}}$	*bla*$_{\text{VIM-27}}$
		dfrA1	*dfrA1*
		dfrA1	
		dfrA1	
	NODE_5length_3484_cov_21.880548	*mph(A)*	*mph(A)*
	NODE_63_length_1518_cov_59.112150	*aph(3'')-Ib*	
		aph(3'')-Ib	
		aph(3'')-Ib	
		aph(6)-Id	*aph(6)-Id*

	NODE_66_length_1454_cov_33.929917	*aph(3')-Ia*	*aph(3')-Ia*
	NODE_76_length_980_cov_56.150059	*qacE*	
		sul1	
	NODE_77_length_93cov_10.344527	*aac(6')-Ib-cr*	*aac(6')-Ib-AKT*
		aac(6')-Ib3	
	NODE_7length_1189_cov_34.515066	*dfrA14*	*dfrA14*
	NODE_88_length_673_cov_58.901099	*aadA1*	*aadA1*
		aadA22	
SRR8607449	NODE_107_length_98cov_77.682670	*qacE*	
		sul1	
	NODE_117_length_587_cov_96.086957	*bla*$_{TEM-104}$	
		bla$_{TEM-141}$	
		bla$_{TEM-164}$	
		bla$_{TEM-1B}$	
		bla$_{TEM-206}$	
		bla$_{TEM-209}$	
		bla$_{TEM-210}$	
		bla$_{TEM-214}$	
		bla$_{TEM-216}$	
		bla$_{TEM-33}$	
		bla$_{TEM-34}$	
	NODE_15_length_151533_cov_35.608602	*bla*$_{SHV-182}$	*bla*$_{SHV-187}$
	NODE_17_length_125336_cov_53.977310	*aph(3'')-Ib*	*aph(3'')-Ib*
		aph(6)-Id	*aph(6)-Id*
		sul2	*sul2*
		tet(A)	*tet(A)*
	NODE_45_length_20517_cov_55.803678	*bla*$_{KPC-2}$	*bla*$_{KPC-2}$
	NODE_49_length_16907_cov_45.329857	*catA1*	*catA1*
	NODE_5_length_270452_cov_35.921787	*fosA*	*fosA6*
	NODE_57_length_10034_cov_41.564046	*rmtB*	*rmtB1*
		tet(G)	*tet(G)*
			floR2
	NODE_64_length_6937_cov_46.406021	*dfrA23*	*dfrA23*
	NODE_65_length_594cov_46.805470	*aadA1*	*aadA1*
		ant(2'')-Ia	*ant(2'')-Ia*
		ARR-2	*arr-2*
		bla$_{OXA-10}$	*bla*$_{OXA-10}$
		bla$_{VEB-1}$	*bla*$_{VEB-1}$
		cmlA1	*cmlA5*
	NODE_72_length_3484_cov_34.576408	*mph(A)*	*mph(A)*
	NODE_80_length_2045_cov_210.351929	*aac(6')-Ib*	*aac(6')-Ib-AKT*
		aac(6')-Ib-cr	

	NODE_88_length_1443_cov_54.449088	*bla*$_{OXA-9}$	*bla*$_{OXA-9}$
	NODE_8length_2036_cov_46.918282	*aadA2*	*aadA2*
		dfrA12	*dfrA12*
	NODE_95_length_1292_cov_43.358798	*aph(3')-Ia*	*aph(3')-Ia*
	NODE_99_length_1186_cov_53.673277	*dfrA14*	*dfrA14*
	NODE_length_499304_cov_34.712499	*OqxA*	
		oqxA	*oqxA*
		OqxB	
		oqxB	*oqxB*
SRR8607450	NODE_13_length_15214cov_39.776909	*bla*$_{SHV-182}$	*bla*$_{SHV-158}$
	NODE_15_length_11966cov_49.179756	*aph(3'')-Ib*	
		sul2	*sul2*
	NODE_155_length_244_cov_81.119658	*aph(6)-Id*	
	NODE_24_length_67992_cov_41.442201	*bla*$_{KPC-2}$	*bla*$_{KPC-2}$
	NODE_39_length_18576_cov_41.176161	*aadA1*	*aadA1*
		ant(2'')-Ia	*ant(2'')-Ia*
		ARR-2	*arr-2*
		bla$_{OXA-10}$	*bla*$_{OXA-10}$
		bla$_{TEM-1D}$	*bla*$_{TEM-12}$
		bla$_{VEB-1}$	*bla*$_{VEB-1}$
		cmlA1	*cmlA5*
		qacE	
		rmtB	*rmtB1*
		sul1	*floR2*
		tet(G)	*tet(G)*
	NODE_48_length_735cov_38.730482	*dfrA23*	*dfrA23*
	NODE_53_length_4649_cov_35.313799	*tet(A)*	*tet(A)*
	NODE_56_length_3484_cov_32.292225	*mph(A)*	*mph(A)*
	NODE_57_length_3387_cov_76.198466	*aph(6)-Id*	
		aph(6)-Id	
	NODE_6_length_270452_cov_39.997100	*fosA*	*fosA6*
	NODE_65_length_1415_cov_68.767081	*dfrA14*	*dfrA14*
	NODE_76_length_1049_cov_159.623644	*aac(6')-Ib*	*aac(6')-Ib-AKT*
		aac(6')-Ib-cr	
	NODE_7length_1292_cov_40.217167	*aph(3')-Ia*	*aph(3')-Ia*
	NODE_83_length_560_cov_98.420323	*aph(6)-Id*	
		aph(6)-Id	
	NODE_length_803719_cov_39.890020	*OqxA*	
		oqxA	*oqxA*
		OqxB	
		oqxB	*oqxB*
	NODE_17_length_129979_cov_56.430598	*OqxA*	

SRR8607451		*oqxA*	*oqxA*
		OqxB	
		oqxB	*oqxB*
	NODE_19_length_125319_cov_59.092666	*aph(3'')-Ib*	*aph(3'')-Ib*
		aph(6)-Id	*aph(6)-Id*
		sul2	*sul2*
		tet(A)	*tet(A)*
	NODE_29_length_51803_cov_56.560279	*aadA2*	*aadA2*
		catA1	*catA1*
		dfrA12	*dfrA12*
	NODE_35_length_33670_cov_56.726798	*fosA*	*fosA6*
	NODE_3length_42814_cov_75.606180	*bla*$_{\text{SHV-12}}$	*bla*$_{\text{SHV-12}}$
	NODE_48_length_12129_cov_74.621646	*aadA1*	*aadA1*
		ant(2'')-Ia	*ant(2'')-Ia*
		ARR-2	*arr-2*
		bla$_{\text{OXA-10}}$	*bla*$_{\text{OXA-10}}$
		bla$_{\text{VEB-1}}$	*bla*$_{\text{VEB-1}}$
		cmlA1	*cmlA5*
	NODE_4length_20517_cov_57.424326	*bla*$_{\text{KPC-2}}$	*bla*$_{\text{KPC-2}}$
	NODE_52_length_10034_cov_44.233068	*rmtB*	*rmtB1*
		tet(G)	*tet(G)*
			floR2
	NODE_64_length_3484_cov_42.867143	*mph(A)*	*mph(A)*
	NODE_76_length_1443_cov_66.438450	*bla*$_{\text{OXA-9}}$	*bla*$_{\text{OXA-9}}$
	NODE_7length_2045_cov_214.358186	*aac(6')-Ib*	*aac(6')-Ib-AKT*
		aac(6')-Ib-cr	
	NODE_89_length_98cov_95.594848	*qacE*	
		sul1	
	NODE_97_length_587_cov_119.102174	*bla*$_{\text{TEM-104}}$	
		bla$_{\text{TEM-141}}$	
		bla$_{\text{TEM-164}}$	
		bla$_{\text{TEM-1B}}$	
		bla$_{\text{TEM-206}}$	
		bla$_{\text{TEM-209}}$	
		bla$_{\text{TEM-210}}$	
		bla$_{\text{TEM-214}}$	
		bla$_{\text{TEM-216}}$	
		bla$_{\text{TEM-33}}$	
		bla$_{\text{TEM-34}}$	
SRR8607452	NODE_12_length_166805_cov_33.600121	*fosA*	*fosA_gen*
	NODE_14_length_135880_cov_30.708065	*OqxA*	
		oqxA	*oqxA10*

		OqxB	
		oqxB	*oqxB13*
	NODE_3_length_391604_cov_32.272844	*bla*$_{SHV-145}$	*bla*$_{SHV-145}$
		bla$_{SHV-179}$	
		bla$_{SHV-194}$	
		bla$_{SHV-199}$	
		bla$_{SHV-26}$	
		bla$_{SHV-78}$	
		bla$_{SHV-98}$	
	NODE_36_length_29803_cov_48.566316	*aph(3'')-Ib*	*aph(3'')-Ib*
		aph(6)-Id	*aph(6)-Id*
		sul2	*sul2*
	NODE_58_length_3813_cov_74.143787	*bla*$_{CTX-M-15}$	*bla*$_{CTX-M-15}$
	NODE_59_length_3745_cov_34.908513	*mph(A)*	*mph(A)*
	NODE_5length_6468_cov_44.860590	*floR*	*floR*
	NODE_60_length_3078_cov_70.222636	*bla*$_{CMY-4}$	*bla*$_{CMY-4}$
	NODE_62_length_2405_cov_55.390694	*aac(6')-Il*	*aac(6')-Il*
		bla$_{VIM-19}$	*bla*$_{VIM-19}$
		dfrA1	*dfrA1*
		dfrA1	
		dfrA1	
	NODE_63_length_2262_cov_44.738642	*aadA2*	*aadA2*
		dfrA12	*dfrA12*
	NODE_64_length_2042_cov_72.198433	*tet(A)*	*tet(A)*
	NODE_68_length_1597_cov_63.112925	*qacE*	
		sul1	*sul1*
	NODE_6length_2532_cov_45.671518	*aac(3)-I*	*aac(3)-Ia*
		aac(6')-Ib-cr	*aac(6')-Ib-AKT*
		aac(6')-Ib3	
	NODE_74_length_1220_cov_56.112534	*aph(3')-Ia*	*aph(3')-Ia*
	NODE_86_length_673_cov_133.386447	*aadA23*	*aadA1*
SRR8607453	NODE_12_length_166805_cov_42.762926	*fosA*	*fosA_gen*
	NODE_15_length_135880_cov_38.107843	*OqxA*	
		oqxA	*oqxA10*
		OqxB	
		oqxB	*oqxB13*
	NODE_3_length_391604_cov_39.195516	*bla*$_{SHV-145}$	*bla*$_{SHV-145}$
		bla$_{SHV-179}$	
		bla$_{SHV-194}$	
		bla$_{SHV-199}$	
		bla$_{SHV-26}$	
		bla$_{SHV-78}$	

		bla$_{SHV-98}$	
	NODE_35_length_29803_cov_65.637316	*aph(3'')-Ib*	*aph(3'')-Ib*
		aph(6)-Id	*aph(6)-Id*
		sul2	*sul2*
	NODE_47_length_6468_cov_60.575461	*floR*	*floR*
	NODE_55_length_3813_cov_112.312263	*bla*$_{CTX-M-15}$	*bla*$_{CTX-M-15}$
	NODE_56_length_3745_cov_42.547264	*mph(A)*	*mph(A)*
	NODE_57_length_3078_cov_92.394781	*bla*$_{CMY-4}$	*bla*$_{CMY-4}$
	NODE_58_length_2532_cov_83.019543	*aac(3)-I*	*aac(3)-Ia*
		aac(6')-Ib-cr	*aac(6')-Ib-AKT*
		aac(6')-Ib3	
	NODE_59_length_2278_cov_80.657369	*aac(6')-Il*	*aac(6')-Il*
		bla$_{VIM-19}$	*bla*$_{VIM-19}$
		dfrA1	*dfrA1*
		dfrA1	
		dfrA1	
	NODE_60_length_2262_cov_55.167681	*aadA2*	*aadA2*
		dfrA12	*dfrA12*
	NODE_68_length_1597_cov_87.489796	*qacE*	
		sul1	*sul1*
	NODE_6length_2042_cov_92.730026	*tet(A)*	*tet(A)*
	NODE_73_length_1220_cov_82.934126	*aph(3')-Ia*	*aph(3')-Ia*
	NODE_85_length_673_cov_200.307692	*aadA23*	*aadA1*
SRR8607454	NODE_1length_159264_cov_49.876364	*oqxA*	*oqxA*
		OqxA	
		oqxB	*oqxB*
		OqxB	
	NODE_26_length_59380_cov_43.201475	*aadA2*	*aadA2*
		catA1	*catA1*
		dfrA12	*dfrA12*
		mph(A)	*mph(A)*
		qacE	
		sul1	*sul1*
	NODE_37_length_20517_cov_48.664051	*bla*$_{KPC-2}$	*bla*$_{KPC-2}$
	NODE_3length_37143_cov_64.029447	*bla*$_{OXA-9}$	*bla*$_{OXA-9}$
		bla$_{TEM-1A}$	*bla*$_{TEM-1}$
	NODE_4length_12954_cov_487.406954	*aac(6')-Ib*	*aac(6')-Ib-AKT*
		aac(6')-Ib-cr	
	NODE_58_length_1292_cov_48.686695	*aph(3')-Ia*	*aph(3')-Ia*
	NODE_68_length_722_cov_170.388235	*bla*$_{SHV-12}$	*bla*$_{SHV-187}$
		bla$_{SHV-129}$	
		bla$_{SHV-13}$	

		bla$_{SHV-155}$	
		bla$_{SHV-172}$	
		bla$_{SHV-31}$	
	NODE_7_length_270452_cov_52.793515	*fosA*	*fosA6*
SRR8607455	NODE_14_length_150867_cov_51.663487	*bla*$_{SHV-182}$	*bla*$_{SHV-187}$
	NODE_17_length_125336_cov_57.837112	*aph(3'')-Ib*	*aph(3'')-Ib*
		aph(6)-Id	*aph(6)-Id*
		sul2	*sul2*
		tet(A)	*tet(A)*
	NODE_28_length_67826_cov_87.152395	*bla*$_{KPC-2}$	*bla*$_{KPC-2}$
	NODE_39_length_13584_cov_79.744817	*bla*$_{TEM-122}$	
		bla$_{TEM-135}$	
		bla$_{TEM-141}$	
		bla$_{TEM-1B}$	*bla*$_{TEM-1}$
		bla$_{TEM-1C}$	
		bla$_{TEM-209}$	
		bla$_{TEM-29}$	
		bla$_{TEM-55}$	
		bla$_{TEM-57}$	
	NODE_4_length_270452_cov_53.580816	*fosA*	*fosA6*
	NODE_42_length_10034_cov_45.346523	*rmtB*	*rmtB1*
		tet(G)	*tet(G)*
			floR2
	NODE_49_length_6937_cov_50.475330	*dfrA23*	*dfrA23*
	NODE_50_length_6593_cov_51.169038	*catA1*	*catA1*
	NODE_56_length_3474_cov_39.161936	*mph(A)*	*mph(A)*
	NODE_5length_5943_cov_51.163686	*aadA1*	*aadA1*
		ant(2'')-Ia	*ant(2'')-Ia*
		ARR-2	*arr-2*
		bla$_{OXA-10}$	*bla*$_{OXA-10}$
		bla$_{VEB-1}$	*bla*$_{VEB-1}$
		cmlA1	*cmlA5*
	NODE_63_length_2045_cov_335.835766	*aac(6')-Ib*	*aac(6')-Ib-AKT*
		aac(6')-Ib-cr	
	NODE_66_length_1443_cov_51.363222	*bla*$_{OXA-9}$	*bla*$_{OXA-9}$
	NODE_6length_2242_cov_49.985343	*aadA2*	*aadA2*
		dfrA12	*dfrA12*
	NODE_72_length_129cov_42.878866	*aph(3')-Ia*	*aph(3')-Ia*
	NODE_75_length_1186_cov_54.296506	*dfrA14*	*dfrA14*
	NODE_8length_98cov_78.613583	*qacE*	
		sul1	
	NODE_length_805090_cov_53.629948	*oqxA*	*oqxA*

		OqxA	
		oqxB	*oqxB*
		OqxB	
SRR8607456	NODE_13_length_15769cov_61.814539	*bla*$_{SHV-182}$	*bla*$_{SHV-158}$
	NODE_25_length_59380_cov_53.584527	*aadA2*	*aadA2*
		catA1	*catA1*
		dfrA12	*dfrA12*
		mph(A)	*mph(A)*
		qacE	
		sul1	*sul1*
	NODE_35_length_20518_cov_54.244765	*bla*$_{KPC-2}$	*bla*$_{KPC-2}$
	NODE_40_length_13275_cov_220.909264	*aac(6')-Ib*	*aac(6')-Ib-AKT*
		aac(6')-Ib-cr	
	NODE_5_length_259736_cov_62.937860	*fosA*	*fosA6*
	NODE_58_length_1443_cov_49.182371	*bla*$_{OXA-9}$	*bla*$_{OXA-9}$
	NODE_6length_1292_cov_54.428326	*aph(3')-Ia*	*aph(3')-Ia*
	NODE_70_length_923_cov_47.054020	*bla*$_{TEM-1A}$	*bla*$_{TEM-1}$
	NODE_length_804259_cov_62.755962	*oqxA*	*oqxA*
		OqxA	
		oqxB	*oqxB*
		OqxB	
SRR8607457	NODE_28_length_59334_cov_47.498015	*aadA2*	*aadA2*
		catA1	*catA1*
		dfrA12	*dfrA12*
		mph(A)	*mph(A)*
		qacE	
		sul1	*sul1*
	NODE_3_length_347754_cov_43.285921	*oqxA*	*oqxA*
		OqxA	
		oqxB	*oqxB*
		OqxB	
	NODE_45_length_12954_cov_217.918141	*aac(6')-Ib*	*aac(6')-Ib-AKT*
		aac(6')-Ib-cr	
	NODE_50_length_6637_cov_66.459293	*bla*$_{TEM-1A}$	*bla*$_{TEM-1}$
	NODE_6_length_270452_cov_44.939373	*fosA*	*fosA6*
	NODE_60_length_3964_cov_121.654157	*bla*$_{KPC-2}$	*bla*$_{KPC-2}$
	NODE_62_length_2204_cov_57.085701	*bla*$_{SHV-182}$	*bla*$_{SHV-187}$
	NODE_69_length_1443_cov_47.559271	*bla*$_{OXA-9}$	*bla*$_{OXA-9}$
	NODE_73_length_1292_cov_49.823176	*aph(3')-Ia*	*aph(3')-Ia*
SRR8607458	NODE_100_length_884_cov_55.388375	*bla*$_{SHV-12}$	*bla*$_{SHV-12}$
	NODE_27_length_58994_cov_23.518406	*aadA2*	*aadA2*
		catA1	*catA1*

		dfrA12	*dfrA12*
		mph(A)	*mph(A)*
		qacE	
		sul1	*sul1*
	NODE_32_length_40236_cov_26.325089	*bla*KPC-2	*bla*KPC-2
	NODE_5_length_270452_cov_37.383117	*fosA*	*fosA6*
	NODE_86_length_1292_cov_24.224893	*aph(3')-Ia*	*aph(3')-Ia*
	NODE_8length_1443_cov_15.065350	*bla*OXA-9	*bla*OXA-9
	NODE_93_length_1128_cov_279.207792	*aac(6')-Ib*	*aac(6')-Ib-AKT*
		aac(6')-Ib-cr	
	NODE_99_length_923_cov_16.360553	*bla*TEM-1A	*bla*TEM-1
	NODE_length_475533_cov_36.064877	*oqxA*	*oqxA*
		OqxA	
		oqxB	*oqxB*
		OqxB	
SRR8607459	NODE_14_length_156830_cov_18.137477	*bla*SHV-182	*bla*SHV-158
	NODE_17_length_125337_cov_19.074778	*aph(3'')-Ib*	*aph(3'')-Ib*
		aph(6)-Id	*aph(6)-Id*
		sul2	*sul2*
		tet(A)	*tet(A)*
	NODE_26_length_78308_cov_19.440414	*OqxA*	
		oqxA	*oqxA*
		OqxB	
		oqxB	*oqxB*
	NODE_44_length_14484_cov_18.253256	*bla*TEM-1B	*bla*TEM-1
		rmtB	*rmtB1*
		tet(G)	*tet(G)*
			floR2
	NODE_54_length_6145_cov_16.816218	*aadA1*	*aadA1*
		ant(2'')-Ia	*ant(2'')-Ia*
		ARR-2	*arr-2*
		*bla*OXA-10	*bla*OXA-10
		*bla*VEB-1	*bla*VEB-1
		cmlA1	*cmlA5*
	NODE_62_length_3485_cov_13.571173	*mph(A)*	*mph(A)*
	NODE_63_length_342cov_18.066181	*aadA2*	*aadA2*
		dfrA12	*dfrA12*
	NODE_66_length_285cov_18.164097	*aac(3)-IIa*	*aac(3)-IIe*
	NODE_7_length_270450_cov_20.569386	*fosA*	*fosA6*
	NODE_70_length_2435_cov_12.987435	*aac(6')-Ib-cr*	*aac(6')-Ib-D181Y*
		aac(6')-Ib-cr	

		bla$_{OXA-1}$	*bla*$_{OXA-1}$
		catB3	
		catB3	
	NODE_76_length_1569_cov_19.325936	*bla*$_{CTX-M-15}$	*bla*$_{CTX-M-15}$
	NODE_79_length_1288_cov_14.708010	*aph(3')-Ia*	*aph(3')-Ia*
	NODE_7length_223cov_25.994487	*bla*$_{OXA-48}$	*bla*$_{OXA-48}$
	NODE_86_length_98cov_27.900468	*qacE*	
		sul1	
SRR8607460	NODE_14_length_156830_cov_21.904986	*bla*$_{SHV-182}$	*bla*$_{SHV-158}$
	NODE_17_length_125337_cov_31.173516	*aph(3'')-Ib*	*aph(3'')-Ib*
		aph(6)-Id	*aph(6)-Id*
		sul2	*sul2*
		tet(A)	*tet(A)*
	NODE_26_length_78308_cov_23.853046	*OqxA*	
		oqxA	*oqxA*
		OqxB	
		oqxB	*oqxB*
	NODE_48_length_14507_cov_26.423574	*bla*$_{TEM-1B}$	*bla*$_{TEM-1}$
		rmtB	*rmtB1*
		tet(G)	*tet(G)*
			floR2
	NODE_59_length_6145_cov_25.502825	*aadA1*	*aadA1*
		ant(2'')-Ia	*ant(2'')-Ia*
		ARR-2	*arr-2*
		bla$_{OXA-10}$	*bla*$_{OXA-10}$
		bla$_{VEB-1}$	*bla*$_{VEB-1}$
		cmlA1	*cmlA5*
	NODE_69_length_3484_cov_8.100387	*mph(A)*	*mph(A)*
	NODE_70_length_342cov_10.398604	*aadA2*	*aadA2*
		dfrA12	*dfrA12*
	NODE_73_length_2854_cov_25.852952	*aac(3)-IIa*	*aac(3)-IIe*
	NODE_77_length_2435_cov_20.337955	*aac(6')-Ib-cr*	*aac(6')-Ib-D181Y*
		aac(6')-Ib-cr	
		bla$_{OXA-1}$	*bla*$_{OXA-1}$
		catB3	
		catB3	
	NODE_78_length_223cov_27.895674	*bla*$_{OXA-48}$	*bla*$_{OXA-48}$
	NODE_8_length_270450_cov_25.828076	*fosA*	*fosA6*
	NODE_82_length_1569_cov_9.239945	*bla*$_{CTX-M-15}$	*bla*$_{CTX-M-15}$
	NODE_94_length_98cov_33.473068	*qacE*	
		sul1	

SRR8607461	NODE_10_length_201017_cov_33.853387	*fosA*	*fosA6*
	NODE_14_length_151820_cov_30.943709	*bla*$_{SHV-182}$	*bla*$_{SHV-158}$
	NODE_3length_35956_cov_34.434732	*bla*$_{KPC-2}$	*bla*$_{KPC-2}$
	NODE_42_length_13275_cov_382.995589	*aac(6')-Ib*	*aac(6')-Ib-AKT*
		aac(6')-Ib-cr	
	NODE_46_length_11160_cov_64.728904	*aadA2*	*aadA2*
		dfrA12	*dfrA12*
		mph(A)	*mph(A)*
		qacE	
		sul1	*sul1*
	NODE_63_length_1292_cov_31.412876	*aph(3')-Ia*	*aph(3')-Ia*
	NODE_length_804764_cov_34.325337	*oqxA*	*oqxA*
		OqxA	
		oqxB	*oqxB*
		OqxB	
SRR8607462	NODE_2_length_512065_cov_47.217288	*bla*$_{SHV-182}$	*bla*$_{SHV-158}$
	NODE_26_length_75485_cov_51.602232	*oqxA*	*oqxA*
		OqxA	
		oqxB	*oqxB*
		OqxB	
	NODE_3_length_270452_cov_51.959053	*fosA*	*fosA6*
	NODE_32_length_39002_cov_93.960463	*dfrA30*	*dfrA30*
		qacE	
	NODE_38_length_22507_cov_106.100223	*bla*$_{KPC-2}$	*bla*$_{KPC-2}$
	NODE_44_length_7900_cov_114.870063	*tet(A)*	*tet(A)*
	NODE_45_length_7438_cov_55.423061	*mph(A)*	*mph(A)*
		sul1	*sul1*
	NODE_52_length_2437_cov_77.539827	*aac(6')-Ib-cr*	*aac(6')-Ib-D181Y*
		aac(6')-Ib-cr	
		bla$_{OXA-1}$	*bla*$_{OXA-1}$
		catB3	
		catB3	
SRR8607463	NODE_2_length_351532_cov_48.606107	*bla*$_{SHV-182}$	*bla*$_{SHV-158}$
	NODE_38_length_39542_cov_110.493645	*catA1*	*catA1*
	NODE_3length_53323_cov_109.916667	*bla*$_{KPC-2}$	*bla*$_{KPC-2}$
	NODE_5_length_270449_cov_54.401817	*fosA*	*fosA6*
	NODE_55_length_10333_cov_111.224672	*aac(3)-IIa*	*aac(3)-IIe*
		bla$_{TEM-1B}$	*bla*$_{TEM-1}$
	NODE_57_length_8450_cov_181.544996	*aadA2*	*aadA2*
		bla$_{CTX-M-2}$	*bla*$_{CTX-M-2}$
		dfrA12	*dfrA12*

		qacE	
		qacE	
		sul1	*sul1*
		sul1	*sul1*
	NODE_58_length_6207_cov_110.450000	*mph(A)*	*mph(A)*
	NODE_69_length_2598_cov_250.659247	*aph(3')-Ia*	*aph(3')-Ia*
	NODE_length_507980_cov_53.510563	*oqxA*	*oqxA*
		OqxA	
		oqxB	*oqxB*
		OqxB	
SRR8607464	NODE_40_length_20489_cov_15.371967	*bla*$_{KPC-2}$	*bla*$_{KPC-2}$
	NODE_43_length_16866_cov_17.739172	*aadA2*	*aadA2*
		catA1	*catA1*
		dfrA12	*dfrA12*
		mph(A)	*mph(A)*
		qacE	
		sul1	*sul1*
	NODE_46_length_13275_cov_425.376103	*aac(6')-Ib*	*aac(6')-Ib-AKT*
		aac(6')-Ib-cr	
	NODE_5_length_270780_cov_33.947202	*fosA*	*fosA6*
	NODE_68_length_2510_cov_50.363407	*bla*$_{SHV-12}$	*bla*$_{SHV-12}$
	NODE_79_length_1443_cov_13.072188	*bla*$_{OXA-9}$	*bla*$_{OXA-9}$
	NODE_84_length_1292_cov_25.979399	*aph(3')-Ia*	*aph(3')-Ia*
	NODE_length_798725_cov_33.563121	*oqxA*	*oqxA*
		OqxA	
		oqxB	*oqxB*
		OqxB	
SRR8607465	NODE_14_length_15129cov_36.359755	*bla*$_{SHV-11}$	*bla*$_{SHV-11}$
		bla$_{SHV-13}$	
		bla$_{SHV-70}$	
	NODE_27_length_59244_cov_28.073177	*aadA2*	*aadA2*
		catA1	*catA1*
		dfrA12	*dfrA12*
		mph(A)	*mph(A)*
		qacE	
		sul1	*sul1*
	NODE_44_length_2038cov_18.620865	*bla*$_{KPC-2}$	*bla*$_{KPC-2}$
	NODE_49_length_13275_cov_503.226270	*aac(6')-Ib*	*aac(6')-Ib-AKT*
		aac(6')-Ib-cr	
	NODE_6_length_270452_cov_40.093610	*fosA*	*fosA6*
	NODE_89_length_1292_cov_22.902146	*aph(3')-Ia*	*aph(3')-Ia*
	NODE_8length_1443_cov_20.472644	*bla*$_{OXA-9}$	*bla*$_{OXA-9}$

	NODE_96_length_923_cov_16.026382	*bla*$_{TEM-1A}$	*bla*$_{TEM-1}$
	NODE_length_486367_cov_38.293125	*oqxA*	*oqxA*
		OqxA	
		oqxB	*oqxB*
		OqxB	
SRR8607466	NODE_12_length_15769cov_31.007134	*bla*$_{SHV-12}$	*bla*$_{SHV-12}$
	NODE_29_length_35933_cov_36.639669	*bla*$_{KPC-2}$	*bla*$_{KPC-2}$
	NODE_38_length_13275_cov_526.294493	*aac(6')-Ib*	*aac(6')-Ib-AKT*
		aac(6')-Ib-cr	
	NODE_44_length_11160_cov_91.283604	*aadA2*	*aadA2*
		dfrA12	*dfrA12*
		mph(A)	*mph(A)*
		qacE	
		sul1	*sul1*
	NODE_58_length_1292_cov_100.821459	*aph(3')-Ia*	*aph(3')-Ia*
	NODE_6_length_270470_cov_33.383964	*fosA*	*fosA6*
	NODE_length_805282_cov_33.267280	*oqxA*	*oqxA*
		OqxA	
		oqxB	*oqxB*
		OqxB	
SRR8607467	NODE_13_length_15769cov_57.844044	*bla*$_{SHV-12}$	*bla*$_{SHV-12}$
	NODE_2_length_47287cov_63.799225	*OqxA*	
		oqxA	*oqxA*
		OqxB	
		oqxB	*oqxB*
	NODE_24_length_67829_cov_109.579939	*bla*$_{KPC-2}$	*bla*$_{KPC-2}$
	NODE_26_length_47909_cov_92.121343	*aph(3'')-Ib*	
		aph(3'')-Ib	
		aph(6)-Id	*aph(6)-Id*
		bla$_{OXA-9}$	*bla*$_{OXA-9}$
		bla$_{TEM-1A}$	*bla*$_{TEM-1}$
		dfrA14	*dfrA14*
		sul2	*sul2*
		tet(A)	*tet(A)*
	NODE_47_length_2252_cov_648.507765	*aac(6')-Ib*	*aac(6')-Ib-AKT*
		aac(6')-Ib-cr	
	NODE_55_length_1292_cov_104.345923	*aph(3')-Ia*	*aph(3')-Ia*
	NODE_6_length_270452_cov_65.901593	*fosA*	*fosA6*
SRR8607468	NODE_13_length_150854_cov_26.671658	*bla*$_{SHV-12}$	*bla*$_{SHV-187}$
		bla$_{SHV-182}$	
	NODE_20_length_93535_cov_46.053711	*aph(3'')-Ib*	
		aph(3'')-Ib	

		aph(6)-Id	*aph(6)-Id*
		aph(6)-Id	
		aph(6)-Id	
		bla$_{\text{TEM-1D}}$	*bla*$_{\text{TEM-12}}$
		dfrA14	*dfrA14*
		sul2	*sul2*
	NODE_22_length_7396cov_23.445594	*bla*$_{\text{KPC-2}}$	*bla*$_{\text{KPC-2}}$
		mph(A)	*mph(A)*
	NODE_5_length_270452_cov_28.124262	*fosA*	*fosA6*
	NODE_64_length_1292_cov_21.625751	*aph(3')-Ia*	*aph(3')-Ia*
	NODE_68_length_1128_cov_403.545455	*aac(6')-Ib*	*aac(6')-Ib-AKT*
		aac(6')-Ib-cr	
	NODE_length_803719_cov_28.354903	*oqxA*	*oqxA*
		OqxA	
		oqxB	*oqxB*
		OqxB	
SRR8607470	NODE_169_length_487_cov_3.369444	*aadA1*	
		aadA1	
		aadA24	
	NODE_183_length_445_cov_2.176101	*ant(2")-Ia*	
		ant(2")-Ia	
		ant(2")-Ia	
	NODE_26_length_59380_cov_40.087709	*aadA2*	*aadA2*
		catA1	*catA1*
		dfrA12	*dfrA12*
		mph(A)	*mph(A)*
		qacE	
		sul1	*sul1*
	NODE_39_length_20517_cov_39.219078	*bla*$_{\text{KPC-2}}$	*bla*$_{\text{KPC-2}}$
	NODE_44_length_13275_cov_522.760116	*aac(6')-Ib*	*aac(6')-Ib-AKT*
		aac(6')-Ib-cr	
	NODE_5_length_270452_cov_42.457531	*fosA*	*fosA6*
	NODE_59_length_2716_cov_72.443414	*bla*$_{\text{SHV-12}}$	*bla*$_{\text{SHV-12}}$
	NODE_73_length_1443_cov_48.159574	*bla*$_{\text{OXA-9}}$	*bla*$_{\text{OXA-9}}$
	NODE_76_length_1329_cov_1.990017	*dfrA23*	*dfrA23*
	NODE_79_length_1292_cov_34.573391	*aph(3')-Ia*	*aph(3')-Ia*
	NODE_94_length_923_cov_41.742462	*bla*$_{\text{TEM-1A}}$	*bla*$_{\text{TEM-1}}$
	NODE_length_804289_cov_43.152229	*OqxA*	
		oqxA	*oqxA*
		OqxB	
		oqxB	*oqxB*
	NODE_109_length_1443_cov_22.472644	*bla*$_{\text{OXA-9}}$	*bla*$_{\text{OXA-9}}$

	NODE_115_length_129cov_18.677835	*aph(3')-Ia*	*aph(3')-Ia*
	NODE_119_length_1186_cov_19.629839	*dfrA14*	*dfrA14*
	NODE_125_length_98cov_40.139344	*qacE*	
		sul1	
	NODE_127_length_923_cov_36.650754	*bla*$_{\text{TEM-104}}$	*bla*$_{\text{TEM-1}}$
		bla$_{\text{TEM-1B}}$	
		bla$_{\text{TEM-79}}$	
	NODE_15_length_129227_cov_23.121022	*bla*$_{\text{SHV-182}}$	*bla*$_{\text{SHV-158}}$
	NODE_16_length_125336_cov_20.485013	*aph(3'')-Ib*	*aph(3'')-Ib*
		aph(6)-Id	*aph(6)-Id*
		sul2	*sul2*
		tet(A)	*tet(A)*
	NODE_17_length_123293_cov_24.408879	*OqxA*	
		oqxA	*oqxA*
		OqxB	
		oqxB	*oqxB*
SRR8607471	NODE_4_length_270452_cov_25.434070	*fosA*	*fosA6*
	NODE_50_length_20517_cov_21.710741	*bla*$_{\text{KPC-2}}$	*bla*$_{\text{KPC-2}}$
	NODE_55_length_16907_cov_23.497259	*catA1*	*catA1*
	NODE_70_length_10034_cov_16.326537	*rmtB*	*rmtB1*
		tet(G)	*tet(G)*
			floR2
	NODE_77_length_6937_cov_17.662261	*dfrA23*	*dfrA23*
	NODE_82_length_5056_cov_21.892270	*aadA1*	*aadA1*
		ant(2'')-Ia	*ant(2'')-Ia*
		ARR-2	*arr-2*
		bla$_{\text{OXA-10}}$	*bla*$_{\text{OXA-10}}$
		cmlA1	*cmlA5*
	NODE_88_length_3484_cov_19.868037	*mph(A)*	*mph(A)*
	NODE_96_length_2242_cov_27.515839	*aadA2*	*aadA2*
		dfrA12	*dfrA12*
	NODE_99_length_2045_cov_220.034411	*aac(6')-Ib*	*aac(6')-Ib-AKT*
		aac(6')-Ib-cr	
	NODE_100_length_2158_cov_12.152634	*bla*$_{\text{CTX-M-15}}$	*bla*$_{\text{CTX-M-15}}$
	NODE_104_length_1860_cov_5.881708	*bla*$_{\text{OXA-1}}$	*bla*$_{\text{OXA-1}}$
		catB3	
		catB3	
SRR3242012	NODE_105_length_1767_cov_6.504268	*catA1*	*catA1*
	NODE_106_length_1737_cov_5.043478	*bla*$_{\text{TEM-1A}}$	*bla*$_{\text{TEM-150}}$
	NODE_109_length_1616_cov_7.673606	*aph(3')-VI*	*aph(3')-VI*
	NODE_11length_133cov_7.569767	*bla*$_{\text{SHV-11}}$	*bla*$_{\text{SHV-187}}$
		bla$_{\text{SHV-13}}$	

		bla$_{SHV-185}$	
		bla$_{SHV-25}$	
		bla$_{SHV-31}$	
		bla$_{SHV-69}$	
		bla$_{SHV-70}$	
	NODE_2_length_277705_cov_6.043768	*fosA*	*fosA6*
	NODE_45_length_41865_cov_6.460779	*dfrA1*	*dfrA1*
			sat2_gen
	NODE_6_length_18768cov_6.148741	*oqxA*	*oqxA6*
		OqxA	
		oqxB	*oqxB20*
		OqxB	
	NODE_62_length_24226_cov_9.405867	*bla*$_{OXA-232}$	*bla*$_{OXA-232}$
	NODE_6length_24365_cov_7.092004	*aadA2*	*aadA2*
		armA	*armA*
		dfrA12	*dfrA12*
		mph(E)	*mph(E)*
		msr(E)	*msr(E)*
		qacE	
		sul1	*sul1*
	NODE_82_length_9433_cov_6.602515	*bla*$_{NDM-1}$	*bla*$_{NDM-1}$
			ble-$_{MBL}$
	NODE_85_length_7483_cov_8.383089	*aac(3)-IId*	*aac(3)-IId*
	NODE_86_length_5368_cov_7.347644	*aph(3'')-Ib*	*aph(3'')-Ib*
		aph(6)-Id	*aph(6)-Id*
		sul2	*sul2*
	NODE_95_length_3047_cov_6.548630	*aac(6')-Ib-cr*	*aac(6')-Ib-AKT*
		aac(6')-Ib3	
		aadA1	*aadA1*
		bla$_{OXA-9}$	*bla*$_{OXA-9}$
SRR4025850	NODE_119_length_768_cov_71.989080	*bla*$_{SHV-101}$	
		bla$_{SHV-133}$	
		bla$_{SHV-148}$	
		bla$_{SHV-26}$	
		bla$_{SHV-46}$	
		bla$_{SHV-50}$	
		bla$_{SHV-98}$	
	NODE_27_length_59252_cov_25.277311	*fosA*	*fosA6*
	NODE_49_length_6205_cov_39.579961	*catA1*	*catA1*
	NODE_59_length_3777_cov_81.356712	*tet(D)*	*tet(D)*
	NODE_60_length_3484_cov_42.998511	*mph(A)*	*mph(A)*
	NODE_66_length_2433_cov_65.460104	*aadA2b*	*aadA2*

		qacE	
		sul1	*sul1*
	NODE_76_length_1824_cov_114.520919	*bla*$_{\text{TEM-1A}}$	*bla*$_{\text{TEM-150}}$
	NODE_90_length_1254_cov_204.976930	*aac(6')-Ib*	*aac(6')-Ib-AKT*
		aac(6')-Ib-cr	
	NODE_94_length_117cov_47.590996	*dfrA12*	*dfrA12*
	NODE_length_784484_cov_25.178651	*oqxA*	*oqxA*
		OqxA	
		oqxB	*oqxB*
		OqxB	
SRR4025851	NODE_24_length_43980_cov_31.994048	*bla*$_{\text{CTX-M-15}}$	*bla*$_{\text{CTX-M-15}}$
	NODE_25_length_41904_cov_44.375637	*bla*$_{\text{CMY-6}}$	*bla*$_{\text{CMY-6}}$
	NODE_28_length_27939_cov_40.637279	*tet(A)*	*tet(A)*
	NODE_32_length_15874_cov_45.912237	*bla*$_{\text{NDM-1}}$	*bla*$_{\text{NDM-1}}$
			ble-$_{\text{MBL}}$
	NODE_39_length_5620_cov_55.522665	*qnrB9*	*qnrB9*
	NODE_3length_18255_cov_57.308970	*aph(3'')-Ib*	*aph(3'')-Ib*
		aph(6)-Id	*aph(6)-Id*
		bla$_{\text{TEM-1B}}$	*bla*$_{\text{TEM-1}}$
		sul2	*sul2*
	NODE_44_length_411cov_40.500000	*rmtC*	*rmtC*
	NODE_47_length_2964_cov_41.202200	*mph(A)*	*mph(A)*
	NODE_49_length_2852_cov_62.001835	*aac(3)-IIa*	*aac(3)-IIe*
	NODE_57_length_1989_cov_75.938776	*dfrA14*	*dfrA14*
	NODE_59_length_1859_cov_54.642032	*bla*$_{\text{OXA-1}}$	*bla*$_{\text{OXA-1}}$
		catB3	
		catB3	
	NODE_62_length_1322_cov_78.403347	*qacE*	
		sul1	*sul1*
	NODE_68_length_1105_cov_25.515337	*aadA2b*	*aadA2*
	NODE_7_length_270412_cov_28.010456	*fosA*	*fosA6*
	NODE_74_length_817_cov_71.036232	*bla*$_{\text{SHV-155}}$	*bla*$_{\text{SHV-155}}$
	NODE_78_length_707_cov_109.667241	*aac(6')-Ib-cr*	*aac(6')-Ib-AKT*
		aac(6')-Ib-cr	
	NODE_length_839487_cov_27.557214	*OqxA*	
		oqxA	*oqxA*
		OqxB	
		oqxB	*oqxB*
SRR4025861	NODE_26_length_59600_cov_33.182772	*catA1*	*catA1*
	NODE_3_length_346135_cov_22.818377	*oqxA*	*oqxA*
		OqxA	
		oqxB	*oqxB*

		OqxB	
	NODE_36_length_23850_cov_57.287316	*aac(3)-IV*	*aac(3)-IVa*
		aph(4)-Ia	*aph(4)-Ia*
		bla$_{KPC-2}$	*bla*$_{KPC-2}$
	NODE_5_length_270337_cov_23.793357	*fosA*	*fosA6*
	NODE_5length_6638_cov_85.337890	*bla*$_{TEM-1A}$	*bla*$_{TEM-1}$
	NODE_60_length_4145_cov_62.014186	*aadA1*	*aadA1*
		cmlA1	*cmlA1*
	NODE_62_length_3484_cov_31.155496	*mph(A)*	*mph(A)*
	NODE_64_length_3197_cov_53.654397	*sul3*	*sul3*
	NODE_6length_359cov_43.889723	*qacE*	
		sul1	*sul1*
	NODE_72_length_1443_cov_54.078267	*bla*$_{OXA-9}$	*bla*$_{OXA-9}$
	NODE_74_length_1272_cov_33.731004	*dfrA12*	*dfrA12*
	NODE_84_length_899_cov_245.045337	*aac(6')-Ib*	*aac(6')-Ib-AKT*
		aac(6')-Ib-cr	
	NODE_87_length_84cov_114.347339	*aadA2b*	*aadA2*
	NODE_90_length_722_cov_60.242017	*bla*$_{SHV-12}$	*bla*$_{SHV-187}$
		bla$_{SHV-129}$	
		bla$_{SHV-13}$	
		bla$_{SHV-155}$	
		bla$_{SHV-172}$	
		bla$_{SHV-31}$	
SRR4025863	NODE_24_length_47178_cov_48.673673	*rmtG*	*rmtG*
		sul2	*sul2*
	NODE_37_length_12866_cov_43.874715	*bla*$_{TEM-1A}$	*bla*$_{TEM-150}$
	NODE_47_length_4348_cov_54.901445	*ARR-2*	*arr-2*
		bla$_{OXA-10}$	*bla*$_{OXA-10}$
		cmlA1	*cmlA5*
	NODE_48_length_3255_cov_73.599744	*sul1*	
		sul1	
		sul1	
		sul1	
		sul1	
		sul1	
		sul1	
	NODE_57_length_1987_cov_41.222581	*bla*$_{CTX-M-2}$	*bla*$_{CTX-M-2}$
		bla$_{CTX-M-97}$	
	NODE_64_length_1442_cov_50.007605	*bla*$_{OXA-9}$	*bla*$_{OXA-9}$
	NODE_6length_1648_cov_43.558185	*catA2*	*catA2*
	NODE_7_length_263213_cov_37.943095	*fosA*	*fosA6*

	NODE_76_length_92cov_38.250630	*aac(6')-Ib*	*aac(6')-Ib-AGKT*
		aac(6')-Ib-cr	
	NODE_84_length_672_cov_128.027523	*aadA1*	*aadA1*
		aadA1	
		ant(3'')-Ia	
	NODE_8length_723_cov_136.817114	*qacE*	
	NODE_length_626658_cov_30.982164	*bla*$_{SHV-187}$	*bla*$_{SHV-187}$
SRR4025977	NODE_13_length_15769cov_24.937936	*bla*$_{SHV-182}$	*bla*$_{SHV-158}$
	NODE_26_length_3331cov_42.158992	*catA1*	*catA1*
	NODE_28_length_3181cov_96.557568	*bla*$_{TEM-150}$	*bla*$_{TEM-150}$
		bla$_{TEM-1A}$	
	NODE_32_length_1906cov_328.798141	*bla*$_{KPC-3}$	*bla*$_{KPC-3}$
	NODE_39_length_7825_cov_42.446350	*mph(A)*	*mph(A)*
		qacE	
		sul1	*sul1*
	NODE_47_length_4569_cov_80.773075	*aac(3)-IV*	*aac(3)-IVa*
		aph(4)-Ia	*aph(4)-Ia*
	NODE_4length_7094_cov_72.572700	*aadA1*	*aadA1*
		cmlA1	*cmlA1*
		sul3	*sul3*
	NODE_59_length_1293_cov_41.562607	*aph(3')-Ia*	*aph(3')-Ia*
	NODE_62_length_1272_cov_45.980786	*dfrA12*	*dfrA12*
	NODE_7_length_270452_cov_30.668166	*fosA*	*fosA6*
	NODE_70_length_899_cov_377.449482	*aac(6')-Ib*	*aac(6')-Ib-AKT*
		aac(6')-Ib-cr	
	NODE_78_length_642_cov_116.433010	*aadA2*	
	NODE_length_839819_cov_30.252035	*oqxA*	*oqxA*
		OqxA	
		oqxB	*oqxB*
		OqxB	
SRR4025979	NODE_20_length_85380_cov_18.113204	*OqxA*	
		oqxA	*oqxA*
		OqxB	
		oqxB	*oqxB*
	NODE_26_length_48844_cov_25.663875	*catA1*	*catA1*
	NODE_32_length_21958_cov_156.775823	*bla*$_{KPC-3}$	*bla*$_{KPC-3}$
		bla$_{TEM-1A}$	*bla*$_{TEM-150}$
	NODE_33_length_17825_cov_36.790598	*aac(3)-IV*	*aac(3)-IVa*
		aph(4)-Ia	*aph(4)-Ia*
	NODE_38_length_7095_cov_34.292623	*aadA1*	*aadA1*
		cmlA1	*cmlA1*

		sul3	*sul3*
	NODE_45_length_359cov_32.766455	*qacE*	
		sul1	*sul1*
	NODE_46_length_3484_cov_24.050343	*mph(A)*	*mph(A)*
	NODE_53_length_1292_cov_23.051502	*aph(3')-Ia*	*aph(3')-Ia*
	NODE_55_length_1272_cov_25.344105	*dfrA12*	*dfrA12*
	NODE_6_length_270452_cov_18.821200	*fosA*	*fosA6*
	NODE_67_length_722_cov_53.709244	*bla*$_{\text{SHV-12}}$	*bla*$_{\text{SHV-187}}$
		bla$_{\text{SHV-129}}$	
		bla$_{\text{SHV-13}}$	
		bla$_{\text{SHV-155}}$	
		bla$_{\text{SHV-172}}$	
		bla$_{\text{SHV-31}}$	
	NODE_73_length_642_cov_75.677670	*aadA2*	
	NODE_78_length_544_cov_2.158273	*aac(6')-Ib*	
		aac(6')-Ib-cr	
		aac(6')-Ib-cr	
SRR4025980	NODE_27_length_71027_cov_77.890071	*bla*$_{\text{TEM-1A}}$	*bla*$_{\text{TEM-150}}$
	NODE_35_length_3586cov_29.373510	*OqxA*	
		oqxA	*oqxA10*
		OqxB	
		oqxB	*oqxB4*
	NODE_4_length_313160_cov_30.656337	*fosA*	*fosA6*
	NODE_44_length_19568_cov_191.679955	*dfrA14*	*dfrA14*
	NODE_53_length_10513_cov_223.626998	*bla*$_{\text{KPC-3}}$	*bla*$_{\text{KPC-3}}$
	NODE_69_length_3483_cov_45.971990	*ant(2'')-Ia*	*ant(2'')-Ia*
		catB3	*catB3*
		qacE	
		sul1	*sul1*
	NODE_70_length_285cov_46.475771	*dfrA19*	*dfrA19*
	NODE_72_length_1907_cov_82.748876	*aadA1*	*aadA1*
		bla$_{\text{OXA-9}}$	*bla*$_{\text{OXA-9}}$
	NODE_84_length_1076_cov_53.327713	*bla*$_{\text{SHV-1}}$	*bla*$_{\text{SHV-187}}$
		bla$_{\text{SHV-102}}$	
		bla$_{\text{SHV-120}}$	
		bla$_{\text{SHV-153}}$	
		bla$_{\text{SHV-2}}$	
		bla$_{\text{SHV-5}}$	
	NODE_96_length_753_cov_302.071885	*aac(6')-Ib*	*aac(6')-Ib-AKT*
		aac(6')-Ib-cr	
		aac(6')-Ib-cr	
	NODE_37_length_25616_cov_31.308329	*floR*	*floR*

SRR4025983		*sul2*	*sul2*
	NODE_46_length_6210_cov_21.497123	*mph(A)*	*mph(A)*
	NODE_50_length_4386_cov_29.828363	*bla*$_{KPC-2}$	*bla*$_{KPC-2}$
	NODE_55_length_2009_cov_20.598831	*aadA2*	*aadA2*
		dfrA12	*dfrA12*
	NODE_6_length_270412_cov_16.570583	*fosA*	*fosA6*
	NODE_60_length_1595_cov_51.113079	*qacE*	
		sul1	*sul1*
	NODE_6length_1566_cov_32.459347	*aac(6')-33*	*aac(6')-33*
		ant(2'')-Ia	*ant(2'')-Ia*
	NODE_70_length_1293_cov_21.373928	*aph(3')-Ia*	*aph(3')-Ia*
	NODE_7length_1255_cov_145.617908	*aac(6')-Ib*	*aac(6')-Ib-AKT*
		aac(6')-Ib-cr	
	NODE_80_length_1080_cov_26.718783	*bla*$_{TEM-1D}$	*bla*$_{TEM-12}$
	NODE_8length_817_cov_48.085507	*bla*$_{SHV-155}$	*bla*$_{SHV-155}$
	NODE_length_621394_cov_16.432560	*oqxA*	*oqxA*
		OqxA	
		oqxB	*oqxB*
		OqxB	
SRR4025984	NODE_25_length_47583_cov_33.900160	*catA1*	*catA1*
	NODE_37_length_10008_cov_279.804979	*bla*$_{KPC-3}$	*bla*$_{KPC-3}$
	NODE_38_length_9532_cov_42.503881	*bla*$_{TEM-150}$	
		bla$_{TEM-171}$	
		bla$_{TEM-1A}$	
		bla$_{TEM-1C}$	
		bla$_{TEM-40}$	
	NODE_40_length_7826_cov_34.605923	*mph(A)*	*mph(A)*
		qacE	
		sul1	*sul1*
	NODE_46_length_542cov_37.558557	*aac(3)-IV*	*aac(3)-IVa*
		aph(4)-Ia	*aph(4)-Ia*
	NODE_48_length_4855_cov_30.092428	*sul3*	*sul3*
	NODE_57_length_1799_cov_31.285885	*cmlA1*	*cmlA1*
	NODE_6_length_270452_cov_16.171790	*fosA*	*fosA6*
	NODE_66_length_1293_cov_38.414237	*aph(3')-Ia*	*aph(3')-Ia*
	NODE_68_length_1272_cov_33.041921	*dfrA12*	*dfrA12*
	NODE_6length_1442_cov_38.489734	*bla*$_{OXA-9}$	*bla*$_{OXA-9}$
	NODE_77_length_737_cov_194.901639	*aac(6')-Ib*	
		aac(6')-Ib-cr	
	NODE_79_length_722_cov_53.157983	*bla*$_{SHV-12}$	*bla*$_{SHV-187}$
		bla$_{SHV-129}$	
		bla$_{SHV-13}$	

		bla$_{\text{SHV-155}}$	
		bla$_{\text{SHV-172}}$	
		bla$_{\text{SHV-31}}$	
	NODE_83_length_642_cov_67.914563	*aadA2*	
		aadA2	
	NODE_87_length_592_cov_68.258065	*aadA24*	
	NODE_length_839786_cov_16.132113	*OqxA*	
		oqxA	*oqxA*
		OqxB	
		oqxB	*oqxB*
SRR4025985	NODE_23_length_58026_cov_17.505708	*OqxA*	
		oqxA	*oqxA11*
		OqxB	
		oqxB	*oqxB20*
	NODE_25_length_43748_cov_38.356915	*bla*$_{\text{KPC-2}}$	*bla*$_{\text{KPC-2}}$
		bla$_{\text{TEM-1B}}$	*bla*$_{\text{TEM-1}}$
	NODE_40_length_3626_cov_40.501286	*aac(6')-Ib-cr*	*aac(6')-Ib-D181Y*
		aac(6')-Ib-cr	
		ARR-3	*arr-3*
		bla$_{\text{OXA-1}}$	*bla*$_{\text{OXA-1}}$
		catB3	*catB3*
	NODE_44_length_1964_cov_24.726728	*aadA1*	*aadA1*
		dfrA1	*dfrA1*
		dfrA1	
		dfrA1	
	NODE_46_length_1594_cov_65.162236	*qacE*	
		sul1	*sul1*
	NODE_47_length_1564_cov_56.927627	*bla*$_{\text{SHV-141}}$	*bla*$_{\text{SHV-141}}$
		bla$_{\text{SHV-30}}$	
	NODE_8_length_262003_cov_18.794109	*fosA*	*fosA_gen*
SRR4025987	NODE_16_length_121233_cov_20.478663	*aadA2*	*aadA2*
		catA1	*catA1*
		dfrA12	*dfrA12*
		mph(A)	*mph(A)*
		qacE	
		sul1	*sul1*
	NODE_29_length_2978cov_33.261820	*bla*$_{\text{TEM-1A}}$	*bla*$_{\text{TEM-150}}$
	NODE_4_length_367443_cov_17.551786	*fosA*	*fosA6*
	NODE_40_length_1111cov_36.247997	*bla*$_{\text{KPC-3}}$	*bla*$_{\text{KPC-3}}$
	NODE_54_length_1293_cov_22.765009	*aph(3')-Ia*	*aph(3')-Ia*
	NODE_57_length_1128_cov_119.418581	*aac(6')-Ib*	*aac(6')-Ib-AKT*

		aac(6')-Ib-cr	
	NODE_59_length_103cov_42.515487	*bla*$_{\text{SHV-12}}$	
		bla$_{\text{SHV-15}}$	
		bla$_{\text{SHV-160}}$	
		bla$_{\text{SHV-66}}$	
	NODE_length_779309_cov_17.151639	*oqxA*	*oqxA*
		OqxA	
		oqxB	*oqxB*
		OqxB	
SRR4025990	NODE_25_length_595cov_25.511332	*aac(6')-Ib-cr*	*aac(6')-Ib-G*
		aac(6')-Ib-Hangzhou	*catB*
		rmtF	*rmtF1*
	NODE_27_length_5720_cov_39.426783	*bla*$_{\text{NDM-7}}$	*bla*$_{\text{NDM-7}}$
			ble-$_{\text{MBL}}$
	NODE_28_length_5673_cov_33.710783	*sul2*	*sul2*
	NODE_34_length_3635_cov_33.272805	*qacE*	
		sul1	*sul1*
	NODE_35_length_3485_cov_34.159321	*mph(A)*	*mph(A)*
	NODE_38_length_2988_cov_49.359664	*bla*$_{\text{CTX-M-15}}$	*bla*$_{\text{CTX-M-15}}$
	NODE_39_length_2909_cov_33.140906	*bla*$_{\text{TEM-1B}}$	*bla*$_{\text{TEM-1}}$
	NODE_40_length_2544_cov_43.473314	*aadA2*	*aadA2*
		dfrA12	*dfrA12*
	NODE_42_length_2209_cov_32.412104	*qnrB1*	*qnrB1*
	NODE_48_length_1649_cov_29.908016	*catA2*	*catA2*
	NODE_5_length_427634_cov_17.861024	*oqxA*	*oqxA6*
		OqxA	
		oqxB	*oqxB19*
		OqxB	
	NODE_55_length_1186_cov_33.898961	*dfrA14*	*dfrA14*
	NODE_5length_1478_cov_66.390081	*aph(3'')-Ib*	
		aph(3'')-Ib	
		aph(3'')-Ib	
		aph(6)-Id	*aph(6)-Id*
	NODE_63_length_749_cov_24.897106	*ARR-2*	*arr-2*
	NODE_9_length_159039_cov_18.519306	*fosA*	*fosA_gen*
	NODE_length_1544546_cov_14.981536	*bla*$_{\text{SHV-11}}$	*bla*$_{\text{SHV-11}}$
		bla$_{\text{SHV-67}}$	
SRR4025991	NODE_19_length_117416_cov_17.187801	*fosA*	*fosA6*
	NODE_1length_143279_cov_12.170574	*bla*$_{\text{SHV-110}}$	*bla*$_{\text{SHV-110}}$
		bla$_{\text{SHV-81}}$	
	NODE_3_length_356175_cov_16.441221	*OqxA*	

		oqxA	*oqxA*
		OqxB	
		oqxB	*oqxB*
	NODE_32_length_47763_cov_14.463179	*bla*$_{\text{CMY-4}}$	*bla*$_{\text{CMY-4}}$
	NODE_37_length_29788_cov_13.044537	*aph(3'')-Ib*	*aph(3'')-Ib*
		aph(6)-Id	*aph(6)-Id*
		sul2	*sul2*
	NODE_45_length_10770_cov_51.696796	*aph(3')-VI*	*aph(3')-VI*
		bla$_{\text{NDM-1}}$	*bla*$_{\text{NDM-1}}$
			ble-$_{\text{MBL}}$
	NODE_57_length_5142_cov_30.703490	*aadA1*	*aadA1*
		aadA1	
		ARR-2	*arr-2*
		bla$_{\text{OXA-10}}$	*bla*$_{\text{OXA-10}}$
		cmlA1	*cmlA5*
	NODE_59_length_473cov_22.779105	*armA*	*armA*
	NODE_62_length_413cov_13.708791	*aac(6')-IIa*	*aac(6')-IIa*
		aadA2	*aadA2*
		dfrA1	*dfrA1*
			catB11
	NODE_65_length_3814_cov_50.461079	*bla*$_{\text{CTX-M-15}}$	*bla*$_{\text{CTX-M-15}}$
	NODE_67_length_3470_cov_39.574634	*qacE*	
		sul1	*sul1*
	NODE_75_length_2057_cov_41.577202	*tet(A)*	*tet(A)*
	NODE_90_length_1254_cov_22.186335	*msr(E)*	*msr(E)*
	NODE_97_length_1066_cov_23.883919	*mph(E)*	*mph(E)*
SRR4025992	NODE_103_length_540_cov_83.983051	*bla*$_{\text{SHV-12}}$	
		bla$_{\text{SHV-129}}$	
		bla$_{\text{SHV-13}}$	
		bla$_{\text{SHV-142}}$	
		bla$_{\text{SHV-148}}$	
		bla$_{\text{SHV-155}}$	
		bla$_{\text{SHV-172}}$	
		bla$_{\text{SHV-185}}$	
		bla$_{\text{SHV-199}}$	
		bla$_{\text{SHV-31}}$	
		bla$_{\text{SHV-61}}$	
		bla$_{\text{SHV-86}}$	
		bla$_{\text{SHV-98}}$	
	NODE_29_length_41954_cov_44.610060	*dfrA1*	*dfrA1*
			sat2_gen
	NODE_3_length_350190_cov_40.825063	*fosA*	*fosA6*

	NODE_35_length_27134_cov_56.339838	bla_{CMY-4}	bla_{CMY-4}
	NODE_47_length_13156_cov_47.986799	*armA*	*armA*
		mph(E)	*mph(E)*
		msr(E)	*msr(E)*
	NODE_56_length_5367_cov_73.705153	*aph(3'')-Ib*	*aph(3'')-Ib*
		aph(6)-Id	*aph(6)-Id*
		sul2	*sul2*
	NODE_64_length_344cov_72.924562	*ARR-2*	*arr-2*
		ere(A)	*ere(A)*
	NODE_65_length_2935_cov_72.821225	*aac(3)-IId*	*aac(3)-IId*
	NODE_69_length_2158_cov_189.774495	$bla_{CTX-M-15}$	$bla_{CTX-M-15}$
	NODE_6length_4377_cov_56.887294	bla_{DHA-1}	
		bla_{DHA-24}	
		bla_{DHA-7}	ble_{-MBL}
		bla_{NDM-1}	bla_{NDM-1}
	NODE_7_length_252609_cov_40.536446	*OqxA*	
		oqxA	*oqxA6*
		OqxB	
		oqxB	*oqxB20*
	NODE_72_length_2057_cov_126.813472	*aac(6')-Ib*	*aac(6')-Ib-AKT*
		aac(6')-Ib-cr	
	NODE_74_length_1746_cov_48.634342	*catA1*	*catA1*
	NODE_78_length_1486_cov_140.567329	$bla_{TEM-104}$	
		$bla_{TEM-126}$	
		$bla_{TEM-148}$	
		$bla_{TEM-176}$	
		$bla_{TEM-198}$	
		bla_{TEM-1B}	
		$bla_{TEM-207}$	
		$bla_{TEM-217}$	
		$bla_{TEM-220}$	
		$bla_{TEM-230}$	
		$bla_{TEM-234}$	
		bla_{TEM-30}	
		bla_{TEM-70}	
	NODE_7length_2106_cov_86.551289	*cmlA1*	*cmlA5*
	NODE_80_length_1442_cov_73.636502	bla_{OXA-9}	bla_{OXA-9}
	NODE_87_length_1044_cov_133.580153	*qacE*	
		sul1	*sul1*
		sul1	
	NODE_98_length_672_cov_183.462385	*aadA1*	*aadA1*
		aadA1	

		ant(3'')-Ia	
SRR4025993	NODE_19_length_35965_cov_27.674228	*aac(6')-Ib-cr*	*aac(6')-Ib-D181Y*
		aac(6')-Ib-cr	
		ARR-3	*arr-3*
		bla$_{OXA-1}$	*bla*$_{OXA-1}$
		catB3	*catB3*
		qacE	
		sul1	*sul1*
	NODE_1length_159343_cov_18.119127	*fosA*	*fosA_gen*
	NODE_2_length_729498_cov_17.565345	*oqxA*	*oqxA6*
		OqxA	
		oqxB	*oqxB19*
		OqxB	
	NODE_23_length_23865_cov_36.063443	*bla*$_{TEM-1A}$	*bla*$_{TEM-150}$
	NODE_24_length_1703cov_26.779697	*aph(3')-VI*	*aph(3')-VI*
		bla$_{CTX-M-15}$	*bla*$_{CTX-M-15}$
		bla$_{NDM-1}$	*bla*$_{NDM-1}$
		qnrS1	*qnrS1*
			ble-$_{MBL}$
	NODE_32_length_2260_cov_37.005626	*aadA1*	*aadA1*
		bla$_{OXA-9}$	*bla*$_{OXA-9}$
	NODE_6_length_392046_cov_14.581888	*bla*$_{SHV-11}$	*bla*$_{SHV-11}$
		bla$_{SHV-67}$	
SRR4025994	NODE_2_length_489874_cov_17.107568	*OqxA*	
		oqxA	*oqxA6*
		OqxB	
		oqxB	*oqxB20*
	NODE_20_length_7023cov_30.023380	*aac(6')-Ib*	*aac(6')-Ib-AKT*
		aac(6')-Ib-cr	
	NODE_22_length_40298_cov_28.025939	*aph(3'')-Ib*	*aph(3'')-Ib*
		aph(6)-Id	*aph(6)-Id*
		sul2	*sul2*
	NODE_26_length_28263_cov_25.580004	*tet(A)*	*tet(A)*
	NODE_34_length_849cov_26.912363	*dfrA14*	*dfrA14*
	NODE_36_length_5620_cov_27.995813	*qnrB9*	*qnrB9*
	NODE_3length_1926cov_29.658357	*bla*$_{NDM-1}$	*bla*$_{NDM-1}$
			ble-$_{MBL}$
	NODE_4_length_367372_cov_17.243557	*fosA*	*fosA6*
	NODE_42_length_3485_cov_25.217094	*mph(A)*	*mph(A)*
	NODE_4length_3815_cov_28.319414	*bla*$_{CTX-M-15}$	*bla*$_{CTX-M-15}$
	NODE_5_length_344597_cov_16.249633	*bla*$_{SHV-182}$	*bla*$_{SHV-158}$

	NODE_50_length_1859_cov_27.253464	*bla*$_{OXA\text{-}1}$	*bla*$_{OXA\text{-}1}$
		catB3	
		catB3	
	NODE_52_length_1649_cov_24.383049	*catA2*	*catA2*
SRR4025996	NODE_2_length_803396_cov_42.032694	*OqxA*	
		oqxA	*oqxA6*
		OqxB	
		oqxB	*oqxB20*
	NODE_34_length_19609_cov_74.438097	*bla*$_{CMY\text{-}6}$	*bla*$_{CMY\text{-}6}$
	NODE_35_length_18255_cov_113.744153	*aph(3'')-Ib*	*aph(3'')-Ib*
		aph(6)-Id	*aph(6)-Id*
		bla$_{TEM\text{-}1B}$	*bla*$_{TEM\text{-}1}$
		sul2	*sul2*
	NODE_37_length_12864_cov_73.110858	*aac(3)-IId*	*aac(3)-IId*
	NODE_4_length_367372_cov_43.136364	*fosA*	*fosA6*
	NODE_42_length_5618_cov_68.960299	*qnrB9*	*qnrB9*
	NODE_44_length_4680_cov_53.987700	*tet(A)*	*tet(A)*
	NODE_45_length_4659_cov_122.963813	*bla*$_{CTX\text{-}M\text{-}15}$	*bla*$_{CTX\text{-}M\text{-}15}$
	NODE_48_length_4247_cov_72.495631	*bla*$_{DHA\text{-}1}$	
		bla$_{DHA\text{-}24}$	
		bla$_{DHA\text{-}7}$	*ble*-$_{MBL}$
		bla$_{NDM\text{-}1}$	*bla*$_{NDM\text{-}1}$
	NODE_53_length_3879_cov_108.194563	*bla*$_{SHV\text{-}182}$	*bla*$_{SHV\text{-}158}$
	NODE_54_length_3485_cov_54.695354	*mph(A)*	*mph(A)*
	NODE_56_length_2589_cov_119.053209	*aac(3)-IIa*	
		aac(3)-IIa	*aac(3)-IIe*
	NODE_58_length_2439_cov_130.377595	*aac(6')-Ib-cr*	*aac(6')-Ib-D181Y*
		aac(6')-Ib-cr	
		bla$_{OXA\text{-}1}$	*bla*$_{OXA\text{-}1}$
		catB3	
		catB3	
	NODE_67_length_1545_cov_130.184767	*dfrA14*	*dfrA14*
	NODE_76_length_1157_cov_62.524272	*rmtC*	*rmtC*
	NODE_85_length_785_cov_151.984802	*qacE*	
		sul1	
SRR4026000	NODE_12_length_159039_cov_38.906785	*fosA*	*fosA_gen*
	NODE_26_length_23865_cov_72.674530	*bla*$_{TEM\text{-}1A}$	*bla*$_{TEM\text{-}150}$
	NODE_29_length_6944_cov_50.831744	*aph(3')-VI*	*aph(3')-VI*
		bla$_{NDM\text{-}1}$	*bla*$_{NDM\text{-}1}$
			ble-$_{MBL}$
	NODE_30_length_5668_cov_106.999459	*qnrS1*	*qnrS1*

	NODE_33_length_5030_cov_54.725474	*sul2*	*sul2*
	NODE_34_length_4673_cov_57.510559	*bla*$_{CTX-M-15}$	*bla*$_{CTX-M-15}$
	NODE_37_length_4007_cov_55.484021	*tet(A)*	*tet(A)*
	NODE_3length_5546_cov_59.012548	*aac(6')-Ib-cr*	*aac(6')-Ib-D181Y*
		aac(6')-Ib-cr	
		ARR-3	*arr-3*
		bla$_{OXA-1}$	*bla*$_{OXA-1}$
		catB3	*catB3*
		qacE	
		sul1	*sul1*
	NODE_4_length_431684_cov_37.061403	*OqxA*	
		oqxA	*oqxA6*
		OqxB	
		oqxB	*oqxB19*
	NODE_43_length_2438_cov_56.637386	*bla*$_{LAP-2}$	*bla*$_{LAP-2}$
	NODE_45_length_2260_cov_77.252227	*aadA1*	*aadA1*
		bla$_{OXA-9}$	*bla*$_{OXA-9}$
	NODE_4length_2786_cov_55.619782	*floR*	*floR*
	NODE_54_length_927_cov_51.756250	*dfrA14*	*dfrA14*
	NODE_7_length_392046_cov_31.086309	*bla*$_{SHV-11}$	*bla*$_{SHV-11}$
		bla$_{SHV-67}$	
SRR4026003	NODE_15_length_115757_cov_17.881225	*fosA*	*fosA_gen*
	NODE_16_length_113612_cov_17.329770	*bla*$_{SHV-12}$	*bla*$_{SHV-12}$
	NODE_2_length_448237_cov_18.106585	*oqxA*	*oqxA6*
		OqxA	
		oqxB	*oqxB24*
		OqxB	
	NODE_29_length_33365_cov_23.621578	*bla*$_{NDM-1}$	*bla*$_{NDM-1}$
			ble-$_{MBL}$
	NODE_40_length_14782_cov_35.331082	*aac(3)-IId*	*aac(3)-IId*
		qnrB1	*qnrB1*
	NODE_44_length_1233cov_10.146919	*aph(3'')-Ib*	*aph(3'')-Ib*
		aph(6)-Id	*aph(6)-Id*
		bla$_{CTX-M-15}$	*bla*$_{CTX-M-15}$
		bla$_{TEM-1B}$	*bla*$_{TEM-1}$
		sul2	*sul2*
	NODE_46_length_899cov_25.432536	*tet(B)*	*tet(B)*
	NODE_53_length_6925_cov_22.847014	*dfrA30*	*dfrA30*
		qacE	
	NODE_56_length_5575_cov_10.091593	*dfrA14*	*dfrA14*
	NODE_74_length_2439_cov_20.826990	*aac(6')-Ib-cr*	*aac(6')-Ib-D181Y*

		aac(6')-Ib-cr	
		bla$_{OXA-1}$	*bla*$_{OXA-1}$
		catB3	
		catB3	
SRR5122322	NODE_16_length_110250_cov_55.871199	*catA1*	*catA1*
	NODE_25_length_32146_cov_99.014772	*bla*$_{TEM-1A}$	*bla*$_{TEM-150}$
	NODE_30_length_1906cov_275.748072	*bla*$_{KPC-3}$	*bla*$_{KPC-3}$
	NODE_37_length_7825_cov_55.344895	*mph(A)*	*mph(A)*
		qacE	
		sul1	*sul1*
	NODE_39_length_7094_cov_74.403761	*aadA1*	*aadA1*
		cmlA1	*cmlA1*
		sul3	*sul3*
	NODE_46_length_4569_cov_78.014183	*aac(3)-IV*	*aac(3)-IVa*
		aph(4)-Ia	*aph(4)-Ia*
	NODE_59_length_1293_cov_62.939966	*aph(3')-Ia*	*aph(3')-Ia*
	NODE_60_length_1272_cov_61.571179	*dfrA12*	*dfrA12*
	NODE_65_length_1027_cov_341.018889	*aac(6')-Ib*	*aac(6')-Ib-AKT*
		aac(6')-Ib-cr	
	NODE_67_length_967_cov_114.852381	*bla*$_{SHV-12}$	
		bla$_{SHV-15}$	
		bla$_{SHV-160}$	
		bla$_{SHV-66}$	
	NODE_7_length_270452_cov_50.659258	*fosA*	*fosA6*
	NODE_76_length_642_cov_140.143689	*aadA2*	
	NODE_length_839818_cov_50.148823	*oqxA*	*oqxA*
		OqxA	
		oqxB	*oqxB*
		OqxB	
SRR5146462	NODE_15_length_117559_cov_35.648435	*bla*$_{SHV-11}$	*bla*$_{SHV-187}$
		bla$_{SHV-13}$	
		bla$_{SHV-185}$	
		bla$_{SHV-25}$	
		bla$_{SHV-31}$	
		bla$_{SHV-69}$	
		bla$_{SHV-70}$	
	NODE_30_length_43980_cov_53.614029	*bla*$_{CTX-M-15}$	*bla*$_{CTX-M-15}$
	NODE_35_length_27939_cov_47.986553	*tet(A)*	*tet(A)*
	NODE_36_length_18255_cov_55.768590	*aph(3'')-Ib*	*aph(3'')-Ib*
		aph(6)-Id	*aph(6)-Id*
		bla$_{TEM-1B}$	*bla*$_{TEM-1}$
		sul2	*sul2*

	NODE_39_length_15874_cov_55.491205	$bla_{\text{NDM-1}}$	$bla_{\text{NDM-1}}$
			ble-$_{\text{MBL}}$
	NODE_3length_41904_cov_71.473490	$bla_{\text{CMY-6}}$	$bla_{\text{CMY-6}}$
	NODE_55_length_411cov_46.008283	*rmtC*	*rmtC*
	NODE_5length_5620_cov_58.210450	*qnrB9*	*qnrB9*
	NODE_60_length_2964_cov_45.942413	*mph(A)*	*mph(A)*
	NODE_6length_2852_cov_73.845505	*aac(3)-IIa*	*aac(3)-IIe*
	NODE_7_length_270452_cov_45.218859	*fosA*	*fosA6*
	NODE_72_length_1859_cov_35.227483	$bla_{\text{OXA-1}}$	$bla_{\text{OXA-1}}$
		catB3	
		catB3	
	NODE_75_length_1322_cov_78.128033	*qacE*	
		sul1	*sul1*
	NODE_78_length_1258_cov_42.508400	*aadA2b*	*aadA2*
	NODE_80_length_1189_cov_74.878531	*dfrA14*	*dfrA14*
	NODE_92_length_707_cov_142.413793	*aac(6')-Ib-cr*	*aac(6')-Ib-AKT*
		aac(6')-Ib-cr	
	NODE_length_520407_cov_43.002366	*OqxA*	
		oqxA	*oqxA*
		OqxB	
		oqxB	*oqxB*
SRR5146463	NODE_14_length_117559_cov_39.826606	$bla_{\text{SHV-182}}$	$bla_{\text{SHV-187}}$
	NODE_2_length_51014cov_47.205375	*oqxA*	*oqxA*
		OqxA	
		oqxB	*oqxB*
		OqxB	
	NODE_29_length_43980_cov_57.886370	$bla_{\text{CTX-M-15}}$	$bla_{\text{CTX-M-15}}$
	NODE_30_length_41904_cov_78.814587	$bla_{\text{CMY-6}}$	$bla_{\text{CMY-6}}$
	NODE_33_length_27939_cov_53.492845	*tet(A)*	*tet(A)*
	NODE_35_length_18255_cov_61.305991	*aph(3'')-Ib*	*aph(3'')-Ib*
		aph(6)-Id	*aph(6)-Id*
		$bla_{\text{TEM-1B}}$	$bla_{\text{TEM-1}}$
		sul2	*sul2*
	NODE_38_length_15874_cov_60.267416	$bla_{\text{NDM-1}}$	$bla_{\text{NDM-1}}$
			ble-$_{\text{MBL}}$
	NODE_50_length_5620_cov_65.658292	*qnrB9*	*qnrB9*
	NODE_55_length_411cov_52.199799	*rmtC*	*rmtC*
	NODE_60_length_2964_cov_50.633128	*mph(A)*	*mph(A)*
	NODE_64_length_2439_cov_88.781142	*aac(6')-Ib-cr*	*aac(6')-Ib-D181Y*
		aac(6')-Ib-cr	
		$bla_{\text{OXA-1}}$	$bla_{\text{OXA-1}}$

		catB3	
		catB3	
	NODE_6length_2850_cov_78.051414	*aac(3)-IIa*	*aac(3)-IIe*
	NODE_7_length_270452_cov_49.379998	*fosA*	*fosA6*
	NODE_74_length_1423_cov_87.961420	*dfrA14*	*dfrA14*
	NODE_75_length_1322_cov_74.568201	*qacE*	
		sul1	*sul1*
	NODE_83_length_1105_cov_46.012270	*aadA2b*	*aadA2*
SRR5167852	NODE_108_length_1262_cov_138.534802	$bla_{\text{SHV-5}}$	$bla_{\text{SHV-165}}$
	NODE_109_length_1254_cov_54.817214	*aac(6')-Ib*	*aac(6')-Ib-AKT*
		aac(6')-Ib-cr	
	NODE_114_length_105cov_94.159091	$bla_{\text{TEM-1A}}$	$bla_{\text{TEM-150}}$
	NODE_29_length_59252_cov_28.754486	*fosA*	*fosA6*
	NODE_60_length_6205_cov_47.082264	*catA1*	*catA1*
	NODE_68_length_3779_cov_137.316539	*tet(D)*	*tet(D)*
	NODE_69_length_3732_cov_61.553953	*mph(A)*	*mph(A)*
	NODE_83_length_2234_cov_53.330802	*aadA2*	
		qacE	
		sul1	*sul1*
	NODE_95_length_1605_cov_47.960758	*dfrA12*	*dfrA12*
	NODE_length_465387_cov_28.157815	*OqxA*	
		oqxA	*oqxA*
		OqxB	
		oqxB	*oqxB*
SRR5167853	NODE_109_length_1370_cov_24.946098	*dfrA12*	*dfrA12*
	NODE_118_length_1126_cov_29.092092	*aac(6')-Ib*	*aac(6')-Ib-AKT*
		aac(6')-Ib-cr	
	NODE_13length_923_cov_62.226131	$bla_{\text{TEM-1A}}$	$bla_{\text{TEM-150}}$
	NODE_14length_768_cov_31.340094	$bla_{\text{SHV-1}}$	
		$bla_{\text{SHV-102}}$	
		$bla_{\text{SHV-120}}$	
		$bla_{\text{SHV-153}}$	
		$bla_{\text{SHV-2}}$	
		$bla_{\text{SHV-3}}$	
		$bla_{\text{SHV-44}}$	
		$bla_{\text{SHV-5}}$	
	NODE_29_length_59252_cov_13.754452	*fosA*	*fosA6*
	NODE_60_length_6205_cov_20.809477	*catA1*	*catA1*
	NODE_73_length_3777_cov_65.511781	*tet(D)*	*tet(D)*
	NODE_74_length_3732_cov_30.206380	*mph(A)*	*mph(A)*
	NODE_8_length_233042_cov_13.022798	*OqxA*	
		oqxA	*oqxA*

		OqxB	
		oqxB	*oqxB*
	NODE_90_length_2234_cov_28.341718	*aadA2*	
		qacE	
		sul1	*sul1*
SRR5168221	NODE_1length_163039_cov_30.109004	*bla*$_{SHV-106}$	*bla*$_{SHV-106}$
		bla$_{SHV-28}$	
	NODE_29_length_41952_cov_49.717442	*dfrA1*	*dfrA1*
			sat2_gen
	NODE_3_length_444346_cov_37.061060	*OqxA*	
		oqxA	*oqxA6*
		OqxB	
		oqxB	*oqxB20*
	NODE_39_length_24370_cov_46.653549	*aadA2*	*aadA2*
		armA	*armA*
		dfrA12	*dfrA12*
		mph(E)	*mph(E)*
		msr(E)	*msr(E)*
		qacE	
		sul1	*sul1*
	NODE_42_length_20573_cov_56.125404	*aac(6')-Ib*	*aac(6')-Ib-AKT*
		aac(6')-Ib-cr	
		aadA1	*aadA1*
		bla$_{CTX-M-15}$	*bla*$_{CTX-M-15}$
		bla$_{OXA-9}$	*bla*$_{OXA-9}$
		bla$_{TEM-1A}$	*bla*$_{TEM-150}$
	NODE_49_length_9874_cov_21.638658	*bla*$_{NDM-1}$	*bla*$_{NDM-1}$
			ble-$_{MBL}$
	NODE_5_length_350035_cov_38.211787	*fosA*	*fosA6*
	NODE_50_length_7198_cov_41.538821	*aac(3)-IId*	*aac(3)-IId*
	NODE_52_length_5366_cov_36.112044	*aph(3'')-Ib*	*aph(3'')-Ib*
		aph(6)-Id	*aph(6)-Id*
		sul2	*sul2*
	NODE_5length_6188_cov_115.028048	*bla*$_{OXA-232}$	*bla*$_{OXA-232}$
	NODE_63_length_1859_cov_28.921478	*bla*$_{OXA-1}$	*bla*$_{OXA-1}$
		catB3	
		catB3	
	NODE_64_length_1767_cov_66.489024	*catA1*	*catA1*
	NODE_7length_1175_cov_16.600191	*aph(3')-VI*	*aph(3')-VI*
SRR5168222	NODE_28_length_41957_cov_25.204662	*dfrA1*	*dfrA1*
			sat2_gen
	NODE_2length_80337_cov_14.686174	*bla*$_{SHV-106}$	*bla*$_{SHV-106}$

		bla$_{\text{SHV-28}}$	
	NODE_3_length_444346_cov_17.791175	*oqxA*	*oqxA6*
		OqxA	
		oqxB	*oqxB20*
		OqxB	
	NODE_36_length_2660cov_23.744844	*aadA2*	*aadA2*
		armA	*armA*
		dfrA12	*dfrA12*
		mph(E)	*mph(E)*
		msr(E)	*msr(E)*
		qacE	
		sul1	*sul1*
	NODE_47_length_12830_cov_25.863890	*bla*$_{\text{TEM-1A}}$	*bla*$_{\text{TEM-150}}$
	NODE_4length_20196_cov_24.925656	*bla*$_{\text{CTX-M-15}}$	*bla*$_{\text{CTX-M-15}}$
	NODE_5_length_350035_cov_18.348160	*fosA*	*fosA6*
	NODE_50_length_10294_cov_11.940494	*bla*$_{\text{NDM-1}}$	*bla*$_{\text{NDM-1}}$
			ble-$_{\text{MBL}}$
	NODE_52_length_6188_cov_53.945719	*bla*$_{\text{OXA-232}}$	*bla*$_{\text{OXA-232}}$
	NODE_53_length_5366_cov_17.962016	*aph(3'')-Ib*	*aph(3'')-Ib*
		aph(6)-Id	*aph(6)-Id*
		sul2	*sul2*
	NODE_59_length_2439_cov_22.891436	*aac(6')-Ib-cr*	*aac(6')-Ib-AKT*
		aac(6')-Ib3	
		bla$_{\text{OXA-1}}$	*bla*$_{\text{OXA-1}}$
		catB3	
		catB3	
	NODE_5length_7198_cov_21.696365	*aac(3)-IId*	*aac(3)-IId*
	NODE_65_length_1767_cov_33.571951	*catA1*	*catA1*
	NODE_6length_2260_cov_25.069386	*aadA1*	*aadA1*
		bla$_{\text{OXA-9}}$	*bla*$_{\text{OXA-9}}$
	NODE_73_length_1175_cov_13.558206	*aph(3')-VI*	*aph(3')-VI*
SRR5168231	NODE_114_length_642_cov_77.932039	*aadA2*	
		aadA2	
	NODE_30_length_59600_cov_28.043801	*catA1*	*catA1*
	NODE_4_length_270338_cov_24.028019	*fosA*	*fosA6*
	NODE_44_length_2385cov_31.085905	*aac(3)-IV*	*aac(3)-IVa*
		aph(4)-Ia	*aph(4)-Ia*
		bla$_{\text{KPC-2}}$	*bla*$_{\text{KPC-2}}$
	NODE_53_length_12725_cov_39.662883	*aac(6')-Ib*	*aac(6')-Ib-AKT*
		aac(6')-Ib-cr	
	NODE_75_length_4145_cov_32.511697	*aadA1*	*aadA1*
		cmlA1	*cmlA1*

	NODE_77_length_359cov_26.595554	*qacE*	
		sul1	*sul1*
	NODE_78_length_3484_cov_24.515043	*mph(A)*	*mph(A)*
	NODE_80_length_3198_cov_28.932921	*sul3*	*sul3*
	NODE_82_length_2632_cov_40.334132	*bla*$_{OXA-9}$	*bla*$_{OXA-9}$
		bla$_{TEM-1A}$	*bla*$_{TEM-1}$
	NODE_84_length_238cov_19.690328	*bla*$_{SHV-12}$	*bla*$_{SHV-12}$
	NODE_94_length_1272_cov_34.294323	*dfrA12*	*dfrA12*
	NODE_length_346757_cov_23.052116	*oqxA*	*oqxA*
		OqxA	
		oqxB	*oqxB*
		OqxB	
SRR5168232	NODE_108_length_923_cov_17.835427	*bla*$_{TEM-1A}$	*bla*$_{TEM-1}$
	NODE_109_length_899_cov_35.944301	*aac(6')-Ib*	*aac(6')-Ib-AKT*
		aac(6')-Ib-cr	
	NODE_117_length_722_cov_12.880672	*bla*$_{SHV-11}$	*bla*$_{SHV-11}$
	NODE_119_length_642_cov_46.718447	*aadA2*	
		aadA2	
	NODE_28_length_59600_cov_13.688699	*catA1*	*catA1*
	NODE_32_length_46726_cov_10.349407	*oqxA*	*oqxA*
		OqxA	
		oqxB	*oqxB*
		OqxB	
	NODE_38_length_33788_cov_11.391403	*fosA*	*fosA6*
	NODE_47_length_2385cov_14.126707	*aac(3)-IV*	*aac(3)-IVa*
		aph(4)-Ia	*aph(4)-Ia*
		bla$_{KPC-2}$	*bla*$_{KPC-2}$
	NODE_78_length_4145_cov_17.889995	*aadA1*	*aadA1*
		cmlA1	*cmlA1*
	NODE_79_length_359cov_14.469400	*qacE*	
		sul1	*sul1*
	NODE_80_length_3484_cov_8.314269	*mph(A)*	*mph(A)*
	NODE_82_length_3198_cov_15.394334	*sul3*	*sul3*
	NODE_95_length_1443_cov_18.009119	*bla*$_{OXA-9}$	*bla*$_{OXA-9}$
	NODE_98_length_1272_cov_15.255022	*dfrA12*	*dfrA12*
SRR5168235	NODE_103_length_92cov_24.186398	*aac(6')-Ib*	*aac(6')-Ib-AGKT*
		aac(6')-Ib-cr	
	NODE_112_length_672_cov_83.473394	*aadA1*	*aadA1*
		aadA1	
	NODE_13_length_132804_cov_20.697385	*bla*$_{SHV-187}$	*bla*$_{SHV-187}$
	NODE_3length_47178_cov_34.049393	*rmtG*	*rmtG*

		sul2	*sul2*
	NODE_46_length_12868_cov_32.659367	*bla*$_{\text{TEM-1A}}$	*bla*$_{\text{TEM-150}}$
	NODE_63_length_5237_cov_50.218982	*bla*$_{\text{OXA-10}}$	*bla*$_{\text{OXA-10}}$
		qacE	
		sul1	*sul1*
	NODE_65_length_3410_cov_28.964666	*ARR-2*	*arr-2*
		cmlA1	*cmlA5*
	NODE_7_length_262007_cov_25.452425	*fosA*	*fosA6*
	NODE_87_length_1442_cov_28.740684	*bla*$_{\text{OXA-9}}$	*bla*$_{\text{OXA-9}}$
	NODE_99_length_972_cov_12.908876	*bla*$_{\text{CTX-M-2}}$	*bla*$_{\text{CTX-M-2}}$
		bla$_{\text{CTX-M-97}}$	
SRR5168236	NODE_10length_1442_cov_14.717871	*bla*$_{\text{OXA-9}}$	*bla*$_{\text{OXA-9}}$
	NODE_116_length_723_cov_32.565436	*qacE*	
	NODE_12length_672_cov_46.904587	*aadA1*	*aadA1*
		aadA1	
		ant(3'')-Ia	
	NODE_35_length_47177_cov_16.247609	*rmtG*	*rmtG*
		sul2	*sul2*
	NODE_5_length_26160cov_12.102913	*fosA*	*fosA6*
	NODE_6length_12810_cov_16.452574	*bla*$_{\text{TEM-1A}}$	*bla*$_{\text{TEM-150}}$
	NODE_7_length_244460_cov_10.570463	*bla*$_{\text{SHV-187}}$	*bla*$_{\text{SHV-187}}$
	NODE_73_length_5959_cov_14.653635	*aac(6')-Ib*	*aac(6')-Ib-AGKT*
		aac(6')-Ib-cr	
	NODE_78_length_5115_cov_18.253007	*bla*$_{\text{CTX-M-2}}$	*bla*$_{\text{CTX-M-2}}$
		sul1	
		sul1	
		sul1	
		sul1	
		sul1	
		sul1	
		sul1	
		sul1	
	NODE_80_length_4348_cov_15.746269	*ARR-2*	*arr-2*
		bla$_{\text{OXA-10}}$	*bla*$_{\text{OXA-10}}$
		cmlA1	*cmlA5*
SRR5168243	NODE_13_length_151290_cov_22.235613	*bla*$_{\text{SHV-182}}$	*bla*$_{\text{SHV-187}}$
	NODE_16_length_110250_cov_33.983627	*catA1*	*catA1*
	NODE_36_length_19370_cov_70.095048	*bla*$_{\text{KPC-3}}$	*bla*$_{\text{KPC-3}}$
	NODE_45_length_9818_cov_30.435662	*aadA2*	*aadA2*
		dfrA12	*dfrA12*
		mph(A)	*mph(A)*

		qacE	
		sul1	*sul1*
	NODE_47_length_7094_cov_29.900244	*aadA1*	*aadA1*
		cmlA1	*cmlA1*
		sul3	*sul3*
	NODE_5length_4569_cov_33.981315	*aac(3)-IV*	*aac(3)-IVa*
		aph(4)-Ia	*aph(4)-Ia*
	NODE_6_length_270452_cov_23.861981	*fosA*	*fosA6*
	NODE_65_length_1293_cov_38.688679	*aph(3')-Ia*	*aph(3')-Ia*
	NODE_87_length_563_cov_94.408257	*aac(6')-Ib*	
		aac(6')-Ib-cr	
		aac(6')-Ib-cr	
	NODE_90_length_547_cov_45.311905	*bla*$_{TEM-150}$	
		bla$_{TEM-171}$	
		bla$_{TEM-1A}$	
		bla$_{TEM-1C}$	
		bla$_{TEM-40}$	
		bla$_{TEM-97}$	
		bla$_{TEM-98}$	
	NODE_length_839962_cov_24.126917	*oqxA*	*oqxA*
		OqxA	
		oqxB	*oqxB*
		OqxB	
SRR5168244	NODE_100_length_547_cov_18.876190	*bla*$_{TEM-150}$	
		bla$_{TEM-171}$	
		bla$_{TEM-1A}$	
		bla$_{TEM-1C}$	
		bla$_{TEM-40}$	
		bla$_{TEM-97}$	
		bla$_{TEM-98}$	
	NODE_16_length_110250_cov_15.708417	*catA1*	*catA1*
	NODE_23_length_66857_cov_24.671077	*aac(6')-Ib*	
		aac(6')-Ib-cr	
	NODE_38_length_19383_cov_29.473307	*bla*$_{KPC-3}$	*bla*$_{KPC-3}$
	NODE_50_length_7825_cov_12.280722	*mph(A)*	*mph(A)*
		qacE	
		sul1	*sul1*
	NODE_52_length_7094_cov_14.778527	*aadA1*	*aadA1*
		cmlA1	*cmlA1*
		sul3	*sul3*
	NODE_56_length_4485_cov_11.211565	*aac(3)-IV*	*aac(3)-IVa*
		aph(4)-Ia	*aph(4)-Ia*

	NODE_76_length_1293_cov_18.145798	*aph(3')-Ia*	*aph(3')-Ia*
	NODE_77_length_1272_cov_14.110917	*dfrA12*	*dfrA12*
	NODE_8_length_270452_cov_10.378204	*fosA*	*fosA6*
	NODE_88_length_722_cov_25.205042	*bla*$_{\text{SHV-12}}$	*bla*$_{\text{SHV-187}}$
		bla$_{\text{SHV-129}}$	
		bla$_{\text{SHV-13}}$	
		bla$_{\text{SHV-155}}$	
		bla$_{\text{SHV-172}}$	
		bla$_{\text{SHV-31}}$	
	NODE_94_length_642_cov_26.485437	*aadA2*	
		aadA2	
	NODE_99_length_563_cov_44.094037	*aac(6')-Ib*	
		aac(6')-Ib-cr	
		aac(6')-Ib-cr	
	NODE_length_523637_cov_10.253691	*OqxA*	
		oqxA	*oqxA*
		OqxB	
		oqxB	*oqxB*
SRR5168370	NODE_14_length_157792_cov_18.342219	*bla*$_{\text{SHV-182}}$	*bla*$_{\text{SHV-158}}$
	NODE_29_length_3331cov_29.306081	*catA1*	*catA1*
	NODE_37_length_19048_cov_55.968183	*bla*$_{\text{KPC-3}}$	*bla*$_{\text{KPC-3}}$
	NODE_3length_32118_cov_25.050671	*bla*$_{\text{TEM-150}}$	
		bla$_{\text{TEM-171}}$	
		bla$_{\text{TEM-1A}}$	
		bla$_{\text{TEM-1C}}$	
		bla$_{\text{TEM-40}}$	
	NODE_48_length_7825_cov_24.978826	*mph(A)*	*mph(A)*
		qacE	
		sul1	*sul1*
	NODE_50_length_7094_cov_25.559064	*aadA1*	*aadA1*
		cmlA1	*cmlA1*
		sul3	*sul3*
	NODE_55_length_4569_cov_26.631247	*aac(3)-IV*	*aac(3)-IVa*
		aph(4)-Ia	*aph(4)-Ia*
	NODE_7_length_288896_cov_18.814502	*OqxA*	
		oqxA	*oqxA*
		OqxB	
		oqxB	*oqxB*
	NODE_70_length_1293_cov_38.613208	*aph(3')-Ia*	*aph(3')-Ia*
	NODE_73_length_1272_cov_29.101310	*dfrA12*	*dfrA12*
	NODE_8_length_270452_cov_19.055152	*fosA*	*fosA6*
	NODE_87_length_642_cov_41.390291	*aadA2*	

		aadA2	
	NODE_93_length_563_cov_107.759174	*aac(6')-Ib*	
		aac(6')-Ib-cr	
		aac(6')-Ib-cr	
SRR516837 1	NODE_12_length_15769cov_19.892253	*bla*$_{\text{SHV-182}}$	*bla*$_{\text{SHV-158}}$
	NODE_30_length_3331cov_31.748614	*catA1*	*catA1*
	NODE_39_length_19370_cov_59.562906	*bla*$_{\text{KPC-3}}$	*bla*$_{\text{KPC-3}}$
	NODE_49_length_9808_cov_30.585890	*aadA1*	*aadA1*
		aadA2b	*aadA2*
		cmlA1	*cmlA1*
		sul3	*sul3*
	NODE_50_length_7825_cov_28.027540	*mph(A)*	*mph(A)*
		qacE	
		sul1	*sul1*
	NODE_56_length_4569_cov_27.725124	*aac(3)-IV*	*aac(3)-IVa*
		aph(4)-Ia	*aph(4)-Ia*
	NODE_6_length_270452_cov_20.967580	*fosA*	*fosA6*
	NODE_67_length_1293_cov_34.596055	*aph(3')-Ia*	*aph(3')-Ia*
	NODE_69_length_1272_cov_37.101310	*dfrA12*	*dfrA12*
	NODE_88_length_563_cov_104.642202	*aac(6')-Ib*	
		aac(6')-Ib-cr	
		aac(6')-Ib-cr	
	NODE_8length_738_cov_122.782324	*aac(6')-Ib*	
		aac(6')-Ib-cr	
	NODE_90_length_547_cov_44.564286	*bla*$_{\text{TEM-150}}$	
		bla$_{\text{TEM-171}}$	
		bla$_{\text{TEM-1A}}$	
		bla$_{\text{TEM-1C}}$	
		bla$_{\text{TEM-40}}$	
		bla$_{\text{TEM-97}}$	
		bla$_{\text{TEM-98}}$	
	NODE_length_839788_cov_21.208488	*OqxA*	
		oqxA	*oqxA*
		OqxB	
		oqxB	*oqxB*
SRR516837 2	NODE_10_length_166060_cov_8.763097	*bla*$_{\text{SHV-182}}$	*bla*$_{\text{SHV-158}}$
	NODE_32_length_3331cov_13.965917	*catA1*	*catA1*
	NODE_33_length_32224_cov_14.681684	*bla*$_{\text{TEM-150}}$	
		bla$_{\text{TEM-171}}$	
		bla$_{\text{TEM-1A}}$	
		bla$_{\text{TEM-1C}}$	
		bla$_{\text{TEM-40}}$	

	NODE_43_length_19048_cov_26.084404	*bla*$_{KPC-3}$	*bla*$_{KPC-3}$
	NODE_5_length_270452_cov_8.798195	*fosA*	*fosA6*
	NODE_52_length_7825_cov_12.036373	*mph(A)*	*mph(A)*
		qacE	
		sul1	*sul1*
	NODE_53_length_7094_cov_14.739486	*aadA1*	*aadA1*
		cmlA1	*cmlA1*
		sul3	*sul3*
	NODE_59_length_4485_cov_10.388481	*aac(3)-IV*	*aac(3)-IVa*
		aph(4)-Ia	*aph(4)-Ia*
	NODE_74_length_1293_cov_18.435678	*aph(3')-Ia*	*aph(3')-Ia*
	NODE_76_length_1272_cov_14.510917	*dfrA12*	*dfrA12*
	NODE_86_length_899_cov_55.099741	*aac(6')-Ib*	*aac(6')-Ib-AKT*
		aac(6')-Ib-cr	
	NODE_93_length_642_cov_21.982524	*aadA2*	
	NODE_length_839490_cov_9.184293	*oqxA*	*oqxA*
		OqxA	
		oqxB	*oqxB*
		OqxB	
SRR5168375	NODE_20_length_85380_cov_32.175267	*oqxA*	*oqxA*
		OqxA	
		oqxB	*oqxB*
		OqxB	
	NODE_27_length_48844_cov_45.486155	*catA1*	*catA1*
	NODE_29_length_42898_cov_34.651329	*bla*$_{SHV-12}$	*bla*$_{SHV-12}$
	NODE_34_length_21958_cov_78.061976	*bla*$_{KPC-3}$	*bla*$_{KPC-3}$
		bla$_{TEM-1A}$	*bla*$_{TEM-150}$
	NODE_35_length_17718_cov_45.083963	*aac(3)-IV*	*aac(3)-IVa*
		aph(4)-Ia	*aph(4)-Ia*
	NODE_46_length_359cov_44.475751	*qacE*	
		sul1	*sul1*
	NODE_47_length_3484_cov_34.008937	*mph(A)*	*mph(A)*
	NODE_4length_7095_cov_48.023823	*aadA1*	*aadA1*
		cmlA1	*cmlA1*
		sul3	*sul3*
	NODE_57_length_1292_cov_45.826609	*aph(3')-Ia*	*aph(3')-Ia*
	NODE_59_length_1272_cov_48.344978	*dfrA12*	*dfrA12*
	NODE_68_length_84cov_89.754902	*aadA2b*	*aadA2*
	NODE_7_length_270452_cov_31.609196	*fosA*	*fosA6*
SRR5168376	NODE_19_length_85380_cov_14.594759	*oqxA*	*oqxA*
		OqxA	
		oqxB	*oqxB*

		OqxB	
	NODE_25_length_48844_cov_20.985939	*catA1*	*catA1*
	NODE_33_length_21957_cov_33.821530	*bla*$_{KPC-3}$	*bla*$_{KPC-3}$
		bla$_{TEM-1A}$	*bla*$_{TEM-150}$
	NODE_35_length_17825_cov_20.647248	*aac(3)-IV*	*aac(3)-IVa*
		aph(4)-Ia	*aph(4)-Ia*
	NODE_42_length_7095_cov_24.129162	*aadA1*	*aadA1*
		cmlA1	*cmlA1*
		sul3	*sul3*
	NODE_46_length_359cov_18.713337	*qacE*	
		sul1	*sul1*
	NODE_47_length_3484_cov_14.547513	*mph(A)*	*mph(A)*
	NODE_58_length_1292_cov_22.710730	*aph(3')-Ia*	*aph(3')-Ia*
	NODE_59_length_1272_cov_19.221834	*dfrA12*	*dfrA12*
	NODE_66_length_84cov_45.477591	*aadA2b*	*aadA2*
	NODE_7_length_270412_cov_13.835044	*fosA*	*fosA6*
SRR5168377	NODE_29_length_71027_cov_54.334781	*bla*$_{TEM-1A}$	*bla*$_{TEM-150}$
	NODE_3_length_313595_cov_23.482757	*fosA*	*fosA6*
	NODE_38_length_35855_cov_22.660574	*oqxA*	*oqxA10*
		OqxA	
		oqxB	*oqxB4*
		OqxB	
	NODE_50_length_2073cov_123.589497	*dfrA14*	*dfrA14*
	NODE_59_length_12464_cov_97.090703	*bla*$_{KPC-3}$	*bla*$_{KPC-3}$
	NODE_70_length_3483_cov_34.935638	*ant(2'')-Ia*	*ant(2'')-Ia*
		catB3	*catB3*
		qacE	
		sul1	*sul1*
	NODE_75_length_2012_cov_33.567639	*bla*$_{SHV-5}$	
	NODE_76_length_1907_cov_49.878652	*aadA1*	*aadA1*
		bla$_{OXA-9}$	*bla*$_{OXA-9}$
	NODE_7length_285cov_45.577460	*dfrA19*	*dfrA19*
	NODE_93_length_753_cov_191.856230	*aac(6')-Ib*	*aac(6')-Ib-AKT*
		aac(6')-Ib-cr	
		aac(6')-Ib-cr	
SRR5168378	NODE_2_length_313595_cov_9.981386	*fosA*	*fosA6*
	NODE_4length_35855_cov_10.153773	*OqxA*	*oqxA10*
		oqxA	
		OqxB	
		oqxB	*oqxB4*
	NODE_5length_19568_cov_51.452857	*dfrA14*	*dfrA14*
	NODE_62_length_10257_cov_32.645903	*bla*$_{KPC-3}$	*bla*$_{KPC-3}$

	NODE_77_length_3483_cov_12.136174	*ant(2")-Ia*	*ant(2")-Ia*
		catB3	*catB3*
		qacE	
		sul1	*sul1*
	NODE_78_length_285cov_20.609398	*dfrA19*	*dfrA19*
	NODE_80_length_2289_cov_12.949584	*bla*$_{\text{SHV-27}}$	*bla*$_{\text{SHV-27}}$
	NODE_82_length_1786_cov_75.493068	*aac(6')-Ib*	
		aac(6')-Ib-cr	
	NODE_8length_1907_cov_28.262921	*aadA1*	*aadA1*
		bla$_{\text{OXA-9}}$	*bla*$_{\text{OXA-9}}$
	NODE_97_length_923_cov_30.668342	*bla*$_{\text{TEM-1A}}$	*bla*$_{\text{TEM-150}}$
SRR5168384	NODE_107_length_1293_cov_21.380789	*aph(3')-Ia*	*aph(3')-Ia*
	NODE_108_length_1255_cov_18.767730	*aac(6')-Ib*	*aac(6')-Ib-AKT*
		aac(6')-Ib-cr	
	NODE_117_length_1080_cov_27.399790	*bla*$_{\text{TEM-1D}}$	*bla*$_{\text{TEM-12}}$
	NODE_119_length_103cov_17.390487	*bla*$_{\text{SHV-182}}$	
	NODE_42_length_33788_cov_13.496301	*fosA*	*fosA6*
	NODE_52_length_25616_cov_26.668210	*floR*	*floR*
		sul2	*sul2*
	NODE_75_length_6210_cov_20.090580	*mph(A)*	*mph(A)*
	NODE_79_length_4386_cov_23.617281	*bla*$_{\text{KPC-2}}$	*bla*$_{\text{KPC-2}}$
	NODE_88_length_2009_cov_21.409671	*aadA2*	*aadA2*
		dfrA12	*dfrA12*
	NODE_95_length_1595_cov_34.105586	*qacE*	
		sul1	*sul1*
	NODE_96_length_1566_cov_24.583739	*aac(6')-33*	*aac(6')-33*
	NODE_96_length_1566_cov_24.583739	*ant(2")-Ia*	*ant(2")-Ia*
	NODE_length_521073_cov_14.247815	*oqxA*	*oqxA*
		OqxA	
		oqxB	*oqxB*
		OqxB	
SRR5168385	NODE_25_length_94569_cov_25.472925	*oqxA*	*oqxA*
		OqxA	
		oqxB	*oqxB*
		OqxB	
	NODE_42_length_25616_cov_46.800855	*floR*	*floR*
		sul2	*sul2*
	NODE_49_length_14703_cov_25.150717	*aac(6')-Ib*	*aac(6')-Ib-AKT*
		aac(6')-Ib-cr	
	NODE_5_length_270452_cov_24.990486	*fosA*	*fosA6*
	NODE_55_length_6210_cov_35.735821	*mph(A)*	*mph(A)*
	NODE_6length_4386_cov_40.506222	*bla*$_{\text{KPC-2}}$	*bla*$_{\text{KPC-2}}$

	NODE_70_length_2009_cov_35.214134	*aadA2*	*aadA2*
		dfrA12	*dfrA12*
	NODE_76_length_1595_cov_62.942098	*qacE*	
		sul1	*sul1*
	NODE_77_length_1566_cov_42.488534	*aac(6')-33*	*aac(6')-33*
		ant(2'')-Ia	*ant(2'')-Ia*
	NODE_85_length_1293_cov_32.771870	*aph(3')-Ia*	*aph(3')-Ia*
	NODE_94_length_1080_cov_49.757608	*bla*$_{\text{TEM-1D}}$	*bla*$_{\text{TEM-12}}$
	NODE_99_length_817_cov_31.494203	*bla*$_{\text{SHV-11}}$	*bla*$_{\text{SHV-11}}$
		bla$_{\text{SHV-155}}$	
		bla$_{\text{SHV-158}}$	
		bla$_{\text{SHV-159}}$	
		bla$_{\text{SHV-182}}$	
SRR5168386	NODE_4_length_270452_cov_23.872457	*fosA*	*fosA6*
	NODE_46_length_20123_cov_43.924085	*floR*	*floR*
	NODE_55_length_6210_cov_34.144501	*mph(A)*	*mph(A)*
	NODE_57_length_5107_cov_43.967871	*sul2*	*sul2*
	NODE_59_length_4386_cov_37.783517	*bla*$_{\text{KPC-2}}$	*bla*$_{\text{KPC-2}}$
	NODE_65_length_2009_cov_33.323061	*aadA2*	*aadA2*
		dfrA12	*dfrA12*
	NODE_69_length_1595_cov_61.754768	*qacE*	
		sul1	*sul1*
	NODE_70_length_1566_cov_37.124392	*aac(6')-33*	*aac(6')-33*
		ant(2'')-Ia	*ant(2'')-Ia*
	NODE_78_length_1293_cov_31.763293	*aph(3')-Ia*	*aph(3')-Ia*
	NODE_86_length_1080_cov_51.584470	*bla*$_{\text{TEM-1D}}$	*bla*$_{\text{TEM-12}}$
	NODE_8length_1255_cov_28.937057	*aac(6')-Ib*	*aac(6')-Ib-AKT*
		aac(6')-Ib-cr	
	NODE_90_length_817_cov_28.443478	*bla*$_{\text{SHV-11}}$	*bla*$_{\text{SHV-11}}$
		bla$_{\text{SHV-155}}$	
		bla$_{\text{SHV-158}}$	
		bla$_{\text{SHV-159}}$	
		bla$_{\text{SHV-182}}$	
	NODE_length_521465_cov_24.021905	*OqxA*	
		oqxA	*oqxA*
		OqxB	
		oqxB	*oqxB*
SRR5168387	NODE_104_length_737_cov_106.950820	*aac(6')-Ib*	
		aac(6')-Ib-cr	
	NODE_108_length_722_cov_55.436975	*bla*$_{\text{SHV-12}}$	*bla*$_{\text{SHV-187}}$
		bla$_{\text{SHV-129}}$	
		bla$_{\text{SHV-13}}$	

		bla$_{SHV-155}$	
		bla$_{SHV-172}$	
		bla$_{SHV-31}$	
	NODE_113_length_642_cov_59.186408	*aadA2*	
		aadA2	
	NODE_116_length_592_cov_67.694624	*aadA1*	
		aadA24	
	NODE_118_length_547_cov_36.354762	*bla*$_{TEM-150}$	
		bla$_{TEM-171}$	
		bla$_{TEM-1A}$	
		bla$_{TEM-1C}$	
		bla$_{TEM-40}$	
		bla$_{TEM-97}$	
		bla$_{TEM-98}$	
	NODE_12length_523_cov_113.297980	*aac(6')-Ib-cr*	
	NODE_33_length_47583_cov_42.852769	*catA1*	*catA1*
	NODE_4_length_270453_cov_22.236381	*fosA*	*fosA6*
	NODE_52_length_10008_cov_111.782411	*bla*$_{KPC-3}$	*bla*$_{KPC-3}$
	NODE_55_length_7826_cov_35.312898	*mph(A)*	*mph(A)*
		qacE	
		sul1	*sul1*
	NODE_60_length_542cov_25.374764	*aac(3)-IV*	*aac(3)-IVa*
		aph(4)-Ia	*aph(4)-Ia*
	NODE_63_length_4856_cov_17.888771	*sul3*	*sul3*
	NODE_80_length_1799_cov_23.355263	*cmlA1*	*cmlA1*
	NODE_86_length_1442_cov_28.175665	*bla*$_{OXA-9}$	*bla*$_{OXA-9}$
	NODE_9_length_19913cov_20.614068	*OqxA*	
		oqxA	*oqxA*
		OqxB	
		oqxB	*oqxB*
	NODE_92_length_1272_cov_43.073362	*dfrA12*	*dfrA12*
	NODE_9length_1293_cov_39.524014	*aph(3')-Ia*	*aph(3')-Ia*
SRR5168388	NODE_102_length_737_cov_98.473770	*aac(6')-Ib*	
		aac(6')-Ib-cr	
	NODE_105_length_722_cov_55.620168	*bla*$_{SHV-12}$	*bla*$_{SHV-187}$
		bla$_{SHV-129}$	
		bla$_{SHV-13}$	
		bla$_{SHV-155}$	
		bla$_{SHV-172}$	
		bla$_{SHV-31}$	
	NODE_117_length_547_cov_35.414286	*bla*$_{TEM-150}$	
		bla$_{TEM-171}$	

		*bla*TEM-1A	
		*bla*TEM-1C	
		*bla*TEM-40	
		*bla*TEM-97	
		*bla*TEM-98	
	NODE_11length_592_cov_47.537634	*aadA1*	
		aadA24	
	NODE_27_length_47583_cov_41.950881	*catA1*	*catA1*
	NODE_45_length_10008_cov_112.158284	*bla*KPC-3	*bla*KPC-3
	NODE_5_length_273090_cov_23.516249	*fosA*	*fosA6*
	NODE_50_length_7826_cov_38.157163	*mph(A)*	*mph(A)*
		qacE	
		sul1	*sul1*
	NODE_56_length_542cov_25.589535	*aac(3)-IV*	*aac(3)-IVa*
		aph(4)-Ia	*aph(4)-Ia*
	NODE_59_length_4855_cov_16.317047	*sul3*	*sul3*
	NODE_73_length_1799_cov_25.539474	*cmlA1*	*cmlA1*
	NODE_78_length_1442_cov_31.425856	*bla*OXA-9	*bla*OXA-9
	NODE_84_length_1293_cov_38.352487	*aph(3')-Ia*	*aph(3')-Ia*
	NODE_85_length_1272_cov_45.106550	*dfrA12*	*dfrA12*
	NODE_93_length_84cov_58.823529	*aadA2b*	*aadA2*
	NODE_99_length_789_cov_126.175227	*aac(6')-Ib*	
		aac(6')-Ib-cr	
		aac(6')-Ib-cr	
	NODE_length_696127_cov_21.849624	*OqxA*	
		oqxA	*oqxA*
		OqxB	
		oqxB	*oqxB*
SRR5168389	NODE_12_length_218218_cov_24.677809	*bla*SHV-145	*bla*SHV-145
		*bla*SHV-179	
		*bla*SHV-194	
		*bla*SHV-199	
		*bla*SHV-26	
		*bla*SHV-78	
		*bla*SHV-98	
	NODE_2_length_35808cov_25.453581	*fosA*	*fosA_gen*
	NODE_22_length_58026_cov_24.346224	*oqxA*	*oqxA11*
		OqxA	
		oqxB	*oqxB20*
		OqxB	
	NODE_25_length_43748_cov_24.192339	*bla*KPC-2	*bla*KPC-2
		*bla*TEM-1B	*bla*TEM-1

	NODE_2length_65197_cov_28.897126	*bla*$_{SHV-30}$	*bla*$_{SHV-30}$
	NODE_43_length_3626_cov_39.111460	*aac(6')-Ib-cr*	*aac(6')-Ib-D181Y*
		aac(6')-Ib-cr	
		ARR-3	*arr-3*
		bla$_{OXA-1}$	*bla*$_{OXA-1}$
		catB3	*catB3*
	NODE_46_length_1964_cov_32.107784	*aadA1*	*aadA1*
		dfrA1	*dfrA1*
		dfrA1	
		dfrA1	
	NODE_50_length_1594_cov_48.355828	*qacE*	
		sul1	*sul1*
SRR5168390	NODE_19_length_58026_cov_24.994577	*OqxA*	
		oqxA	*oqxA11*
		OqxB	
		oqxB	*oqxB20*
	NODE_25_length_35690_cov_27.302280	*bla*$_{SHV-30}$	*bla*$_{SHV-30}$
	NODE_2length_43748_cov_23.788267	*bla*$_{KPC-2}$	*bla*$_{KPC-2}$
		bla$_{TEM-1B}$	*bla*$_{TEM-1}$
	NODE_38_length_3626_cov_39.002572	*aac(6')-Ib-cr*	*aac(6')-Ib-D181Y*
		aac(6')-Ib-cr	
		ARR-3	*arr-3*
		bla$_{OXA-1}$	*bla*$_{OXA-1}$
		catB3	*catB3*
	NODE_43_length_1964_cov_31.823625	*aadA1*	*aadA1*
		dfrA1	*dfrA1*
		dfrA1	
		dfrA1	
	NODE_46_length_1594_cov_45.713020	*qacE*	
		sul1	*sul1*
	NODE_6_length_358249_cov_25.889616	*fosA*	*fosA_gen*
	NODE_length_539004_cov_22.929661	*bla*$_{SHV-145}$	*bla*$_{SHV-145}$
		bla$_{SHV-179}$	
		bla$_{SHV-194}$	
		bla$_{SHV-199}$	
		bla$_{SHV-26}$	
		bla$_{SHV-78}$	
		bla$_{SHV-98}$	
SRR5168393	NODE_14_length_151290_cov_22.499567	*bla*$_{SHV-182}$	*bla*$_{SHV-158}$
	NODE_16_length_121236_cov_33.734900	*aadA2*	*aadA2*
		catA1	*catA1*

		dfrA12	*dfrA12*
		mph(A)	*mph(A)*
		qacE	
		sul1	*sul1*
	NODE_3_length_367932_cov_24.192338	*fosA*	*fosA6*
	NODE_33_length_29909_cov_24.702471	*bla*$_{TEM-1A}$	*bla*$_{TEM-150}$
	NODE_45_length_10526_cov_20.273776	*bla*$_{KPC-3}$	*bla*$_{KPC-3}$
	NODE_63_length_1293_cov_31.018868	*aph(3')-Ia*	*aph(3')-Ia*
	NODE_66_length_1256_cov_28.809566	*aac(6')-Ib*	*aac(6')-Ib-AKT*
		aac(6')-Ib-cr	
	NODE_length_779309_cov_24.219908	*OqxA*	
		oqxA	*oqxA*
		OqxB	
		oqxB	*oqxB*
SRR5168394	NODE_14_length_150867_cov_22.695137	*bla*$_{SHV-182}$	*bla*$_{SHV-158}$
	NODE_16_length_121236_cov_33.467562	*aadA2*	*aadA2*
		catA1	*catA1*
		dfrA12	*dfrA12*
		mph(A)	*mph(A)*
		qacE	
		sul1	*sul1*
	NODE_3_length_369350_cov_24.937087	*fosA*	*fosA6*
	NODE_33_length_2978cov_24.218082	*bla*$_{TEM-1A}$	*bla*$_{TEM-150}$
	NODE_45_length_12054_cov_20.791565	*bla*$_{KPC-3}$	*bla*$_{KPC-3}$
	NODE_63_length_1293_cov_33.594340	*aph(3')-Ia*	*aph(3')-Ia*
	NODE_67_length_1128_cov_33.100899	*aac(6')-Ib*	*aac(6')-Ib-AKT*
		aac(6')-Ib-cr	
	NODE_length_449995_cov_23.823357	*OqxA*	
		oqxA	*oqxA*
		OqxB	
		oqxB	*oqxB*
SRR5168481	NODE_102_length_1186_cov_19.395656	*dfrA14*	*dfrA14*
	NODE_119_length_749_cov_21.583601	*ARR-2*	*arr-2*
	NODE_15_length_111094_cov_14.796886	*bla*$_{SHV-11}$	*bla*$_{SHV-11}$
		bla$_{SHV-67}$	
	NODE_16_length_111018_cov_15.681561	*bla*$_{CTX-M-15}$	*bla*$_{CTX-M-15}$
	NODE_36_length_46995_cov_15.387877	*OqxA*	
		oqxA	*oqxA6*
		OqxB	
		oqxB	*oqxB19*
	NODE_56_length_1003cov_22.384693	*bla*$_{NDM-7}$	*bla*$_{NDM-7}$
			ble-$_{MBL}$

	NODE_5length_16177_cov_15.903738	*fosA*	*fosA_gen*
	NODE_64_length_595cov_21.253777	*aac(6')-Ib-cr*	*aac(6')-Ib-G*
		aac(6')-Ib-Hangzhou	*catB*
		rmtF	*rmtF1*
	NODE_67_length_5437_cov_24.502072	*sul2*	*sul2*
	NODE_76_length_3635_cov_23.929875	*qacE*	
		sul1	*sul1*
	NODE_77_length_3485_cov_27.563728	*mph(A)*	*mph(A)*
	NODE_82_length_2909_cov_14.380302	bla_{TEM-1B}	bla_{TEM-1}
	NODE_84_length_2544_cov_29.967729	*aadA2*	*aadA2*
		dfrA12	*dfrA12*
	NODE_85_length_2210_cov_21.147864	*qnrB1*	*qnrB1*
	NODE_93_length_1649_cov_12.810118	*catA2*	*catA2*
	NODE_96_length_1478_cov_54.619541	*aph(3'')-Ib*	
		aph(3'')-Ib	
		aph(3'')-Ib	
		aph(6)-Id	*aph(6)-Id*
SRR5168482	NODE_113_length_749_cov_15.397106	*ARR-2*	*arr-2*
	NODE_16_length_135066_cov_18.781724	*fosA*	*fosA_gen*
	NODE_30_length_53947_cov_16.115162	*oqxA*	*oqxA6*
		OqxA	
		oqxB	*oqxB19*
		OqxB	
	NODE_40_length_3355cov_19.024174	$bla_{CTX-M-15}$	$bla_{CTX-M-15}$
	NODE_5_length_32037cov_15.446619	bla_{SHV-11}	bla_{SHV-11}
		bla_{SHV-67}	
	NODE_55_length_12984_cov_29.697286	*aph(3'')-Ib*	*aph(3'')-Ib*
		aph(3'')-Ib	*aph(3'')-Ib*
		aph(3'')-Ib	
		aph(3'')-Ib	
		aph(3'')-Ib	
		aph(6)-Id	*aph(6)-Id*
		aph(6)-Id	*aph(6)-Id*
		bla_{TEM-1B}	bla_{TEM-1}
		sul2	*sul2*
	NODE_62_length_6522_cov_34.858327	*qnrB1*	*qnrB1*
	NODE_63_length_595cov_20.413633	*aac(6')-Ib-cr*	*aac(6')-Ib-G*
		aac(6')-Ib-Hangzhou	*catB*
		rmtF	*rmtF1*
	NODE_67_length_4978_cov_15.775098	bla_{NDM-7}	bla_{NDM-7}
			ble-$_{MBL}$

	NODE_73_length_3635_cov_26.065279	*qacE*	
		sul1	*sul1*
	NODE_75_length_3485_cov_31.952353	*mph(A)*	*mph(A)*
	NODE_84_length_2544_cov_34.282168	*aadA2*	*aadA2*
		dfrA12	*dfrA12*
	NODE_90_length_1649_cov_12.887648	*catA2*	*catA2*
	NODE_97_length_1186_cov_22.279509	*dfrA14*	*dfrA14*
SRR5168483	NODE_10length_2544_cov_34.218866	*aadA2*	*aadA2*
		dfrA12	*dfrA12*
	NODE_113_length_1649_cov_9.670171	*catA2*	*catA2*
	NODE_115_length_1478_cov_63.984456	*aph(3'')-Ib*	
		aph(3'')-Ib	
		aph(3'')-Ib	
		aph(6)-Id	*aph(6)-Id*
	NODE_119_length_1186_cov_18.477809	*dfrA14*	*dfrA14*
	NODE_132_length_749_cov_22.765273	*ARR-2*	*arr-2*
	NODE_1length_133240_cov_16.188463	*fosA*	*fosA_gen*
	NODE_34_length_53828_cov_15.316549	*oqxA*	*oqxA6*
		OqxA	
		oqxB	*oqxB19*
		OqxB	
	NODE_36_length_5019cov_14.762424	*bla*$_{\text{SHV-11}}$	*bla*$_{\text{SHV-11}}$
		bla$_{\text{SHV-67}}$	
	NODE_52_length_33496_cov_18.106027	*bla*$_{\text{CTX-M-15}}$	*bla*$_{\text{CTX-M-15}}$
	NODE_76_length_595cov_18.560096	*aac(6')-Ib-cr*	*aac(6')-Ib-G*
		aac(6')-Ib-Hangzhou	*catB*
		rmtF	*rmtF1*
	NODE_77_length_5847_cov_31.159441	*qnrB1*	*qnrB1*
	NODE_79_length_5673_cov_25.481789	*sul2*	*sul2*
	NODE_8length_5408_cov_15.108313	*bla*$_{\text{NDM-7}}$	*bla*$_{\text{NDM-7}}$
			ble-$_{\text{MBL}}$
	NODE_93_length_3635_cov_23.460091	*qacE*	
		sul1	*sul1*
	NODE_94_length_3485_cov_26.215605	*mph(A)*	*mph(A)*
	NODE_99_length_2909_cov_13.937096	*bla*$_{\text{TEM-1B}}$	*bla*$_{\text{TEM-1}}$
SRR5168485	NODE_20_length_117416_cov_25.697354	*fosA*	*fosA6*
	NODE_24_length_107744_cov_19.745932	*bla*$_{\text{SHV-110}}$	*bla*$_{\text{SHV-110}}$
		bla$_{\text{SHV-81}}$	
	NODE_34_length_47763_cov_30.091548	*bla*$_{\text{CMY-4}}$	*bla*$_{\text{CMY-4}}$
	NODE_42_length_29788_cov_27.350595	*aph(3'')-Ib*	*aph(3'')-Ib*
		aph(6)-Id	*aph(6)-Id*

		sul2	*sul2*
	NODE_46_length_10770_cov_74.492342	*aph(3')-VI*	*aph(3')-VI*
		bla$_{\text{NDM-1}}$	*bla*$_{\text{NDM-1}}$
			ble-$_{\text{MBL}}$
	NODE_54_length_8630_cov_28.173703	*armA*	*armA*
		mph(E)	*mph(E)*
		msr(E)	*msr(E)*
	NODE_65_length_413cov_24.021728	*aac(6')-IIa*	*aac(6')-IIa*
		aadA2	*aadA2*
		dfrA1	*dfrA1*
			catB11
	NODE_66_length_3967_cov_58.386719	*bla*$_{\text{CTX-M-15}}$	*bla*$_{\text{CTX-M-15}}$
	NODE_69_length_3470_cov_80.797487	*qacE*	
		sul1	*sul1*
	NODE_6length_5142_cov_65.066002	*aadA1*	*aadA1*
		aadA1	
		ARR-2	*arr-2*
		bla$_{\text{OXA-10}}$	*bla*$_{\text{OXA-10}}$
		cmlA1	*cmlA5*
	NODE_77_length_2057_cov_60.479793	*tet(A)*	*tet(A)*
	NODE_length_356652_cov_23.472205	*OqxA*	
		oqxA	*oqxA*
		OqxB	
		oqxB	*oqxB*
SRR5168486	NODE_83_length_2057_cov_64.537824	*tet(A)*	*tet(A)*
	NODE_7length_3967_cov_58.034375	*bla*$_{\text{CTX-M-15}}$	*bla*$_{\text{CTX-M-15}}$
	NODE_75_length_3470_cov_84.525576	*qacE*	
		sul1	*sul1*
	NODE_68_length_413cov_22.312937	*aac(6')-IIa*	*aac(6')-IIa*
		aadA2	*aadA2*
		dfrA1	*dfrA1*
			catB11
	NODE_64_length_5142_cov_65.630110	*aadA1*	*aadA1*
		aadA1	
		ARR-2	*arr-2*
		bla$_{\text{OXA-10}}$	*bla*$_{\text{OXA-10}}$
		cmlA1	*cmlA5*
	NODE_58_length_7052_cov_24.853574	*armA*	*armA*
		mph(E)	*mph(E)*
		msr(E)	*msr(E)*
	NODE_49_length_10770_cov_73.074039	*aph(3')-VI*	*aph(3')-VI*
		bla$_{\text{NDM-1}}$	*bla*$_{\text{NDM-1}}$

			ble-$_{\text{MBL}}$
	NODE_42_length_29788_cov_27.121135	*aph(3'')-Ib*	*aph(3'')-Ib*
		aph(6)-Id	*aph(6)-Id*
		sul2	*sul2*
	NODE_34_length_47763_cov_30.843984	*bla*$_{\text{CMY-4}}$	*bla*$_{\text{CMY-4}}$
	NODE_2_length_358924_cov_25.065380	*OqxA*	
		oqxA	*oqxA*
		OqxB	
		oqxB	*oqxB*
	NODE_19_length_117416_cov_24.952843	*fosA*	*fosA6*
	NODE_13_length_142235_cov_19.567815	*bla*$_{\text{SHV-110}}$	*bla*$_{\text{SHV-110}}$
		bla$_{\text{SHV-81}}$	
SRR5168488	NODE_116_length_684_cov_58.122083	*bla*$_{\text{TEM-168}}$	
		bla$_{\text{TEM-181}}$	
		bla$_{\text{TEM-183}}$	
		bla$_{\text{TEM-1A}}$	
		bla$_{\text{TEM-231}}$	
		bla$_{\text{TEM-54}}$	
		bla$_{\text{TEM-93}}$	
	NODE_117_length_672_cov_102.412844	*aadA1*	*aadA1*
		aadA1	
		ant(3'')-Ia	
	NODE_15_length_139794_cov_23.080756	*bla*$_{\text{SHV-106}}$	
		bla$_{\text{SHV-28}}$	*bla*$_{\text{SHV-28}}$
	NODE_2_length_350548_cov_27.158444	*fosA*	*fosA6*
	NODE_3length_41954_cov_32.731370	*dfrA1*	*dfrA1*
			sat2_gen
	NODE_40_length_27134_cov_40.062428	*bla*$_{\text{CMY-4}}$	*bla*$_{\text{CMY-4}}$
	NODE_53_length_13156_cov_27.548008	*armA*	*armA*
		mph(E)	*mph(E)*
		msr(E)	*msr(E)*
	NODE_63_length_5366_cov_36.838519	*aph(3'')-Ib*	*aph(3'')-Ib*
		aph(6)-Id	*aph(6)-Id*
		sul2	*sul2*
	NODE_67_length_4392_cov_67.855569	*aac(3)-IId*	*aac(3)-IId*
	NODE_68_length_4377_cov_33.960706	*bla*$_{\text{DHA-1}}$	
		bla$_{\text{DHA-24}}$	
		bla$_{\text{DHA-7}}$	*ble-*$_{\text{MBL}}$
		bla$_{\text{NDM-1}}$	*bla*$_{\text{NDM-1}}$
	NODE_72_length_344cov_46.143030	*ARR-2*	*arr-2*
		ere(A)	*ere(A)*
	NODE_74_length_2629_cov_46.387690	*aac(6')-Ib*	*aac(6')-Ib-AKT*

		aac(6')-Ib-cr	
		*bla*OXA-9	*bla*OXA-9
	NODE_78_length_2158_cov_98.783358	*bla*CTX-M-15	*bla*CTX-M-15
	NODE_79_length_2106_cov_54.118747	*cmlA1*	*cmlA5*
	NODE_8_length_251432_cov_24.701904	*oqxA*	*oqxA6*
		OqxA	
		oqxB	*oqxB20*
		OqxB	
	NODE_8length_1746_cov_37.643607	*catA1*	*catA1*
	NODE_97_length_1044_cov_48.693566	*qacE*	
		sul1	
		sul1	*sul1*
SRR5168489	NODE_105_length_684_cov_55.642729	*bla*TEM-104	
		*bla*TEM-126	
		*bla*TEM-148	
		*bla*TEM-176	
		*bla*TEM-198	
		*bla*TEM-1B	
		*bla*TEM-207	
		*bla*TEM-217	
		*bla*TEM-220	
		*bla*TEM-230	
		*bla*TEM-234	
		*bla*TEM-30	
		*bla*TEM-70	
	NODE_2_length_350548_cov_27.332469	*fosA*	*fosA6*
	NODE_22_length_80338_cov_23.147062	*bla*SHV-106	
		*bla*SHV-172	
		*bla*SHV-28	*bla*SHV-28
		*bla*SHV-40	
		*bla*SHV-56	
		*bla*SHV-76	
		*bla*SHV-79	
		*bla*SHV-85	
		*bla*SHV-89	
	NODE_30_length_41954_cov_32.851077	*dfrA1*	*dfrA1*
			sat2_gen
	NODE_36_length_25502_cov_42.799133	*bla*CMY-4	*bla*CMY-4
	NODE_40_length_21478_cov_36.014894	*catA1*	*catA1*
	NODE_47_length_13156_cov_25.659222	*armA*	*armA*
		mph(E)	*mph(E)*
		msr(E)	*msr(E)*

	NODE_55_length_5366_cov_34.957244	*aph(3")-Ib*	*aph(3")-Ib*
		aph(6)-Id	*aph(6)-Id*
		sul2	*sul2*
	NODE_59_length_4377_cov_32.016235	*bla*$_{DHA-1}$	
		bla$_{DHA-24}$	
		bla$_{DHA-7}$	*ble-*$_{MBL}$
		bla$_{NDM-1}$	*bla*$_{NDM-1}$
	NODE_60_length_4050_cov_55.171043	*aac(6')-Ib*	*aac(6')-Ib-AKT*
		aac(6')-Ib-cr	
		aadA1	*aadA1*
		bla$_{OXA-9}$	*bla*$_{OXA-9}$
	NODE_63_length_344cov_47.751358	*ARR-2*	*arr-2*
		ere(A)	*ere(A)*
	NODE_64_length_2935_cov_46.168091	*aac(3)-IId*	*aac(3)-IId*
	NODE_65_length_2712_cov_90.916054	*bla*$_{CTX-M-15}$	*bla*$_{CTX-M-15}$
	NODE_69_length_2106_cov_46.274886	*cmlA1*	*cmlA5*
	NODE_7_length_251432_cov_24.795050	*oqxA*	*oqxA6*
		OqxA	
		oqxB	*oqxB20*
		OqxB	
	NODE_88_length_1044_cov_52.071974	*qacE*	
		sul1	
		sul1	
		sul1	*sul1*
SRR5168490	NODE_10_length_159039_cov_20.112559	*fosA*	*fosA_gen*
	NODE_2_length_730176_cov_19.927136	*OqxA*	
		oqxA	*oqxA6*
		OqxB	
		oqxB	*oqxB19*
	NODE_22_length_35965_cov_23.469390	*aac(6')-Ib-cr*	*aac(6')-Ib-D181Y*
		aac(6')-Ib-cr	
		ARR-3	*arr-3*
		bla$_{OXA-1}$	*bla*$_{OXA-1}$
		catB3	*catB3*
		qacE	
		sul1	*sul1*
	NODE_26_length_23865_cov_20.661977	*bla*$_{TEM-1A}$	*bla*$_{TEM-150}$
	NODE_27_length_1703cov_21.986512	*aph(3')-VI*	*aph(3')-VI*
		bla$_{CTX-M-15}$	*bla*$_{CTX-M-15}$
		bla$_{NDM-1}$	*bla*$_{NDM-1}$
			ble-$_{MBL}$

		qnrS1	*qnrS1*
	NODE_35_length_2260_cov_21.000000	*aadA1*	*aadA1*
		bla$_{OXA-9}$	*bla*$_{OXA-9}$
	NODE_6_length_391609_cov_18.249682	*bla*$_{SHV-11}$	*bla*$_{SHV-11}$
		bla$_{SHV-67}$	
SRR5168491	NODE_12_length_159039_cov_20.341623	*fosA*	*fosA_gen*
	NODE_24_length_35965_cov_24.408170	*aac(6')-Ib-cr*	*aac(6')-Ib-D181Y*
		aac(6')-Ib-cr	
		ARR-3	*arr-3*
		bla$_{OXA-1}$	*bla*$_{OXA-1}$
		catB3	*catB3*
		qacE	
		sul1	*sul1*
	NODE_3_length_392046_cov_18.711958	*bla*$_{SHV-11}$	*bla*$_{SHV-11}$
		bla$_{SHV-67}$	
	NODE_30_length_23865_cov_20.650560	*bla*$_{TEM-1A}$	*bla*$_{TEM-150}$
	NODE_33_length_1703cov_21.258045	*aph(3')-VI*	*aph(3')-VI*
		bla$_{CTX-M-15}$	*bla*$_{CTX-M-15}$
		bla$_{NDM-1}$	*bla*$_{NDM-1}$
			ble-$_{MBL}$
		qnrS1	*qnrS1*
	NODE_4length_2260_cov_20.788561	*aadA1*	*aadA1*
		bla$_{OXA-9}$	*bla*$_{OXA-9}$
	NODE_7_length_262377_cov_19.024805	*OqxA*	
		oqxA	*oqxA6*
		OqxB	
		oqxB	*oqxB19*
SRR5168492	NODE_2_length_490419_cov_22.177580	*OqxA*	
		oqxA	*oqxA6*
		OqxB	
		oqxB	*oqxB20*
	NODE_25_length_40298_cov_24.730876	*aph(3'')-Ib*	*aph(3'')-Ib*
		aph(6)-Id	*aph(6)-Id*
		sul2	*sul2*
	NODE_29_length_27938_cov_32.687678	*tet(A)*	*tet(A)*
	NODE_2length_7023cov_21.661403	*aac(6')-Ib*	*aac(6')-Ib-AKT*
		aac(6')-Ib-cr	
	NODE_33_length_1926cov_23.786506	*bla*$_{NDM-1}$	*bla*$_{NDM-1}$
			ble-$_{MBL}$
	NODE_35_length_865cov_26.515368	*dfrA14*	*dfrA14*
	NODE_37_length_5620_cov_25.167304	*qnrB9*	*qnrB9*

	NODE_4_length_367739_cov_23.566595	*fosA*	*fosA6*
	NODE_40_length_3815_cov_25.323482	*bla*$_{\text{CTX-M-15}}$	*bla*$_{\text{CTX-M-15}}$
	NODE_49_length_1859_cov_25.398961	*bla*$_{\text{OXA-1}}$	*bla*$_{\text{OXA-1}}$
		catB3	
		catB3	
	NODE_4length_3485_cov_21.591721	*mph(A)*	*mph(A)*
	NODE_5_length_344160_cov_22.923435	*bla*$_{\text{SHV-182}}$	*bla*$_{\text{SHV-158}}$
	NODE_52_length_1649_cov_21.946781	*catA2*	*catA2*
SRR5168493	NODE_2_length_490419_cov_22.695989	*oqxA*	*oqxA6*
		OqxA	
		oqxB	*oqxB20*
		OqxB	
	NODE_25_length_40298_cov_25.716711	*aph(3'')-Ib*	*aph(3'')-Ib*
		aph(6)-Id	*aph(6)-Id*
		sul2	*sul2*
	NODE_29_length_27938_cov_32.458955	*tet(A)*	*tet(A)*
	NODE_32_length_1926cov_23.328891	*bla*$_{\text{NDM-1}}$	*bla*$_{\text{NDM-1}}$
			ble-$_{\text{MBL}}$
	NODE_34_length_11777_cov_24.305751	*aac(6')-Ib*	*aac(6')-Ib-AKT*
		aac(6')-Ib-cr	
	NODE_35_length_8762_cov_23.924609	*dfrA14*	*dfrA14*
	NODE_37_length_5620_cov_26.558893	*qnrB9*	*qnrB9*
	NODE_4_length_367739_cov_24.128203	*fosA*	*fosA6*
	NODE_40_length_3815_cov_28.330803	*bla*$_{\text{CTX-M-15}}$	*bla*$_{\text{CTX-M-15}}$
	NODE_49_length_1859_cov_24.493072	*bla*$_{\text{OXA-1}}$	*bla*$_{\text{OXA-1}}$
		catB3	
		catB3	
	NODE_4length_3485_cov_25.382668	*mph(A)*	*mph(A)*
	NODE_5_length_365233_cov_24.071527	*bla*$_{\text{SHV-182}}$	*bla*$_{\text{SHV-158}}$
	NODE_52_length_1649_cov_21.989488	*catA2*	*catA2*
SRR5168496	NODE_13_length_185424_cov_16.462819	*oqxA*	*oqxA6*
		OqxA	
		oqxB	*oqxB20*
		OqxB	
	NODE_3_length_367770_cov_18.445288	*fosA*	*fosA6*
	NODE_43_length_12864_cov_31.529874	*aac(3)-IId*	*aac(3)-IId*
	NODE_48_length_870cov_38.693609	*bla*$_{\text{CMY-6}}$	*bla*$_{\text{CMY-6}}$
	NODE_4length_18255_cov_27.406719	*aph(3'')-Ib*	*aph(3'')-Ib*
		aph(6)-Id	*aph(6)-Id*
		bla$_{\text{TEM-1B}}$	*bla*$_{\text{TEM-1}}$
		sul2	*sul2*
	NODE_52_length_5618_cov_27.237844	*qnrB9*	*qnrB9*

	NODE_53_length_4680_cov_18.886888	*tet(A)*	*tet(A)*
	NODE_54_length_4659_cov_36.631068	*bla*$_{CTX-M-15}$	*bla*$_{CTX-M-15}$
	NODE_56_length_4247_cov_28.112136	*bla*$_{DHA-1}$	
		bla$_{DHA-24}$	
		bla$_{DHA-7}$	*ble-*$_{MBL}$
		bla$_{NDM-1}$	*bla*$_{NDM-1}$
	NODE_62_length_3365_cov_32.964793	*bla*$_{SHV-182}$	*bla*$_{SHV-158}$
	NODE_64_length_2589_cov_35.190902	*aac(3)-IIa*	
		aac(3)-IIa	
	NODE_6length_3485_cov_17.563431	*mph(A)*	*mph(A)*
	NODE_75_length_1859_cov_27.732679	*bla*$_{OXA-1}$	*bla*$_{OXA-1}$
		catB3	
		catB3	
	NODE_80_length_1545_cov_44.899154	*dfrA14*	*dfrA14*
	NODE_89_length_1157_cov_34.558252	*rmtC*	*rmtC*
	NODE_95_length_785_cov_48.960486	*qacE*	
		sul1	
	NODE_98_length_707_cov_97.913793	*aac(6')-Ib-cr*	*aac(3)-IIe*
		aac(6')-Ib-cr	*aac(6')-Ib-AKT*
SRR5168497	NODE_3_length_367638_cov_19.296261	*fosA*	*fosA6*
	NODE_39_length_18255_cov_28.659036	*aph(3'')-Ib*	*aph(3'')-Ib*
		aph(6)-Id	*aph(6)-Id*
		bla$_{TEM-1B}$	*bla*$_{TEM-1}$
		sul2	*sul2*
	NODE_46_length_870cov_41.829251	*bla*$_{CMY-6}$	*bla*$_{CMY-6}$
	NODE_4length_12515_cov_31.198741	*aac(3)-IId*	*aac(3)-IId*
	NODE_50_length_5618_cov_28.739574	*qnrB9*	*qnrB9*
	NODE_53_length_4680_cov_19.894575	*tet(A)*	*tet(A)*
	NODE_54_length_4659_cov_37.295234	*bla*$_{CTX-M-15}$	*bla*$_{CTX-M-15}$
	NODE_56_length_4247_cov_29.516748	*bla*$_{DHA-1}$	
		bla$_{DHA-24}$	
		bla$_{DHA-7}$	*ble-*$_{MBL}$
		bla$_{NDM-1}$	*bla*$_{NDM-1}$
	NODE_63_length_295cov_39.207861	*bla*$_{SHV-182}$	*bla*$_{SHV-158}$
	NODE_64_length_2589_cov_35.977254	*aac(3)-IIa*	
		aac(3)-IIa	
	NODE_6length_3485_cov_20.351102	*mph(A)*	*mph(A)*
	NODE_74_length_173cov_23.877182	*bla*$_{OXA-1}$	*bla*$_{OXA-1}$
		catB3	
		catB3	
	NODE_77_length_1545_cov_42.753173	*dfrA14*	*dfrA14*
	NODE_86_length_1157_cov_31.107767	*rmtC*	*rmtC*

	NODE_87_length_1134_cov_40.327706	*qacE*	
		sul1	*sul1*
	NODE_90_length_953_cov_92.220339	*aac(6')-Ib-cr*	*aac(3)-IIe*
		aac(6')-Ib3	*aac(6')-Ib-AKT*
	NODE_length_803036_cov_18.898132	*OqxA*	
		oqxA	*oqxA6*
		OqxB	
		oqxB	*oqxB20*
SRR5168498	NODE_38_length_18255_cov_27.927902	*aph(3'')-Ib*	*aph(3'')-Ib*
		aph(6)-Id	*aph(6)-Id*
		bla$_{TEM-1B}$	*bla*$_{TEM-1}$
		sul2	*sul2*
	NODE_4_length_367739_cov_18.406662	*fosA*	*fosA6*
	NODE_46_length_870cov_41.146606	*bla*$_{CMY-6}$	*bla*$_{CMY-6}$
	NODE_4length_12515_cov_30.448499	*aac(3)-IId*	*aac(3)-IId*
	NODE_50_length_5618_cov_32.327445	*qnrB9*	*qnrB9*
	NODE_52_length_4680_cov_17.770042	*tet(A)*	*tet(A)*
	NODE_53_length_4659_cov_37.900927	*bla*$_{CTX-M-15}$	*bla*$_{CTX-M-15}$
	NODE_55_length_4247_cov_27.784951	*bla*$_{DHA-1}$	
		bla$_{DHA-24}$	
		bla$_{DHA-7}$	*ble-*$_{MBL}$
		bla$_{NDM-1}$	*bla*$_{NDM-1}$
	NODE_63_length_3485_cov_16.738535	*mph(A)*	*mph(A)*
	NODE_66_length_295cov_34.044972	*bla*$_{SHV-182}$	*bla*$_{SHV-158}$
	NODE_67_length_2589_cov_33.902924	*aac(3)-IIa*	
		aac(3)-IIa	
	NODE_74_length_1859_cov_26.566975	*bla*$_{OXA-1}$	*bla*$_{OXA-1}$
		catB3	
		catB3	
	NODE_77_length_1545_cov_41.263047	*dfrA14*	*dfrA14*
	NODE_86_length_1157_cov_38.839806	*rmtC*	*rmtC*
	NODE_88_length_976_cov_82.194346	*aac(6')-Ib-cr*	*aac(3)-IIe*
		aac(6')-Ib3	*aac(6')-Ib-AKT*
	NODE_93_length_785_cov_46.196049	*qacE*	
		sul1	
	NODE_length_803394_cov_18.236793	*OqxA*	
		oqxA	*oqxA6*
		OqxB	
		oqxB	*oqxB20*
SRR5168509	NODE_15_length_15995cov_14.510699	*fosA*	*fosA_gen*
	NODE_2_length_392046_cov_12.376984	*bla*$_{SHV-11}$	*bla*$_{SHV-11}$
		bla$_{SHV-67}$	

	NODE_44_length_17272_cov_12.583844	*aadA1*	*aadA1*
		bla$_{\text{OXA-9}}$	*bla*$_{\text{OXA-9}}$
		bla$_{\text{TEM-1A}}$	*bla*$_{\text{TEM-150}}$
	NODE_48_length_6944_cov_13.010709	*aph(3')-VI*	*aph(3')-VI*
		bla$_{\text{NDM-1}}$	*bla*$_{\text{NDM-1}}$
			ble-$_{\text{MBL}}$
	NODE_49_length_5668_cov_34.894243	*qnrS1*	*qnrS1*
	NODE_50_length_5546_cov_12.615243	*aac(6')-Ib-cr*	*aac(6')-Ib-D181Y*
		aac(6')-Ib-cr	
		ARR-3	*arr-3*
		bla$_{\text{OXA-1}}$	*bla*$_{\text{OXA-1}}$
		catB3	*catB3*
		qacE	
		sul1	*sul1*
	NODE_53_length_4673_cov_16.058513	*bla*$_{\text{CTX-M-15}}$	*bla*$_{\text{CTX-M-15}}$
	NODE_57_length_4007_cov_15.685825	*tet(A)*	*tet(A)*
	NODE_58_length_3779_cov_22.933461	*floR*	*floR*
	NODE_5length_5534_cov_16.539671	*sul2*	*sul2*
	NODE_6_length_262438_cov_12.800974	*OqxA*	
		oqxA	*oqxA6*
		OqxB	
		oqxB	*oqxB19*
	NODE_62_length_2438_cov_21.073994	*bla*$_{\text{LAP-2}}$	*bla*$_{\text{LAP-2}}$
	NODE_72_length_927_cov_27.206250	*dfrA14*	*dfrA14*
SRR5168510	NODE_10_length_159039_cov_18.570391	*fosA*	*fosA_gen*
	NODE_13_length_111050_cov_16.993996	*bla*$_{\text{SHV-11}}$	*bla*$_{\text{SHV-11}}$
		bla$_{\text{SHV-67}}$	
	NODE_2_length_729158_cov_18.038387	*OqxA*	
		oqxA	*oqxA6*
		OqxB	
		oqxB	*oqxB19*
	NODE_34_length_23865_cov_18.174994	*bla*$_{\text{TEM-1A}}$	*bla*$_{\text{TEM-150}}$
	NODE_37_length_6944_cov_14.907731	*aph(3')-VI*	*aph(3')-VI*
		bla$_{\text{NDM-1}}$	*bla*$_{\text{NDM-1}}$
			ble-$_{\text{MBL}}$
	NODE_38_length_5668_cov_49.652409	*qnrS1*	*qnrS1*
	NODE_39_length_5662_cov_25.786811	*sul2*	*sul2*
	NODE_40_length_5546_cov_17.800517	*aac(6')-Ib-cr*	*aac(6')-Ib-AKT*
		aac(6')-Ib3	
		ARR-3	*arr-3*
		bla$_{\text{OXA-1}}$	*bla*$_{\text{OXA-1}}$

		catB3	*catB3*
		qacE	
		sul1	*sul1*
	NODE_45_length_4007_cov_22.960825	*tet(A)*	*tet(A)*
	NODE_48_length_2786_cov_23.489658	*floR*	*floR*
	NODE_4length_4673_cov_20.775187	*bla*CTX-M-15	*bla*CTX-M-15
	NODE_53_length_2260_cov_16.313643	*aadA1*	*aadA1*
		*bla*OXA-9	*bla*OXA-9
	NODE_5length_2438_cov_27.990480	*bla*LAP-2	*bla*LAP-2
	NODE_63_length_927_cov_27.592500	*dfrA14*	*dfrA14*
SRR5168511	NODE_10_length_178182_cov_16.644980	*bla*SHV-11	*bla*SHV-11
		*bla*SHV-67	
	NODE_13_length_159039_cov_17.773906	*fosA*	*fosA_gen*
	NODE_2_length_729352_cov_17.526623	*OqxA*	
		oqxA	*oqxA6*
		OqxB	
		oqxB	*oqxB19*
	NODE_33_length_23865_cov_17.585433	*bla*TEM-1A	*bla*TEM-150
	NODE_36_length_12696_cov_27.356035	*floR*	*floR*
		sul2	*sul2*
	NODE_38_length_6944_cov_15.367757	*aph(3')-VI*	*aph(3')-VI*
		*bla*NDM-1	*bla*NDM-1
			*ble-*MBL
	NODE_39_length_5668_cov_45.581123	*qnrS1*	*qnrS1*
	NODE_40_length_5546_cov_16.851449	*aac(6')-Ib-cr*	*aac(6')-Ib-D181Y*
		aac(6')-Ib-cr	
		ARR-3	*arr-3*
		*bla*OXA-1	*bla*OXA-1
		catB3	*catB3*
		qacE	
		sul1	*sul1*
	NODE_45_length_4007_cov_22.013660	*tet(A)*	*tet(A)*
	NODE_4length_4673_cov_19.236472	*bla*CTX-M-15	*bla*CTX-M-15
	NODE_50_length_2438_cov_24.672003	*bla*LAP-2	*bla*LAP-2
	NODE_52_length_2260_cov_18.731364	*aadA1*	*aadA1*
		*bla*OXA-9	*bla*OXA-9
	NODE_59_length_927_cov_34.068750	*dfrA14*	*dfrA14*
SRR5168517	NODE_29_length_56093_cov_10.589483	*bla*SHV-36	*bla*SHV-36
	NODE_39_length_33365_cov_20.576058	*bla*NDM-1	*bla*NDM-1
			*ble-*MBL
	NODE_4_length_287746_cov_11.342154	*fosA*	*fosA_gen*

	NODE_5_length_285449_cov_10.655961	*OqxA*	
		oqxA	*oqxA6*
		OqxB	
		oqxB	*oqxB24*
	NODE_55_length_1233cov_18.581613	*aph(3'')-Ib*	*aph(3'')-Ib*
		aph(6)-Id	*aph(6)-Id*
		bla$_{CTX-M-15}$	*bla*$_{CTX-M-15}$
		bla$_{TEM-1B}$	*bla*$_{TEM-1}$
		sul2	*sul2*
	NODE_58_length_9817_cov_14.605366	*aac(3)-IId*	*aac(3)-IId*
	NODE_60_length_882cov_21.254888	*tet(B)*	*tet(B)*
	NODE_66_length_6219_cov_13.605877	*dfrA30*	*dfrA30*
		qacE	
	NODE_69_length_5575_cov_21.491373	*dfrA14*	*dfrA14*
	NODE_86_length_2439_cov_17.434256	*aac(6')-Ib-cr*	*aac(6')-Ib-D181Y*
		aac(6')-Ib-cr	
		bla$_{OXA-1}$	*bla*$_{OXA-1}$
		catB3	
		catB3	
	NODE_9length_1979_cov_27.753240	*qnrB1*	*qnrB1*
SRR5168518	NODE_14_length_113612_cov_12.750372	*bla*$_{SHV-12}$	*bla*$_{SHV-12}$
	NODE_20_length_74099_cov_13.667658	*fosA*	*fosA_gen*
	NODE_36_length_3331cov_29.289597	*tet(B)*	*tet(B)*
	NODE_4_length_276119_cov_12.970438	*oqxA*	*oqxA6*
		OqxA	
		oqxB	*oqxB24*
		OqxB	
	NODE_43_length_16963_cov_23.277857	*bla*$_{NDM-1}$	*bla*$_{NDM-1}$
			ble-$_{MBL}$
	NODE_49_length_1233cov_22.140528	*aph(3'')-Ib*	*aph(3'')-Ib*
		aph(6)-Id	*aph(6)-Id*
		bla$_{CTX-M-15}$	*bla*$_{CTX-M-15}$
		bla$_{TEM-1B}$	*bla*$_{TEM-1}$
		sul2	*sul2*
	NODE_52_length_9817_cov_18.132095	*aac(3)-IId*	*aac(3)-IId*
	NODE_60_length_6219_cov_15.015923	*dfrA30*	*dfrA30*
		qacE	
	NODE_62_length_5575_cov_25.787628	*dfrA14*	*dfrA14*
	NODE_89_length_1979_cov_35.301296	*qnrB1*	*qnrB1*
	NODE_8length_2439_cov_19.159170	*aac(6')-Ib-cr*	*aac(6')-Ib-D181Y*
		aac(6')-Ib-cr	

		bla$_{\text{OXA-1}}$	*bla*$_{\text{OXA-1}}$
		catB3	
		catB3	
SRR5168519	NODE_13_length_113612_cov_13.970824	*bla*$_{\text{SHV-36}}$	*bla*$_{\text{SHV-36}}$
	NODE_2_length_671547_cov_14.222777	*oqxA*	*oqxA6*
		OqxA	
		oqxB	*oqxB24*
		OqxB	
	NODE_33_length_3331cov_32.538181	*tet(B)*	*tet(B)*
	NODE_42_length_16963_cov_24.724638	*bla*$_{\text{NDM-1}}$	*bla*$_{\text{NDM-1}}$
			ble-$_{\text{MBL}}$
	NODE_45_length_14782_cov_34.738246	*aac(3)-IId*	*aac(3)-IId*
		qnrB1	*qnrB1*
	NODE_46_length_14617_cov_15.361905	*fosA*	*fosA_gen*
	NODE_50_length_1233cov_23.888889	*aph(3")-Ib*	*aph(3")-Ib*
		aph(6)-Id	*aph(6)-Id*
		bla$_{\text{CTX-M-15}}$	*bla*$_{\text{CTX-M-15}}$
		bla$_{\text{TEM-1B}}$	*bla*$_{\text{TEM-1}}$
		sul2	*sul2*
	NODE_63_length_5575_cov_24.709802	*dfrA14*	*dfrA14*
	NODE_6length_6219_cov_16.849475	*dfrA30*	*dfrA30*
		qacE	
	NODE_83_length_2439_cov_21.363754	*aac(6')-Ib-cr*	*aac(6')-Ib-D181Y*
		aac(6')-Ib-cr	
		bla$_{\text{OXA-1}}$	*bla*$_{\text{OXA-1}}$
		catB3	
		catB3	
SRR2724077	NODE_16_length_129117_cov_14.123746	*OqxA*	
		oqxA	*oqxA8*
		OqxB	
		oqxB	*oqxB12*
	NODE_42_length_18244_cov_25.315394	*bla*$_{\text{TEM-1B}}$	*bla*$_{\text{TEM-1}}$
	NODE_4length_1980cov_102.379587	*dfrA14*	*dfrA14*
	NODE_54_length_5490_cov_109.017155	*qnrS1*	*qnrS1*
	NODE_58_length_1938_cov_132.405301	*aac(6')-Ib-cr*	*aac(6')-Ib-G*
		aac(6')-Ib3	
		bla$_{\text{VIM-1}}$	*bla*$_{\text{VIM-1}}$
	NODE_70_length_1119_cov_83.164315	*bla*$_{\text{SHV-12}}$	*bla*$_{\text{SHV-12}}$
	NODE_length_369238_cov_15.668336	*fosA5*	*fosA5*
SRR2724078	NODE_42_length_1875cov_42.245060	*bla*$_{\text{KPC-2}}$	*bla*$_{\text{KPC-2}}$
	NODE_45_length_13584_cov_28.791335	*bla*$_{\text{TEM-1A}}$	*bla*$_{\text{TEM-1}}$

	NODE_5_length_270452_cov_21.162675	*fosA*	*fosA6*
	NODE_7_length_251477_cov_19.040589	*oqxA*	*oqxA*
		OqxA	
		oqxB	*oqxB*
		OqxB	
	NODE_75_length_1443_cov_50.067629	*bla*$_{OXA-9}$	*bla*$_{OXA-9}$
	NODE_83_length_1128_cov_164.616384	*aac(6')-Ib*	*aac(6')-Ib-AKT*
		aac(6')-Ib-cr	
	NODE_92_length_722_cov_39.211765	*bla*$_{SHV-12}$	*bla*$_{SHV-187}$
		bla$_{SHV-129}$	
		bla$_{SHV-13}$	
		bla$_{SHV-155}$	
		bla$_{SHV-172}$	
		bla$_{SHV-31}$	
SRR2724081	NODE_104_length_52cov_380.362944	*aac(6')-Ib-cr*	*aac(6')-Ib-AKT*
		aac(6')-Ib3	
	NODE_12_length_137014_cov_106.263750	*bla*$_{SHV-182}$	*bla*$_{SHV-158}$
	NODE_2_length_80308cov_132.470230	*oqxA*	*oqxA6*
		OqxA	
		oqxB	*oqxB20*
		OqxB	
	NODE_3_length_367372_cov_130.294863	*fosA*	*fosA6*
	NODE_38_length_13204_cov_131.304963	*aph(3')-VI*	*aph(3')-VI*
		bla$_{NDM-1}$	*bla*$_{NDM-1}$
			ble-$_{MBL}$
		qnrS1	*qnrS1*
	NODE_3length_28200_cov_136.550173	*aadA2*	*aadA2*
		armA	*armA*
		dfrA12	*dfrA12*
		mph(E)	*mph(E)*
		msr(E)	*msr(E)*
		qacE	
		sul1	*sul1*
	NODE_47_length_6229_cov_139.144871	*tet(A)*	*tet(A)*
	NODE_55_length_2853_cov_116.581438	*aac(3)-IIa*	*aac(3)-IIe*
	NODE_58_length_2405_cov_262.190518	*bla*$_{CTX-M-15}$	*bla*$_{CTX-M-15}$
	NODE_60_length_2260_cov_124.612283	*aadA1*	*aadA1*
		bla$_{OXA-9}$	*bla*$_{OXA-9}$
	NODE_63_length_1860_cov_239.222158	*bla*$_{OXA-1}$	*bla*$_{OXA-1}$
		catB3	
		catB3	

	NODE_73_length_1423_cov_114.653549	*bla*$_{TEM-1A}$	*bla*$_{TEM-150}$
	NODE_77_length_1287_cov_136.849138	*aph(3')-Ia*	*aph(3')-Ia*
SRR2724082	NODE_23_length_59380_cov_23.420080	*aadA2*	*aadA2*
		catA1	*catA1*
		dfrA12	*dfrA12*
		mph(A)	*mph(A)*
		qacE	
		sul1	*sul1*
	NODE_33_length_20625_cov_23.102644	*bla*$_{KPC-2}$	*bla*$_{KPC-2}$
	NODE_40_length_13275_cov_121.403788	*aac(6')-Ib*	*aac(6')-Ib-AKT*
		aac(6')-Ib-cr	
	NODE_63_length_1443_cov_25.876900	*bla*$_{OXA-9}$	*bla*$_{OXA-9}$
	NODE_66_length_1292_cov_22.569099	*aph(3')-Ia*	*aph(3')-Ia*
	NODE_7_length_270452_cov_19.933783	*fosA*	*fosA6*
	NODE_75_length_923_cov_23.992462	*bla*$_{TEM-1A}$	*bla*$_{TEM-1}$
	NODE_79_length_722_cov_43.867227	*bla*$_{SHV-12}$	*bla*$_{SHV-187}$
		bla$_{SHV-129}$	
		bla$_{SHV-13}$	
		bla$_{SHV-155}$	
		bla$_{SHV-172}$	
		bla$_{SHV-31}$	
	NODE_length_804588_cov_19.191631	*OqxA*	
		oqxA	*oqxA*
		OqxB	
		oqxB	*oqxB*
SRR2724083	NODE_112_length_1443_cov_21.386018	*bla*$_{OXA-9}$	*bla*$_{OXA-9}$
	NODE_124_length_1128_cov_138.933067	*aac(6')-Ib*	*aac(6')-Ib-AKT*
		aac(6')-Ib-cr	
	NODE_129_length_923_cov_15.604271	*bla*$_{TEM-1A}$	*bla*$_{TEM-1}$
	NODE_14length_722_cov_36.959664	*bla*$_{SHV-12}$	*bla*$_{SHV-187}$
		bla$_{SHV-129}$	
		bla$_{SHV-13}$	
		bla$_{SHV-155}$	
		bla$_{SHV-172}$	
		bla$_{SHV-31}$	
	NODE_27_length_58008_cov_11.738394	*OqxA*	
		oqxA	*oqxA*
		OqxB	
		oqxB	*oqxB*
	NODE_35_length_42692_cov_16.792764	*bla*$_{KPC-2}$	*bla*$_{KPC-2}$
	NODE_6_length_270452_cov_12.855245	*fosA*	*fosA6*
	NODE_30_length_51149_cov_27.974991	*aadA2*	*aadA2*

		catA1	*catA1*
		dfrA12	*dfrA12*
		qacE	
		sul1	*sul1*
	NODE_48_length_10395_cov_34.682217	*bla*$_{KPC-3}$	*bla*$_{KPC-3}$
	NODE_5_length_37569cov_19.019997	*OqxA*	
		oqxA	*oqxA*
		OqxB	
		oqxB	*oqxB*
	NODE_54_length_4412_cov_23.992532	*mph(A)*	*mph(A)*
	NODE_57_length_2405_cov_40.206760	*aac(6')-Ib-cr*	
		aadA1	*aadA1*
		bla$_{OXA-9}$	*bla*$_{OXA-9}$
	NODE_6_length_270452_cov_20.934679	*fosA*	*fosA6*
SRR2724085	NODE_66_length_1293_cov_28.114923	*aph(3')-Ia*	*aph(3')-Ia*
	NODE_76_length_722_cov_35.600000	*bla*$_{SHV-12}$	*bla*$_{SHV-187}$
		bla$_{SHV-129}$	
		bla$_{SHV-13}$	
		bla$_{SHV-155}$	
		bla$_{SHV-172}$	
		bla$_{SHV-31}$	
	NODE_88_length_547_cov_47.261905	*bla*$_{TEM-150}$	
		bla$_{TEM-171}$	
		bla$_{TEM-1A}$	
		bla$_{TEM-1C}$	
		bla$_{TEM-40}$	
		bla$_{TEM-97}$	
		bla$_{TEM-98}$	
	NODE_92_length_484_cov_98.375350	*aac(6')-Ib*	
		aac(6')-Ib-cr	
SRR2724086	NODE_13_length_159039_cov_15.516688	*fosA*	*fosA_gen*
	NODE_3_length_350410_cov_14.176540	*oqxA*	*oqxA6*
		OqxA	
		oqxB	*oqxB19*
		OqxB	
	NODE_43_length_6686_cov_84.460741	*aph(3'')-Ib*	*aph(3'')-Ib*
		aph(3'')-Ib	
		aph(3'')-Ib	
		aph(3'')-Ib	
		aph(6)-Id	*aph(6)-Id*
	NODE_50_length_3484_cov_73.530235	*mph(A)*	*mph(A)*
	NODE_53_length_2233_cov_104.220323	*aac(6')-Il*	*aac(6')-Il*

		bla$_{\text{VIM-27}}$	*bla*$_{\text{VIM-27}}$
		dfrA1	*dfrA1*
		dfrA1	
		dfrA1	
	NODE_56_length_1537_cov_88.027660	*qacE*	
		sul1	*sul1*
	NODE_60_length_129cov_87.427835	*aph(3')-Ia*	*aph(3')-Ia*
	NODE_67_length_93cov_42.927861	*aac(6')-Ib-cr*	*aac(6')-Ib-AKT*
		aac(6')-Ib3	
	NODE_68_length_896_cov_95.529259	*aadA22*	*aadA1*
		aadA23	
	NODE_8_length_262234_cov_12.640811	*bla*$_{\text{SHV-11}}$	*bla*$_{\text{SHV-11}}$
		bla$_{\text{SHV-67}}$	
SRR2724087	NODE_13_length_159039_cov_46.723426	*fosA*	*fosA_gen*
	NODE_2_length_587927_cov_38.742133	*bla*$_{\text{SHV-11}}$	*bla*$_{\text{SHV-11}}$
		bla$_{\text{SHV-67}}$	
	NODE_4_length_350895_cov_41.812749	*oqxA*	*oqxA6*
		OqxA	
		oqxB	*oqxB19*
		OqxB	
	NODE_44_length_4346_cov_76.886940	*aac(6')-Il*	*aac(6')-Il*
		aadA22	*aadA1*
		aadA23	
		bla$_{\text{VIM-27}}$	*bla*$_{\text{VIM-27}}$
		dfrA1	*dfrA1*
		dfrA1	
		dfrA1	
SRR2724088	NODE_33_length_35434_cov_71.547625	*bla*$_{\text{TEM-1A}}$	*bla*$_{\text{TEM-1}}$
	NODE_4_length_270452_cov_49.780742	*fosA*	*fosA6*
	NODE_48_length_20517_cov_57.911721	*bla*$_{\text{KPC-2}}$	*bla*$_{\text{KPC-2}}$
	NODE_54_length_16866_cov_48.642332	*aadA2*	*aadA2*
		catA1	*catA1*
		dfrA12	*dfrA12*
		mph(A)	*mph(A)*
		qacE	
		sul1	*sul1*
	NODE_86_length_1443_cov_66.715805	*bla*$_{\text{OXA-9}}$	*bla*$_{\text{OXA-9}}$
	NODE_87_length_1428_cov_85.730976	*bla*$_{\text{SHV-12}}$	*bla*$_{\text{SHV-12}}$
	NODE_99_length_1128_cov_382.700300	*aac(6')-Ib*	*aac(6')-Ib-AKT*
		aac(6')-Ib-cr	
	NODE_9length_1292_cov_49.786266	*aph(3')-Ia*	*aph(3')-Ia*
	NODE_length_484358_cov_44.346190	*OqxA*	

		oqxA	*oqxA*
		OqxB	
		oqxB	*oqxB*
SRR2724089	NODE_25_length_59380_cov_41.336996	*aadA2*	*aadA2*
		catA1	*catA1*
		dfrA12	*dfrA12*
		mph(A)	*mph(A)*
		qacE	
		sul1	*sul1*
	NODE_36_length_20518_cov_23.441911	*bla*$_{\text{KPC-2}}$	*bla*$_{\text{KPC-2}}$
	NODE_43_length_13275_cov_202.181320	*aac(6')-Ib*	*aac(6')-Ib-AKT*
		aac(6')-Ib-cr	
	NODE_6_length_270452_cov_19.483921	*fosA*	*fosA6*
	NODE_72_length_1443_cov_30.017477	*bla*$_{\text{OXA-9}}$	*bla*$_{\text{OXA-9}}$
	NODE_77_length_1292_cov_42.852361	*aph(3')-Ia*	*aph(3')-Ia*
	NODE_86_length_923_cov_25.143216	*bla*$_{\text{TEM-1A}}$	*bla*$_{\text{TEM-1}}$
	NODE_92_length_722_cov_42.363025	*bla*$_{\text{SHV-12}}$	*bla*$_{\text{SHV-187}}$
		bla$_{\text{SHV-129}}$	
		bla$_{\text{SHV-13}}$	
		bla$_{\text{SHV-155}}$	
		bla$_{\text{SHV-172}}$	
		bla$_{\text{SHV-31}}$	
	NODE_length_804616_cov_18.636311	*oqxA*	*oqxA*
		OqxA	
		oqxB	*oqxB*
		OqxB	
SRR2724090	NODE_108_length_13232_cov_14.763220	*aac(6')-Ib*	*aac(6')-Ib-AKT*
		aac(6')-Ib-cr	
	NODE_124_length_11167_cov_3.347283	*aadA2*	*aadA2*
		catA1	*catA1*
		dfrA12	*dfrA12*
		qacE	
	NODE_143_length_7033_cov_2.903852	*mph(A)*	*mph(A)*
		sul1	
		sul1	
		sul1	
		sul1	
		sul1	
		sul1	
		sul1	
		sul1	
	NODE_214_length_1428_cov_6.333590	*bla*$_{\text{SHV-12}}$	*bla*$_{\text{SHV-12}}$

	NODE_220_length_1292_cov_2.894421	*aph(3')-Ia*	*aph(3')-Ia*
	NODE_24length_923_cov_3.570352	*bla*$_{\text{TEM-1A}}$	*bla*$_{\text{TEM-1}}$
	NODE_253_length_762_cov_2.462992	*bla*$_{\text{OXA-9}}$	
	NODE_3length_53009_cov_5.144492	*oqxA*	*oqxA*
		OqxA	
		oqxB	*oqxB*
		OqxB	
	NODE_45_length_43399_cov_4.988075	*fosA*	*fosA6*
	NODE_55_length_3911cov_4.222476	*bla*$_{\text{KPC-2}}$	*bla*$_{\text{KPC-2}}$
SRR2724091	NODE_2_length_774662_cov_109.376676	*OqxA*	
		oqxA	*oqxA9*
		OqxB	
		oqxB	*oqxB25*
	NODE_28_length_21148_cov_101.278483	*bla*$_{\text{NDM-1}}$	*bla*$_{\text{NDM-1}}$
		rmtC	*rmtC*
		sul1	*sul1*
		sul1	*ble*-$_{\text{MBL}}$
	NODE_3_length_265747_cov_109.949748	*fosA*	*fosA_gen*
	NODE_30_length_17133_cov_142.197401	*bla*$_{\text{TEM-1B}}$	*bla*$_{\text{TEM-1}}$
	NODE_33_length_15304_cov_79.330764	*tet(A)*	*tet(A)*
	NODE_38_length_8327_cov_168.555854	*bla*$_{\text{CTX-M-15}}$	*bla*$_{\text{CTX-M-15}}$
	NODE_48_length_3777_cov_131.626301	*tet(D)*	*tet(D)*
	NODE_52_length_2848_cov_80.358324	*aac(3)-IIa*	*aac(3)-IIe*
	NODE_54_length_2439_cov_84.169550	*aac(6')-Ib-cr*	*aac(6')-Ib-D181Y*
		aac(6')-Ib-cr	
		bla$_{\text{OXA-1}}$	*bla*$_{\text{OXA-1}}$
		catB3	
	NODE_54_length_2439_cov_84.169550	*catB3*	
	NODE_55_length_178cov_377.970375	*bla*$_{\text{OXA-181}}$	*bla*$_{\text{OXA-181}}$
	NODE_length_2830080_cov_94.441012	*bla*$_{\text{SHV-40}}$	*bla*$_{\text{SHV-187}}$
		bla$_{\text{SHV-56}}$	
		bla$_{\text{SHV-79}}$	
		bla$_{\text{SHV-85}}$	
		bla$_{\text{SHV-89}}$	
SRR2724092	NODE_136_length_7044_cov_5.933063	*qnrB1*	*qnrB1*
	NODE_138_length_695cov_4.312573	*tet(A)*	*tet(A)*
	NODE_143_length_5909_cov_4.500865	*aph(3'')-Ib*	*aph(3'')-Ib*
		aph(6)-Id	*aph(6)-Id*
		bla$_{\text{TEM-1B}}$	*bla*$_{\text{TEM-1}}$
		sul2	*sul2*
	NODE_164_length_3867_cov_5.604545	*dfrA14*	*dfrA14*

	NODE_17_length_86583_cov_5.408601	*fosA*	*fosA_gen*
	NODE_176_length_223cov_8.227311	*bla*OXA-48	*bla*OXA-48
	NODE_17length_2853_cov_4.634996	*aac(3)-IIa*	*aac(3)-IIe*
	NODE_180_length_1947_cov_6.362637	*bla*CTX-M-15	*bla*CTX-M-15
	NODE_194_length_1257_cov_4.398230	*aac(6')-Ib-cr*	*aac(6')-Ib-D181Y*
		aac(6')-Ib-cr	
	NODE_197_length_1189_cov_3.096987	*catB3*	
		catB3	
	NODE_54_length_32610_cov_5.418865	*OqxA*	
		oqxA	*oqxA3*
		OqxB	
		oqxB	*oqxB11*
	NODE_5length_3483cov_3.686203	*bla*SHV-76	*bla*SHV-76
SRR2724093	NODE_19_length_111844_cov_20.032505	*bla*SHV-11	*bla*SHV-11
		*bla*SHV-67	
	NODE_20_length_107485_cov_14.344788		*ble-*MBL
		*bla*NDM-1	*bla*NDM-1
	NODE_25_length_95402_cov_23.298442	*fosA*	*fosA_gen*
	NODE_27_length_85268_cov_23.023829	*oqxA*	*oqxA6*
		OqxA	
		oqxB	*oqxB19*
		OqxB	
	NODE_35_length_41812_cov_14.495316	*bla*CMY-6	*bla*CMY-6
	NODE_47_length_13540_cov_13.511847	*qnrB1*	*qnrB1*
		tet(A)	*tet(A)*
	NODE_50_length_8606_cov_11.369563	*aph(3'')-Ib*	*aph(3'')-Ib*
		aph(6)-Id	*aph(6)-Id*
		*bla*TEM-1B	*bla*TEM-1
		sul2	*sul2*
	NODE_52_length_5480_cov_12.801777	*dfrA14*	*dfrA14*
	NODE_53_length_5329_cov_16.028941	*qacE*	
		rmtC	*rmtC*
		sul1	*sul1*
	NODE_55_length_4299_cov_15.412601	*bla*CTX-M-15	*bla*CTX-M-15
	NODE_62_length_2338_cov_13.230429	*aac(6')-Ib-cr*	*aac(6')-Ib-D181Y*
		aac(6')-Ib-cr	
		*bla*OXA-1	*bla*OXA-1
		catB3	
		catB3	
	NODE_6length_2753_cov_12.009342	*aac(3)-IIa*	*aac(3)-IIe*
	NODE_128_length_3513_cov_5.766391	*bla*TEM-1B	*bla*TEM-1

SRR2724096		*tet(D)*	*tet(D)*
	NODE_138_length_3116_cov_101.527601	*qnrB19*	*qnrB19*
	NODE_142_length_2455_cov_190.324313	*bla*$_{\text{KPC-2}}$	*bla*$_{\text{KPC-2}}$
	NODE_153_length_129cov_7.334192	*aph(3')-Ia*	*aph(3')-Ia*
	NODE_159_length_1094_cov_2.023785	*qacE*	
		sul1	
		sul1	
		sul1	
		sul1	
		sul1	
		sul1	
	NODE_163_length_976_cov_2.665489	*dfrA12*	*dfrA12*
		dfrA12	
		dfrA12	
	NODE_19_length_83990_cov_4.685845	*OqxA*	
		oqxA	*oqxA*
		OqxB	
		oqxB	*oqxB*
	NODE_3length_61729_cov_5.019983	*fosA*	*fosA6*
	NODE_7_length_150169_cov_4.690100	*bla*$_{\text{SHV-182}}$	*bla*$_{\text{SHV-158}}$
	NODE_88_length_9660_cov_6.978915	*aadA1*	*aadA1*
		aadA2b	*aadA2*
		cmlA1	*cmlA1*
		sul3	*sul3*
	NODE_92_length_8275_cov_185.664948	*aph(3')-VIa*	*aph(3')-VIa*
SRR2724097	NODE_24_length_89480_cov_36.359373	*OqxA*	
		oqxA	*oqxA6*
		OqxB	
		oqxB	*oqxB20*
	NODE_26_length_84578_cov_37.143939	*fosA*	*fosA6*
	NODE_3_length_339860_cov_30.792535	*bla*$_{\text{SHV-182}}$	*bla*$_{\text{SHV-158}}$
	NODE_44_length_890cov_51.713055	*aph(3'')-Ib*	*aph(3'')-Ib*
		aph(6)-Id	*aph(6)-Id*
		bla$_{\text{TEM-1B}}$	*bla*$_{\text{TEM-1}}$
		sul2	*sul2*
	NODE_46_length_602cov_54.182369	*qnrB1*	*qnrB1*
	NODE_49_length_3714_cov_49.786362	*bla*$_{\text{CTX-M-15}}$	*bla*$_{\text{CTX-M-15}}$
	NODE_52_length_2339_cov_48.072944	*aac(6')-Ib-cr*	*aac(6')-Ib-D181Y*
		aac(6')-Ib-cr	
		bla$_{\text{OXA-1}}$	*bla*$_{\text{OXA-1}}$
		catB3	

		catB3	
	NODE_53_length_223cov_29.708406	$bla_{\text{OXA-245}}$	$bla_{\text{OXA-245}}$
	NODE_57_length_1549_cov_52.360734	*catA2*	*catA2*
SRR2724098	NODE_2_length_613775_cov_128.186970	*oqxA*	*oqxA*
		OqxA	
		oqxB	*oqxB*
		OqxB	
	NODE_25_length_86896_cov_168.445585		$ble\text{-}_{\text{MBL}}$
		$bla_{\text{NDM-1}}$	$bla_{\text{NDM-1}}$
	NODE_37_length_36913_cov_175.400234	$bla_{\text{CMY-6}}$	$bla_{\text{CMY-6}}$
	NODE_46_length_9117_cov_202.081646	*aph(3'')-Ib*	*aph(3'')-Ib*
		aph(6)-Id	*aph(6)-Id*
		qnrS1	*qnrS1*
		sul2	*sul2*
	NODE_48_length_5338_cov_147.506237	*aac(6')-Ib-cr*	*aac(6')-Ib11*
		aac(6')-Ib3	
		qacE	
		sul1	*sul1*
	NODE_54_length_3814_cov_158.543260	$bla_{\text{CTX-M-15}}$	$bla_{\text{CTX-M-15}}$
	NODE_55_length_3610_cov_176.890899	*aph(3')-VI*	*aph(3')-VI*
	NODE_57_length_3484_cov_155.265118	*mph(A)*	*mph(A)*
	NODE_6_length_271132_cov_93.448940	$bla_{\text{SHV-182}}$	$bla_{\text{SHV-158}}$
	NODE_60_length_2853_cov_124.769993	*aac(3)-IIa*	*aac(3)-IIe*
	NODE_66_length_1860_cov_131.474899	$bla_{\text{OXA-1}}$	$bla_{\text{OXA-1}}$
		catB3	
		catB3	
	NODE_67_length_1746_cov_167.995676	*catA1*	*catA1*
	NODE_7_length_270452_cov_129.834917	*fosA*	*fosA6*
	NODE_80_length_1187_cov_235.587736	*dfrA14*	*dfrA14*
	NODE_8length_1160_cov_329.421104	*rmtC*	*rmtC*
SRR2724100	NODE_29_length_59380_cov_34.604830	*aadA2*	*aadA2*
		catA1	*catA1*
		dfrA12	*dfrA12*
		mph(A)	*mph(A)*
		qacE	
		sul1	*sul1*
	NODE_44_length_20517_cov_22.787592	$bla_{\text{KPC-2}}$	$bla_{\text{KPC-2}}$
	NODE_50_length_11829_cov_86.187575	*aac(6')-Ib*	*aac(6')-Ib-AKT*
		aac(6')-Ib-cr	
	NODE_7_length_212550_cov_14.572038	*fosA*	*fosA6*
	NODE_84_length_1292_cov_43.473820	*aph(3')-Ia*	*aph(3')-Ia*
	NODE_8length_1443_cov_25.672492	$bla_{\text{OXA-9}}$	$bla_{\text{OXA-9}}$

	NODE_93_length_923_cov_26.522613	*bla*$_{TEM-1A}$	*bla*$_{TEM-1}$
	NODE_96_length_722_cov_40.329412	*bla*$_{SHV-12}$	*bla*$_{SHV-187}$
		bla$_{SHV-129}$	
		bla$_{SHV-13}$	
		bla$_{SHV-155}$	
		bla$_{SHV-172}$	
		bla$_{SHV-31}$	
	NODE_length_453027_cov_13.556308	*oqxA*	*oqxA*
		OqxA	
		oqxB	*oqxB*
		OqxB	
SRR2724109	NODE_17_length_90868_cov_36.462184	*dfrA14*	*dfrA14*
	NODE_22_length_7980_cov_35.337705	*bla*$_{CTX-M-15}$	*bla*$_{CTX-M-15}$
		bla$_{TEM-1B}$	*bla*$_{TEM-1}$
	NODE_24_length_4640_cov_30.407268	*aph(3'')-Ib*	*aph(3'')-Ib*
		aph(6)-Id	*aph(6)-Id*
		sul2	*sul2*
	NODE_26_length_3420_cov_52.327057		*ble*-$_{MBL}$
		bla$_{NDM-7}$	*bla*$_{NDM-7}$
	NODE_28_length_2437_cov_33.034632	*aac(6')-Ib-cr*	*aac(6')-Ib-D181Y*
		aac(6')-Ib-cr	
		bla$_{OXA-1}$	*bla*$_{OXA-1}$
		catB3	
		catB3	
	NODE_3_length_530956_cov_23.428622	*bla*$_{SHV-172}$	*bla*$_{SHV-215}$
		bla$_{SHV-94}$	
		bla$_{SHV-96}$	
	NODE_30_length_2212_cov_35.117026	*qnrB1*	*qnrB1*
	NODE_4_length_428125_cov_27.984544	*oqxA*	*oqxA6*
		OqxA	
		oqxB	*oqxB19*
		OqxB	
	NODE_7_length_359500_cov_28.629043	*fosA*	*fosA6*
SRR2724111	NODE_120_length_1443_cov_19.151976	*bla*$_{OXA-9}$	*bla*$_{OXA-9}$
	NODE_127_length_1292_cov_16.454936	*aph(3')-Ia*	*aph(3')-Ia*
	NODE_134_length_105cov_15.339827	*bla*$_{TEM-1A}$	*bla*$_{TEM-1}$
	NODE_137_length_904_cov_12.355212	*bla*$_{SHV-12}$	
		bla$_{SHV-15}$	
		bla$_{SHV-160}$	
		bla$_{SHV-66}$	
	NODE_3_length_270452_cov_10.317914	*fosA*	*fosA6*

	NODE_54_length_20625_cov_16.344277	*bla*$_{KPC-2}$	*bla*$_{KPC-2}$
	NODE_67_length_16866_cov_13.310054	*aadA2*	*aadA2*
		catA1	*catA1*
		dfrA12	*dfrA12*
		mph(A)	*mph(A)*
		qacE	
		sul1	*sul1*
	NODE_72_length_13275_cov_48.364314	*aac(6')-Ib*	*aac(6')-Ib-AKT*
		aac(6')-Ib-cr	
	NODE_9_length_16354cov_9.403252	*oqxA*	*oqxA*
		OqxA	
		oqxB	*oqxB*
		OqxB	
SRR2724113	NODE_16_length_65999_cov_41.500774	*aac(6')-Im*	*aac(6')-Im*
		aph(2'')-Ib	*aph(2'')-IIa*
	NODE_36_length_8486_cov_33.495155	*aph(3')-Ia*	*aph(3')-Ia*
	NODE_3length_17596_cov_33.313527	*bla*$_{TEM-1B}$	*bla*$_{TEM-1}$
	NODE_46_length_5545_cov_34.378553	*aph(3'')-Ib*	*aph(3'')-Ib*
		aph(6)-Id	*aph(6)-Id*
		sul2	*sul2*
	NODE_52_length_3914_cov_41.385794	*aac(6')-Ib-cr*	*aac(6')-Ib-AKT*
		aac(6')-Ib3	
	NODE_53_length_3777_cov_30.043014	*tet(D)*	*tet(D)*
	NODE_54_length_3612_cov_30.546055	*mph(A)*	*mph(A)*
	NODE_56_length_3028_cov_38.863495	*ARR-3*	*arr-3*
		bla$_{OXA-1}$	*bla*$_{OXA-1}$
		catB3	*catB3*
		qacE	
	NODE_6_length_26378cov_24.827972	*fosA*	*fosA6*
	NODE_65_length_1924_cov_31.313856	*bla*$_{NDM-1}$	*bla*$_{NDM-1}$
	NODE_66_length_1650_cov_39.568615	*catA2*	*catA2*
	NODE_7_length_249686_cov_23.173302	*OqxA*	
		oqxA	*oqxA6*
		OqxB	
		oqxB	*oqxB19*
	NODE_72_length_1182_cov_38.941232	*dfrA14*	*dfrA14*
	NODE_74_length_1127_cov_33.859000	*aadA2b*	*aadA2*
	NODE_77_length_987_cov_91.260465	*sul1*	*sul1*
	NODE_8length_814_cov_34.656477	*dfrA5*	*dfrA5*
	NODE_94_length_538_cov_53.890511	*bla*$_{SHV-155}$	
SRR2724122	NODE_28_length_59380_cov_48.305369	*aadA2*	*aadA2*
		catA1	*catA1*

		dfrA12	*dfrA12*
		mph(A)	*mph(A)*
		qacE	
		sul1	*sul1*
	NODE_32_length_35434_cov_35.770442	*bla*$_{TEM-1A}$	*bla*$_{TEM-1}$
	NODE_38_length_20517_cov_29.322070	*bla*$_{KPC-2}$	*bla*$_{KPC-2}$
	NODE_42_length_12133_cov_97.359404	*aac(6')-Ib*	*aac(6')-Ib-AKT*
		aac(6')-Ib-cr	
	NODE_65_length_1443_cov_31.930851	*bla*$_{OXA-9}$	*bla*$_{OXA-9}$
	NODE_68_length_1292_cov_46.383691	*aph(3')-Ia*	*aph(3')-Ia*
	NODE_7_length_270452_cov_18.746021	*fosA*	*fosA6*
	NODE_78_length_722_cov_52.149580	*bla*$_{SHV-12}$	*bla*$_{SHV-187}$
		bla$_{SHV-129}$	
		bla$_{SHV-13}$	
		bla$_{SHV-155}$	
		bla$_{SHV-172}$	
		bla$_{SHV-31}$	
	NODE_length_485668_cov_17.128298	*oqxA*	*oqxA*
		OqxA	
		oqxB	*oqxB*
		OqxB	
SRR2724123	NODE_100_length_2184_cov_16.993680	*bla*$_{CTX-M-15}$	*bla*$_{CTX-M-15}$
	NODE_107_length_1860_cov_5.275245	*bla*$_{OXA-1}$	*bla*$_{OXA-1}$
		catB3	
		catB3	
	NODE_116_length_1358_cov_10.672624	*bla*$_{TEM-1B}$	*bla*$_{TEM-1}$
	NODE_2_length_350414_cov_7.605779	*fosA*	*fosA_gen*
	NODE_32_length_61479_cov_6.362172	*bla*$_{SHV-106}$	*bla*$_{SHV-212}$
		bla$_{SHV-28}$	
	NODE_74_length_8440_cov_12.055936	*aph(3')-VI*	*aph(3')-VI*
		qnrS1	*qnrS1*
	NODE_80_length_7376_cov_5.243206	*ere(B)*	*ere(B)*
	NODE_82_length_6175_cov_5.057209	*ARR-2*	*arr-2*
		dfrA5	*dfrA5*
		ere(A)	*ere(A)*
		qacE	
		sul1	*sul1*
	NODE_83_length_6020_cov_6.522993	*aph(3'')-Ib*	*aph(3'')-Ib*
		aph(6)-Id	*aph(6)-Id*
		sul2	*sul2*
	NODE_86_length_5739_cov_9.718282		*ble-*$_{MBL}$
		bla$_{NDM-1}$	*bla*$_{NDM-1}$

	NODE_88_length_4774_cov_4.954594	*catA1*	*catA1*
	NODE_92_length_419cov_6.674459	*tet(A)*	*tet(A)*
	NODE_95_length_2854_cov_7.650532	*aac(3)-IIa*	*aac(3)-IIe*
	NODE_97_length_2654_cov_13.007519	*aac(6')-Ib-cr*	*aac(6')-Ib-AKT*
		aac(6')-Ib3	
		aadA1	*aadA1*
		bla$_{\text{OXA-9}}$	*bla*$_{\text{OXA-9}}$
	NODE_length_45023cov_7.509556	*OqxA*	
		oqxA	*oqxA10*
		OqxB	
		oqxB	*oqxB17*
SRR2724129	NODE_106_length_647_cov_30.657095	*bla*$_{\text{TEM-150}}$	
		bla$_{\text{TEM-171}}$	
		bla$_{\text{TEM-1A}}$	
		bla$_{\text{TEM-1C}}$	
		bla$_{\text{TEM-40}}$	
		bla$_{\text{TEM-90}}$	
	NODE_17_length_121618_cov_10.021915	*bla*$_{\text{SHV-172}}$	*bla*$_{\text{SHV-215}}$
		bla$_{\text{SHV-94}}$	
		bla$_{\text{SHV-96}}$	
	NODE_23_length_96616_cov_11.020785	*oqxA*	*oqxA6*
		OqxA	
		oqxB	*oqxB19*
		OqxB	
	NODE_28_length_77865_cov_12.145701	*fosA*	*fosA6*
	NODE_49_length_27563_cov_11.299477	*aph(3'')-Ib*	*aph(3'')-Ib*
		aph(6)-Id	*aph(6)-Id*
		sul2	*sul2*
	NODE_52_length_2219cov_15.780719	*dfrA14*	*dfrA14*
	NODE_65_length_4050_cov_28.574218	*aac(6')-Ib*	*aac(6')-Ib-AKT*
		aac(6')-Ib-cr	
		aadA1	*aadA1*
		bla$_{\text{OXA-9}}$	*bla*$_{\text{OXA-9}}$
	NODE_69_length_3485_cov_34.337318	*bla*$_{\text{CTX-M-15}}$	*bla*$_{\text{CTX-M-15}}$
	NODE_6length_14793_cov_18.053060	*aph(3')-VI*	*aph(3')-VI*
		bla$_{\text{NDM-1}}$	*bla*$_{\text{NDM-1}}$
			ble-$_{\text{MBL}}$
		qnrS1	*qnrS1*
	NODE_85_length_1765_cov_11.591813	*qnrB1*	*qnrB1*
SRR2724130	NODE_10_length_146270_cov_10.473108	*bla*$_{\text{SHV-12}}$	*bla*$_{\text{SHV-12}}$
	NODE_109_length_1299_cov_22.327170	*bla*$_{\text{OXA-9}}$	*bla*$_{\text{OXA-9}}$
	NODE_117_length_984_cov_122.832078	*aac(6')-Ib*	*aac(6')-Ib-AKT*

		aac(6')-Ib-cr	
	NODE_122_length_779_cov_24.233425	*bla*$_{\text{TEM-122}}$	
		bla$_{\text{TEM-163}}$	
		bla$_{\text{TEM-168}}$	
		bla$_{\text{TEM-1A}}$	*bla*$_{\text{TEM-122}}$
	NODE_24_length_84692_cov_10.834044	*fosA*	*fosA6*
	NODE_26_length_77566_cov_19.244843	*bla*$_{\text{KPC-2}}$	*bla*$_{\text{KPC-2}}$
	NODE_2length_89748_cov_10.519483	*oqxA*	*oqxA*
		OqxA	
		oqxB	*oqxB*
		OqxB	
SRR2724134	NODE_26_length_59380_cov_18.414342	*aadA2*	*aadA2*
		catA1	*catA1*
		dfrA12	*dfrA12*
		mph(A)	*mph(A)*
		qacE	
		sul1	*sul1*
	NODE_4length_20625_cov_18.009025	*bla*$_{\text{KPC-2}}$	*bla*$_{\text{KPC-2}}$
	NODE_6_length_270452_cov_19.082146	*fosA*	*fosA6*
	NODE_72_length_1443_cov_21.540274	*bla*$_{\text{OXA-9}}$	*bla*$_{\text{OXA-9}}$
	NODE_75_length_1292_cov_18.158798	*aph(3')-Ia*	*aph(3')-Ia*
	NODE_80_length_1128_cov_150.916084	*aac(6')-Ib*	*aac(6')-Ib-AKT*
		aac(6')-Ib-cr	
	NODE_86_length_923_cov_18.659548	*bla*$_{\text{TEM-1A}}$	*bla*$_{\text{TEM-1}}$
	NODE_93_length_722_cov_35.601681	*bla*$_{\text{SHV-12}}$	*bla*$_{\text{SHV-187}}$
		bla$_{\text{SHV-129}}$	
		bla$_{\text{SHV-13}}$	
		bla$_{\text{SHV-155}}$	
		bla$_{\text{SHV-172}}$	
		bla$_{\text{SHV-31}}$	
	NODE_length_489037_cov_16.829883	*OqxA*	
		oqxA	*oqxA*
		OqxB	
		oqxB	*oqxB*
SRR2724135	NODE_14_length_137810_cov_79.749177	*aac(6')-Ib-cr*	*aac(6')-Ib11*
		aac(6')-Ib3	*ble*-$_{\text{MBL}}$
		bla$_{\text{CMY-6}}$	*bla*$_{\text{CMY-6}}$
		bla$_{\text{NDM-1}}$	*bla*$_{\text{NDM-1}}$
		qacE	
		rmtC	*rmtC*
		sul1	*sul1*
	NODE_3_length_606979_cov_50.387960	*oqxA*	*oqxA*

		OqxA	
		oqxB	*oqxB*
		OqxB	
	NODE_6_length_349148_cov_54.932801	*fosA*	*fosA6*
	NODE_length_666615_cov_39.606872	*bla*$_{SHV-110}$	*bla*$_{SHV-110}$
		bla$_{SHV-81}$	
SRR2724137	NODE_12_length_18044cov_30.093803	*bla*$_{SHV-106}$	*bla*$_{SHV-106}$
		bla$_{SHV-28}$	
	NODE_13_length_174875_cov_40.133964	*OqxA*	
		oqxA	*oqxA6*
		OqxB	
		oqxB	*oqxB20*
	NODE_37_length_8135_cov_53.941808	*tet(A)*	*tet(A)*
	NODE_38_length_698cov_70.768310	*dfrA14*	*dfrA14*
	NODE_4_length_350007_cov_42.890251	*fosA*	*fosA6*
	NODE_40_length_5889_cov_53.291392	*aph(3'')-Ib*	*aph(3'')-Ib*
		aph(6)-Id	*aph(6)-Id*
		bla$_{TEM-1B}$	*bla*$_{TEM-1}$
		sul2	*sul2*
	NODE_45_length_2987_cov_66.957692	*bla*$_{CTX-M-15}$	*bla*$_{CTX-M-15}$
	NODE_46_length_223cov_85.662850	*bla*$_{OXA-162}$	*bla*$_{OXA-162}$
	NODE_4length_5803_cov_75.209831	*qnrB1*	*qnrB1*
SRR2724138	NODE_12_length_142768_cov_16.885033	*bla*$_{SHV-110}$	*bla*$_{SHV-110}$
		bla$_{SHV-81}$	*bla*$_{TEM-150}$
	NODE_27_length_72682_cov_19.809634	*oqxA*	*oqxA*
		OqxA	
		oqxB	*oqxB*
		OqxB	
	NODE_4_length_270780_cov_21.868780	*fosA*	*fosA6*
	NODE_4length_39608_cov_26.727844	*aph(3'')-Ib*	*aph(3'')-Ib*
		aph(3'')-Ib	
		aph(3'')-Ib	
		aph(3'')-Ib	
		aph(6)-Id	*aph(6)-Id*
		tet(B)	*tet(B)*
	NODE_64_length_5345_cov_18.560943	*aadA2b*	*aadA2*
		qacE	
		sul1	*sul1*
	NODE_65_length_4800_cov_387.684357	*aac(6')-Ib*	*aac(6')-Ib-AKT*
		aac(6')-Ib-cr	
		aadA1	*aadA1*
		bla$_{OXA-9}$	*bla*$_{OXA-9}$

		bla$_{\text{TEM-1A}}$	
	NODE_70_length_2966_cov_49.234590	*qnrB19*	*qnrB19*
	NODE_7length_295cov_47.581445	*bla*$_{\text{KPC-2}}$	*bla*$_{\text{KPC-2}}$
SRR2724139	NODE_24_length_51072_cov_19.209186	*aadA2*	*aadA2*
		catA1	*catA1*
		dfrA12	*dfrA12*
		mph(A)	*mph(A)*
		qacE	
		sul1	*sul1*
	NODE_3length_37143_cov_23.660201	*bla*$_{\text{OXA-9}}$	*bla*$_{\text{OXA-9}}$
		bla$_{\text{TEM-1A}}$	*bla*$_{\text{TEM-1}}$
	NODE_42_length_20517_cov_20.580432	*bla*$_{\text{KPC-2}}$	*bla*$_{\text{KPC-2}}$
	NODE_7_length_270452_cov_17.725138	*fosA*	*fosA6*
	NODE_78_length_1292_cov_19.025751	*aph(3')-Ia*	*aph(3')-Ia*
	NODE_89_length_722_cov_47.759664	*bla*$_{\text{SHV-12}}$	*bla*$_{\text{SHV-187}}$
		bla$_{\text{SHV-129}}$	
		bla$_{\text{SHV-13}}$	
		bla$_{\text{SHV-155}}$	
		bla$_{\text{SHV-172}}$	
		bla$_{\text{SHV-31}}$	
	NODE_length_49211cov_16.090785	*oqxA*	*oqxA*
		OqxA	
		oqxB	*oqxB*
		OqxB	
SRR2724140	NODE_28_length_59380_cov_16.480668	*aadA2*	*aadA2*
		catA1	*catA1*
		dfrA12	*dfrA12*
		mph(A)	*mph(A)*
		qacE	
		sul1	*sul1*
	NODE_29_length_57736_cov_24.142339	*bla*$_{\text{KPC-2}}$	*bla*$_{\text{KPC-2}}$
	NODE_44_length_13275_cov_98.381959	*aac(6')-Ib*	*aac(6')-Ib-AKT*
		aac(6')-Ib-cr	
	NODE_6_length_270452_cov_13.967521	*fosA*	*fosA6*
	NODE_7_length_261624_cov_11.457294	*oqxA*	*oqxA*
		OqxA	
		oqxB	*oqxB*
		OqxB	
	NODE_78_length_1443_cov_16.886778	*bla*$_{\text{OXA-9}}$	*bla*$_{\text{OXA-9}}$
	NODE_8length_1292_cov_13.056652	*aph(3')-Ia*	*aph(3')-Ia*
	NODE_90_length_923_cov_15.989950	*bla*$_{\text{TEM-1A}}$	*bla*$_{\text{TEM-1}}$
	NODE_96_length_722_cov_34.337815	*bla*$_{\text{SHV-12}}$	*bla*$_{\text{SHV-187}}$

		bla$_{SHV-129}$	
		bla$_{SHV-13}$	
		bla$_{SHV-155}$	
		bla$_{SHV-172}$	
		bla$_{SHV-31}$	
