# Supplementary material for: Pipeline validation for the identification of antimicrobial-resistant genes in carbapenem-resistant Klebsiella pneumoniae
Source: Sci Rep. 2023 Sep 14;13:15189. doi: 10.1038/s41598-023-42154-6 (PMC10502106; doi:10.1038/s41598-023-42154-6)
Supplement: Supplementary file 4 — Supplementary Information 4. [file 41598_2023_42154_MOESM4_ESM.pdf]

Table S4 – SRAs used in this study.

Bioproject	SRA accession
PRJEB28660	ERR2796902
PRJEB28660	ERR2796903
PRJEB28660	ERR2796904
PRJEB28660	ERR2796907
PRJEB28660	ERR2796910
PRJEB28660	ERR2796911
PRJEB28660	ERR2796912
PRJEB28660	ERR2796917
PRJEB28660	ERR2796919
PRJEB28660	ERR2796920
PRJEB28660	ERR2796923
PRJEB28660	ERR2796926
PRJEB28660	ERR2796929
PRJEB28660	ERR2796930
PRJEB28660	ERR2796931
PRJEB28660	ERR2796942
PRJEB28660	ERR2796944
PRJEB28660	ERR2796946
PRJEB28660	ERR2796948
PRJEB28660	ERR2796949
PRJEB28660	ERR2796950
PRJEB28660	ERR2796952
PRJEB28660	ERR2796953
PRJEB28660	ERR2796955
PRJEB28660	ERR2796956
PRJEB28660	ERR2796957
PRJEB28660	ERR2796958
PRJEB28660	ERR2796961
PRJEB28660	ERR2796963
PRJEB28660	ERR2796964
PRJEB28660	ERR2796969
PRJEB28660	ERR2796976
PRJEB28660	ERR2796977
PRJEB28660	ERR2796978
PRJEB28660	ERR2796981
PRJEB28660	ERR2796983
PRJEB28660	ERR2796984
PRJEB28660	ERR2796988
PRJEB28660	ERR2796989
PRJEB28660	ERR2796990

PRJEB28660	ERR2796992
PRJEB28660	ERR2796993
PRJEB28660	ERR2796994
PRJEB28660	ERR2796995
PRJEB28660	ERR2796996
PRJEB28660	ERR2796998
PRJEB28660	ERR2797002
PRJEB28660	ERR2797003
PRJEB28660	ERR2797004
PRJEB28660	ERR2797005
PRJEB28660	ERR2797006
PRJEB28660	ERR2797007
PRJEB28660	ERR2797008
PRJEB28660	ERR2797009
PRJEB28660	ERR2797010
PRJEB28660	ERR2797011
PRJNA292904	SRR3242012
PRJNA292904	SRR4025850
PRJNA292904	SRR4025851
PRJNA292904	SRR4025861
PRJNA292904	SRR4025863
PRJNA292902	SRR4025977
PRJNA292902	SRR4025979
PRJNA292902	SRR4025980
PRJNA292902	SRR4025983
PRJNA292902	SRR4025984
PRJNA292902	SRR4025985
PRJNA292902	SRR4025987
PRJNA292902	SRR4025990
PRJNA292902	SRR4025991
PRJNA292902	SRR4025992
PRJNA292902	SRR4025993
PRJNA292902	SRR4025994
PRJNA292902	SRR4025996
PRJNA292902	SRR4026000
PRJNA292902	SRR4026003
PRJNA292902	SRR5122322
PRJNA292904	SRR5146462
PRJNA292904	SRR5146463
PRJNA292904	SRR5167852
PRJNA292904	SRR5167853
PRJNA292904	SRR5168221
PRJNA292904	SRR5168222

PRJNA292904	SRR5168231
PRJNA292904	SRR5168232
PRJNA292904	SRR5168235
PRJNA292904	SRR5168236
PRJNA292902	SRR5168243
PRJNA292902	SRR5168244
PRJNA292902	SRR5168370
PRJNA292902	SRR5168371
PRJNA292902	SRR5168372
PRJNA292902	SRR5168375
PRJNA292902	SRR5168376
PRJNA292902	SRR5168377
PRJNA292902	SRR5168378
PRJNA292902	SRR5168384
PRJNA292902	SRR5168385
PRJNA292902	SRR5168386
PRJNA292902	SRR5168387
PRJNA292902	SRR5168388
PRJNA292902	SRR5168389
PRJNA292902	SRR5168390
PRJNA292902	SRR5168393
PRJNA292902	SRR5168394
PRJNA292902	SRR5168481
PRJNA292902	SRR5168482
PRJNA292902	SRR5168483
PRJNA292902	SRR5168485
PRJNA292902	SRR5168486
PRJNA292902	SRR5168488
PRJNA292902	SRR5168489
PRJNA292902	SRR5168490
PRJNA292902	SRR5168491
PRJNA292902	SRR5168492
PRJNA292902	SRR5168493
PRJNA292902	SRR5168496
PRJNA292902	SRR5168497
PRJNA292902	SRR5168498
PRJNA292902	SRR5168509
PRJNA292902	SRR5168510
PRJNA292902	SRR5168511
PRJNA292902	SRR5168517
PRJNA292902	SRR5168518
PRJNA292902	SRR5168519
PRJNA295003	SRR2724077

PRJNA295003	SRR2724078
PRJNA295003	SRR2724081
PRJNA295003	SRR2724082
PRJNA295003	SRR2724083
PRJNA295003	SRR2724085
PRJNA295003	SRR2724086
PRJNA295003	SRR2724087
PRJNA295003	SRR2724088
PRJNA295003	SRR2724089
PRJNA295003	SRR2724090
PRJNA295003	SRR2724091
PRJNA295003	SRR2724092
PRJNA295003	SRR2724093
PRJNA295003	SRR2724096
PRJNA295003	SRR2724097
PRJNA295003	SRR2724098
PRJNA295003	SRR2724100
PRJNA295003	SRR2724109
PRJNA295003	SRR2724111
PRJNA295003	SRR2724113
PRJNA295003	SRR2724122
PRJNA295003	SRR2724123
PRJNA295003	SRR2724129
PRJNA295003	SRR2724130
PRJNA295003	SRR2724134
PRJNA295003	SRR2724135
PRJNA295003	SRR2724137
PRJNA295003	SRR2724138
PRJNA295003	SRR2724139
PRJNA295003	SRR2724140
PRJNA307517	SRR8607448
PRJNA307517	SRR8607449
PRJNA307517	SRR8607450
PRJNA307517	SRR8607451
PRJNA307517	SRR8607452
PRJNA307517	SRR8607453
PRJNA307517	SRR8607454
PRJNA307517	SRR8607455
PRJNA307517	SRR8607456
PRJNA307517	SRR8607457
PRJNA307517	SRR8607458
PRJNA307517	SRR8607459
PRJNA307517	SRR8607460

PRJNA307517	SRR8607461
PRJNA307517	SRR8607462
PRJNA307517	SRR8607463
PRJNA307517	SRR8607464
PRJNA307517	SRR8607465
PRJNA307517	SRR8607466
PRJNA307517	SRR8607467
PRJNA307517	SRR8607468
PRJNA307517	SRR8607470
PRJNA307517	SRR8607471
PRJNA308116	SRR4115668
PRJNA308116	SRR5132378
PRJNA308116	SRR5132379
PRJNA308116	SRR5132381
PRJNA308116	SRR5132447
PRJNA308116	SRR5132448
PRJNA308116	SRR5132449
PRJNA308116	SRR5132450
PRJNA308116	SRR5132451
PRJNA308116	SRR5132452
PRJNA308116	SRR5132453
PRJNA392824	SRR5809251
PRJNA392824	SRR5809254
PRJNA392824	SRR5809260
PRJNA392824	SRR5809261
PRJNA392824	SRR5809262
PRJNA392824	SRR5809264
PRJNA392824	SRR5809265
PRJNA392824	SRR5809266
PRJNA392824	SRR5809267
PRJNA392824	SRR5809268
PRJNA279657	SRR3465532
